# Isolable Phosphonioacetylides as Strong Neutral Carbon Donors with Variable Steric Demand and Tunable Donor Properties

**DOI:** 10.1021/acs.inorgchem.6c00919

**Published:** 2026-05-12

**Authors:** Franka Brylak, Liane Perktold, Lucas C. Torres, Luis Stechele, Klaus Wurst, Michael Seidl, Christopher B. Caputo, Fabian Dielmann

**Affiliations:** † Institute of General, Inorganic and Theoretical Chemistry, Universität Innsbruck, Innsbruck 6020, Austria; ‡ Department of Chemistry, York University, 4700 Keele St, Toronto, Ontario M3J 1P3, Canada

## Abstract

Phosphonioacetylides are rod-shaped, neutral carbon donors with ambiphilic character at the terminal carbon. Despite their analogy to CO and isocyanides, they remain underexplored due to their high reactivity, and only one isolable free phosphonioacetylide has been reported. Here, we describe two new isolable, room-temperature-persistent examples stabilized either by two N-heterocyclic imines (NHIs) bearing bulky, flexible *tert*-octyl groups or by a single dipp-substituted NHI combined with alkyl substituents. Comprehensive characterization (SCXRD, NMR, IR, MS, DFT) confirms their strong donor properties and the accessibility of the reactive C_2_ unit. DFT analysis quantify how the substituents at phosphorus modulate the frontier molecular orbital energies, spanning a wide range of σ and π-donor strengths and π-acceptor abilities, with trends that mirror those of the corresponding phosphines. Both phosphonioacetylides readily form Au^I^ complexes, whereas the less electron-rich compound shows the tendency to eliminate its C_2_ moiety thermally or upon hydrolysis. These results establish design principles for stabilizing and tuning phosphonioacetylides and open avenues for their use in coordination chemistry and catalysis.

## Graphical abstract

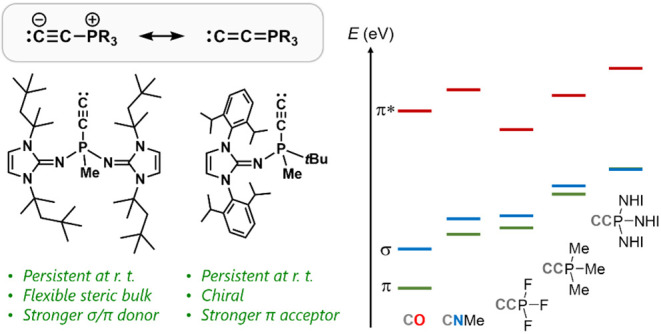

## Introduction

Neutral, rod-shaped carbon ligands such as carbon monoxide and isocyanides (RNC) are utilized in numerous fields of organometallic chemistry as reactants, as spectroscopic probes, and as steering ligands in transition metal catalysis.^1–9^ Carbon monoxide is a relatively weak σ-donor but worshiped for its strong π-acidity, whereas isocyanides generally combine stronger σ-donor ability with attenuated π-acidity, while having the advantage of tunable stereoelectronic properties via the R substituent.^10–15^ However, they exhibit significantly weaker donor properties than sp^2^- or sp^3^-hybridized neutral carbon ligands such as N-heterocyclic carbenes (NHCs) or ylides,^16^ which have become indispensable not only as ligands in coordination chemistry and catalysis but also as organocatalysts and reagents in synthesis.^17–20^

Phosphonioacetylides represent a conceptually intriguing and little-explored extension of this family of neutral, sp-hybridized carbon donor ligands. Both DFT analysis and Tolman electronic parameter (TEP) measurements indicate that these species are markedly stronger σ-donors than classical isocyanides,^21–23^ with TEP values placing phosphonioacetylides in the range of NHCs in terms of net electron-donating ability.^21^ The first metal complex featuring a triphenylphosphonioacetylide ligand **A** was reported in 1970 via *in situ* ligand conversion at the metal center,^24,25^ and Bestmann subsequently provided NMR spectroscopic evidence for the persistence of free **A** at low temperature.^26–29^ Only recently, Ong, Zhao, Frenking, and co-workers isolated the first bench-stable phosphonioacetylide **B** by installing bulky, electron-donating N-heterocyclic imine (NHI) substituents that shield the reactive PC_2_ unit.^30^ Despite the steric encumbrance, the linear C_2_ fragment remains sufficiently accessible to engage in coordination and to function as an ancillary ligand in catalysis.^23^

We have recently shown that reducing the steric profile of the NHI substituent by replacing 2,4-diisopropylphenyl (dipp) with *tert*-butyl (*t*Bu) groups at the endocyclic N atoms gives phosphonioacetylide **C** which exhibits diminished thermal stability. While the free ligand is not persistent at ambient temperature, its lithium and potassium salts are isolable at room temperature and serve as competent transmetalation precursors.^21^ Motivated by these findings and as part of our ongoing efforts to exploit NHI substituents for generating electron-rich ligands including phosphines,^31–34^ pyridines,^35–37^ and carbenes,^38,39^ we sought to explore how substituent variation governs the stability and donor properties of phosphonioacetylides. Herein, we report two new phosphonioacetylides that are isolable crystalline solids and persistent at ambient temperature and feature an accessible C_2_ unit. We analyze by DFT how substituent effects modulate the electronic properties of this little-explored class of rod-shaped carbon ligands.

## Results and Discussion

### Synthesis and Characterization of Phosphonioacetylides **6** and **10**

To access new room-temperature-persistent phosphonioacetylides, we varied both steric and electronic features guided by the previous findings (Figure 1). Two structural modifications were pursued: (i) replacing *tert*-butyl groups with conformationally flexible *tert*-octyl (*t*Oct) groups on the NHI to increase steric protection without altering the electronics at phosphorus and (ii) enhancing electrophilicity at phosphorus by employing only one NHI substituent together with two alkyl groups (*t*Bu and Me).

**Figure 1:**
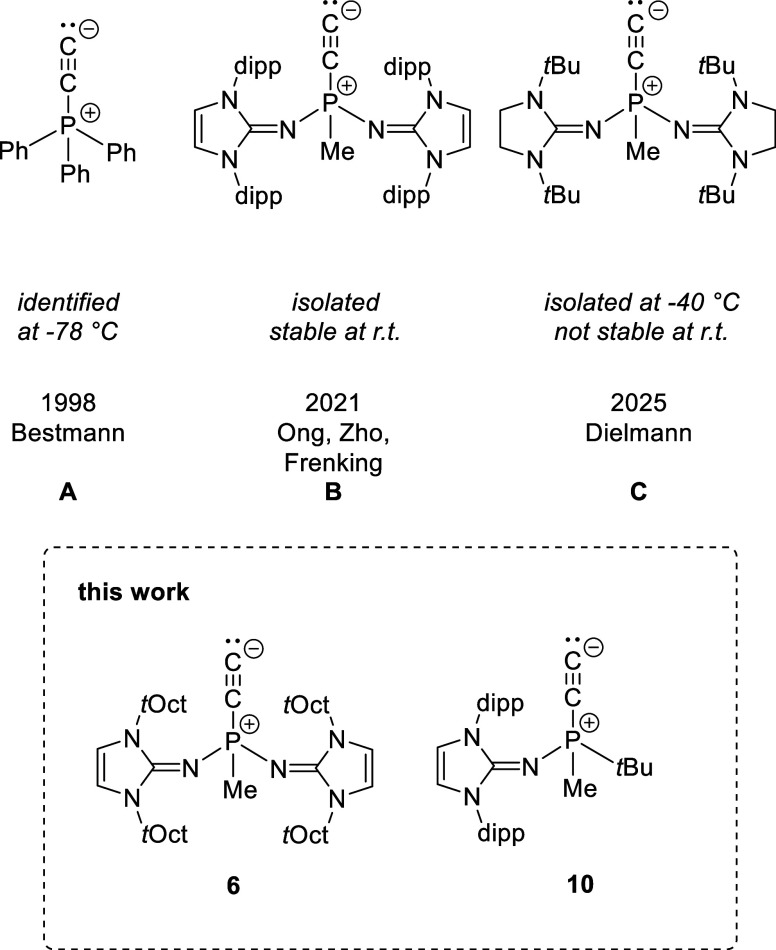
Previously reported phosphonioacetylides (A–C) and two new isolable examples introduced in this work.

The synthesis of phosphonioacetylide **6** starts with the imidazolium salt I*t*Oct·HBF_4_ reported by Szostak and co-workers.^40^ Deprotonation furnished the free carbene **1**, which was isolated and fully characterized. A single-crystal X-ray diffraction (SCXRD) study reveals that for steric reasons, the “dangling” *t*Bu group resides above or below the imidazole plane, leading to a notably bulky carbene. This congestion becomes further evident in the subsequent Staudinger-type reaction with trimethylsilyl azide to NHI **2** after N_2_ elimination. While the corresponding *t*Bu derivative converts within 72 h under reflux in toluene,^41,42^ formation of **2** required 15 days under identical conditions. Further indication of steric strain shows the solid-state structure, displaying a nearly linear C1–N1–Si1 angle of 169.52(9)°. Combining NHI **2** with PCl_3_ gave the phosphenium salt **3** as a bright yellow solid (90% yield) with the “dangling” *t*Bu groups positioned above and below the NHI plane, flanking the low-coordinate P center. Following the reported sequence used for **B** and **C**, treatment of **3** with ethynyl magnesium chloride afforded phosphine **4**, which upon methylation with iodomethane furnished phosphonium salt **5**. Deprotonation of **5** with KHMDS at −40 °C yielded phosphonioacetylide **6** after low-temperature workup. In contrast to **C**, compound **6** is stable at room temperature, indicating that four *t*Oct groups provide more effective steric protecting of the reactive PC_2_ moiety than *t*Bu groups, which can also be recognized in the solid-state structures of the *t*Oct derivatives (Figure 2). The overall yield starting from I*t*Oct·HBF_4_ was 33%.

**Figure 2:**
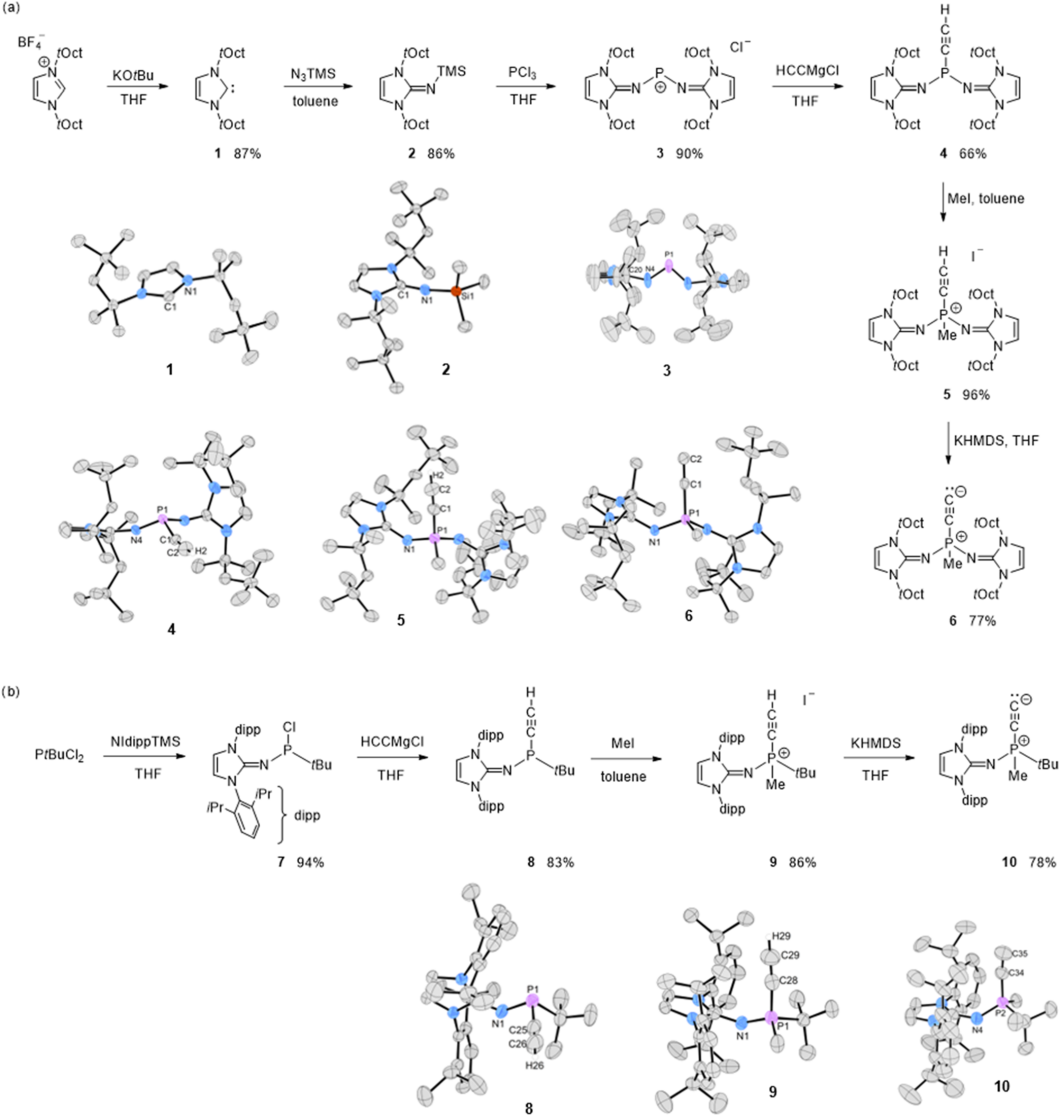
Synthesis of phosphonioacetylides **6** (a) and **10** (b). Molecular structures of **1**-**6** (a) and **8**-**10** (b) in the solid state; hydrogen atoms except the hydrogen attached at C_β_ and counterions (chloride for **3**, iodide for **5** and **9**) are omitted for clarity. Ellipsoids are displayed at 50% probability. Selected structural data are shown in Table 1.

The reaction sequence can be conveniently tracked by ^31^P NMR spectroscopy. The highly deshielded resonance of **3** (278.7 ppm) is shifted to lower frequency for phosphine **4** (63.0 ppm). The signal appears as a singlet because the small coupling to the alkynyl proton (^3^ *J*_PH_ = 3 Hz) is not resolved. For the phosphonium cation **5**, the ^31^P signal is a doublet of quartets at −34.5 ppm (^3^ *J*_PH_ = 10 Hz, ^2^ *J*_PH_ = 13 Hz). After deprotonation, **6** shows a quartet at −37.4 ppm (^2^ *J*_PH_ = 13 Hz), and the absence of an alkynyl ^3^ *J*_PH_ confirms the formation of the phosphonioacetylide. The ^13^C resonances of the C_2_ unit in **6** appear as two doublets for C_α_ at 105.5 ppm (^1^ *J*_PC_ = 143 Hz) and C_β_ at 216.4 ppm (^2^ *J*_PC_ = 16 Hz), comparable to those of **B** (C_α_: 98.9 ppm, ^1^ *J*_CP_ = 141 Hz; C_β_: 208.5 ppm)^30^ and significantly downfield from the corresponding carbons in phosphonium salt **5** (C_α_: 83.5 ppm, ^1^ *J*_CP_ = 171 Hz; C_β_: 93.2 ppm, ^2^ *J*_CP_ = 29 Hz).

SCXRD measurements reveal that the CC bond distance is slightly shortened when converting the P^III^ compound **4** (1.176(10) Å) into P^V^ cation **5** (1.153(10) Å) but is significantly elongated for phosphonioacetylide **6** (1.226(2) Å) lying within the range of an elongated triple bond (C≡C: 1.20 Å, C=C: 1.34 Å).^43,44^

The second phosphonioacetylide **10** was prepared by reacting PCl_2_ *t*Bu with the NHI TMSNIdipp to give phosphine **7** via elimination of TMSCl. In contrast to **3**, chloride remains bound to phosphorus, indicating a more electrophilic phosphorus center when only one NHI donor is present. Introduction of the C_2_ unit with ethynyl magnesium chloride followed by methylation with methyl iodide gave **9**, which upon deprotonation furnished phosphonioacetylide **10** in 52% overall yield. As above, the transformations are readily monitored by characteristic ^31^P resonances (Table 1). For **10**, the ^13^C signals of the C_2_ unit appear as doublets for C_α_ at 105.5 ppm (^1^ *J*_PC_ = 143 Hz, C_6_D_6_) and C_β_ at 231.8 ppm (^2^ *J*_PC_ = 17 Hz, C_6_D_6_). Notably, the terminal β carbon resonance is significantly deshielded compared to those of **6** (216.4 ppm, C_6_D_6_), **B** (208.5 ppm, THF-*d*_8_),^30^ and **A** (228.9 ppm, CD_2_Cl_2_),^28,29^ consistent with an increased carbene character at the terminal carbon due to diminished P→C_2_ charge migration, although it remains unclear to what extent solvent effects may influence the chemical shifts. Because phosphorus in **10** bears four different substituents, the product is formed as a racemic mixture. SCXRD reveals an asymmetric unit containing two crystallographically independent molecules, each comprising both enantiomers in positional disorder, with C–C bond lengths ranging from 1.214 to 1.242 Å, comparable to **B** (1.237(4) Å)^30^ and **6** (1.226(2) Å). The infrared (IR) stretching frequency for **10** at 1936.7 cm^–1^ is slightly red-shifted relative to **6** (1947.2 cm^–1^) and **B** (1948 cm^–1^),^30^ consistent with a reduced CC bond order arising from enhanced backbonding into the more electrophilic phosphorus center.

**Table 1:** Selected Structural and Spectroscopic Data for Compounds **3**–**10**, **13**, and **14** ^a^,^f^

	**3**	**4**	**5**	**6**	**7**	**8**	**9**	**10**	**13**	**14**
C_α_≡C_β_		1.18(1)^b^	1.15(1)	1.226(2)		1.177(5)^b^	1.193(6)	1.232(6)^c^		1.219(9)^b^
C_α_–P		1.79(1)^b^	1.764(7)	1.729(2)		1.790(4)^b^	1.733(4)	1.713(2)^c^		1.702(9)^b^
P–C_α_-C_β_		168(1)^b^	174.2(6)	179.2(2)		176.3(3)^b^	174.6(4)	168.5(3)^c^		173.9(7)^b^
%*V*_bur_			23.1	25.6			28.3	22.0^b^		24.6
δ(^31^P)	278.7 (s)^d^	63.0 (s)^e^	–34.5 (qd)^d^	–37.4 (q)^e^	157.6 (dec)^e^	34.4 (dh)^e^	11.2 (m)^d^	–2.6 (m)^e^	–33.5 (q)^d^	8.0 (m)^d^
δ(^13^C) C_α_		96.4^e^	83.5^d^	105.5^e^		89.2^e^	72.8^d^	90.8^e^	98.4^d^	86.7^d^
δ(^13^C) C_β_		86.1^e^	93.2^d^	216.4^e^		89.3^e^	98.9^d^	231.8^e^	156.9^d^	163.5^d^

a Bond lengths (Å) and bond angles (°) of the solid-state structures shown in Figure 2, calculated buried volumes^e^, and ^31^P and ^13^C­{^1^H} NMR chemical shifts (ppm).

b Bond length/angle chosen with lowest amount of disorder.

c Averaged (weighted mean).

d Recorded in CD_3_CN,

e Recorded in C_6_D_6_,

f Buried volume (*%V*_bur_) was calculated with the SambVca 2.1 web tool,^45–47^ and the following parameters were used: Bondi radii scaled by 1.17, sphere radius = 3.5, C_β_ selected to coordinate to center of the sphere with distance of 2.0, mesh spacing for numerical integration = 0.1, H atoms not included.

### Reactivity of Phosphonioacetylides **6** and **10**

Both phosphonioacetylides **6** and **10** are air-sensitive, crystalline solids that remain intact for months at ambient temperature under inert atmosphere. Slow decomposition is observed when solutions of **6** or **10** are heated at 90 °C for several hours (Figures S97–S102). For **6**, ^1^H and ^31^P NMR monitoring reveals the formation of multiple products. In contrast, **10** reacts more selectively to give phosphine (NHI)­(*t*Bu)­MeP (**11**) as the major product via formal elimination of the C_2_ unit. This behavior contrasts with the CH insertion followed by dipp migration reported for **B**,^30^ despite the greater ambiphilicity anticipated for **10**. The restricted rotation of the NHI group, which may hinder approach of the C_2_ fragment at the isopropyl CH bond, and the better accessibility of the C_2_ unit enabling intermolecular reactions could account for the different outcome. However, the mechanism of this C_2_ cleavage and the identity of any external acceptor remain to be elucidated. The reactions with water parallel these trends: **6** reacts unselectively to afford several phosphorus-containing products (Figure S103), whereas **10** cleanly yields phosphine oxide (NHI)­(*t*Bu)­MePO (**12**) with concomitant acetylene elimination detected by ^1^H and ^13^C NMR spectroscopy. The formation of **12** was confirmed by SCXRD measurements and by independent synthesis from **11** and N_2_O. Notably, the lithium-stabilized phosphonioacetylide **C** reacts with water by simple protonation at the terminal carbon without C_2_ cleavage, presumably because Li^+^ stabilizes hydroxide and suppresses fragmentation.^21^

In previous studies, the Tolman electronic parameter (TEP) placed phosphonioacetylides within the range of classical NHCs in terms of overall donor strength.^21^ To more selectively probe σ donation, we employed the Huynh electronic parameter (HEP), which gauges the σ donor ability of a ligand L from the influence of L on the carbene ^13^C NMR resonance in square-planar Pd­(II) complexes *trans*-[PdBr_2_(iPr_2_-bimy)­(L)] (iPr_2_-bimy = 1,3-diisopropylbenzimidazolin-2-ylidene).^48^ Because carbon donors with NHC-like strength can promote cis/trans isomerization in these Pd­(II) systems, we instead analyzed linear Au­(I) complexes [Au­(iPr_2_-bimy)­(L)]^+^, for which the ^13^C carbene chemical shift correlates with HEP via the established relationship HEP = 1.19­[Au]–45.0, where [Au] is the carbene ^13^C shift (ppm) in [Au­(iPr_2_-bimy)­(L)]^+^.^49^ Treating **6** or **10** with [AuCl­(iPr_2_-bimy)] afforded mixtures of homoleptic and heteroleptic cationic Au­(I) complexes, formulated as [Au­(L^1^)­(L^2^)]Cl (L^1^, L^2^ = iPr_2_-bimy, **6** or **10**; Figure 3), a common outcome for strong carbon donors.^49,50^ With **10**, the desired heteroleptic complex **16** formed in appreciable amounts, while the reaction with **6** predominantly afforded the homoleptic salts **13** and [Au­(*i*Pr_2_-bimy)_2_]­Cl, with only trace **15**. Consequently, the reporter carbene signal for **15** could not be observed, likely due to its very low concentration and broadening from ^4^ *J*_CP_ coupling. Attempts to isolate the heteroleptic species **16** via anion exchange to PF_6_^–^ with KPF_6_, alumina column chromatography (CH_2_Cl_2_/MeOH), or recrystallization from typical solvents were unsuccessful. Nonetheless, in the crude mixture from **10**, the carbene ^13^C resonance of the heteroleptic complex **16** was unambiguously identified at δ_C_ = 188.9 ppm. Applying the above-mentioned correlation gave an HEP value of 177.2 ppm for **10**, placing its σ-donor strength in the NHC regime (*cf*. IPr, HEP = 177.5 ppm; IPr = 1,3-bis­(2,6-diisopropylphenyl)­imidazolin-2-ylidene). Because the heteroleptic complexes could not be isolated, we prepared the homoleptic complexes [Au­(**6**)_2_]Cl (**13**) and [Au­(**10**)_2_]Cl (**14**) by treating [AuCl­(tht)] with two equivalents of **6** and **10**, respectively. Coordination to Au­(I) is accompanied by an approximately 5 ppm shift of the ^31^P resonance to higher frequency (**13**: −33.5 ppm, **14**: 8.0 and 8.0 ppm). Notably, **14** is obtained as the mixture of diastereomers due to the racemic nature of **10**. Percent buried volumes (*V*_bur_)^45–47^ derived from all available solid-state structures (Table 1, Figures 2 and 3) fall in similar ranges for **6** (23.1–25.6%) and **10** (22.0–28.3%), substantially lower than **B** (32.6%)^23^ but higher than **C** (18.2–20.5%).^21^ These steric metrics underline that **6** and **10** are less bulky than **B**, potentially enabling broader reactivity while retaining sufficient protection for persistence at room temperature.

**Figure 3:**
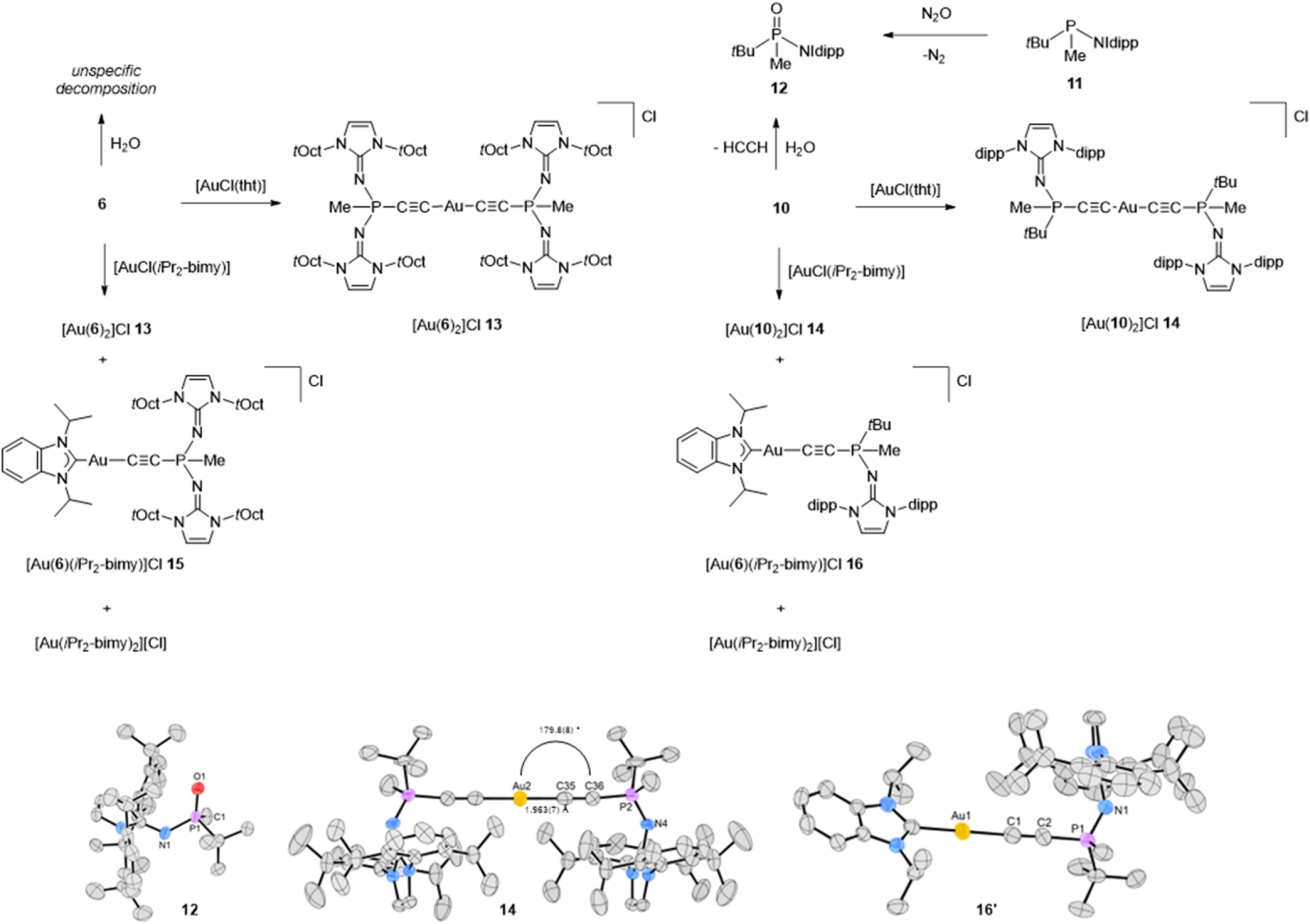
Reactions of phosphonioacetylides **6** and **10** with H_2_O, [AuCl­(tht)], and [AuCl­(iPr_2_-bimy)], and the synthesis of phosphine oxide **12** from phosphine **11**. Molecular structures of **12**_,_ **14** and **16′** in the solid state; hydrogen atoms, the chloride counterion of **14**, and the PF_6_ counterion of **16′** are omitted for clarity. Ellipsoids are displayed at 50% probability. Selected structural data are shown in Table 1.

### Theoretical Studies

To dissect how the substituents at phosphorus modulate the donor–acceptor properties in phosphonioacetylides, Hill and co-workers previously evaluated the energies of the key interaction orbitals (π-donor, σ-donor, and π-acceptor) for CCPPh_3_ at the B3LYP/6-311G* level, benchmarking against CO and CNMe.^22^ We revisited and extended this analysis to survey substituent effects at phosphorus on the relative frontier orbital energies (Figure 4). From the viewpoint of an incoming metal center, the relevant orbitals retain closely analogous topology across CO, CNMe, and CCPR_3_, reflecting the shared linear geometry of the terminal carbon atom. Relative orbital energies show that phosphonioacetylides are intrinsically stronger σ and π-donors than CNMe and CO, regardless of substitution, with donor strength increasing as the P substituents become more electron-releasing. The trend follows that observed for phosphines: *F* < OMe < aryl ≈ alkyl ≈ NMe_2_ < NHI.^33,34,48,51–53^ The π-acceptor levels correlate with the σ-donor levels, giving similar energy gaps between donor and acceptor manifolds that span approximately 5.95 eV (CCPF_3_) to 6.98 eV [CCP­(NIMe)_3_] (NIMe = 1,3-dimethylimidazolin-2-ylidenamino). The magnitude of substituent control is highlighted by the extremes: with three electron-withdrawing fluorides, CCPF_3_ is a substantially stronger σ-donor and π-acceptor than CO, whereas CCP­(NIMe)_3_, bearing three NHI substituents, is a powerful σ/π-donor with minimal π-acceptor character, underscoring the tunability of phosphonioacetylides. Orbital ordering also reflects substituent effects. In most cases, the HOMO is the σ-donor orbital and the HOMO–1 is the π-donor. However, with electron-donating P substituents, the LUMO is not the canonical π-acceptor centered on the C_2_ fragment; instead, it is either *P*-centered or associated with aryl π* character (Figures S155–S165). This deviation suggests alternative, P- or aryl-involved low-energy pathways for decomposition of the free phosphonioacetylides.

**Figure 4:**
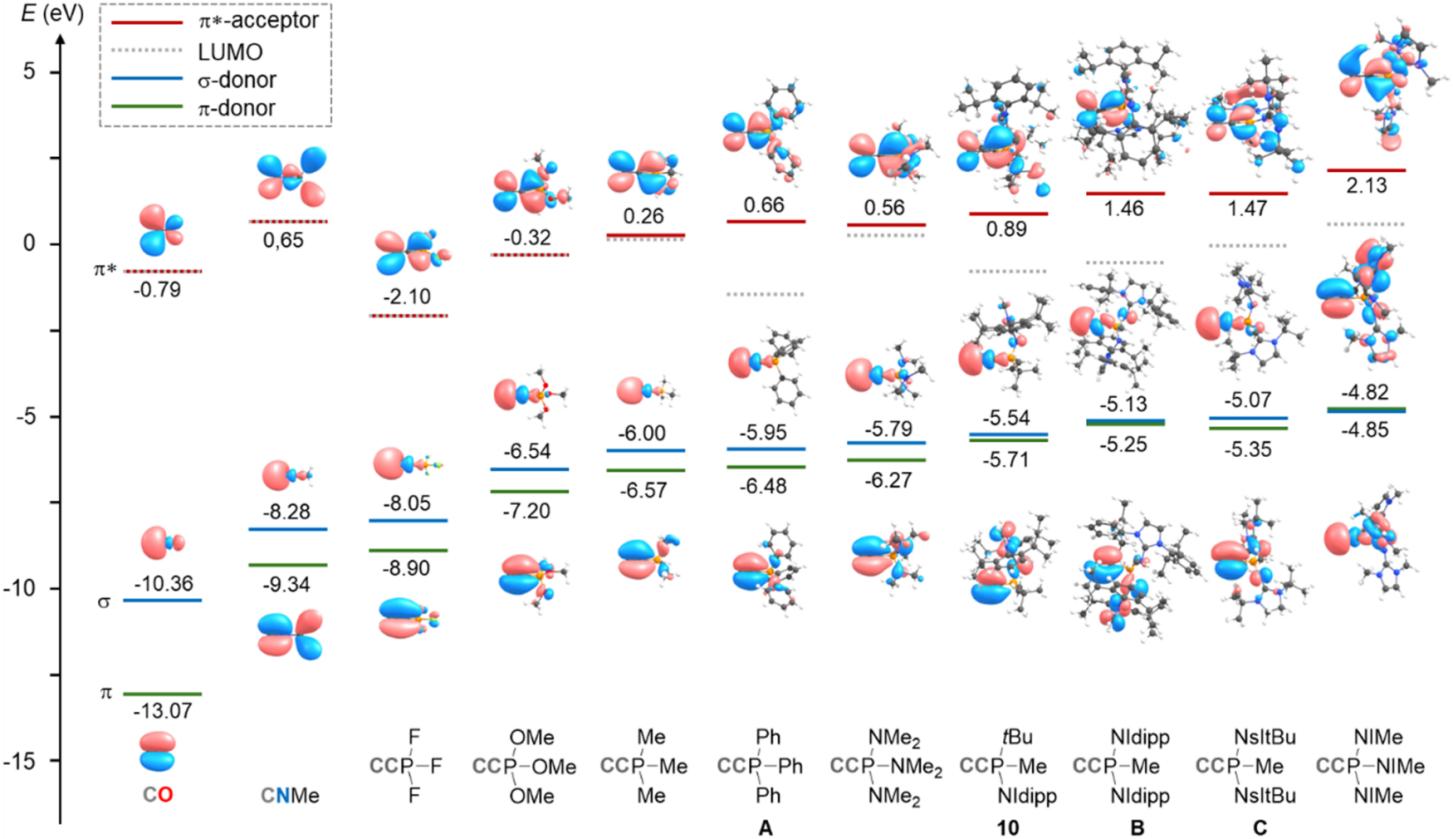
Important frontier orbitals of carbon monoxide, methylisocyanide, and a series of phosphonioacetylides are shown corresponding to the σ-donor, π-donor, and π-acceptor ligand properties. In the case of degenerate π orbitals, only one orbital is depicted. Energies were calculated at the B3LYP/6-311G* level of theory, orbital plots are drawn at the 0.03 isosurface.

To gain further insights into the tunability of phosphonioacetylides, we selected the three representative models CCPF_3_, CCPMe_3_, and CCP­(NIMe)_3_ for further analysis (Table 2). The calculated gas-phase proton affinities (PA) are 222.6, 265.1, and 297.9 kcal mol^–1^ for CCPF_3_, CCPMe_3_, and CCP­(NIMe)_3_, respectively, substantially exceeding those of CO (140.7 kcal mol^–1^) and CNMe (199.9 kcal mol^–1^) and consistent with their elevated donor frontier orbital energies (Figure 4). Notably, the PA of CCP­(NIMe)_3_ surpasses that of the prototypical NHC 1,3-dimethylimidazolin-2-ylidene (261.9 kcal mol^–1^), indicating that electron-donating P substituents can render phosphonioacetylides significantly stronger bases than classical NHCs. The increasing basicity from CCPF_3_ to CCPMe_3_ to CCP­(NIMe)_3_ correlates with natural bond orbital (NBO) charges: the C_2_ fragment becomes more negatively charged, primarily at the terminal carbon, and its polarization decreases with more donating P substituents. Across this series, the Wiberg bond index (WBI) for the P–C bond decreases while that for the C–C bond increases, consistent with enhanced electron density localized on the C_2_ unit. The NBO charges and WBIs of compounds **10**, **B**, and **C** fall within the range observed for CCP­(NIMe)_3_ (see SI, DFT calculations and EDA-NOCV analysis). Energy decomposition analysis (EDA) combined with natural orbitals for chemical valence (NOCV)^54–57^ further clarifies the bonding picture. It suggests that the bonding in the P–C_2_ bond is best described by a fragmentation into C_2_^–^ and R_3_P^+^ in their electronic doublet states, in line with prior analysis of **B** ^30^ and consistent with the NBO charge distribution (see SI for details). Breakdown of the orbital interaction energy Δ*E*_orb_ into the pairwise contributions reveals a dominant electron-sharing σ component, Δ*E*_orb(1)_, complemented by two orthogonal π-donation components from C_2_^–^ into the cationic phosphine fragment, Δ*E*_orb(2)_ and Δ*E*_orb(3)_. In agreement with the WBI trends, electron-withdrawing substituents at P diminish the electron-sharing σ contribution while enhancing C_2_^–^→P π donation. Collectively, these results indicate that resonance forms **I** and **II** best describe the electronic structure of the central P–C_2_ unit for electron-donating substituents, whereas resonance form **III** gains importance with electron-withdrawing P substituents, reflecting increased ambiphilicity at the terminal carbon (Figure 5).

**Figure 5:**
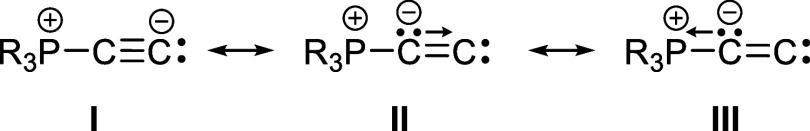
Resonance structures of phosphonioacetylides derived from NBO and EDA-NOCV analyses. Forms **I** and **II** (favored by electron-donating P substituents) emphasize an electron-sharing PC σ bond, CC multiple bond, and negative charge on C_α_ and C_β_, whereas form **III** (enhanced by electron-withdrawing P substituents) highlights increased C_2_^–^→P π donation and ambiphilicity at C_β_.

**Table 2:** Calculated Electronic-Structure Metrics^a^

	CCPF_3_	CCPMe_3_	CCP(NIMe)_3_
PA at *C*_β_ (kcal mol^–1^)	222.6	265.1	297.9
NBO charge of P (in e)	2.43	1.54	2.11
NBO charge of *C*_α_ (in e)	–1.10	–0.98	–0.96
NBO charge of *C*_β_ (in e)	0.20	0.01	–0.17
WBI of *P–C*_α_	1.27	1.13	0.98
WBI of *C*_α_ *–C*_β_	2.28	2.52	2.64
Δ*E*_orb_ (kcal mol^–1^)	–494.79 (57%)	–411.60 (56%)	–393.17 (55%)
Δ*E*_orb(1)_ (P–C_2_ σ electron-sharing bond)	–360.14 (73%)	–330.29 (80%)	–324.65 (83%)
Δ*E*_orb(2)_ (C_2_^–^→P π donation)	–55.73 (11%)	–25.37 (6%)	–13.64 (3%)
Δ*E*_orb(3)_ (C_2_^–^→P π donation)	–57.90 (12%)	–30.26 (7%)	–23.00 (6%)
Δ*E*_orb(rest)_	–21.02 (4%)	–25.68 (6%)	–31.88 (8%)

a Calculated gas-phase proton affinities (PA) using B3LYP-D3/6-311+G­(2df,p), natural atomic charges and Wiberg bond indices (WBI) from the NBO analysis, and orbital interaction energies Δ*E*_orb_ from the EDA-NOCV calculations using B3LYP/6-311G­(d).

## Conclusion

We have prepared and characterized two room-temperature-persistent phosphonioacetylides (**6** and **10**) that illustrate how NHI-driven steric and electronic tuning governs their stability, donor strength, and reactivity. Installing two bulky, yet conformationally flexible *tert*-octyl NHIs (**6**) or a single dipp-NHI (**10**) combined with alkyl groups affords isolable ligands with accessible, reactive C_2_ units. Percent buried volumes indicate that both ligands are less sterically demanding than **B** yet bulkier than **C**, consistent with their persistence. Their strong donor properties are evidenced by clean formation of cationic, linear Au­(I) complexes from [AuCl­(tht)], and by HEP measurements that place their σ donor ability in the NHC regime. The two electronically distinct donors display divergent reactivity: **6** decomposes unselectively upon heating or hydrolysis, whereas **10** undergoes formal C_2_ elimination to give phosphine **11** and, with water, the phosphine oxide **12** and acetylene. The mechanism of this C_2_ cleavage and any potential transfer to an external acceptor remain to be established, which is an objective of future work. DFT calculations establish that phosphorus substituents systematically tune frontier orbital energies, with σ-/π-donor strength increasing from electron withdrawing to electron-donating groups (*F* < OMe < aryl ≈ alkyl ≈ NMe_2_ < NHI) and concomitant modulation of π-acceptor ability. In the most electron-rich cases, the LUMO shifts to the P center or acquires aryl π* character, suggesting decomposition pathways of the free ligands. Complementary theoretical studies show that the calculated gas-phase proton affinities rise from CCPF_3_ (222.6 kcal mol^–1^) to CCPMe_3_ (265.1 kcal mol^–1^) to CCP­(NIMe)_3_ (297.9 kcal mol^–1^), surpassing CO, CNMe, and even classical NHCs (1,3-dimethylimidazolin-2-ylidene, 261.9 kcal mol^–1^). NBO analysis shows increasing negative charge localized at the terminal carbon and decreasing polarization of the C_2_ unit with more donating P substituents, while WBI indicate a weakening of the P–C bond and strengthening of the C–C bond across the series. EDA–NOCV indicated that the P–C bond is best described by a major electron-sharing P–C σ component supplemented by two C_2_^–^→P π donations, with electron-withdrawing P substituents diminishing the electron-sharing σ contribution while enhancing the π interaction. Together, these results provide clear design principles for stabilizing and tailoring phosphonioacetylides as strong, tunable neutral carbon donors. Ongoing studies aim to expand this ligand family and further explore their reactivity and coordination chemistry.

## Experimental Section

### General Remarks

Unless otherwise noted, all manipulations were performed under an inert atmosphere of dry argon, using standard Schlenk and drybox techniques. Dry and oxygen-free solvents were employed. ItOct·HBF4,^40^ TMSNIdipp,^41^ and [Au­(tht)­Cl] ^58,59^ were prepared following a literature procedure. All other compounds were purchased from commercial sources and used as received. Caution! Phosphorus trichloride is highly corrosive and moisture-reactive and must be handled with care. Methyl iodide is very toxic and a potent alkylating agent and must be handled with extreme care. Trimethylsilyl azide must be kept away from acids and metals to prevent the formation of explosive azides.

### Syntheses of the New Compounds and Their Most Relevant NMR Characterization Data

**1**: THF (50 mL) was slowly added to a stirred mixture of I*t*Oct·HBF_4_ (10.20 g, 30.9 mmol, 1.00 equiv) and KO*t*Bu (4.16 g, 37.1 mmol, 1.20 equiv) in a Schlenk tube at – 78 °C. The reaction mixture was slowly warmed to room temperature and stirred overnight. All volatiles were removed *in vacuo*. Compound **1** was extracted from the residual solid with *n*-hexane (40 mL). After filtration, the solvent was removed *in vacuo* to yield the carbene **1** as a white solid (7.85 g, 26.9 mmol, 87%). ^**1**^ **H NMR** (400 MHz, C_6_D_6_) δ (ppm) = 6.74 (s, 2H, CH), 1.87 (s, 4H, CH_2_), 1.61 (s, 12H, (CH_3_)_2_), 0.87 (s, 18H, C­(CH_3_)_3_). ^**13**^ **C­{** ^**1**^ **H} NMR** (101 MHz, C_6_D_6_) δ (ppm) = 214.7 (s, NCN), 115.6 (s, CH), 59.2 (s, qC­(CH_3_)_2_), 55.5 (s, CH_2_), 31.9 (s, qC­(CH_3_)_3_), 31.8 (s, (CH_3_)_2_), 31.3 (s, (CH_3_)_3_).

**2**: Trimethylsilyl azide (2.00 mL, 15.23 mmol, 1.50 equiv) was slowly added to a solution of **1** (2.97 g, 10.15 mmol, 1.00 equiv) in toluene (60 mL) in a Schlenk flask equipped with an aircondenser and an overpressure bubbler. The reaction mixture was heated to reflux at 120 °C for 15 d. The solvent was removed *in vacuo* which gave a viscous oil. To facilitate the evaporation of toluene, the residue was redissolved in pentane (20 mL) and all volatiles were removed *in vacuo* to afford imine **2** as an off-white solid (3.31 g, 8.72 mmol, 86%). ^**1**^ **H NMR** (400 MHz, C_6_D_6_) δ (ppm) = 6.02 (s, 2H, CH), 2.07 (s, 4H, CH_2_), 1.38 (s, 12H, (CH_3_)_2_), 0.93 (s, 18H, C­(CH_3_)_3_), 0.54 (s, 9H, Si­(CH_3_)_3_). ^**13**^ **C­{** ^**1**^ **H} NMR** (101 MHz, C_6_D_6_) δ (ppm) = 139.6 (s, NCN), 107.7 (s, CH), 57.9 (s, qC­(CH_3_)_2_), 47.7 (s, CH_2_), 31.9 (s, qC­(CH_3_)_3_), 31.4 (s, (CH_3_)_3_), 29.7 (s, (CH_3_)_2_), 5.0 (s, Si­(CH_3_)_3_). ^**29**^ **Si DEPT-19.5 NMR** (80 MHz, C_6_D_6_) δ (ppm) = −29.1 (s).

**3**: PCl_3_ in toluene (2.4 mL, 1.05 M, 2.52 mmol, 1.00 equiv) was added dropwise to a solution of **2** (2.00 g, 5.27 mmol, 2.10 equiv) in THF (60 mL) in a Schlenk tube, resulting in the precipitation of **3** as a yellow solid. The suspension was stirred overnight at room temperature to complete the reaction, and then all volatiles were removed *in vacuo*. The solid residue was washed with diethyl ether (2 × 20 mL) to remove the excess of **2**. After drying the remaining solid *in vacuo*, product **3** was obtained as a bright yellow solid (1.52 g, 2.24 mmol, 90%). ^**1**^ **H NMR** (400 MHz, CD_3_CN) δ (ppm) = 7.08 (s, 2H, CH), 2.15 (s, 4H, CH_2_), 1.70 (s, 12H, (CH_3_)_2_), 0.85 (s, 18H, C­(CH_3_)_3_). ^**1**^ **H­{** ^**31**^ **P} NMR** (400 MHz, CD_3_CN) δ (ppm) = 7.08 (s, 2H, CH), 2.15 (s, 4H, CH_2_), 1.70 (s, 12H, (CH_3_)_2_), 0.85 (s, 18H, C­(CH_3_)_3_). ^**13**^ **C­{** ^**1**^ **H} NMR** (101 MHz, CD_3_CN) δ (ppm) = 145.2 (s, NCN), 114.9 (s, CH), 62.9 (s, qC­(CH_3_)_2_), 51.4 (d, ^5^ *J*_CP_ = 4 Hz, CH_2_), 32.6 (s, qC­(CH_3_)_3_), 31.2 (d, ^7^ *J*_CP_ = 1 Hz, (CH_3_)_3_), 30.8 (d, ^5^ *J*_CP_ = 3 Hz, (CH_3_)_2_). ^**31**^ **P NMR** (162 MHz, CD_3_CN): δ (ppm) = 278.7 (s).

**4**: Ethynyl magnesium chloride in THF (5.9 mL, 0.5 M, 2.944 mmol, 2.00 equiv) was added dropwise to a suspension of **3** (1.000 g, 1.472 mmol, 1.00 equiv) in THF (10 mL) at −78 °C. While stirring, the reaction mixture was gently warmed to room temperature overnight in the cooling bath. All volatiles were removed *in vacuo*. The residue was extracted with *n*-hexane (3 × 40 mL), and the solvent was removed *in vacuo*. Phosphine **4** was obtained as white solid (652 mg, 0.976 mmol, 66%). ^**1**^ **H NMR** (400 MHz, C_6_D_6_) δ (ppm) = 6.17 (s, 4H, CH (NCHCHN)), 2.80 (d, ^3^ *J*_HP_ = 3.1 Hz, 1H, CH­(CCH)), 2.54 (dd, ^4^ *J*_HH_ = 14.9 Hz, ^3^ *J*_HP_ = 2.5 Hz, 4H, CH_2_), 2.38 (dd, ^4^ *J*_HH_ = 14.9 Hz, ^3^ *J*_HP_ = 2.8 Hz, 4H, CH_2_), 1.67 (d, ^5^ *J*_HH_ = 17.4 Hz, 24H, C­(CH_3_)_2_), 1.09 (s, 36H, C­(CH_3_)_3_). ^**13**^ **C­{** ^**1**^ **H} NMR** (101 MHz, C_6_D_6_) δ (ppm) = 144.3 (d, ^2^ *J*_CP_ = 29 Hz, NCN), 109.2 (s, CH), 96.4 (d, ^1^ *J*_CP_ = 74 Hz, αC (P-CC)), 86.1 (d, ^2^ *J*_CP_ = 13 Hz, βC (H–CC)), 59.2 (s, qC­(CH_3_)_2_), 50.3 (d, ^5^ *J*_CP_ = 10 Hz, CH_2_), 32.2 (s, qC­(CH_3_)_3_), 31.7 (d, ^7^ *J*_CP_ = 2 Hz, (CH_3_)_3_), 31.1 (dd, ^5^ *J*_CP_ = 5 Hz, ^5^ *J*_CP_ = 4 Hz, (CH_3_)_2_). ^**31**^ **P NMR** (162 MHz, C_6_D_6_) δ (ppm) = 63.0 (s).

**5**: Methyl iodide in toluene (1.06 mL, 0.765 M, 0.807 mmol, 1.20 equiv) was slowly added to a solution of **4** (450 mg, 0.673 mmol, 1.00 equiv) in *n*-hexane (30 mL). The reaction mixture was stirred for overnight at room temperature. All volatiles were removed *in vacuo*. **5** was obtained as white solid (522 mg, 0.644 mmol, 96%). ^**1**^ **H NMR** (400 MHz, CD_3_CN) δ (ppm) = 6.97 (s, 4H, CH (NCHCHN)), 3.57 (d, ^3^ *J*_HP_ = 10.3 Hz, 1H, CH (CCH)), 2.14–2.02 (m, 8H, CH_2_), 1.90 (d, ^2^ *J*_HP_ = 13.4, 3H, PCH_3_), 1.76 (d, ^5^ *J*_HH_ = 12.7 Hz, 24H, C­(CH_3_)_2_), 0.89 (s, 36H, C­(CH_3_)_3_). ^**13**^ **C­{** ^**1**^ **H} NMR** (101 MHz, CD_3_CN) δ (ppm) = 144.2 (d, ^2^ *J*_CP_ = 16 Hz, NCN), 114.1 (s, CH), 93.2 (d, ^2^ *J*_CP_ = 29 Hz, βC (H–CC)), 83.5 (d, ^1^ *J*_CP_ = 171 Hz, αC (P-CC)), 63.2 (s, qC­(CH_3_)_2_), 51.8 (s, CH_2_), 32.4 (s, qC­(CH_3_)_3_), 31.3 (s, (CH_3_)_3_), 30.9 (d, ^5^ *J*_CP_ = 7 Hz, (CH_3_)_2_), 22.5 (d, ^1^ *J*_CP_ = 114 Hz, PCH_3_). ^**31**^ **P NMR** (162 MHz, CD_3_CN) δ (ppm) = −34.5 (qd, ^2^ *J*_PH_ = 13 Hz, ^3^ *J*_PH_ = 10 Hz). ^**31**^ **P­{** ^**1**^ **H} NMR** (162 MHz, CD_3_CN) δ (ppm) = – 34.5 (s).

**6**: A solution of KHMDS (26 mg, 0.130 mmol, 1.06 equiv) in THF (1 mL) cooled to −40 °C was quickly added to a stirred suspension of **5** (100 mg, 0.123 mmol, 1.00 equiv) in THF (2 mL) at −40 °C. It is noted that the base needs to be added quickly and the reaction temperature must be −40 °C or lower to avoid side reactions. Immediately after the addition, all volatiles were removed *in vacuo* while keeping the mixture at −40 °C. The residue was washed with cold diethyl ether (2 × 1 mL, −40 °C) to remove excess KHMDS but avoid dissolving of the product. The residue was extracted at ambient temperature with toluene (3 × 2 mL) to remove formed potassium iodide. After filtration, the solvent was removed *in vacuo*. Phosphonioacetylide **6** was obtained as a brown solid (65 mg, 0.095 mmol, 77%). ^**1**^ **H NMR** (400 MHz, C_6_D_6_) δ (ppm) = 6.19 (s, 4H, CH (NCHCHN)), 2.29 (s, 8H, CH_2_), 1.91 (s, 3H, PCH_3_), 1.79 (s, 12H, C­(CH_3_)_2_), 1.63 (s, 12H, C­(CH_3_)_2_), 1.06 (s, 36H, C­(CH_3_)_3_). ^**13**^ **C­{** ^**1**^ **H} NMR** (176 MHz, C_6_D_6_) δ (ppm) = 216.4 (d, ^2^ *J*_CP_ = 16 Hz, βC (CC)), 145.3 (d, ^2^ *J*_CP_ = 15 Hz, NCN), 111.2 (s, CH), 105.5 (d, ^1^ *J*_CP_ = 143 Hz, αC (P-CC)), 61.4 (s, qC­(CH_3_)_2_), 51.0 (s, CH_2_), 32.1 (s, qC­(CH_3_)_3_), 31.5 (s, (CH_3_)_3_), 31.0 (d, ^5^ *J*_CP_ = 45 Hz, (CH_3_)_2_), 23.9 (d, ^1^ *J*_CP_ = 108 Hz, PCH_3_). ^**31**^ **P NMR** (162 MHz, C_6_D_6_): δ (ppm) = −37.4 (q, ^2^ *J*_CH_ = 13 Hz). ^**31**^ **P­{** ^**1**^ **H} NMR** (162 MHz, C_6_D_6_): δ (ppm) = −37.4 (s).

**7**: A solution of the silylated N-heterocyclic imine TMSNIdipp (2.000 g, 4.203 mmol, 1.00 equiv) in toluene (10 mL) was slowly added via cannula transfer to a stirred solution of *t*BuPCl_2_ in toluene (15.8 mL, 0.271 M, 4.287 mmol, 1.02 equiv) at −78 °C. The reaction mixture was stirred for 30 min while keeping it at −78 °C, then allowed to warm to room temperature and stirred for an additional 16 h. All volatiles including excess *t*BuPCl_2_ were removed *in vacuo* (60 °C at 10^–3^ mbar) affording the title compound as a beige/white powder (2.078 g; 3.949 mmol, 94%). ^**1**^ **H NMR** (400 MHz, C_6_D_6_) δ (ppm) = 7.26–7.22 (m, Ar–H (para)), 7.15–7.11­(m, 4H, Ar–H (meta)), 5.98 (s, 2H, NCHCHN), 3.16 (sept, ^3^ *J*_HH_ = 6.9 Hz, 2H, CH (*i*Pr)), 2.98 (sept, ^3^ *J*_HH_ = 6.9 Hz, 2H, CH (*i*Pr)), 1.49 (d, ^3^ *J*_HH_ = 6.9 Hz, 6H, CH_3_ (*i*Pr)), 1.43 (d, ^3^ *J*_HH_ = 6.8 Hz, 6H, CH_3_ (*i*Pr)), 1.15 (d, ^3^ *J*_HH_ = 2.3 Hz, 6H, CH_3_ (*i*Pr)), 1.13 (d, ^3^ *J*_HH_ = 2.3 Hz, 6H, CH_3_ (*i*Pr)), 0.96 (d, ^3^ *J*_HP_ = 12 Hz, 9H, CH_3_ (*t*Bu)). ^**13**^ **C­{** ^**1**^ **H} NMR** (C_6_D_6_, 101 MHz) δ (ppm) = 150.4 (d, ^2^ *J*_CP_ = 13 Hz, NCN), 147.7 (s, qC (Ar-*i*Pr)), 147.6 (s, qC (Ar- *i*Pr)), 147.1, (s, ipso-C (Ar–H)), 133.6, (s, ortho-C (Ar)), 130.3 (s, para-C (Ar–H)), 124.3 (s, meta-C (Ar–H)), 124.0 (s, meta-C (Ar–H)), 116.3 (s, NCHCHN), 36.7 (d, ^1^ *J*_CP_ = 24 Hz, qC (*t*Bu)), 29.3 (s, CH (*i*Pr)), 29.3 (s, CH (*i*Pr)), 29.2 (s, CH (*i*Pr)), 25.1 (s, CH_3_ (*i*Pr)), 24.9 (s, CH_3_ (*i*Pr)), 24.8 (d, ^2^ *J*_CP_ = 18 Hz, CH_3_ (*t*Bu)), 23.3 (s, CH_3_ (*i*Pr)), 22.9 (s, CH_3_ (*i*Pr)), 22.8 (s, CH_3_ (*i*Pr)). ^**31**^ **P NMR** (C_6_D_6_, 162 MHz) δ (ppm) = 157.6 (decet, ^3^ *J*_PH_ = 13 Hz). ^**31**^ **P­{** ^**1**^ **H} NMR** (162 MHz, C_6_D_6_) δ (ppm) = 157.6 (s).

**8**: Ethynyl magnesium chloride in THF (8.1 mL, 0.5 M, 4.052 mmol, 2.00 equiv) was added dropwise to **7** (1.066 g, 2.026 mmol, 1.00 equiv) in THF (20 mL) at −78 °C. The reaction mixture was allowed to warm to room temperature over 16 h while stirring. All volatile compounds were removed *in vacuo*. The residue was extracted with hexane (3 × 20 mL). All volatiles were removed *in vacuo* and the product was received as a white solid (1.673 g, 0.863 mmol, 83%). ^**1**^ **H NMR** (400 MHz, C_6_D_6_) δ (ppm) = 7.26–7.10 (m, 6H, Ar–H), 5.95 (s, 2H, NCHCHN), 3.31 (hept, ^3^ *J*_HH_ = 6.9 Hz, 2H, CH (*i*Pr)), 3.07 (hept, ^3^ *J*_HH_ = 6.9 Hz, 2H, CH (*i*Pr)), 2.24 (d, ^3^ *J*_HP_ = 1.8 Hz, 1H, CCH), 1.50 (d, ^3^ *J*_HH_ = 6.8 Hz, 6H, CH_3_ (*i*Pr)), 1.41 (d, ^3^ *J*_HH_ = 6.9 Hz, 6H, CH_3_ (*i*Pr)), 1.19 (d, ^3^ *J*_HH_ = 6.9 Hz, 12H, CH_3_ (*i*Pr)), 1.02 (d, ^3^ *J*_PH_ = 13.4 Hz, 9H, CH_3_ (*t*Bu)). ^**13**^ **C­{** ^**1**^ **H} NMR** (101 MHz, C_6_D_6_) δ (ppm) = 148.8 (d, ^2^ *J*_CP_ = 18 Hz, NCN), 148.5 (d, *J*_CP_ = 2 Hz, q-C (Ar-*i*Pr)), 147.4 (s, C (Ar–H)), 134.5 (s, ipso-C (Ar)), 129.9 (s, C (Ar–H)), 124.0 (s, C (Ar–H)), 123.9 (s, C (Ar–H)), 115.5 (s, NCHCHN), 89.3 (d, ^2^ *J*_CP_ = 9 Hz, βC (H–CC)), 89.2 (d, ^1^ *J*_CP_ = 54 Hz, αC (P-CC)), 32.5 (d, ^1^ *J*_CP_ = 3 Hz, q-C (*t*Bu)), 29.3 (d, *J*_CP_ = 3 Hz, CH (*i*Pr)), 29.0 (s, CH (*i*Pr)), 25.8 (d, ^2^ *J*_CP_ = 16 Hz, CH_3_ (*t*Bu)), 25.1 (s, CH_3_ (*i*Pr)), 24.8 (s, CH_3_ (*i*Pr)), 23.6 (s, CH_3_ (*i*Pr)), 22.8 (d, *J*_CP_ = 4 Hz, CH_3_ (*i*Pr)). ^**31**^ **P NMR** (162 MHz, C_6_D_6_) δ (ppm) = 34.4 (dh, ^3^ *J*_HP_ = 27 Hz, ^3^ *J*_HP_ = 14 Hz). ^**31**^ **P­{** ^**1**^ **H} NMR** (162 MHz, C_6_D_6_) δ (ppm) = 34.4 (s).

**9**: Methyl iodide in toluene (0.35 mL, 0.803 M, 0.281 mmol, 1.45 equiv) was added dropwise to a solution of **8** (100 mg, 0.194 mmol, 1.00 equiv) in toluene (20 mL). The reaction mixture was stirred overnight at ambient temperature. The resulting suspension was filtered, and the residue was washed with *n*-hexane (20 mL). The residue was dried *in vacuo* to afford the product **9** as a white solid (110 mg, 0.167 mmol, 86%). ^**1**^ **H NMR** (400 MHz, CD_3_CN) δ (ppm) = 7.59–7.40 (m, 6H, Ar–H), 7.20 (s, 2H, NCHCHN), 3.69 (d, ^3^ *J*_HP_ = 9.7 Hz, 1H, CCH), 2.79–2.61 (m, 4H, CH (*i*Pr)), 1.32 (dd, *J*_HH_ = 6.8 Hz, *J*_HH_ = 12.2 Hz, 12H, CH_3_ (*i*Pr)), 1.20 (t, *J*_HH_ = 6.9 Hz, 12H, CH_3_ (*i*Pr)), 0.90 (d, ^2^ *J*_HP_ = 13.1 Hz, 3H, CH_3_ (Me)), 0.76 (d, ^3^ *J*_PH_ = 19.4 Hz, 9H, CH_3_ (*t*Bu)). ^**13**^ **C­{** ^**1**^ **H} NMR** (101 MHz, CD_3_CN) δ (ppm) = 147.7 (d, ^2^ *J*_CP_ = 13 Hz, NCN), 146.4 (s, q-C (Ar-*i*Pr)), 132.3 (s, C (Ar–H)), 132.1 (s, ipso-C (Ar)), 125.7 (s, C (Ar–H)), 125.6 (s, C (Ar–H)), 120.0 (s, NCHCHN), 98.9 (d, ^2^ *J*_CP_ = 21 Hz, βC (H–CC)), 72.8 (d, ^1^ *J*_CP_ = 136 Hz, αC (P-CC)), 33.5 (d, ^1^ *J*_CP_ = 95 Hz, q-C (*t*Bu)), 29.7 (d, *J*_CP_ = 3 Hz, CH (*i*Pr)), 25.4 (s, CH_3_ (*i*Pr)), 25.4 (s, CH_3_ (*i*Pr)), 23.1 (s, CH_3_ (*i*Pr)), 22.9 (s, CH_3_ (*i*Pr)), 22.6 (d, ^3^ *J*_CP_ = 1 Hz, CH_3_ (*t*Bu)), 12.7 (d, ^1^ *J*_CP_ = 71 Hz, CH_3_ (Me)). ^**31**^ **P NMR** (162 MHz, CD_3_CN) δ (ppm) = 11.6–10.8 (m). ^**31**^ **P­{** ^**1**^ **H} NMR** (162 MHz, CD_3_CN) δ (ppm) = 11.2 (s).

**10**: A solution of KHMDS (62 mg, 0.311 mmol, 1.02 equiv) in THF (2 mL) was added to a stirred suspension of **9** (200 mg, 0.304 mmol, 1.00 equiv) in THF (5 mL) at −40 °C. Immediately after the addition, the volatiles were removed *in vacuo* while warming up to room temperature. The residue was washed with cold Et_2_O (2 mL, −40 °C) to remove excess KHMDS and avoid dissolving of the product.[Fn fn1] The residue was extracted with toluene (3 × 5 mL) to remove formed potassium iodide, and the solvent was removed *in vacuo* to afford phosphonioacetylide **10** as an off-white powder (125 mg, 0.236 mmol, 78%). ^**1**^ **H NMR** (600 MHz, C_6_D_6_) δ (ppm) = 7.20–7.07 (m, 6H, Ar–H), 6.19 (s, 2H, NCHCHN), 3.38 (hept, ^3^ *J*_HH_ = 6.8 Hz, 2H, CH (*i*Pr)), 3.09 (hept, ^3^ *J*_HH_ = 6.8 Hz, 2H, CH (*i*Pr)), 1.60 (d, ^3^ *J*_HH_ = 6.7 Hz, 6H, CH_3_ (*i*Pr)), 1.48 (d, ^3^ *J*_HH_ = 6.7 Hz, 6H, CH_3_ (*i*Pr)), 1.09 (d, ^3^ *J*_HH_ = 6.9 Hz, 6H, CH_3_ (*i*Pr)), 1.07 (d, ^3^ *J*_HH_ = 7.0 Hz, 6H, CH_3_ (*i*Pr)), 0.75 (d, ^2^ *J*_HP_ = 17.1 Hz, 9H, CH_3_ (*t*Bu)), 0.31 (d, ^3^ *J*_PH_ = 12.6 Hz, 3H, CH_3_ (Me)). ^**13**^ **C­{** ^**1**^ **H} NMR** (151 MHz, C_6_D_6_) δ (ppm) = 231.8 (d, ^2^ *J*_CP_ = 17 Hz, βC (CC)), 149.3 (s, q-C (Ar-*i*Pr)), 147.9 (d, ^2^ *J*_CP_ = 42 Hz, NCN), 132.9 (s, ipso-C (Ar–H)), 130.17 (s, C (Ar)), 128.3 (s, C (Ar–H)), 124.7 (s, C (Ar–H)), 124.5 (s, C (Ar–H)), 117.3 (s, NCHCHN), 90.8 (d, ^1^ *J*_CP_ = 112 Hz, αC (P-CC)), 32.4 (d, ^1^ *J*_CP_ = 94 Hz, q-C (*t*Bu)), 29.1 (s, CH (*i*Pr)), 28.9 (s, CH (*i*Pr)), 25.8 (s, CH_3_ (*i*Pr)), 25.6 (s, CH_3_ (*i*Pr)), 23.9 (s, CH_3_ (*i*Pr)), 23.6 (d, ^2^ *J*_CP_ = 3 Hz, CH_3_ (*t*Bu)), 23.4 (s, CH_3_ (*i*Pr)), 13.5 (d, ^1^ *J*_CP_ = 72 Hz, CH_3_ (Me)). ^**31**^ **P NMR** (162 MHz, C_6_D_6_) δ (ppm) = −2.4 to −2.8 (m). ^**31**^ **P­{** ^**1**^ **H} NMR** (162 MHz, C_6_D_6_) δ (ppm) = −2.6 (s).

**11**: Methylmagnesium chloride in THF (0.07 mL, 3.0 M, 0.210 mmol, 1.10 equiv) was added dropwise to **7** (100 mg, 0.190 mmol, 1.00 equiv) in THF (5 mL) at −78 °C. The reaction mixture was allowed to warm to room temperature while stirring over 16 h. All volatile compounds were removed *in vacuo*. The residue was extracted with *n*-hexane (2 × 25 mL). All volatiles were removed *in vacuo*, and the product **11** was received as a white solid (50 mg, 0.099 mmol, 52%). ^**1**^ **H NMR** (400 MHz, C_6_D_6_) δ (ppm) = 7.26–7.22 (m, 2H, Ar–H (para)), 7.17–7.11 (m, 4H, Ar–H (meta)), 5.96 (s, 2H, NCHCHN), 3.42 (sept, ^3^ *J*_HH_ = 6.9 Hz, 2H, CH (*i*Pr)), 3.07 (sept, ^3^ *J*_HH_ = 7.0 Hz, 2H, CH (*i*Pr)), 1.52 (d, ^3^ *J*_HH_ = 6.9 Hz, 6H, CH_3_ (*i*Pr)), 1.36 (d, ^3^ *J*_HH_ = 6.9 Hz, 6H, CH_3_ (*i*Pr)), 1.19 (dd, ^3^ *J*_HH_ = 7.9 Hz, ^3^ *J*_HH_ = 6.9 Hz, 12H, CH_3_ (*i*Pr)), 0.84 (d, ^3^ *J*_HP_ = 11.7 Hz, 9H, CH_3_ (*t*Bu)), 0.51 (d, ^3^ *J*_HP_ = 5.2 Hz, 3H, CH_3_ (Me)). ^**13**^ **C­{** ^**1**^ **H} NMR** (C_6_D_6_, 101 MHz) δ (ppm) = 148.9 (s, q-C (Ar- *i*Pr)), 148.9 (s, ipso-C (Ar–H)), 147.3 (s, q-C (Ar-*i*Pr)), 147.0 (d, ^2^ *J*_CP_ = 16 Hz, NCN), 135.1, (s, ortho-C (Ar)), 129.6 (s, para-C (Ar–H)), 123.8 (s, meta-C (Ar–H)), 115.1 (s, NCHCHN), 30.4 (d, ^1^ *J*_CP_ = 6 Hz, q-C (*t*Bu)), 29.4 (s, CH (*i*Pr)), 29.4 (s, CH (*i*Pr)), 28.9 (s, CH (*i*Pr)), 25.7 (s, ^2^ *J*_CP_ = 16 Hz, CH_3_ (*t*Bu)), 25.2 (s, CH_3_ (*i*Pr)), 25.0 (s, CH_3_ (*i*Pr)), 23.3 (s, CH_3_ (*i*Pr)), 22.6 (s, CH_3_ (*i*Pr)), 22.6 (s, CH_3_ (*i*Pr)), 14.2 (d, ^1^ *J*_CP_ = 13 Hz, CH_3_ (Me)). ^**31**^ **P NMR** (C_6_D_6_, 162 MHz) δ (ppm) = 42.1 (m). ^**31**^ **P­{** ^**1**^ **H} NMR** (162 MHz, C_6_D_6_) δ (ppm) = 42.1 (s).

**12**: In an NMR tube with a gas cap, a solution of **11** (12 mg, 0.0237 mmol, 1.00 equiv) in benzene (0.6 mL) was frozen, and subsequently, the argon atmosphere was removed *in vacuo*. After warming the reaction mixture to room temperature, N_2_O (1.5 bar) was pressured on the reaction vessel. The reaction was monitored by ^31^P NMR, revealing the formation of a transient species at 42.1 ppm presumably the phosphine-N_2_O-adduct, which reacts over time to the reaction product **12** at 31.1 ppm. The reaction mixture was stirred overnight under N_2_O atmosphere to complete the reaction. After removal of all volatiles *in vacuo*, the phosphine oxide **12** was received as a white powder (14 mg, 0.0230 mmol, 97%). ^**1**^ **H NMR** (400 MHz, C_6_D_6_) δ (ppm) = 7.25–7.17 (m, 4H, Ar–H), 7.11–7.09 (m, 4H, Ar–H), 6.06 (s, 2H, NCHCHN), 3.33 (sept, ^3^ *J*_HH_ = 6.9 Hz, 2H, CH (*i*Pr)), 2.94 (sept, ^3^ *J*_HH_ = 6.9 Hz, 2H, CH (*i*Pr)), 1.57 (d, ^3^ *J*_HH_ = 6.8 Hz, 6H, CH_3_ (*i*Pr)), 1.38 (d, ^3^ *J*_HH_ = 6.9 Hz, 6H, CH_3_ (*i*Pr)), 1.14 (t, ^3^ *J*_HH_ = 7.2 Hz, 12H, CH_3_ (*i*Pr)), 0.88 (d, ^3^ *J*_HP_ = 14.5 Hz, 9H, CH_3_ (*t*Bu)), 0.48 (d, ^3^ *J*_HP_ = 12.6 Hz, 3H, CH_3_ (Me)). ^**13**^ **C­{** ^**1**^ **H} NMR** (C_6_D_6_, 101 MHz) δ (ppm) = 148.4 (s, C (Ar–H)), 147.5 (d, ^2^ *J*_CP_ = 10 Hz, NCN), 147.4 (s, q-C (Ar-*i*Pr)), 134.1 (s, ipso-C (Ar)), 130.1 (s, C (Ar–H)), 124.2 (s, C (Ar–H)), 124.1 (s, C (Ar–H)), 115.9 (s, NCHCHN), 33.1 (d, ^1^ *J*_CP_ = 106 Hz, q-C (*t*Bu)), 29.4 (s, CH (*i*Pr)), 29.0 (s, CH (*i*Pr)), 25.4 (s, CH_3_ (*i*Pr)), 25.1 (s, CH_3_ (*t*Bu)), 25.0 (s, CH_3_ (*i*Pr)), 23.2 (s, CH_3_ (*i*Pr)), 23.0 (s, CH_3_ (*i*Pr)), 12.8 (d, ^1^ *J*_CP_ = 79 Hz, CH_3_ (Me)). ^**31**^ **P NMR** (C_6_D_6_, 162 MHz) δ (ppm) = 31.4–30.9 (m). ^**31**^ **P­{** ^**1**^ **H} NMR** (162 MHz, C_6_D_6_) δ (ppm) = 31.1 (s).

**13**: Phosphonioacetylide **6** (31 mg, 0.0312 mmol, 3.00 equiv) and Au­(tht)Cl (5 mg, 0.0156 mmol, 1.00 equiv) were dissolved in toluene (3 mL) and stirred overnight. Using ^31^P NMR, full conversion was observed with residual **6**. All volatile compounds were removed *in vacuo*. The residue was washed with diethyl ether to remove the residual **6** (2 × 1 mL). The residue was dried *in vacuo*. **13** was obtained as a white solid (5 mg, 0.0031 mmol, 20%). ^**1**^ **H NMR** (400 MHz, CD_3_CN) δ (ppm) = 6.87 (s, 8H, CH (NCHCHN)), 2.19–2.05 (m, 16H, CH_2_), 1.77–1.72 (m, 54H, C­(CH_3_)_2_ + PCH_3_), 0.90 (s, 36H, C­(CH_3_)_3_). ^**13**^ **C­{** ^**1**^ **H} NMR** (101 MHz, CD_3_CN) δ (ppm) = 156.9 (dd, ^2^ *J*_CP_ = 20 Hz, ^4^ *J*_CP_ = 3 Hz, βC (Au-CC)), 145.1 (d, ^2^ *J*_CP_ = 16 Hz, NCN), 113.3 (s, CH), 98.4 (d, ^1^ *J*_CC_ = 180 Hz, αC (P-CC)), 62.7 (s, qC­(CH_3_)_2_), 51.7 (s, CH_2_), 32.5 (s, qC­(CH_3_)_3_), 31.6 (s, (CH_3_)_3_), 31.1 (d, ^5^ *J*_CP_ = 18 Hz, (CH_3_)_2_), 23.1 (d, ^1^ *J*_CP_ = 112 Hz, PCH_3_). ^**31**^ **P NMR** (162 MHz, CD_3_CN) δ (ppm) = −33.5 (q, ^3^ *J*_PH_ = 13 Hz). ^**31**^ **P­{** ^**1**^ **H} NMR** (162 MHz, CD_3_CN) δ (ppm) = −33.5 (s).

**14**: Au­(tht)Cl (6 mg, 0.0189 mmol, 1.00 equiv) was added to a solution of **10** (20 mg, 0.0378 mmol, 2.00 equiv) in toluene (3 mL). The reaction mixture was stirred for 1 h, where a suspension was formed. The absence of phosphorus species in the supernatant was confirmed by ^31^P NMR, indicating a complete reaction. The reaction mixture was filtered, and the residue was washed with hexane (3 mL). The residue was dried *in vacuo*. **14** was obtained as a white solid (15 mg, 0.0116 mmol, 61%). ^**1**^ **H NMR** (400 MHz, CD_3_CN) δ (ppm) = 7.56–7.52 (m, 2H, Ar–H), 7.43–7.37 (m, 4H, Ar–H), 7.11 (s, 2H, NCHCHN), 2.96 (hept, *J*_HH_ = 6.7 Hz, 2H, CH (*i*Pr)), 2.77 (hept, *J*_HH_ = 6.7 Hz, 2H, CH (*i*Pr)), 1.41 (d, *J*_HH_ = 6.8 Hz, 6H, CH_3_ (*i*Pr)), 1.35 (d, *J*_HH_ = 6.8 Hz, 6H, CH_3_ (*i*Pr)), 1.22 (d, *J*_HH_ = 6.9 Hz, 6H, CH_3_ (*i*Pr)), 1.17 (d, *J*_HH_ = 6.9 Hz, 6H, CH_3_ (*i*Pr)), 0.70 (d, ^3^ *J*_HP_ = 18.3 Hz, 9H, CH_3_ (*t*Bu)), 0.52 (d, ^2^ *J*_HP_ = 13.0 Hz, 3H, CH_3_ (Me)). ^**13**^ **C­{** ^**1**^ **H} NMR** (101 MHz, CD_3_CN) δ (ppm) = 163.5 (dd, ^2^ *J*_CP_ = 14 Hz, ^4^ *J*_CP_ = 3 Hz, βC (Au-CC)), 148.0 (d, ^2^ *J*_CP_ = 24 Hz, NCN), 147.9 (s, q-C (Ar-*i*Pr) (overlays with multiplet of CN, can be proven by NMR in CDCl_3_)), 132.8 (s, ipso-C (Ar)), 131.9 (s, C (Ar–H)), 125.6 (s, C (Ar–H)), 125.3 (s, C (Ar–H)), 119.5 (s, C (NCHCHN)), 86.7 (d, ^1^ *J*_CC_ = 142 Hz, αC (P-CC)), 33.1 (d, ^1^ *J*_CP_ = 96 Hz, q-C (*t*Bu)), 29.5 (d, *J*_CP_ = 8 Hz, CH (*i*Pr)), 25.6 (s, CH_3_ (*i*Pr)), 25.3 (s, CH_3_ (*i*Pr)), 24.0 (s, CH_3_ (*i*Pr)), 23.7 (d, ^3^ *J*_CP_ = 2 Hz, CH_3_ (*t*Bu)), 23.1 (s, *J*_CP_ = 2 Hz, CH_3_ (*i*Pr)), 13.2 (dd, ^1^ *J*_CP_ = 73 Hz, ^7^ *J*_CP_ = 2 Hz, CH_3_ (Me)). ^**31**^ **P NMR** (162 MHz, CD_3_CN) δ (ppm) = 8.2–7.8 (m). ^**31**^ **P­{** ^**1**^ **H} NMR** (162 MHz, CD_3_CN) δ (ppm) = 8.0 (s), 8.0 (s).

Determination of the HEP parameter: Reaction of **6** with [AuCl­(*i*Pr_2_-bimy)]: [ClAu­(*i*Pr_2_-bimy)] (5 mg, 0.0115 mmol, 1 equiv) and **6** (7 mg, 0.0115 mmol, 1 equiv) were dissolved in THF (1 mL). A color change to red was observed instantaneously. All volatiles were removed *in vacuo*. The residue was examined with ^1^H, ^13^C, and ^31^P NMR indicating the formation of a mixture of homoleptic and heteroleptic Au­(I) complexes. [Au­(*i*Pr_2_-bimy)_2_]Cl was identified by comparison with the reported complex [Au­(*i*Pr_2_-bimy)_2_]­Br.^60^ Residual [ClAu­(*i*Pr_2_-bimy)] was identified by comparison of literature-reported data.^61^

^**1**^ **H NMR** (400 MHz, CDCl_3_) δ (ppm) = 7.78–7.73 (m, 4H, Ar–H (NHC), [Au­(*i*Pr_2_-bimy)_2_]­[Cl]), 7.65–7.63 (m, 2H+2H, Ar–H (NHC), [ClAu­(*i*Pr_2_-bimy)]+**15**), 7.49–7.45 (m, 4H, Ar–H (NHC), [Au­(*i*Pr_2_-bimy)_2_]­[Cl]), 7.38–7.35 (m, 2H+2H, Ar–H (NHC), [ClAu­(*i*Pr_2_-bimy)]+**15**), 6.78 (s, 4H, NCHCHN, **15**), 6.68 (s, 8H, NCHCHN, **13**), 5.50 (hept, *J*_HH_ = 7.0 Hz, 2H, CH (*i*Pr (NHC), [ClAu­(*i*Pr_2_-bimy)])), 5.42 (hept, *J*_HH_ = 7.0 Hz, 4H, CH (*i*Pr (NHC), [Au­(*i*Pr_2_-bimy)_2_]­[Cl])), 5.27 (hept, *J*_HH_ = 7.0 Hz, 2H, CH (*i*Pr (NHC), **15**)), 2.21–2.01 (m, 8H+16H, CH_2_ (*t*Oct), **13**+**15**), 1.86 (d, *J*_HH_ = 7.2 Hz, 24H, CH_3_ (*i*Pr (NHC), [Au­(*i*Pr_2_-bimy)_2_]­[Cl])), 1.77–1.71 (m, 12H+54H+40H, CH_3_ (*i*Pr (NHC) + C­(CH_3_)_2_ (*t*Oct) + PCH_3_, [ClAu­(*i*Pr_2_-bimy)]+**13**+**15**)), 0.94 (s, 36H, C­(CH_3_)_3_ (*t*Oct), **15**), 0.89 (s, 36H, C­(CH_3_)_3_ (*t*Oct), **13**).

^**13**^ **C­{** ^**1**^ **H} NMR** (101 MHz, CDCl_3_) δ (ppm) = 186.9 (s, C_Carbene_ (NHC), [Au­(*i*Pr_2_-bimy)_2_]­[Cl]), 143.9 (d, ^2^ *J*_CP_ = 16 Hz, NCN (NHI), **13**), 132.8 (s, C (Ar, NHC), [Au­(*i*Pr_2_-bimy)_2_]­[Cl]), 125.0 (s, C (Ar, NHC), [Au­(*i*Pr_2_-bimy)_2_]­[Cl]), 124.3 (s, C (Ar, NHC), **15**), 124.0 (s, C (Ar, NHC), [ClAu­(*i*Pr_2_-bimy)]), 113.3 (s, C (Ar, NHC), [Au­(*i*Pr_2_-bimy)_2_]­[Cl]), 113.2 (s, C (Ar, NHC), **15**), 112.9 (s, C (Ar, NHC), [ClAu­(*i*Pr_2_-bimy)]), 112.3 (s, C (NCHCHN (NHI), **15**), 112.0 (s, C (NCHCHN (NHI), **13**), 62.1 (s, qC­(CH_3_)_2_) (*t*Oct), **15**), 61.9 (s, qC­(CH_3_)_2_ (*t*Oct), **13**), 54.5 (s, CH (*i*Pr, NHC),), [ClAu­(*i*Pr_2_-bimy)]+**15**), 54.0 (s, CH (*i*Pr, NHC), [Au­(*i*Pr_2_-bimy)_2_]­[Cl]), 51.4 (s, CH_2_ (*t*Oct), **15**), 51.2 (s, CH_2_ (*t*Oct), **13**), 32.1 (s, qC­(CH_3_)_3_ (*t*Oct), **15**), 32.0 (s, qC­(CH_3_)_3_ (*t*Oct), **13**), 31.4 (s, (CH_3_)_3_ (*t*Oct), **13**), 31.4 (s, (CH_3_)_3_ (*t*Oct), **15**), 30.8 (d, ^5^ *J*_CP_ = 19 Hz, (CH_3_)_2_ (*t*Oct), **13**), 23.6 (s, CH_3_ (*i*Pr, NHC), **15**), 22.8 (s, CH_3_ (*i*Pr, NHC), [Au­(*i*Pr_2_-bimy)_2_]­[Cl]), 22.1 (d, ^1^ *J*_CP_ = 64 Hz, CH_3_ (Me), **13**), 21.5 (s, CH_3_ (*i*Pr, NHC), [ClAu­(*i*Pr_2_-bimy)]).

It is noted that some of the quaternary carbons are not found in ^13^C NMR due to low concentrations in the mixture and coupling to phosphorus.

^**31**^ **P NMR** (162 MHz, CDCl_3_) δ (ppm) = −34.4 (q, ^2^ *J*_HP_ = 13 Hz, **13**), −35.2 (q, ^2^ *J*_HP_ = 14 Hz, **15**).

^**31**^ **P­{** ^**1**^ **H} NMR** (162 MHz, CDCl_3_) δ (ppm) = −34.4 (s, **13**), −35.2 (s, **15**).

Determination of the HEP parameter: Reaction of **10** with [AuCl­(*i*Pr_2_-bimy)]: [AuCl­(*i*Pr_2_-bimy)] (9 mg, 0.0207 mmol, 1 equiv) and **10** (11 mg, 0.0207 mmol, 1 equiv) were dissolved in THF (1 mL). A white precipitation formed instantly. The supernatant was decanted, and the residue was dried *in vacuo*. The residue was examined with ^1^H, ^13^C, and ^31^P NMR, which confirms the formation of the gold­(I) complexes [Au­(**10**)_2_]Cl (**14**), [Au­(**10**)­(*i*Pr_2_-bimy)]Cl (**16**), and [Au­(*i*Pr_2_-bimy)_2_]­Cl^60^ according to their indicative ^1^H and ^13^C NMR resonances. The ^31^P­{^1^H} NMR spectrum further confirms the formation of **14** and **16**. (Note that the two diastereomers of **14** appear as two separate resonances in CD_3_CN (8.0 ppm, 8.0 ppm) and as one signal in CDCl_3_ (7.4 ppm); see Figures S139 and S140). It is noted that upon exchange of chloride with PF_6_^–^ counteranions using either KPF_6_ or TlPF_6_, the same product mixture was obtained. Attempts to separate the complexes by column chromatography (alumina, CH_2_Cl_2_/MeOH) or recrystallization (THF) were unsuccessful. However, single crystals suitable for an SCXRD analysis were obtained, which reveal the literature-known homoleptic complex [Au­(*i*Pr_2_-bimy)_2_]­PF_6_ and the desired complex [Au­(**10**)­(*i*Pr_2_-bimy)]­PF_6_ **16**′.

See above for the NMR data for isolated complex **14**. The NMR data for complex [Au­(*i*Pr_2_-bimy)_2_]Br is reported in the literature.^60^ Assignment of the resonances of **14**, **16**, and [Au­(*i*Pr_2_-bimy)_2_]Cl in the mixture:

^**1**^ **H NMR** (400 MHz, CDCl_3_) δ (ppm) = 7.77–7.74 (m, 4H, Ar–H (NHC), [Au­(*i*Pr_2_-bimy)_2_]­[Cl]), 7.68–7.66 (m, 4H, Ar–H (NHC), **16**), 7.54–7.29 (m, 4H+6H+4H, Ar–H (NHC+NHI), **14**+**16**+[Au­(*i*Pr_2_-bimy)_2_]­[Cl]), 6.94 (s, 2H, NCHCHN, **16**), 6.86 (s, 2H, NCHCHN, **14**), 5.41 (hept, *J*_HH_ = 7.0 Hz, 4H, CH (*i*Pr (NHC), [Au­(*i*Pr_2_-bimy)_2_]­[Cl])), 5.32 (hept, *J*_HH_ = 7.0 Hz, 2H, CH (*i*Pr (NHC), **16**)), 2.96 (hept, *J*_HH_ = 7.1 Hz, 2H+1H, CH (*i*Pr (NHI), **14**+**16**)), 2.77 (hept, *J*_HH_ = 6.9 Hz, 2H+1H, CH (*i*Pr (NHI), **14**+**16**)), 1.86 (d, *J*_HH_ = 6.9 Hz, 24H, CH_3_ (*i*Pr (NHC), [Au­(*i*Pr_2_-bimy)_2_]­[Cl])), 1.77 (d, *J*_HH_ = 7.0 Hz, 12H, CH_3_ (*i*Pr (NHC), **16**)), 1.43 (d, *J*_HH_ = 6.8 Hz, 3H, CH_3_ (*i*Pr (NHI), **16**)), 1.41 (d, *J*_HH_ = 6.6 Hz, 6H, CH_3_ (*i*Pr (NHI), **16**)), 1.37 (d, *J*_HH_ = 6.8 Hz, 3H, CH_3_ (*i*Pr (NHI), **16**)), 1.34 (d, *J*_HH_ = 6.8 Hz, 6H, CH_3_ (*i*Pr (NHI), **14**)), 1.24 (m, 6H+3H, CH_3_ (*i*Pr (NHI), **14**+**16**)), 1.19 (m, 6H+3H, CH_3_ (*i*Pr (NHI), **14**+**16**)), 0.77 (d, ^3^ *J*_HP_ = 18.5 Hz, 9H, CH_3_ (*t*Bu, **16**)), 0.70 (d, ^3^ *J*_HP_ = 18.3 Hz, 9H, CH_3_ (*t*Bu, **14**)), 0.58 (d, ^2^ *J*_HP_ = 12.9 Hz, 3H, CH_3_ (Me, **16**)), 0.49 (d, ^2^ *J*_HP_ = 12.9 Hz, 3H, CH_3_ (Me, **14**)).

^**13**^ **C­{** ^**1**^ **H} NMR** (101 MHz, CDCl_3_) δ (ppm) = 188.9 (d, ^4^ *J*_CP_ = 3 Hz, C_Carbene_ (NHC), **16**), 186.9 (s, C_Carbene_ (NHC), [Au­(*i*Pr_2_-bimy)_2_]­[Cl]), 148.0 (m, NCN + q–C (Ar-*i*Pr, NHI), **14**+**16**), 132.7 (s, C (Ar, NHC), [Au­(*i*Pr_2_-bimy)_2_]­[Cl]), 132.7 (s, C (Ar, NHC), **16**), 131.7 (s, ipso-C (Ar, NHI), **14**), 131.7 (s, ipso-C (Ar, NHI), **16**), 131.4 (s, C (Ar–H, NHI), **16**), 131.3 (s, C (Ar–H, NHI), **14**), 125.0 (s, C (Ar, NHC), [Au­(*i*Pr_2_-bimy)_2_]­[Cl]), 124.8 (s, C (Ar, NHC), **16**), 124.8 (s, C (Ar–H, NHI), **14**), 124.7 (s, C (Ar–H, NHI), **16**), 124.6 (s, C (Ar–H, NHI), **14**), 124.5 (s, C (Ar–H, NHI), **16**), 118.5 (s, C (NCHCHN), **16**), 118.2 (s, C (NCHCHN, **14**)), 113.3 (s, C (Ar, NHC), [Au­(*i*Pr_2_-bimy)_2_]­[Cl]), 113.2 (s, C (Ar, NHC), **16**), 54.0 (s, CH (*i*Pr, NHC), [Au­(*i*Pr_2_-bimy)_2_]­[Cl]), 53.8 (s, CH (*i*Pr, NHC), **16**), 32.7 (d, ^1^ *J*_CP_ = 95 Hz, q-C (*t*Bu), **14**+**16**), 28.9 (m, *J*_CP_ = 8 Hz, CH (*i*Pr, NHI), **14**+**16**), 25.7 (s, CH_3_ (*i*Pr, NHI), **14**+**16**), 25.3 (s, CH_3_ (*i*Pr, NHI), **14**+**16**), 23.8 (s, CH_3_ (*i*Pr, NHI), **14**+**16**), 23.5 (s, *J*_CP_ = 2 Hz, CH_3_ (*i*Pr, NHI), **14**+**16**), 23.1 (d, ^3^ *J*_CP_ = 2 Hz, CH_3_ (*t*Bu), **16**), 23.0 (s, CH_3_ (*t*Bu), **14**), 23.1 (s, CH_3_ (*i*Pr, NHC), [Au­(*i*Pr_2_-bimy)_2_]­[Cl]), 22.4 (s, CH_3_ (*i*Pr, NHC), **16**), 12.8 (d, ^1^ *J*_CP_ = 73 Hz, CH_3_ (Me), **16**).

It is noted that some of the quaternary carbons are not found in ^13^C NMR due to low concentrations in the mixture and coupling to phosphorus.

^**31**^ **P NMR** (162 MHz, CDCl_3_) δ (ppm) = 7.5 (m, **14**+**15**).

^**31**^ **P­{** ^**1**^ **H} NMR** (162 MHz, CDCl_3_) δ (ppm) = 7.5 (s, **15**), 7.4 (s, **14**).

## Computational Section

The calculation followed a method reported by Wagler in 2009.^22^ The geometry optimizations and frequency calculations were carried out using the B3LYP^62,63^ functional and the 6-311G­(d)^64,65^ basis set implemented in Gaussian 16.^66–69^ The π-acceptor, σ-donor, and π-donor orbitals were selected with the most similar topology to those of CO, CNMe, and CCPPh_3_. For the π-acceptor and π-donor orbitals, two orthogonal orbitals were found, respectively. In case of the π-donor orbital, the highest lying orbital with suitable orbital coefficients was chosen. For the π-acceptor orbitals, the lowest lying orbital with suitable orbital coefficients was chosen.^21^ The energies are not always on the same level of those orthogonal orbitals as for large systems orbital mixing is observed. The topologies of the orbitals are displayed with Chemcraft.^70^

Natural bond orbital analysis was performed at the same level of theory (B3LYP/6-311G­(d)). Results for selected calculated atomic charges of P, C_α_, and C_β_ as well as Wiberg bond indices for P–C_α_ and C_α_–C_β_ are presented in the Supporting Information.

Using the previous calculated geometries (B3LYP/6-311G­(d)), all structures were reoptimized with the B3LYP^62,63^ functional and the 6-311+G­(2df,p)^67–69,71–77^ basis set along with Grimme’s dispersion correction (D3)^78^ implemented in Gaussian 16.^66^ This followed a method reported by Sundermeyer and co-workers in 2019.^79^ A frequency calculation at the same level of theory was performed of the final structure to obtain Δ*H* and confirm the calculated structure is a local minimum.

To determine the proton affinity (PA) in the gas-phase the enthalpy of the following reaction is calculated:^80^

$$\Delta H:B+H^{+}\to {\mathrm{BH}}^{+}$$

PA=−ΔH

$$\Delta H=H\left({\mathrm{BH}}^{+}\right)-H\left(B\right)-H\left(H^{+}\right)$$

EDA-NOCV analysis^54–57^ was performed at the same level of theory (B3LYP/6-311G­(d)). The calculation was performed using Orca 6.1.1.^81–94^ For details, see Supporting Information.

## Supplementary Material



## Footnotes

$ Notably, if **10** gets dissolved during washing with Et_2_O, it can be conveniently recovered by recrystallization from the filtrate at −40 °C

## Notes

Synthetic details, synthetic procedures, light and temperature stability, hydrolysis, NMR spectra, mass spectrometry data, elemental analysis, IR data, crystallographic data, computational details, depicted relevant frontier orbitals, and atomic coordinates of the optimized geometries (PDF)

## Abbreviations

NHI: *N*-heterocyclic imines
NHC: *N*-heterocyclic carbenes
SCXRD: single-crystal X-ray diffraction
NMR: nuclear magnetic resonance
IR: infrared
MS: mass spectrometry
DFT: density functional theory
TEP: Tolman electronic parameter
HEP: Huynh electronic parameter
RNC: isocyanides
dipp: 2,4-diisopropylphenyl
*t*Bu: *tert*-butyl
*t*Oct: *tert*-octyl
HOMO: highest occupied molecular orbital
LUMO: lowest unoccupied molecular orbital
PA: gas-phase proton affinities
EDA-NOCV: energy decomposition analysis coupled with natural orbitals for chemical valence
