# Supplementary material for: Isolable Phosphonioacetylides as Strong Neutral Carbon Donors with Variable Steric Demand and Tunable Donor Properties
Source: Inorg Chem. 2026 May 12;65(20):11231–43. doi: 10.1021/acs.inorgchem.6c00919 (PMC13213908; doi:10.1021/acs.inorgchem.6c00919)
Supplement: Supplementary file 1 [file ic6c00919_si_001.pdf]

Supporting Information for

Isolable phosphonioacetylides as strong neutral carbon donors with variable steric demand and tunable donor properties

Franka Brylak,[a] Liane Perktold,[a] Lucas C. Torres,[a,b] Luis Stechele,[a] Klaus Wurst,[a] Michael Seidl,[a] Christopher B. Caputo,[b] and Fabian Dielmann[a]*

[a] Institute of General, Inorganic and Theoretical Chemistry, Universität Innsbruck, 6020 Innsbruck, Austria; Email: Fabian.Dielmann@uibk.ac.at

[b] Department of Chemistry, York University, 4700 Keele St, Toronto, ON M3J 1P3, Canada

Contents

Synthetic Details S4

Preparation of compounds 1-14 S5

I*t*Oct **1** S5

TMSNI*t*Oct **2** S8

$[P(NI\textit{t}Oct)_2][Cl]$ **3** S11

$P(CCH)(NI\textit{t}Oct)_2$ **4** S15

$[P(CCH)Me(NI\textit{t}Oct)_2][I]$ **5** S19

$P(CC)Me(NI\textit{t}Oct)_2$ **6** S24

PCl(NIdipp)*t*Bu **7** S30

P(CCH)(NIdipp)*t*Bu **8** S36

[P(CCH)Me(NIdipp)*t*Bu][I] **9** S43

P(CC)Me(NIdipp)*t*Bu **10** S50

PMe(NIdipp)tBu **11** S57

OPMe(NIdipp)tBu **12** S60

Light and temperature stability S64

Reactivity with water S68

$[Au((CC)PMe(NI\textit{t}Oct)_2)_2][Cl]$ **13** S73

$[Au((CC)PMe(NIdipp)\textit{t}Bu)_2][Cl]$ **14** S78

Reaction of **6** with $[AuCl(\textit{i}Pr_2\text{-bimy})]$ S84

Determination of the Huynh Electronic Parameter (HEP) for phosphonioacetlyide **10**: Reaction of **10** with $[AuCl(\textit{i}Pr_2\text{-bimy})]$ S87

X-ray diffraction studies S93

General S93

Single-crystal X-ray structure analysis of **1** S93
Single-crystal X-ray structure analysis of **2** S94
Single-crystal X-ray structure analysis of **3** S95
Single-crystal X-ray structure analysis of **4** S97
Single-crystal X-ray structure analysis of **5** S98
Single-crystal X-ray structure analysis of **6** S99
Single-crystal X-ray structure analysis of **8** S101
Single-crystal X-ray structure analysis of **9** S102
Single-crystal X-ray structure analysis of **10** S103
Single-crystal X-ray structure analysis of **12** S105
Single-crystal X-ray structure analysis of **14** S106
Single-crystal X-ray structure analysis of **16’** S107
DFT calculations S110
Calculation of the relative energies of the frontier orbitals corresponding to the donor and acceptor properties S110
Depicted relevant frontier orbitals S111
Natural Bond Orbital Analysis S115
Gas phase proton affinity S115
EDA-NOCV analysis S116
Calculated IR spectrum of **8** S117
Atomic coordinates of the optimized geometry of CO (B3LYP/6-311G(d)) S117
Atomic coordinates of the optimized geometry of CO (B3LYP-D3/6-311+G(2df,p)) S118
Atomic coordinates of the optimized geometry of CNMe (B3LYP/6-311G(d)) S118
Atomic coordinates of the optimized geometry of CNMe (B3LYP-D3/6-311+G(2df,p)) S118
Atomic coordinates of the optimized geometry of $CCPF_3$ (B3LYP/6-311G(d)) S118
Atomic coordinates of the optimized geometry of $CCPF_3$ (B3LYP-D3/6-311+G(2df,p)) S118
Atomic coordinates of the optimized geometry of $CCP(OMe)_3$ (B3LYP/6-311G(d)) S118
Atomic coordinates of the optimized geometry of $CCPMe_3$ (B3LYP/6-311G(d)) S119
Atomic coordinates of the optimized geometry of $CCPPh_3$ (**A**) (B3LYP/6-311G(d)) S119
Atomic coordinates of the optimized geometry of $CCP(NMe_2)_3$ (B3LYP/6-311G(d)) S120
Atomic coordinates of the optimized geometry of CCPMe*t*Bu(NIdipp) (**10**) (B3LYP/6-311G(d)) S121
Atomic coordinates of the optimized geometry of $CCPMe(NIdipp)_2$ (**B**) (B3LYP/6-311G(d)) S124

Atomic coordinates of the optimized geometry of CCPMe(NsI*t*Bu)$_2$ (**C**) (B3LYP/6-311G(d)) S127

Atomic coordinates of the optimized geometry of CCP(NIMe)$_3$ (B3LYP/6-311G(d)) S129

Atomic coordinates of the optimized geometry of CCP(NIMe)$_3$ (B3LYP-D3/6-311+G(2df,p)) S130

Atomic coordinates of the optimized geometry of $[HCO]^+$ (B3LYP-D3/6-311+G(2df,p)) S131

Atomic coordinates of the optimized geometry of $[HCNMe]^+$ (B3LYP-D3/6-311+G(2df,p)) S131

Atomic coordinates of the optimized geometry of $[HCCPF_3]^+$ (B3LYP-D3/6-311+G(2df,p)) S131

Atomic coordinates of the optimized geometry of $[HCCPMe_3]^+$ (B3LYP-D3/6-311+G(2df,p)) S132

Atomic coordinates of the optimized geometry of $[HCCPNIMe_3]^+$ (B3LYP-D3/6-311+G(2df,p)) S132

Atomic coordinates of the optimized geometry of IMe (B3LYP-D3/6-311+G(2df,p)) S133

Atomic coordinates of the optimized geometry of $[IMe]^+$ (B3LYP-D3/6-311+G(2df,p)) S134

Atomic coordinates of the optimized geometry of P(CCH)MeNIdipp (**8**) (B3LYP/6-311G(d)) S134

References S136

Synthetic Details

General remarks: Unless otherwise noted, all manipulations were performed under an inert atmosphere of dry argon, using standard Schlenk and drybox techniques. Dry and oxygen-free solvents were employed. All glassware was oven-dried at 150 °C prior to use. ^{1}H, ^{13}C, and ^{31}P NMR spectra were recorded at 300 K on BRUKER ARX 300 MHZ, BRUKER AVANCE (IV) NEO 400 MHZ, 400 MHZ BRUKER AVANCE 4 NEO SPECTROMETER, 600 MHZ AVANCE II+ SPECTROMETER or 700 MHZ AVANCE 4 NEO SPECTROMETER. Chemical shifts (δ) are given in parts per million (ppm) relative to $SiMe_4$ (^{1}H, ^{13}C), 85% H_3PO_4 (^{31}P), and they were referenced to the residual solvent signals (C_6D_6: ^{1}H δ_H = 7.16, ^{13}C δ_C = 128.06; CD_3CN: δ_H = 1.94, ^{13}C δ_C = 118.26) or internally by the instrument after locking and shimming to the deuterated solvent (^{31}P). NMR multiplicities are abbreviated as follows: s = singlet, d = doublet, t = triplet, p = pentet, hept = heptet, dh = decet, m = multiplet, br = broad signal. Mass spectrometry was recorded using an ORBITRAP (THERMO SCIENTIFIC) SPECTROMETER at the University of Innsbruck or a JEOL ACCUTOF PLUS 4G at the University of Toronto. Electronspray ionization (ESI, ORBITRAP (THERMO SCIENTIFIC) SPECTROMETER) or direct analysis in real time ionization (DART, JEOL ACCUTOF PLUS 4G) were used as ionization methods. IR spectra were obtained on a BRUKER ALPHA SPECTROMETER. I*t*Oct·HBF_4[1], TMSNIdipp[2] and [Au(tht)Cl][3] were prepared following a literature procedure. All other compounds were purchased from commercial sources (ABCR, Acros Organics, Sigma Aldrich, TCI) and used as received.

Preparation of compounds 1-14

I*t*Oct **1**

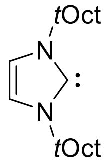

THF (50 mL) was slowly added to a stirred mixture of I*t*Oct·HBF_4 (10.20 g, 30.9 mmol, 1.00 eq.) and KO*t*Bu (4.16 g, 37.1 mmol, 1.20 eq.) in a Schlenk tube at −78 °C. The reaction mixture was slowly warmed to room temperature and stirred overnight. All volatiles were removed *in vacuo*. Compound **1** was extracted from the residual solid with *n*-hexane (40 mL). After filtration, the solvent was removed *in vacuo* to yield the carbene **1** as a white solid (7.85 g, 26.9 mmol, 87%).

^{1}H NMR (400 MHz, C_6D_6) δ (ppm) = 6.74 (s, 2H, CH), 1.87 (s, 4H, CH_2), 1.61 (s, 12H, $(CH_3)_2$), 0.87 (s, 18H, $C(CH_3)_3$).

^{13}C{^{1}H} NMR (101 MHz, C_6D_6) δ (ppm) = 214.7 (s, NCN), 115.6 (s, CH), 59.2 (s, q$C(CH_3)_2$), 55.5 (s, CH_2), 31.9 (s, q$C(CH_3)_3$), 31.8 (s, $(CH_3)_2$), 31.3 (s, $(CH_3)_3$).

HRMS (ESI, CH_3CN): m/z = 293.2946 ($[M+H]^+$, calculated: 293.2951).

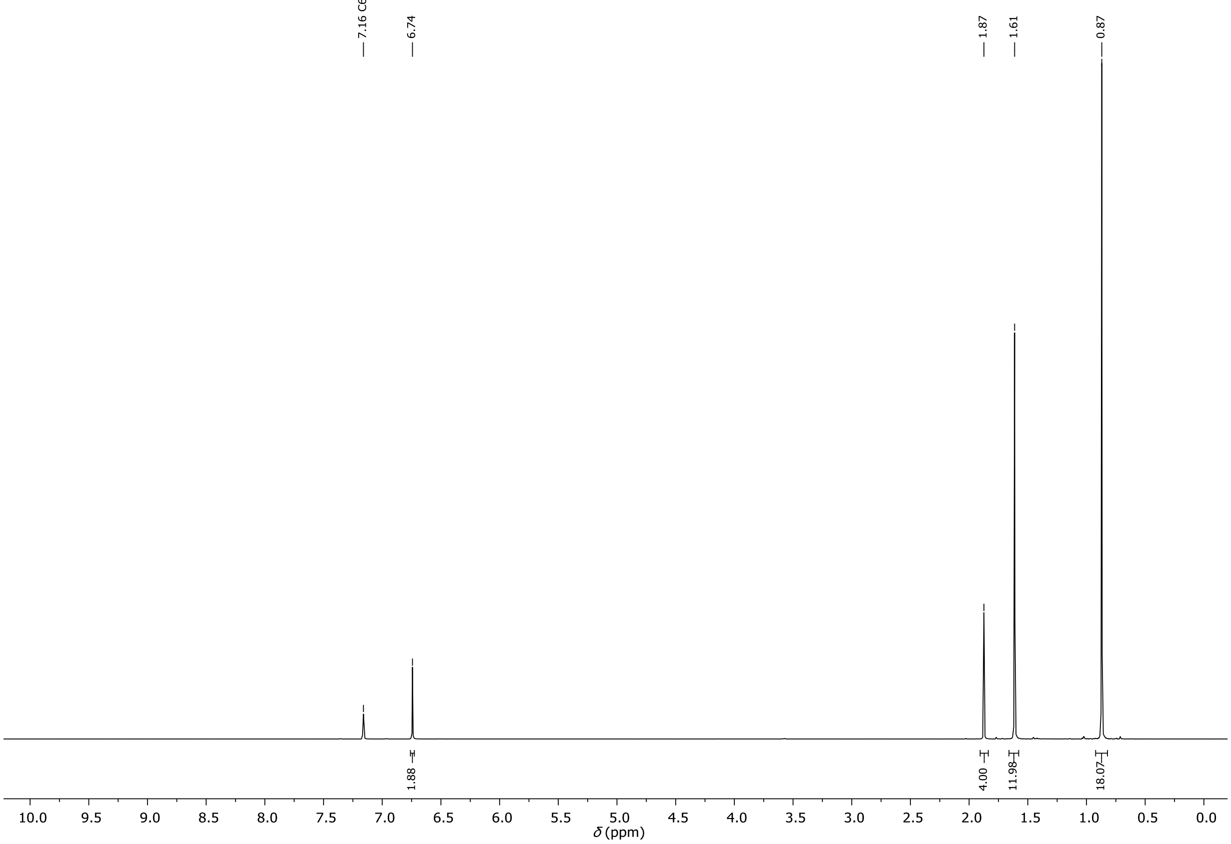

Figure S1: ^{1}H NMR spectrum (400 MHz, C_6D_6) of **1**.

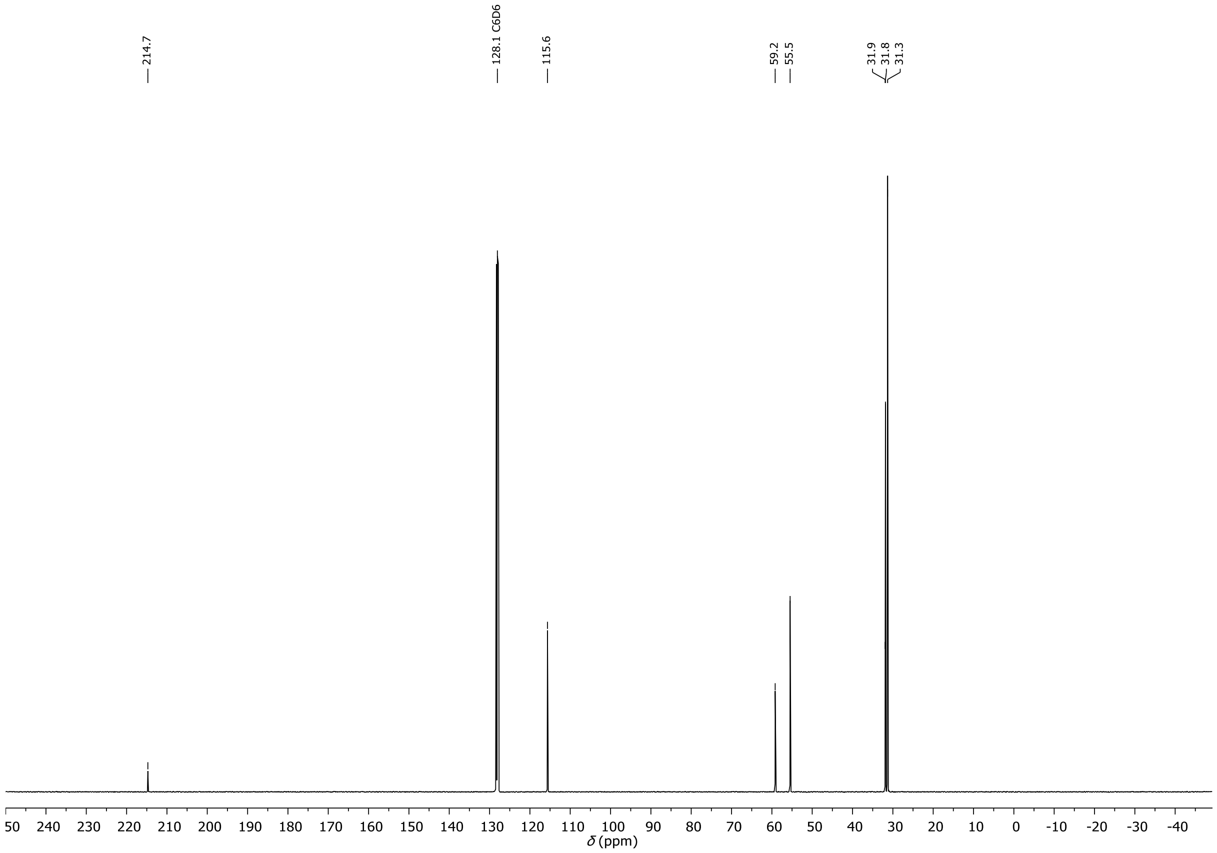

Figure S2: $^{13}C\{^{1}H\}$ NMR (101 MHz, C_6D_6) of **1**.

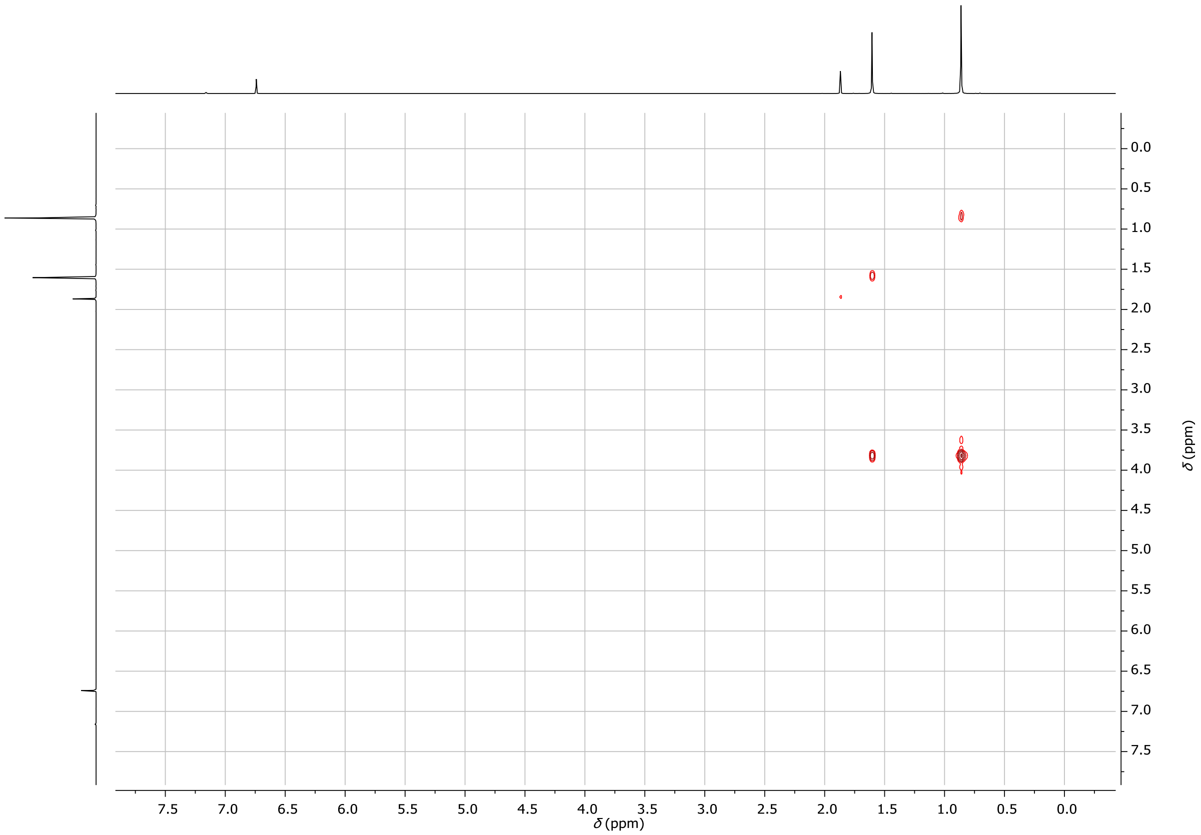

Figure S3: COSY (^{1}H, ^{1}H) NMR spectrum (400 MHz, 400 MHz, C_6D_6) of **1**.

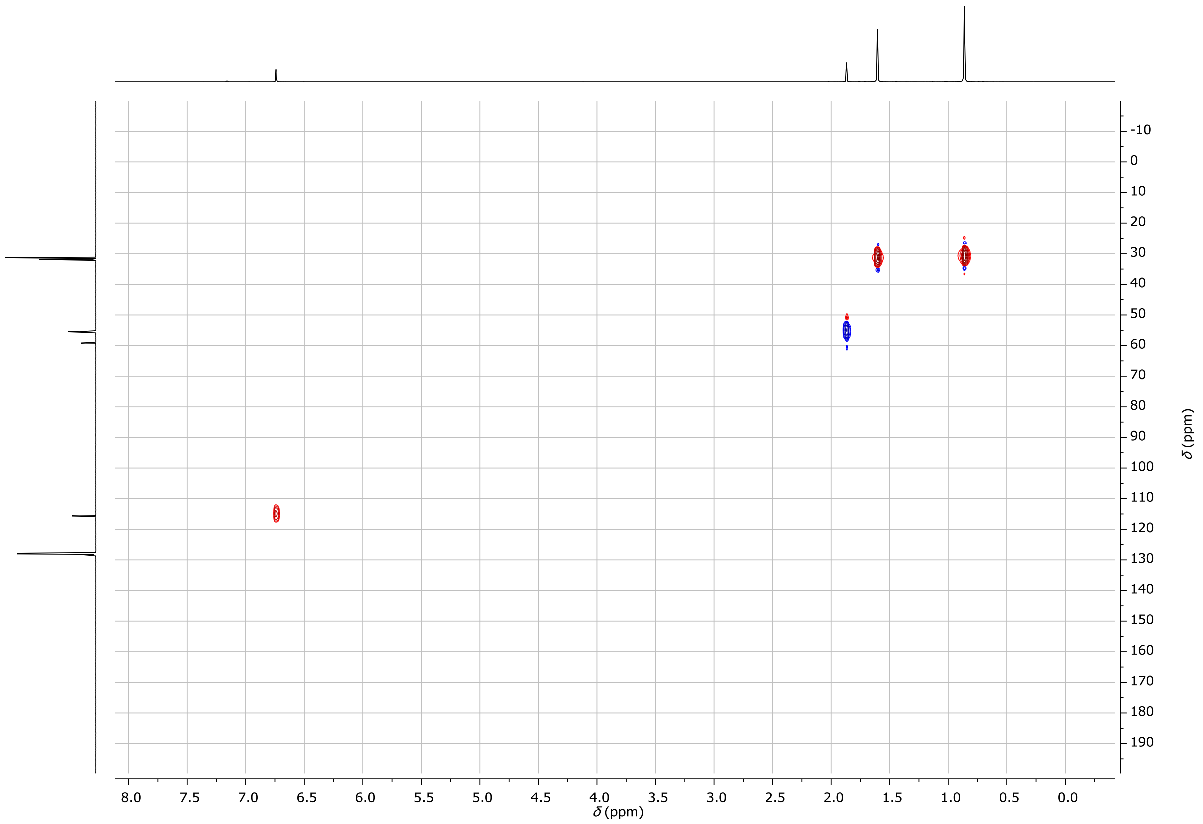

Figure S4: HSQC (^1H, ^{13}C) NMR spectrum (400 MHz, 101 MHz, C_6D_6) of **1**.

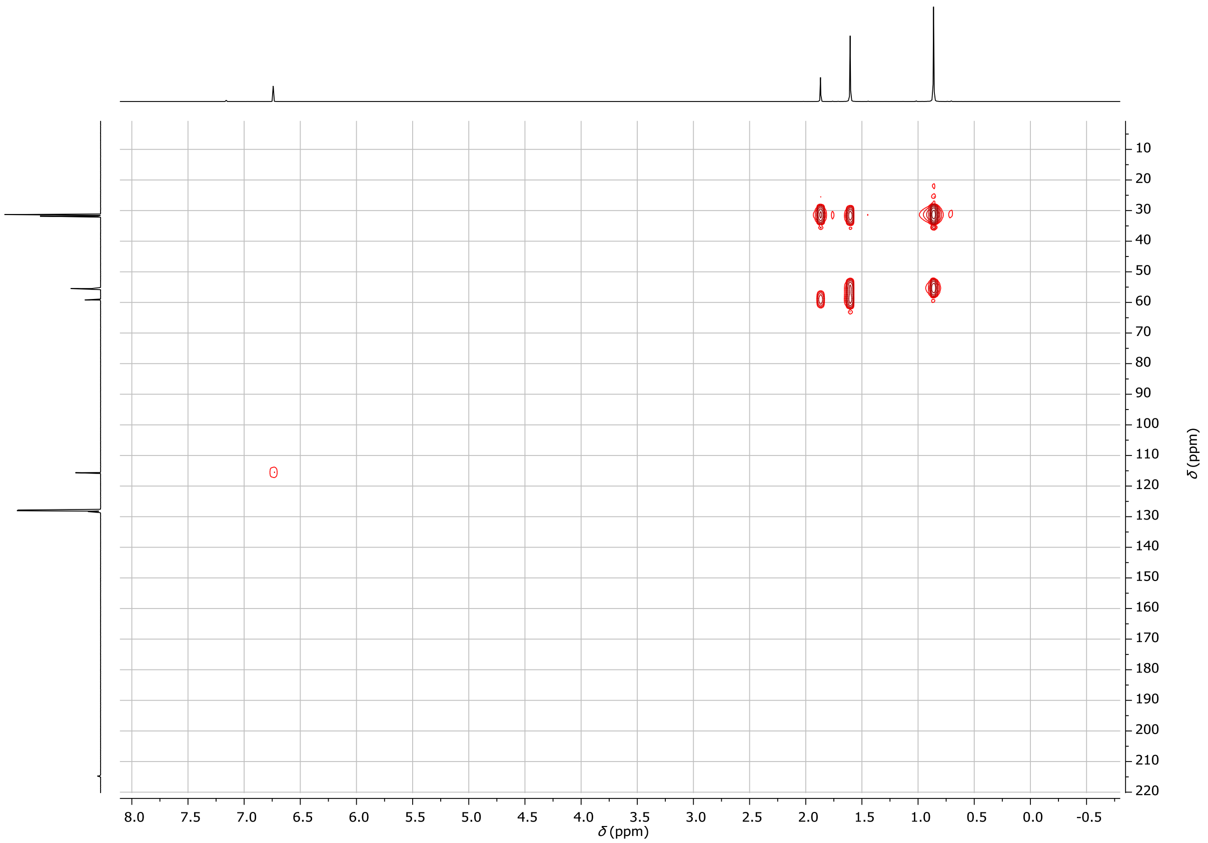

Figure S5: HMBC (^1H, ^{13}C) NMR spectrum (400 MHz, 101 MHz, C_6D_6) of **1**.

TMSNI*t*Oct **2**

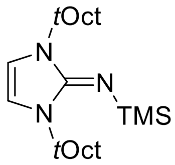

Trimethylsilyl azide (2.00 mL, 15.23 mmol, 1.50 eq.) was slowly added to a solution of **1** (2.97 g, 10.15 mmol, 1.00 eq.) in toluene (60 mL) in a Schlenk flask equipped with an aircondenser and an overpressure bubbler. The reaction mixture was heated to reflux at 120 °C for 15 d. The solvent was removed *in vacuo* which gave a viscous oil. To facilitate the evaporation of toluene, the residue was redissolved in pentane (20 mL) and all volatiles were removed *in vacuo* to afford imine **2** as an off-white solid (3.31 g, 8.72 mmol, 86%).

Note that the reaction time can be shortened to four days by using xylene instead of toluene as solvent. As the reaction time also depends on the scale of the reaction, it is recommended to monitor the reaction progress by ^{1}H NMR or ^{13}C NMR spectroscopy.

^{1}H NMR (400 MHz, C_6D_6) δ (ppm) = 6.02 (s, 2H, CH), 2.07 (s, 4H, CH_2), 1.38 (s, 12H, $(CH_3)_2$), 0.93 (s, 18H, $C(CH_3)_3$), 0.54 (s, 9H, $Si(CH_3)_3$).

^{13}C{^{1}H} NMR (101 MHz, C_6D_6) δ (ppm) = 139.6 (s, NCN), 107.7 (s, CH), 57.9 (s, $qC(CH_3)_2$), 47.7 (s, CH_2), 31.9 (s, $qC(CH_3)_3$), 31.4 (s, $(CH_3)_3$), 29.7 (s, $(CH_3)_2$), 5.0 (s, $Si(CH_3)_3$).

^{29}Si DEPT-19.5 NMR (80 MHz, C_6D_6) δ (ppm) = -29.1 (s).

HRMS (ESI, CH_3CN): m/z = 308.3058 ($[M+H]^+$, calculated: 380.3456).

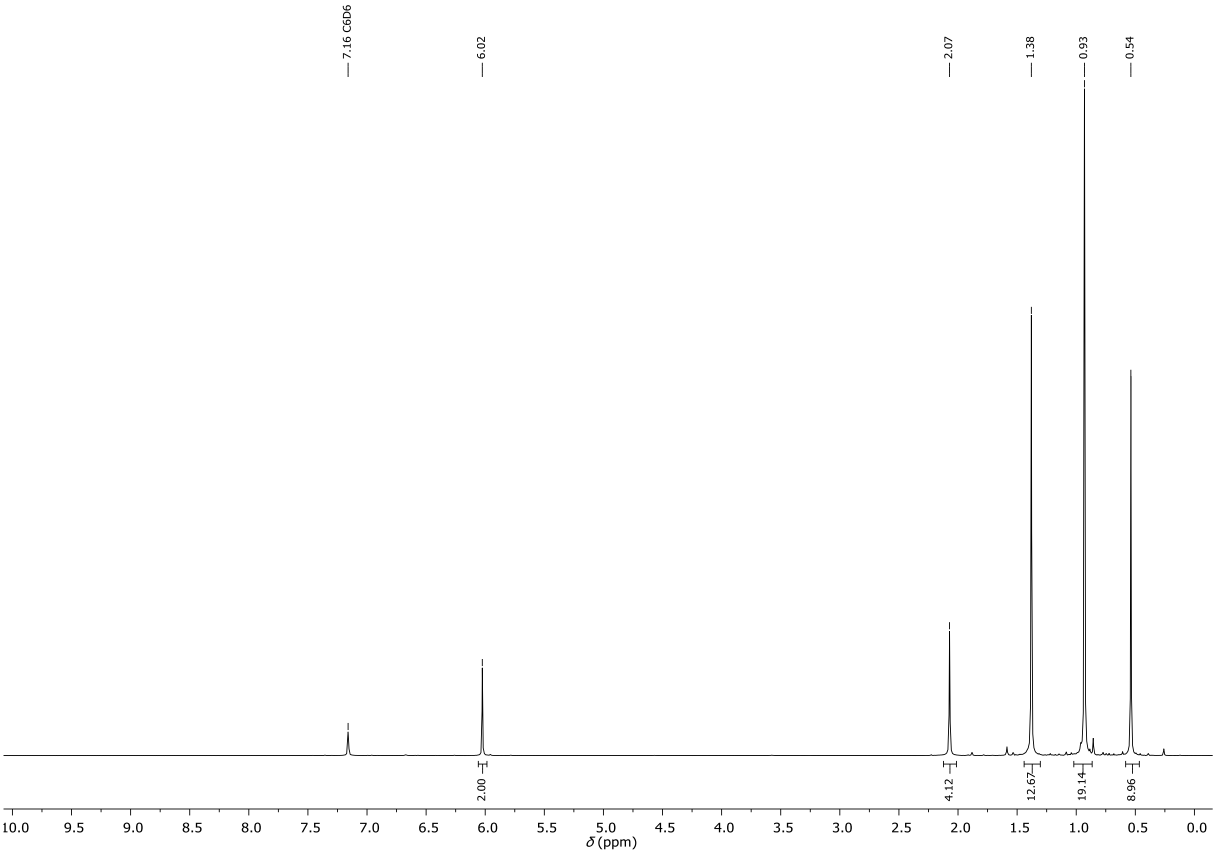

Figure S6: ^{1}H NMR spectrum (400 MHz, C_6D_6) of **2**.

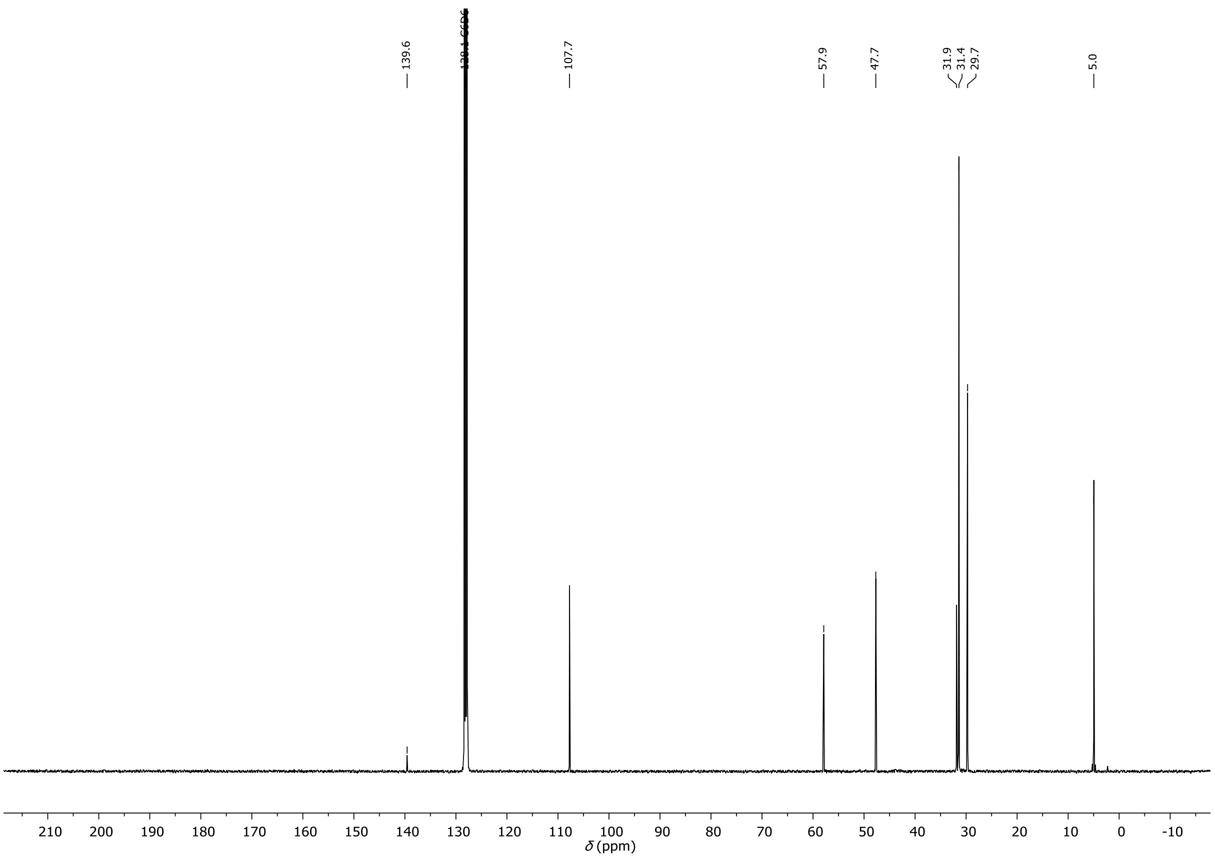

Figure S7: $^{13}C\{^{1}H\}$ NMR spectrum (101 MHz, C_6D_6) of **2**.

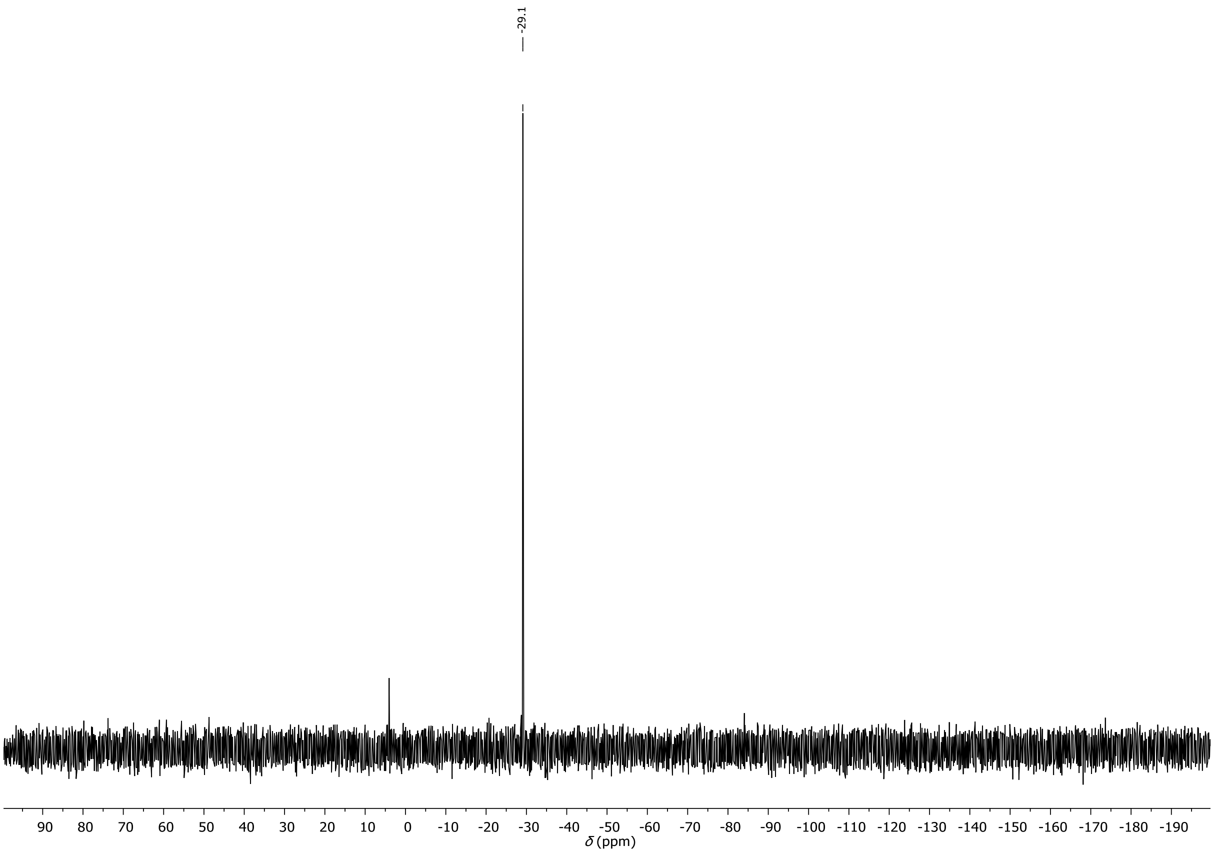

Figure S8: ^{29}Si DEPT-19.5 NMR spectrum (80 MHz, C_6D_6) of **2**.

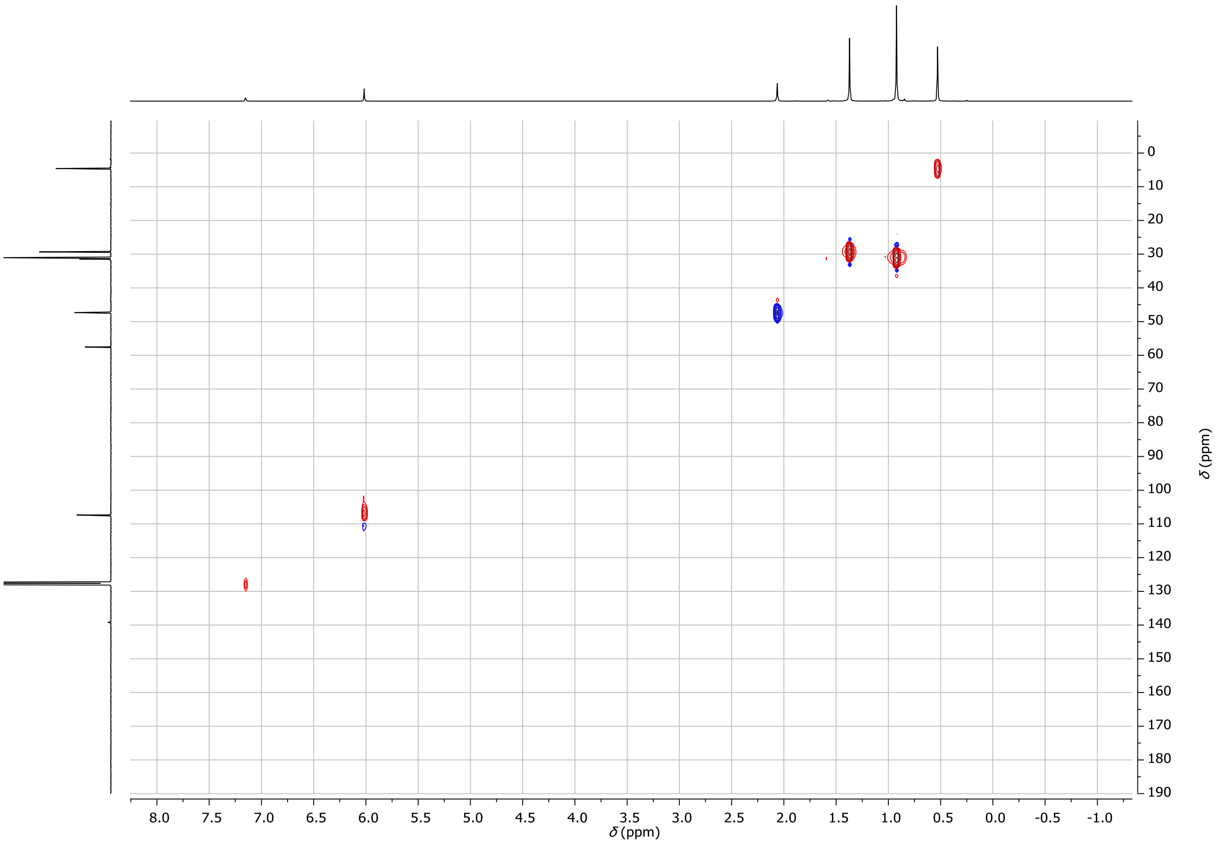

Figure S9: HSQC (^{1}H, ^{13}C) NMR spectrum (400 MHz, 101 MHz, C_6D_6) of **2**.

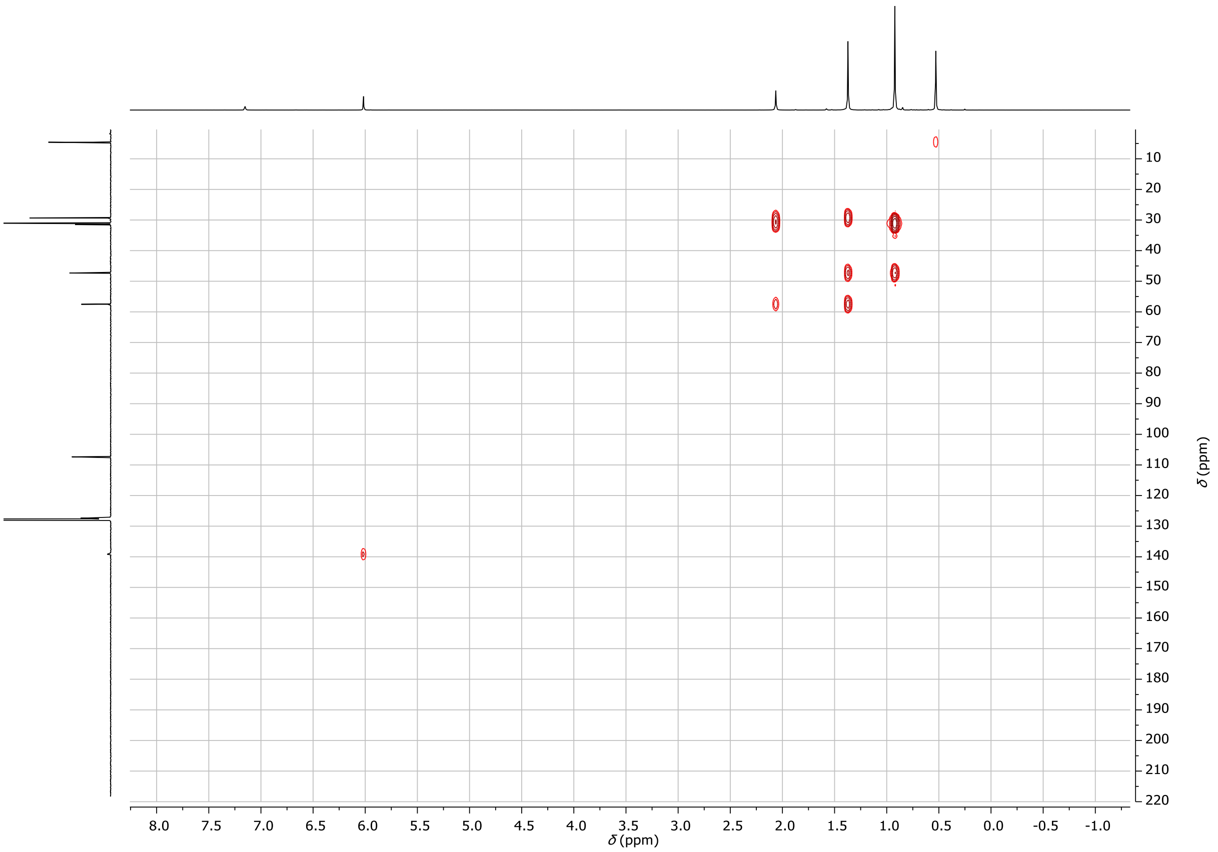

Figure S10: HMBC (^{1}H, ^{13}C) NMR spectrum (400 MHz, 101 MHz, C_6D_6) of **2**.

[P(NI*t*Oct)$_2$][Cl] **3**

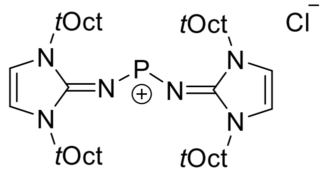

PCl_3 in toluene (2.4 mL, 1.05 M, 2.52 mmol, 1.00 eq.) was added dropwise to a solution of **2** (2.00 g, 5.27 mmol, 2.10 eq.) in THF (60 mL) in a Schlenk tube, resulting in the precipitation of **3** as a yellow solid. The suspension was stirred overnight at room temperature to complete the reaction and then all volatiles were removed *in vacuo*. The solid residue was washed with diethyl ether (2x 20 mL) to remove the excess of **2**. After drying the remaining solid *in vacuo,* product **3** was obtained as a bright yellow solid (1.52 g, 2.24 mmol, 90%).

^{1}H NMR (400 MHz, CD_3CN) δ (ppm) = 7.08 (s, 2H, CH), 2.15 (s, 4H, CH_2), 1.70 (s, 12H, $(CH_3)_2$), 0.85 (s, 18H, $C(CH_3)_3$).

^{1}H{^{31}P} NMR (400 MHz, CD_3CN) δ (ppm) = 7.08 (s, 2H, CH), 2.15 (s, 4H, CH_2), 1.70 (s, 12H, $(CH_3)_2$), 0.85 (s, 18H, $C(CH_3)_3$).

^{13}C{^{1}H} NMR (101 MHz, CD_3CN) δ (ppm) = 145.2 (s, NCN), 114.9 (s, CH), 62.9 (s, q$C(CH_3)_2$), 51.4 (d, $^5J_{CP}$ = 4 Hz, CH_2), 32.6 (s, q$C(CH_3)_3$), 31.2 (d, $^7J_{CP}$ = 1 Hz, $(CH_3)_3$), 30.8 (d, $^5J_{CP}$ = 3 Hz, $(CH_3)_2$).

^{31}P NMR (162 MHz, CD_3CN): δ (ppm) = 278.7 (s).

^{31}P{^{1}H} NMR (162 MHz, CD_3CN): δ (ppm) = 278.7 (s).

HRMS (ESI, CH_3CN): m/z = 643.5542 ([M-Cl]$^+$, calculated: 643.5551).

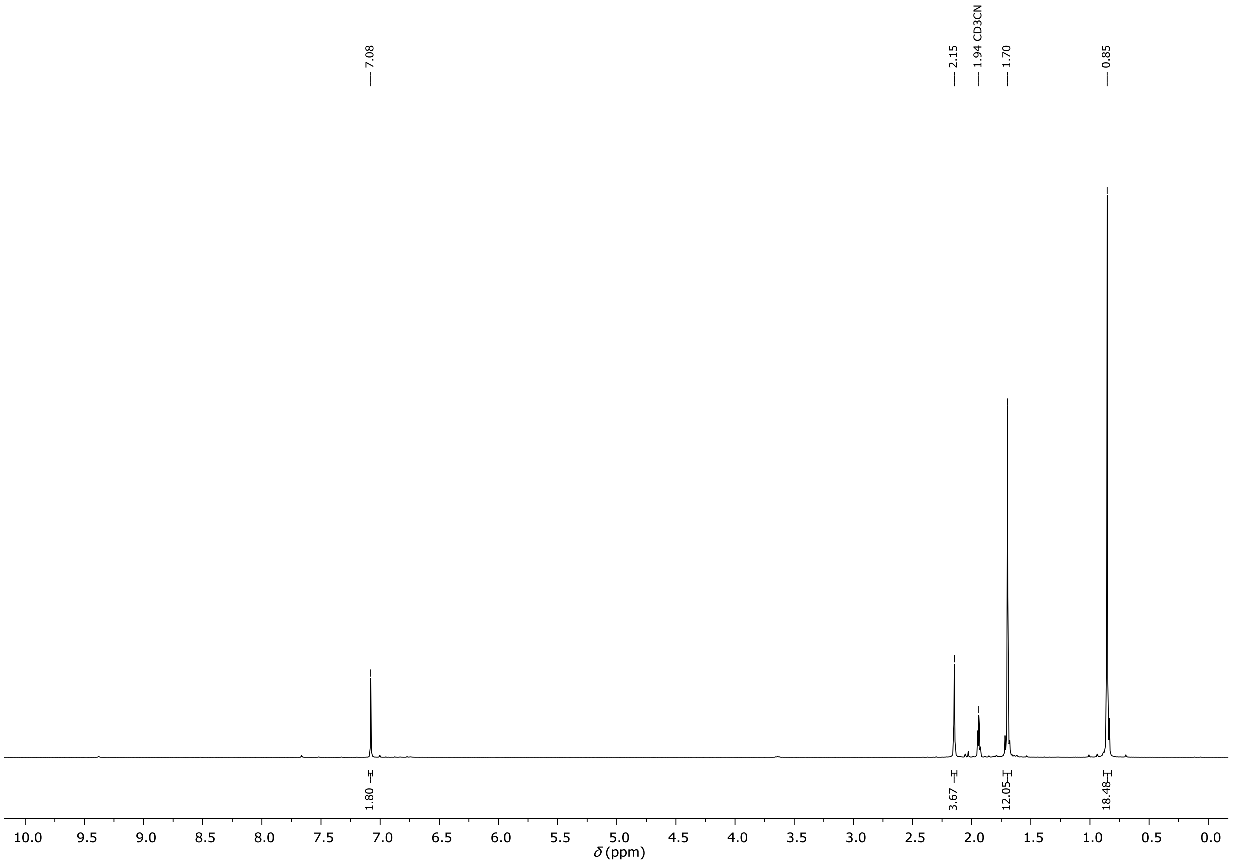

Figure S11: ^{1}H NMR spectrum (400 MHz, CD_3CN) of **3**.

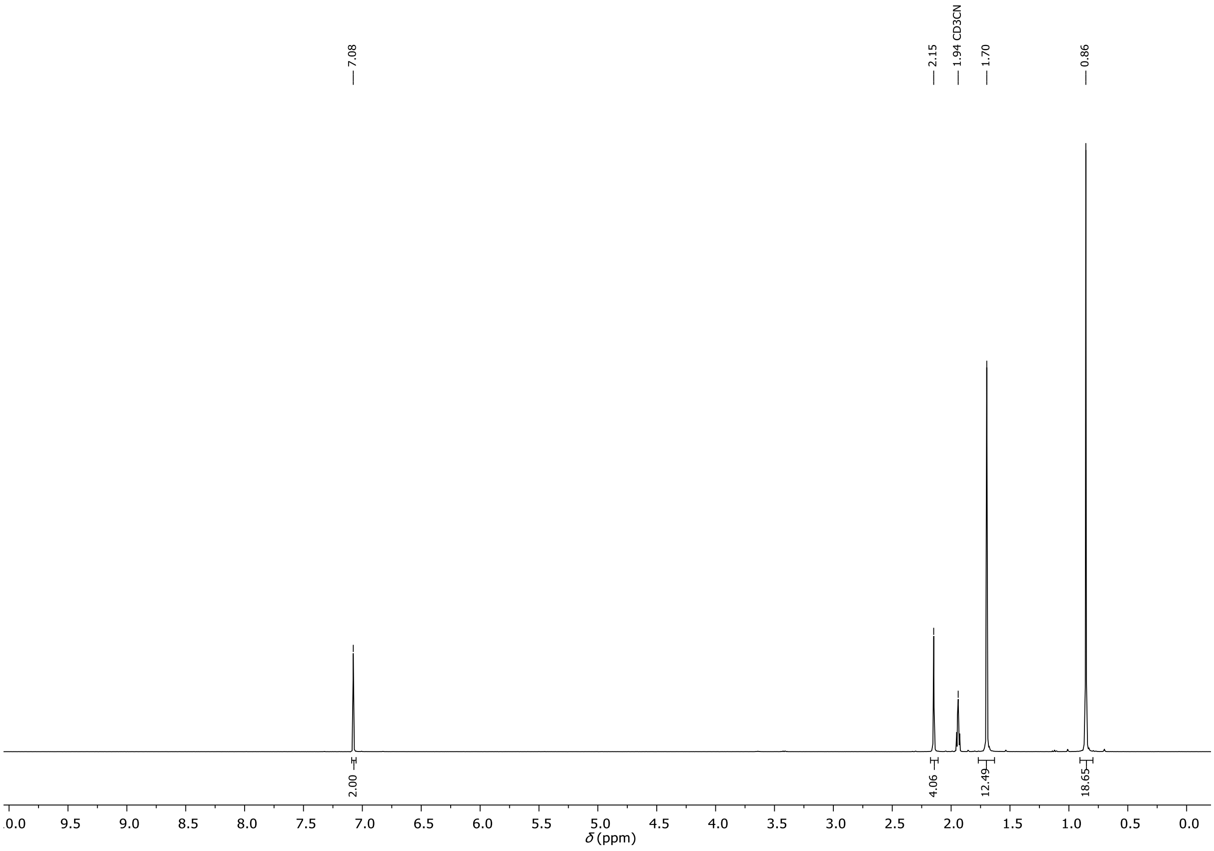

Figure S12: $^{1}H\{^{31}P\}$ NMR spectrum (400 MHz, CD_3CN) of **3**.

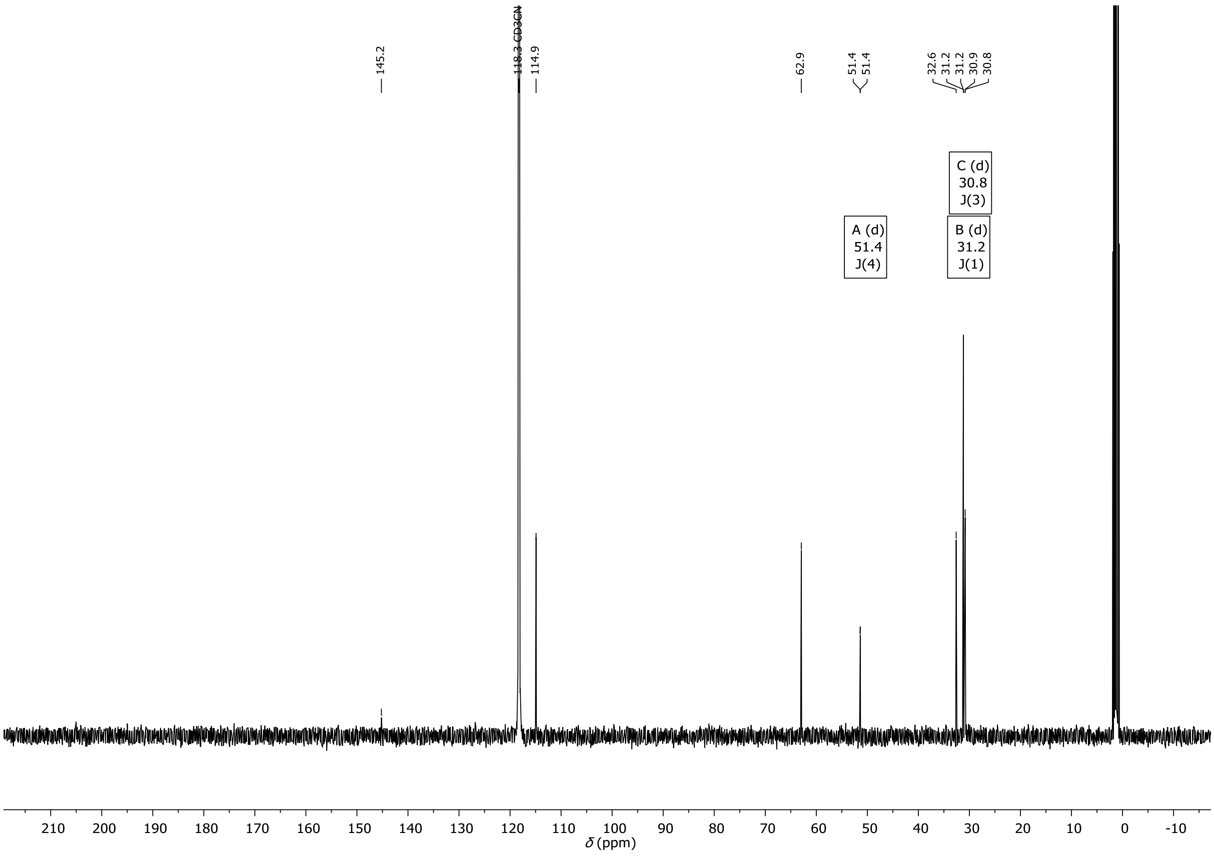

Figure S13: $^{13}C\{^{1}H\}$ NMR spectrum (101 MHz, CD_3CN) of **3**.

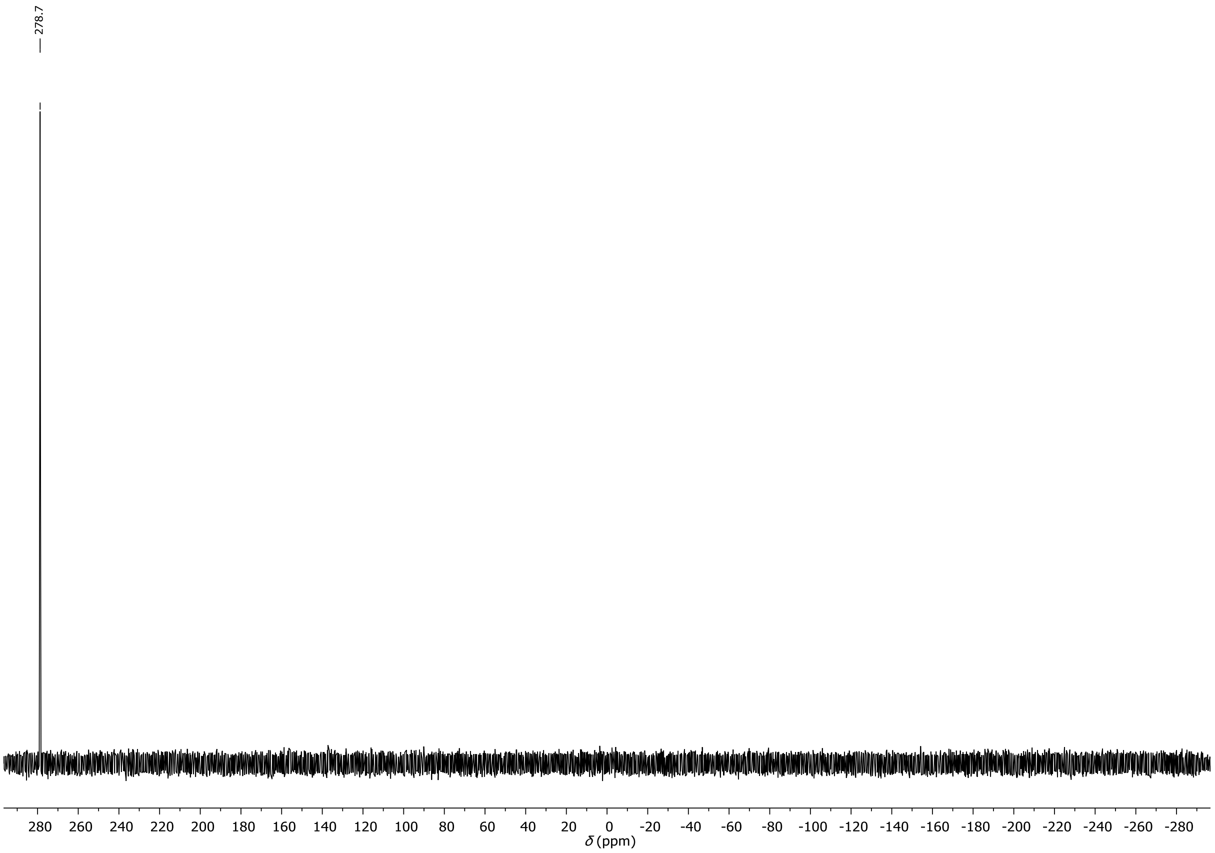

Figure S14: ^{31}P NMR spectrum (162 MHz, CD_3CN) of **3**.

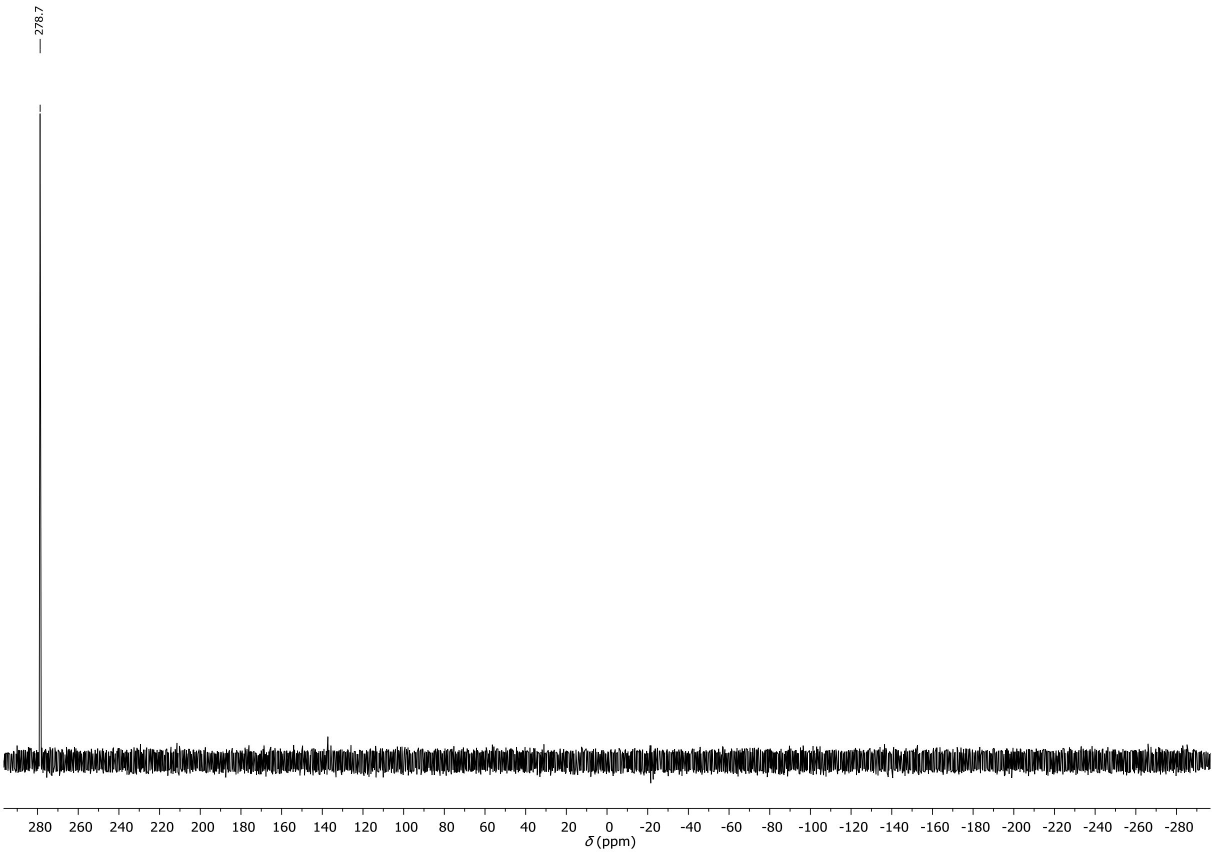

Figure S15: $^{31}P\{^1H\}$ NMR spectrum (162 MHz, CD_3CN) of **3**.

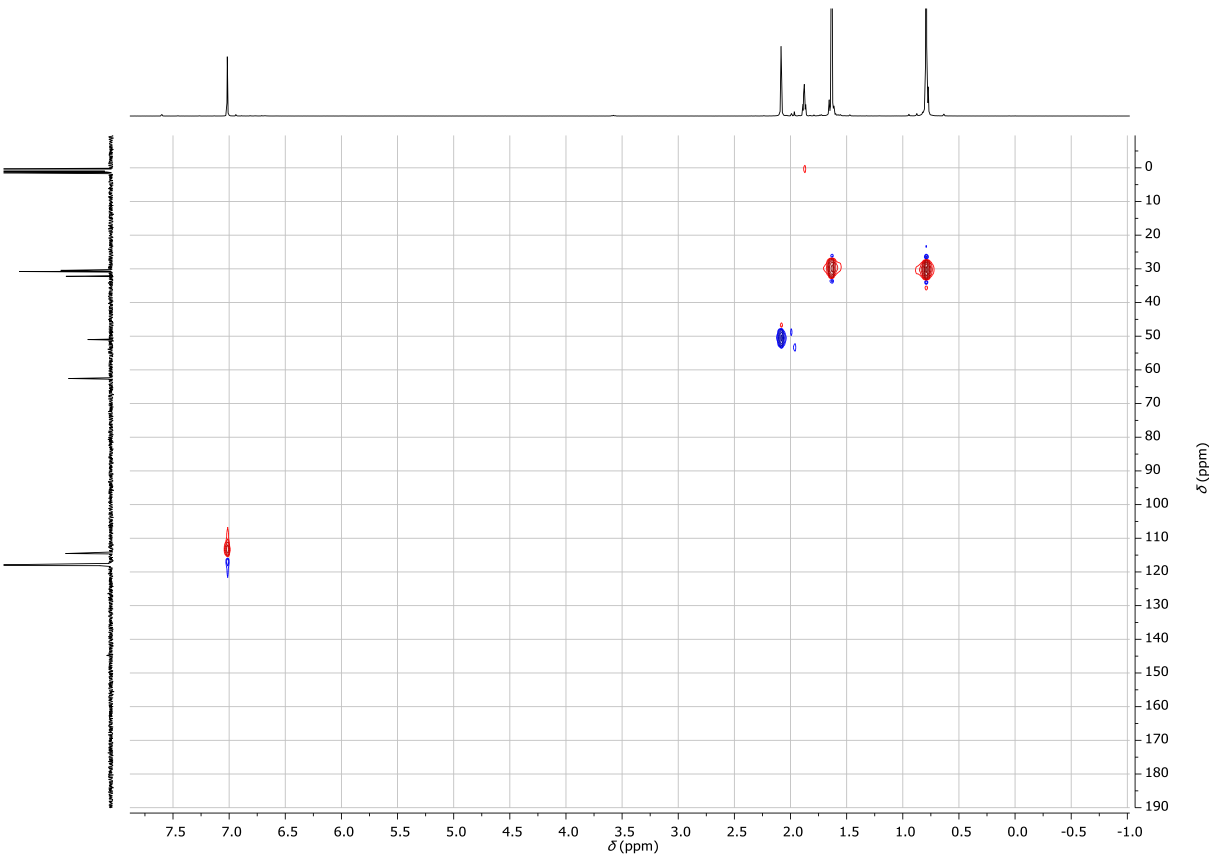

Figure S16: HSQC (^1H, ^{13}C) NMR spectrum (400 MHz, 101 MHz, CD_3CN) of **3**.

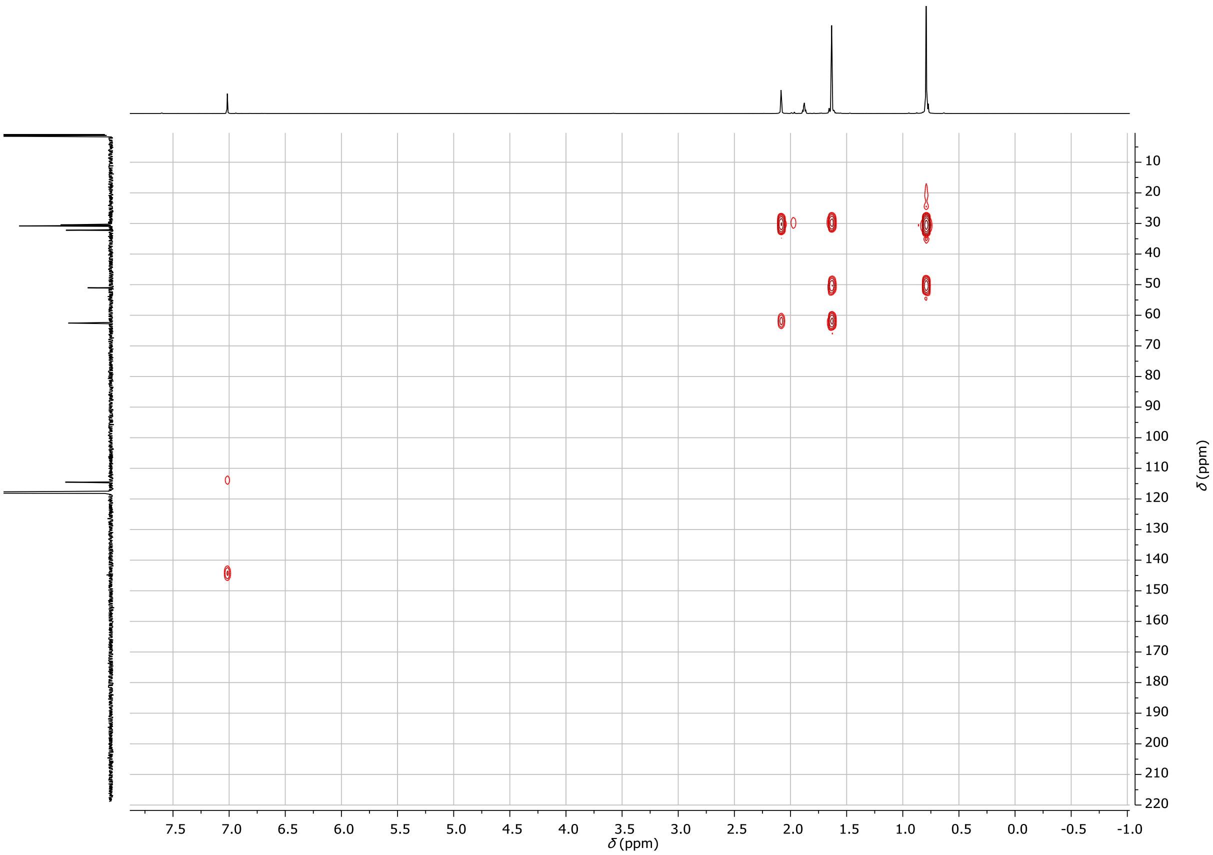

Figure S17: HMBC (^1H, ^{13}C) NMR spectrum (400 MHz, 101 MHz, CD_3CN) of **3**.

P(CCH)(NI*t*Oct)$_2$ **4**

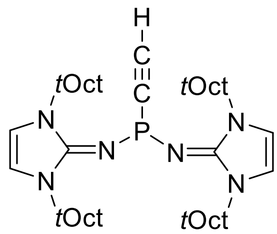

Ethynylmagnesium chloride in THF (5.9 mL, 0.5 M, 2.944 mmol, 2.00 eq) was added dropwise to a suspension of **3** (1.000 g, 1.472 mmol, 1.00 eq.) in THF (10 mL) at −78 °C. While stirring, the reaction mixture was gently warmed to room temperature overnight in the cooling bath. All volatiles were removed *in vacuo*. The residue was extracted with *n*-hexane (3x 40 mL) and the solvent was removed *in vacuo*. Phosphine **4** was obtained as white solid (652 mg, 0.976 mmol, 66%).

^{1}H NMR (400 MHz, C_6D_6) δ (ppm) = 6.17 (s, 4H, CH (NCHCHN)), 2.80 (d, $^3J_{HP}$ = 3.1 Hz, 1H, CH(CCH)), 2.54 (dd, $^4J_{HH}$ = 14.9 Hz, $^3J_{HP}$ = 2.5 Hz, 4H, CH_2), 2.38 (dd, $^4J_{HH}$ = 14.9 Hz, $^3J_{HP}$ = 2.8 Hz, 4H, CH_2), 1.67 (d, $^5J_{HH}$ = 17.4 Hz, 24H, $C(CH_3)_2$), 1.09 (s, 36H, $C(CH_3)_3$).

^{1}H{^{31}P} NMR (400 MHz, C_6D_6) δ (ppm) = 6.17 (s, 4H, CH (NCHCHN)), 2.79 (s, 1H, CH(CCH)), 2.53 (d, $^4J_{HH}$ = 14.9 Hz, 4H, CH_2), 2.38 (d, $^4J_{HH}$ = 14.9 Hz, 4H, CH_2), 1.67 (d, $^5J_{HH}$ = 17.4 Hz, 24H, $C(CH_3)_2$), 1.09 (s, 36H, $C(CH_3)_3$).

^{13}C{^{1}H} NMR (101 MHz, C_6D_6) δ (ppm) = 144.3 (d, $^2J_{CP}$ = 29 Hz, NCN), 109.2 (s, CH), 96.4 (d, $^1J_{CP}$ = 74 Hz, αC (P-CC)), 86.1 (d, $^2J_{CP}$ = 13 Hz, βC (H-CC)), 59.2 (s, q$C(CH_3)_2$), 50.3 (d, $^5J_{CP}$ = 10 Hz, CH_2), 32.2 (s, q$C(CH_3)_3$), 31.7 (d, $^7J_{CP}$ = 2 Hz, $(CH_3)_3$), 31.1 (dd, $^5J_{CP}$ = 5 Hz, $^5J_{CP}$ = 4 Hz, $(CH_3)_2$).

^{31}P NMR (162 MHz, C_6D_6) δ (ppm) = 63.0 (s).

^{31}P{^{1}H} NMR (162 MHz, C_6D_6) δ (ppm) = 63.0 (s).

IR (neat): ν_{max}/cm^{-1} = 3298.37 (*H-C*≡C), 3009.53, 2948.36, 2900.70, 2867.40, 1606.87, 1571.78, 1543.11, 1482.70, 1466.23, 1408.08, 1394.75, 1361.50, 1324.27, 1305.45, 1243.09, 1219.88, 1185.57, 1123.77, 1030.36, 1006.48, 980.94, 949.51, 925.17, 903.51, 867.66, 820.12, 727.54, 675.77, 633.63, 606.63, 577.10, 560.14, 533.44, 519.87, 469.78, 419.55.

IR (in DCM): ν_{max}/cm^{-1} = 3295.63 (*H-C*≡C), 2950.43, 2902.60, 2869.11, 1608.13, 1542.84, 1484.16, 1409.26, 1394.79, 1363.62, 1323.91, 1305.28, 1264.57, 1243.75, 1221.62, 1186.74, 1133.63, 1031.20, 980.55, 950.63, 923.88, 903.59, 867.26, 819.21, 739.91, 706.97, 644.32, 560.15, 464.64, 421.97.

HRMS (ESI, CH_3CN): m/z = 669.5683 ($[M+H]^+$, calculated: 669.5707).

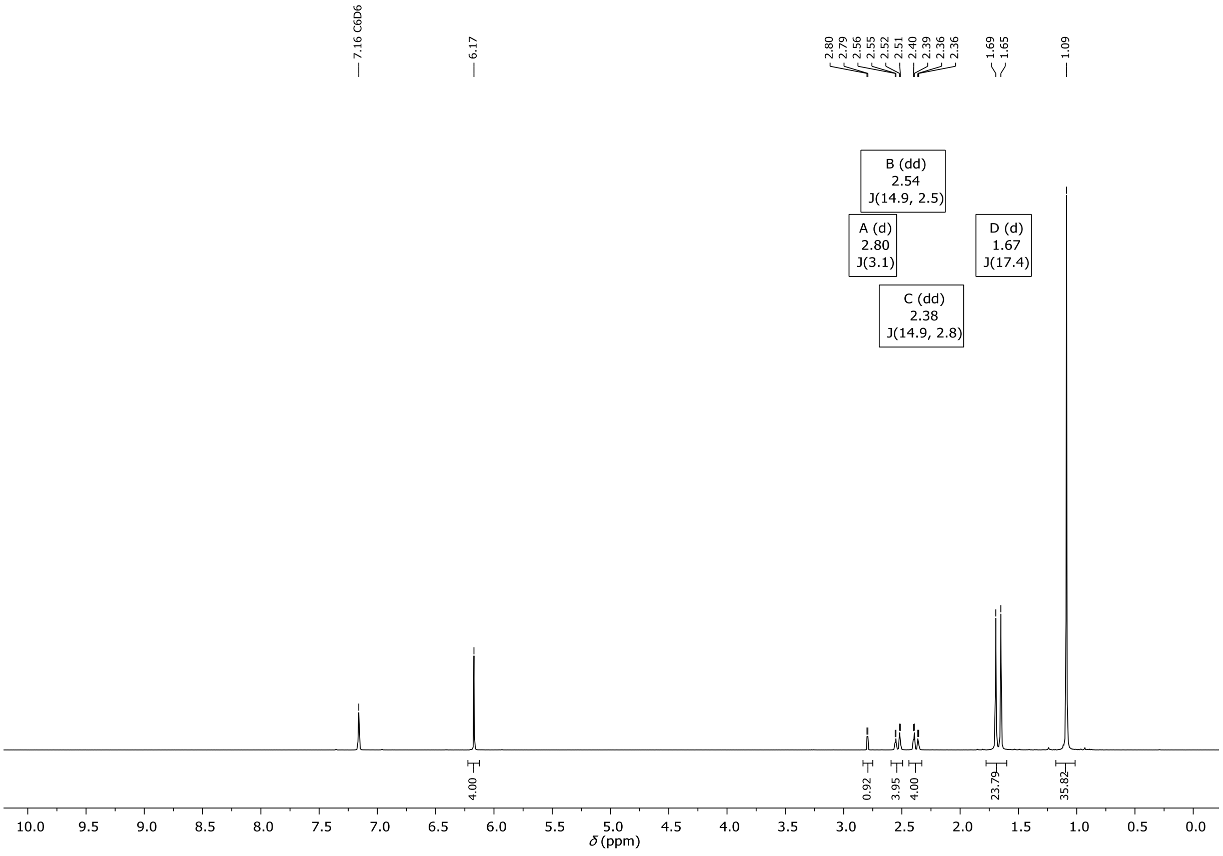

Figure S18: ^{1}H NMR spectrum (400 MHz, C_6D_6) of **4**.

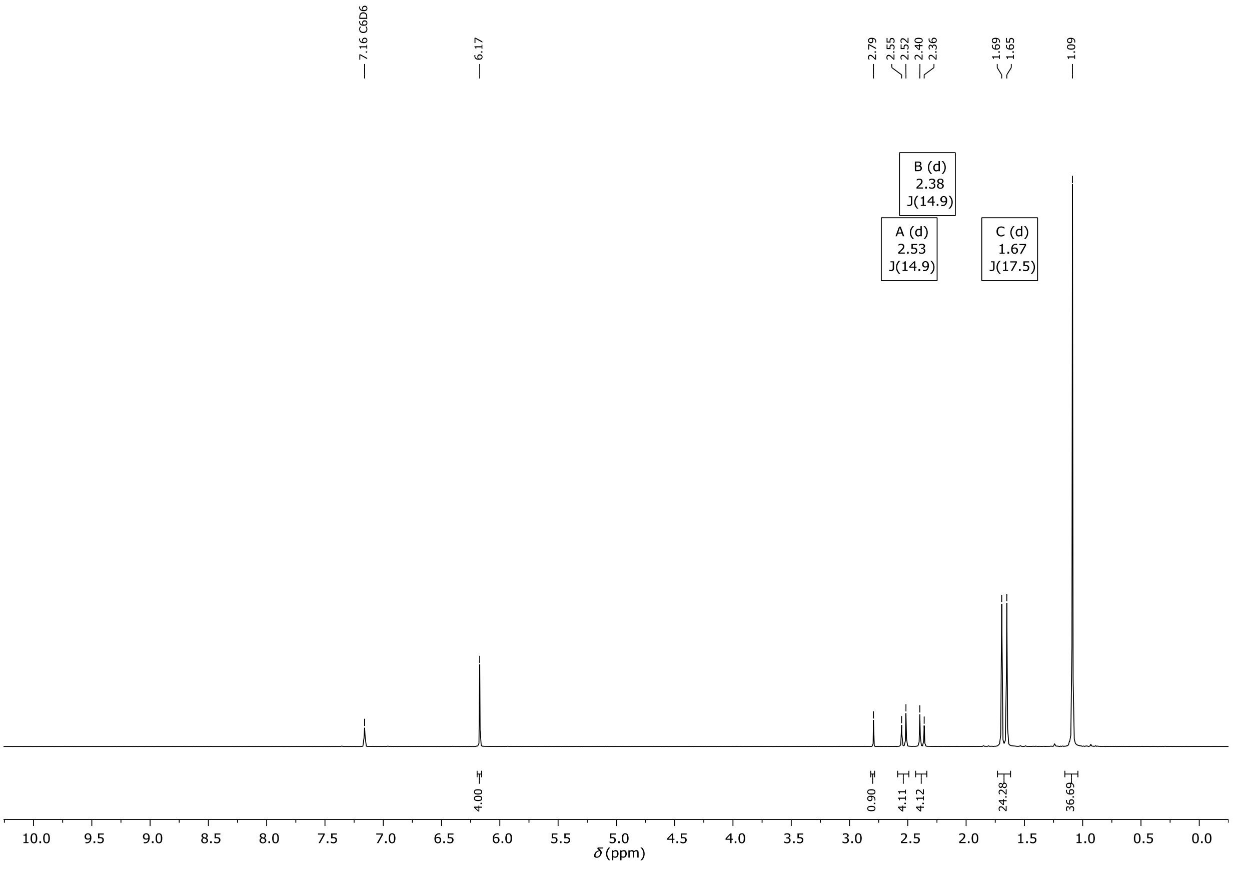

Figure S19: $^{1}H\{^{31}P\}$ NMR spectrum (400 MHz, C_6D_6) of **4**.

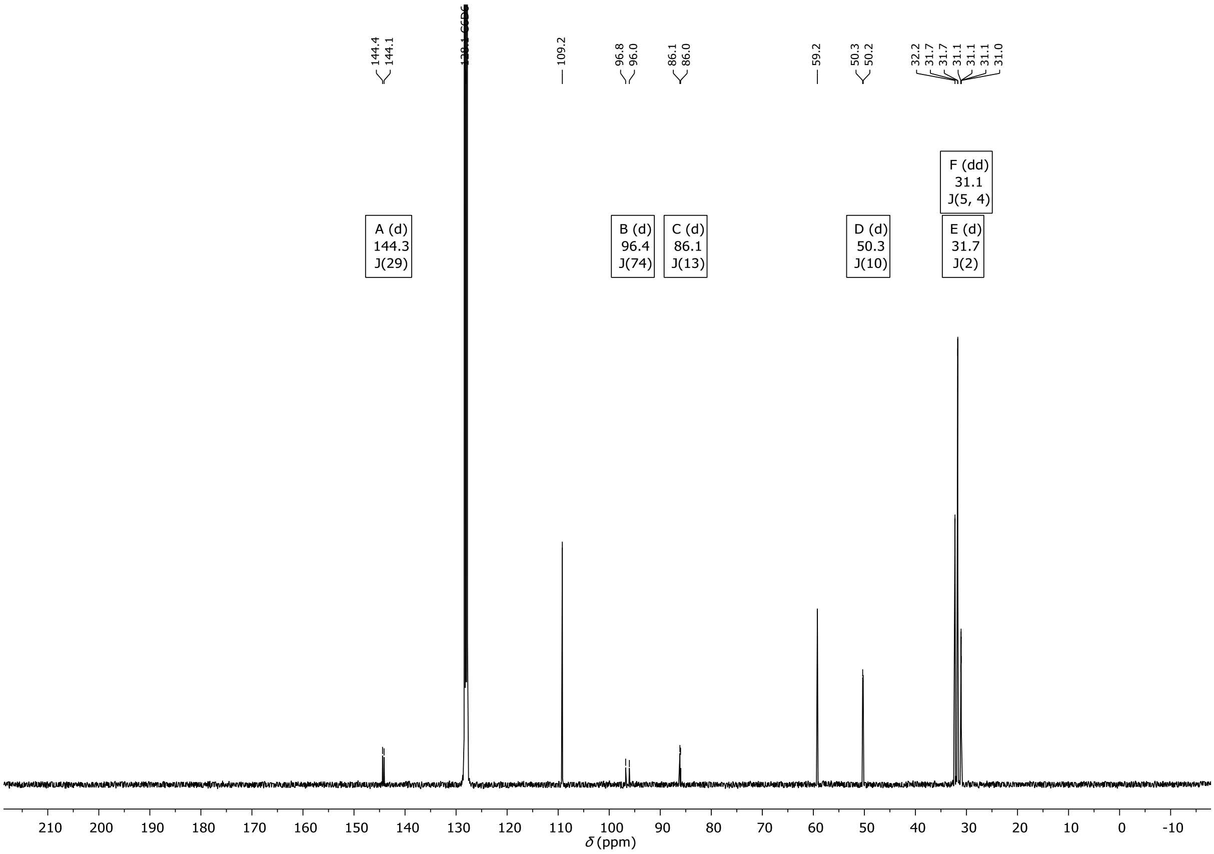

Figure S20: $^{13}C\{^1H\}$ NMR spectrum (101 MHz, C_6D_6) of **4**.

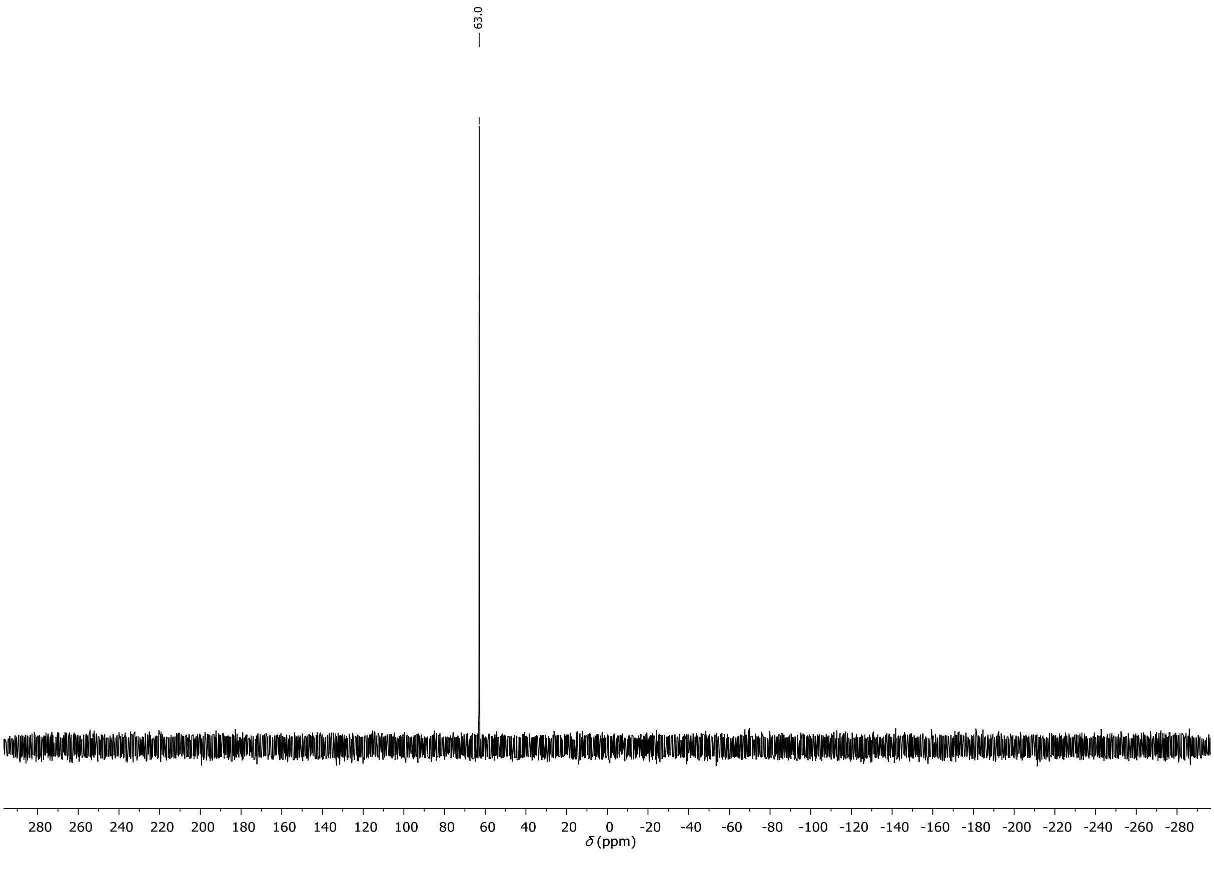

Figure S21: ^{31}P NMR spectrum (162 MHz, C_6D_6) of **4**.

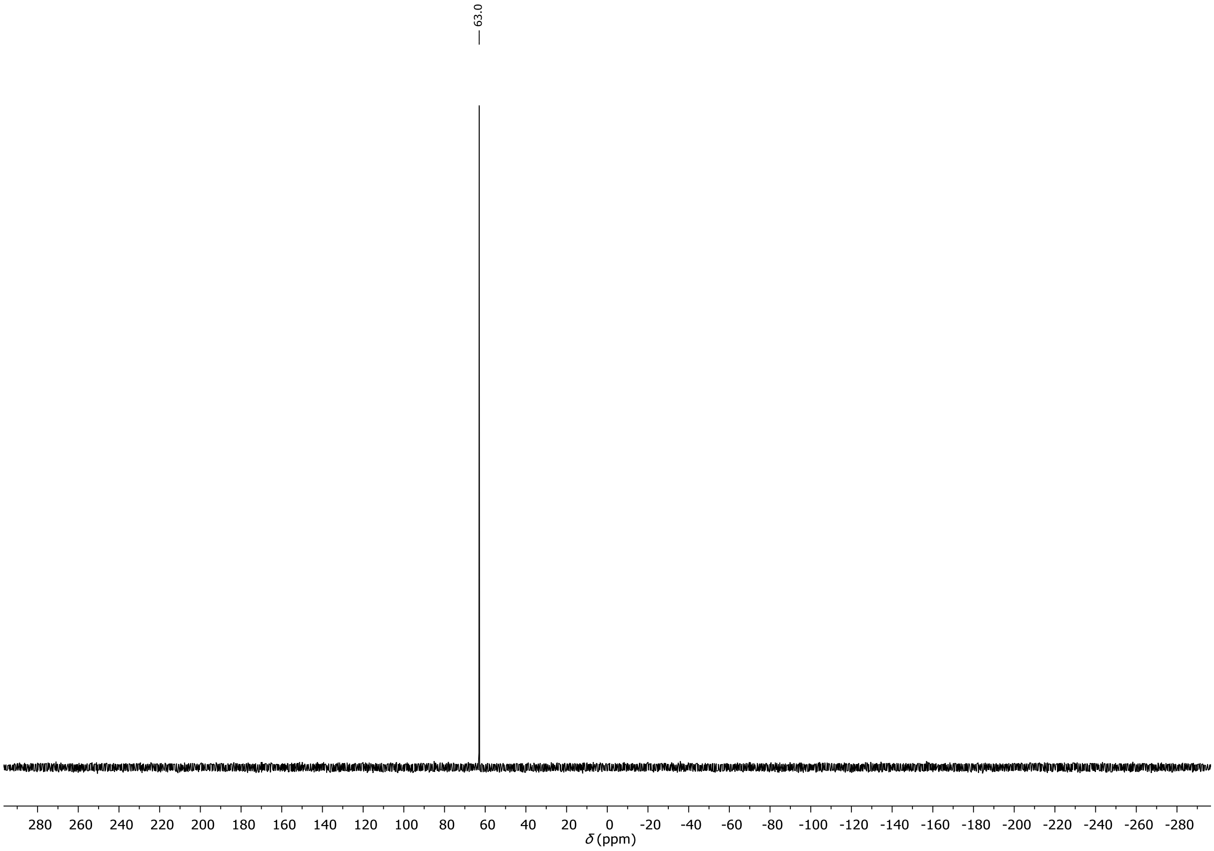

Figure S22: $^{31}P\{^{1}H\}$ NMR spectrum (162 MHz, C_6D_6) of **4**.

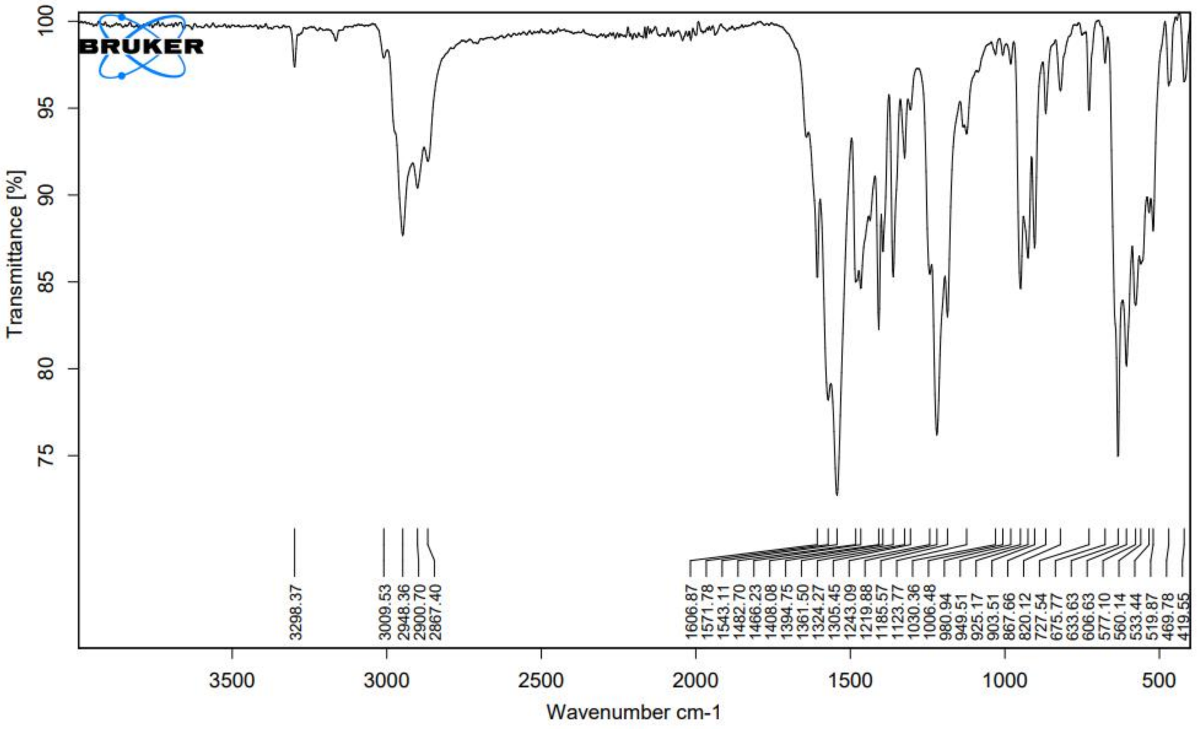

Figure S23: IR spectrum (neat) of **4**.

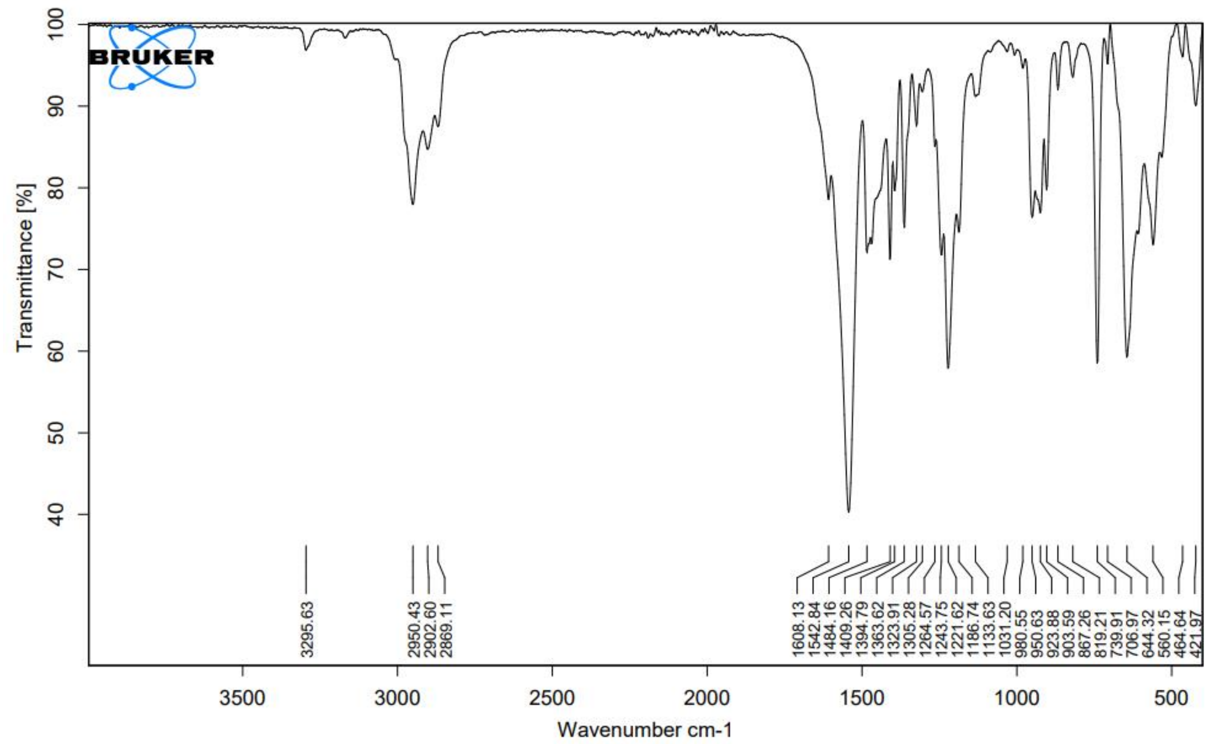

Figure S24: IR spectrum (in DCM) of **4**.

<u>[P(CCH)Me(NI*t*Oct)$_2$][I] **5**</u>

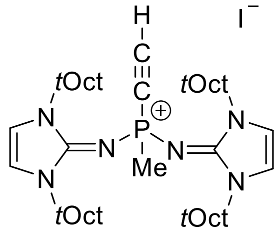

Methyl iodide in toluene (1.06 mL, 0.765 M, 0.807 mmol, 1.20 eq.) was slowly added to a solution of **4** (450 mg, 0.673 mmol, 1.00 eq.) in *n*-hexane (30 mL). The reaction mixture was stirred for overnight at room temperature. All volatiles were removed *in vacuo*. **5** was obtained as white solid (522 mg, 0.644 mmol, 96%).

^{1}**H NMR** (400 MHz, CD_3CN) δ (ppm) = 6.97 (s, 4H, CH (NCHCHN)), 3.57 (d, $^{3}J_{HP}$ = 10.3 Hz, 1H, CH (CCH)), 2.14 - 2.02 (m, 8H, CH_2), 1.90 (d, $^{2}J_{HP}$ = 13.4, 3H, PCH_3), 1.76 (d, $^{5}J_{HH}$ = 12.7 Hz, 24H, $C(CH_3)_2$), 0.89 (s, 36H, $C(CH_3)_3$).

^{1}**H{^{31}P} NMR** (400 MHz, CD_3CN) δ (ppm) = 6.97 (s, 4H, CH (NCHCHN)), 3.57 (s, 1H, CH (CCH)), 2.14 - 2.02 (m, 8H, CH_2), 1.90 (s, 3H, PCH_3), 1.76 (d, $^{5}J_{HH}$ = 12.5 Hz, 24H, $C(CH_3)_2$), 0.89 (s, 36H, $C(CH_3)_3$).

^{13}**C{^{1}H} NMR** (101 MHz, CD_3CN) δ (ppm) = 144.2 (d, $^{2}J_{CP}$ = 16 Hz, NCN), 114.1 (s, CH), 93.2 (d, $^{2}J_{CP}$ = 29 Hz, βC (H-CC)), 83.5 (d, $^{1}J_{CP}$ = 171 Hz, αC (P-CC)), 63.2 (s, $qC(CH_3)_2$), 51.8 (s, CH_2), 32.4 (s, $qC(CH_3)_3$), 31.3 (s, $(CH_3)_3$), 30.9 (d, $^{5}J_{CP}$ = 7 Hz, $(CH_3)_2$), 22.5 (d, $^{1}J_{CP}$ = 114 Hz, PCH_3).

^{31}**P NMR** (162 MHz, CD_3CN) δ (ppm) = −34.5 (qd, $^{2}J_{PH}$ = 13 Hz, $^{3}J_{PH}$ = 10 Hz).

^{31}**P{^{1}H} NMR** (162 MHz, CD_3CN) δ (ppm) = −34.5 (s).

IR (neat): ν_{max}/cm^{-1} = 3093.47 (*H-C*≡C), 2948.57, 2902.12, 2868.51, 2032.11 (C≡C), 1520.63, 1484.66, 1418.55, 1394.75, 1366.30, 1324.90, 1277.70, 1231.89, 1214.15, 1186.19, 1121.99, 1040.73, 999.25, 959.76, 907.76, 875.83, 743.43, 692.86, 634.48, 588.12, 467.16, 431.67.

IR (in DCM): ν_{max}/cm^{-1} = 3096.89 (*H-C*≡C), 2952.94, 2905.48, 2870.79, 2034.53 (C≡C), 1523.55, 1485.81, 1419.38, 1395.49, 1367.43, 1325.32, 1272.46, 1232.02, 1215.06, 1186.79, 1122.80, 1041.36, 999.61, 960.48, 907.81, 876.37, 823.15, 742.91, 693.38, 546.92, 467.64.

HRMS (ESI, CH_3CN): m/z = 683.5840 ($[M-I]^+$, calculated: 683.5864).

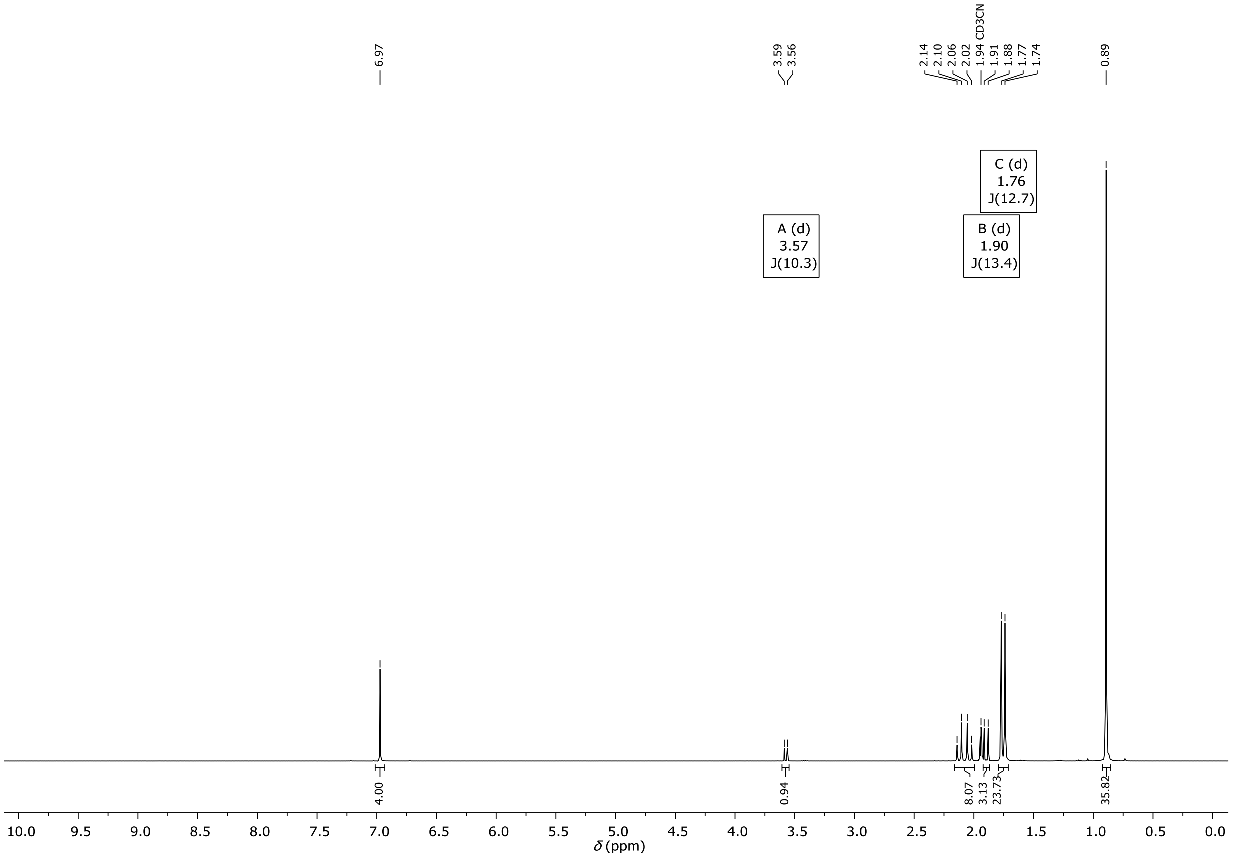

Figure S25: ^{1}H NMR spectrum (400 MHz, CD_3CN) of **5**.

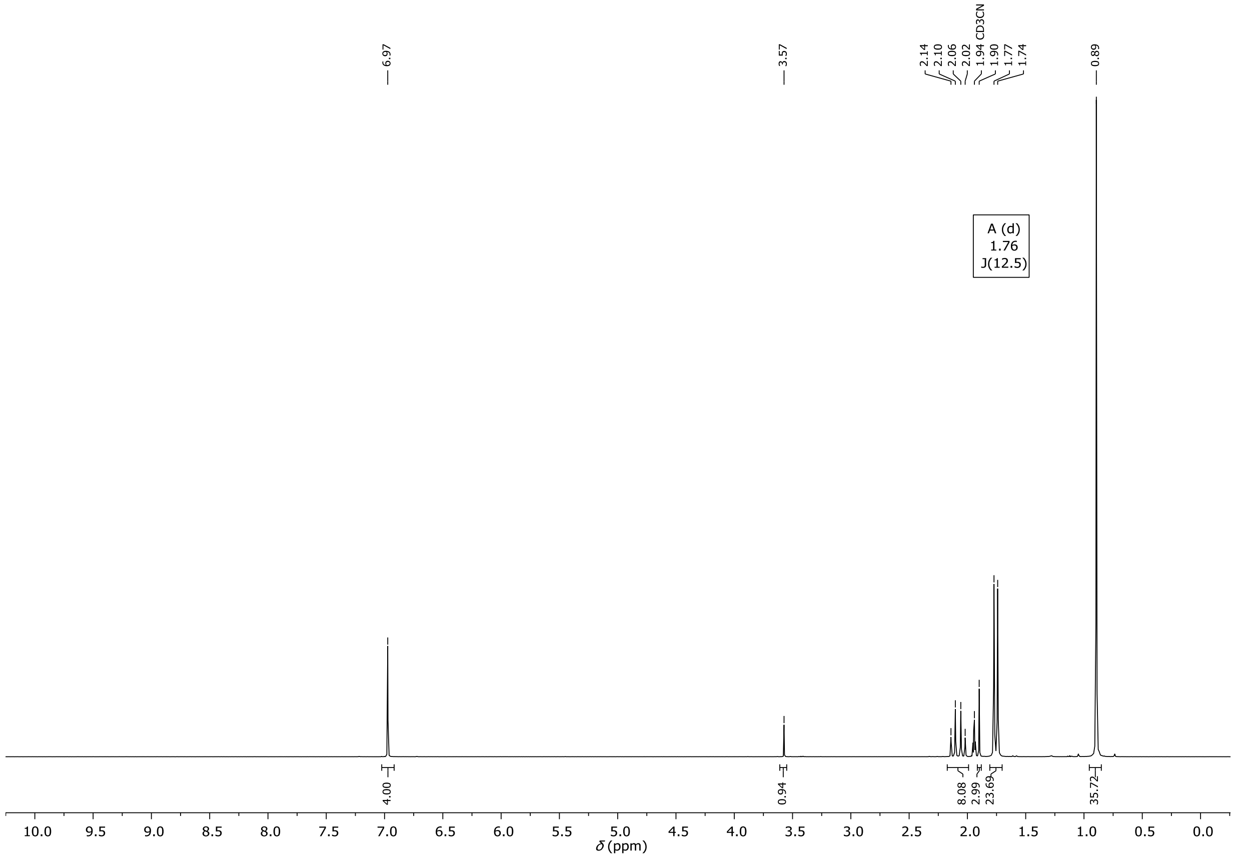

Figure S26: $^{1}H\{^{31}P\}$ NMR spectrum (400 MHz, CD_3CN) of **5**.

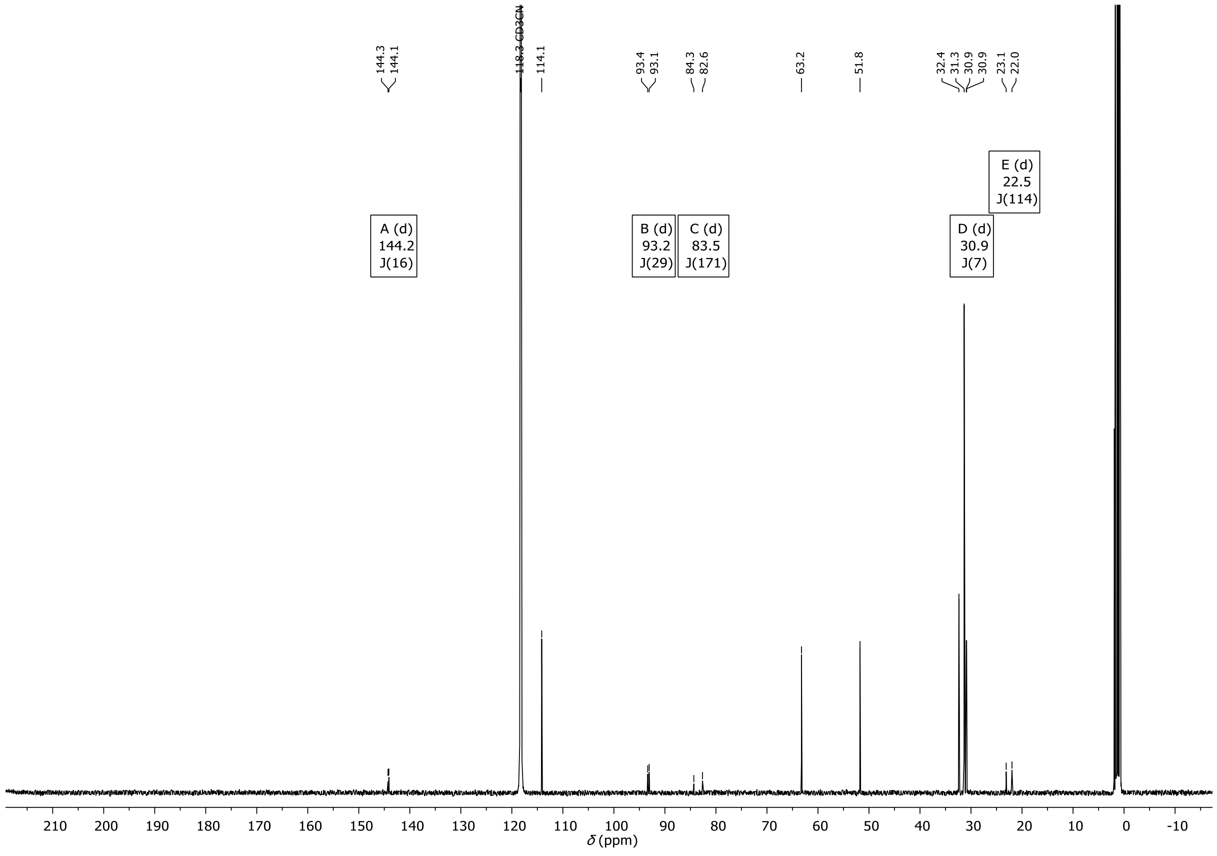

Figure S27: $^{13}C\{^{1}H\}$ NMR spectrum (101 MHz, CD_3CN) of **5**.

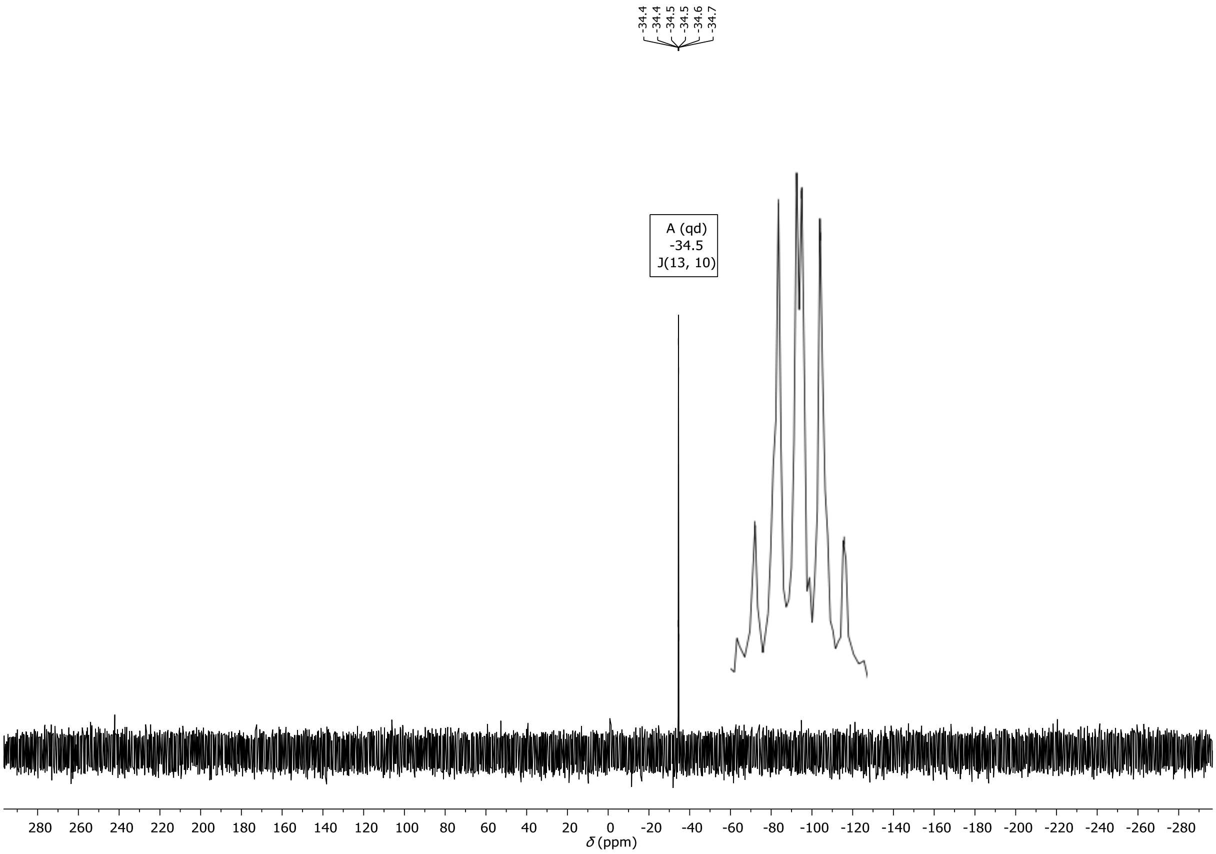

Figure S28: ^{31}P NMR spectrum (162 MHz, CD_3CN) of **5**.

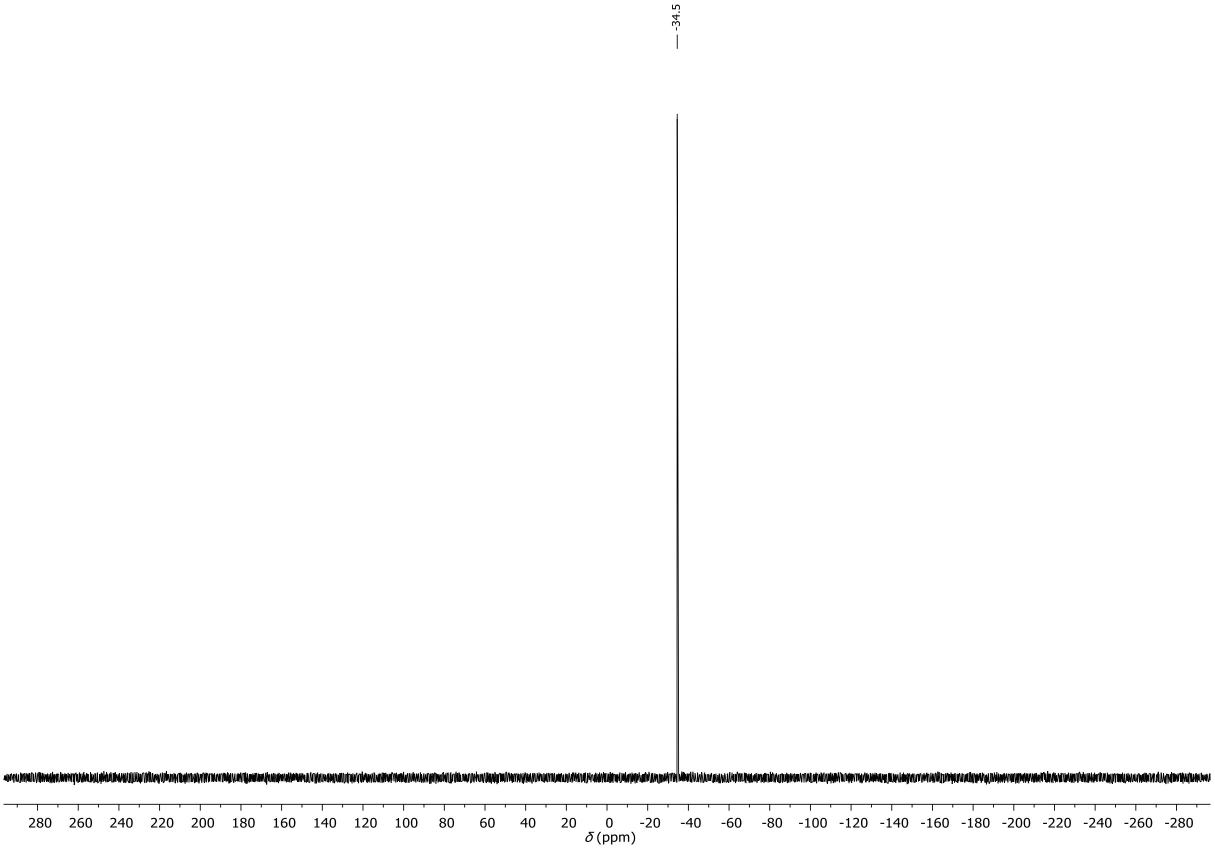

Figure S29: $^{31}P\{^1H\}$ NMR spectrum (162 MHz, CD_3CN) of **5**.

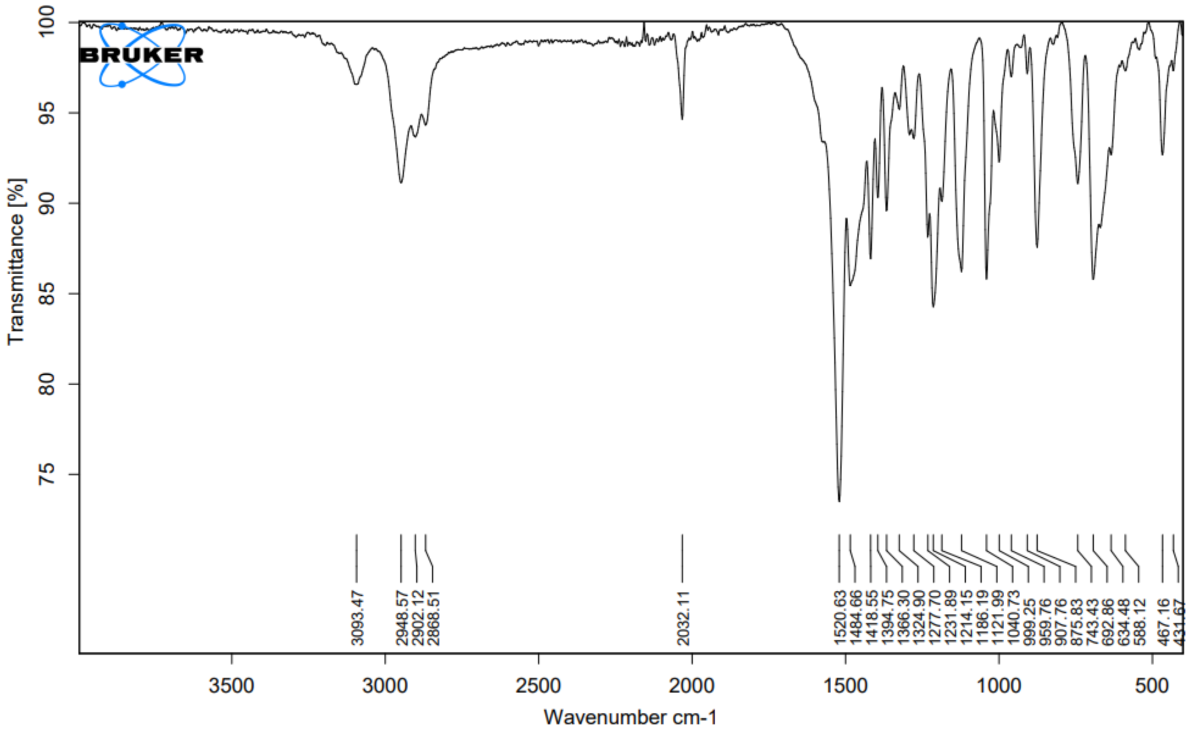

Figure S30: IR spectrum (neat) of **5**.

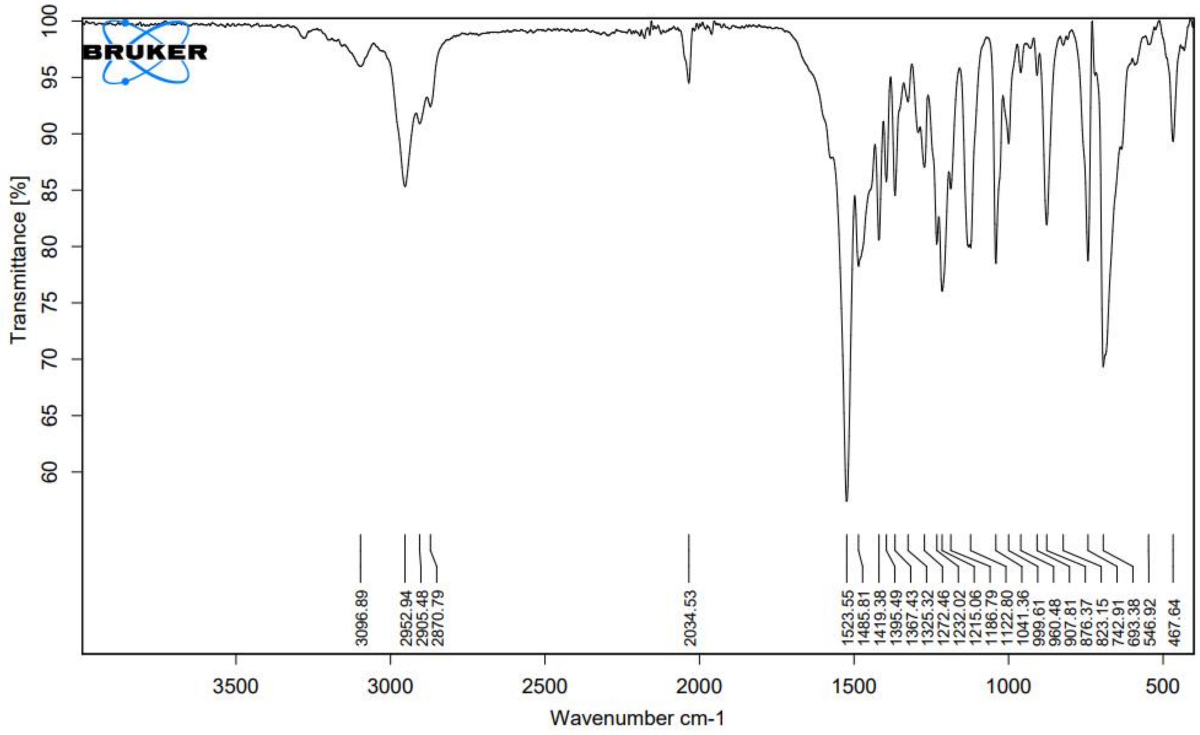

Figure S31: IR spectrum (in DCM) of **5**.

P(CC)Me(NI*t*Oct)$_2$ **6**

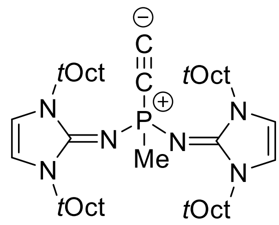

A solution of KHMDS (26 mg, 0.130 mmol, 1.06 eq.) in THF (1 mL) cooled to −40 °C was quickly added to a stirred suspension of **5** (100 mg, 0.123 mmol, 1.00 eq.) in THF (2 mL) at −40 °C. Note that the base needs to be added quickly and the reaction temperature must be -40 °C or lower to avoid side reactions. Immediately after the addition, all volatiles were removed *in vacuo* while keeping the mixture at −40 °C. The residue was washed with cold diethyl ether (2x 1 mL, −40 °C) to remove excess KHMDS but avoid dissolving of the product. The residue was extracted at ambient temperature with toluene (3 x 2 mL) to remove formed potassium iodide. After filtration, the solvent was removed *in vacuo*. Phosphonioacetylide **6** was obtained as a brown solid (65 mg, 0.095 mmol, 77%).

^{1}H NMR (400 MHz, C_6D_6) δ (ppm) = 6.19 (s, 4H, CH (NCHCHN)), 2.29 (s, 8H, CH_2), 1.91 (s, 3H, PCH_3), 1.79 (s, 12H, $C(CH_3)_2$), 1.63 (s, 12H, $C(CH_3)_2$), 1.06 (s, 36H, $C(CH_3)_3$).

^{1}H{^{31}P} NMR (400 MHz, C_6D_6) δ (ppm) = 6.19 (s, 4H, CH (NCHCHN)), 2.29 (s, 8H, CH_2), 1.91 (d, $^2J_{HP}$ = 13.0 Hz, 3H, PCH_3), 1.79 (s, 12H, $C(CH_3)_2$), 1.62 (s, 12H, $C(CH_3)_2$), 1.05 (s, 36H, $C(CH_3)_3$).

^{13}C{^{1}H} NMR (176 MHz, C_6D_6) δ (ppm) = 216.4 (d, $^2J_{CP}$ = 16 Hz, βC (CC)), 145.3 (d, $^2J_{CP}$ = 15 Hz, NCN), 111.2 (s, CH), 105.5 (d, $^1J_{CP}$ = 143 Hz, αC (P-CC)), 61.4 (s, q$C(CH_3)_2$), 51.0 (s, CH_2), 32.1 (s, q$C(CH_3)_3$), 31.5 (s, $(CH_3)_3$), 31.0 (d, $^5J_{CP}$ = 45 Hz, $(CH_3)_2$), 23.9 (d, $^1J_{CP}$ = 108 Hz, PCH_3).

^{31}P NMR (162 MHz, C_6D_6): δ (ppm) = −37.4 (q, $^2J_{CH}$ = 13 Hz).

^{31}P{^{1}H} NMR (162 MHz, C_6D_6): δ (ppm) = −37.4 (s).

HRMS (ESI, THF) m/z = 683.5856 ([M+H]+, calculated: 683.5864).

IR (neat): ν_{max}/cm^{-1} = 2948.23, 2902.16, 2870.00, 1947.18 (C≡C), 1524.01, 1479.84, 1416.89, 1393.42, 1365.56, 1325.55, 1287.10, 1219.46, 1184.68, 1133.09, 1112.33, 1013.71, 985.47, 955.42, 907.93, 875.11, 746.61, 697.42, 490.83, 472.93, 431.98.

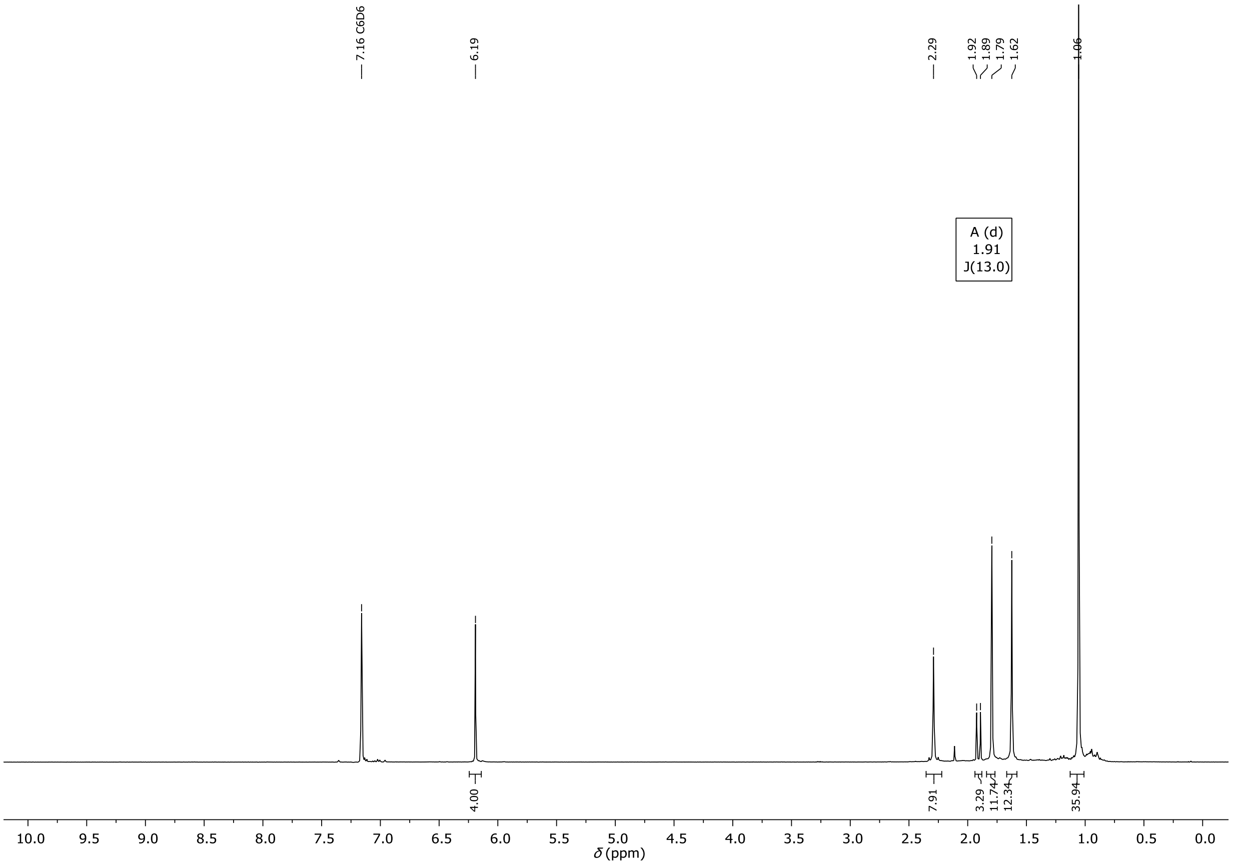

Figure S32: ^{1}H NMR spectrum (400 MHz, C_6D_6) of **6**.

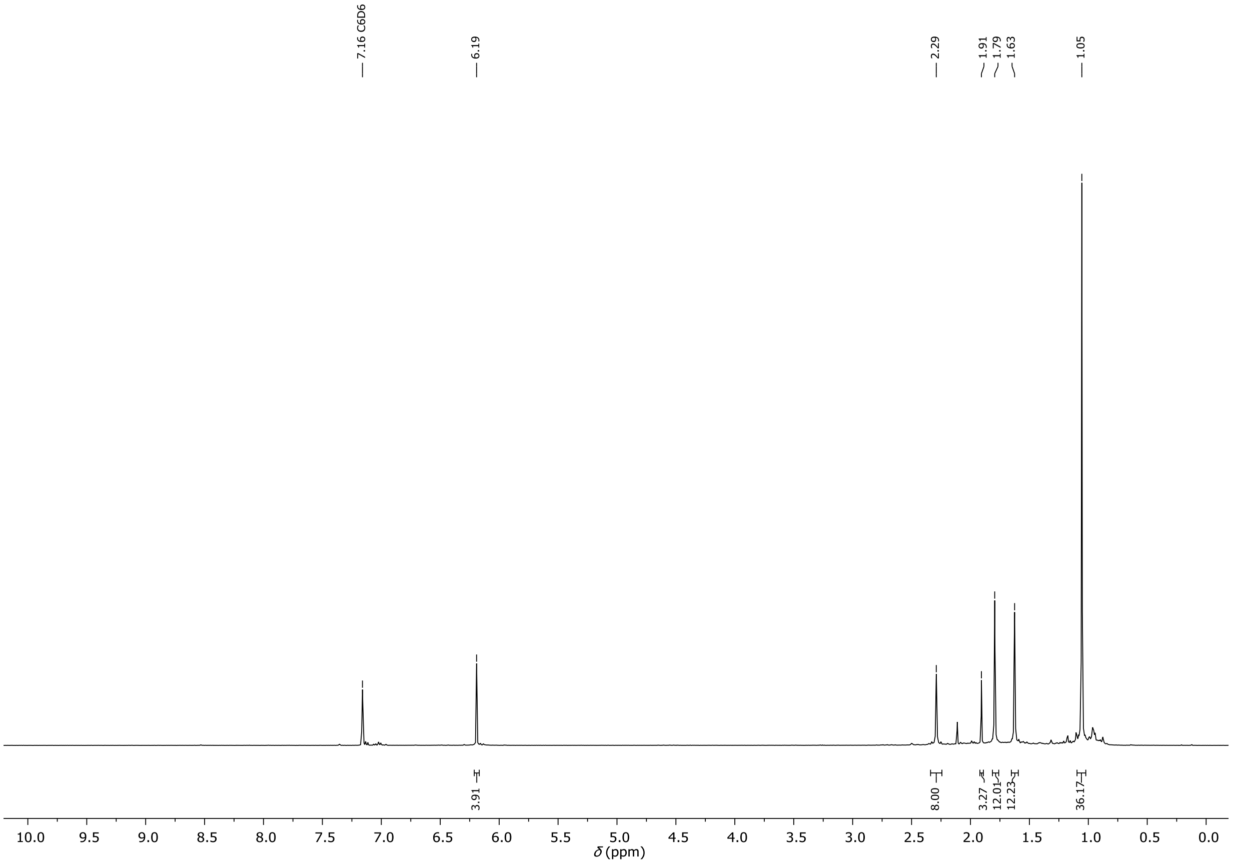

Figure S33: $^{1}H\{^{31}P\}$ NMR spectrum (400 MHz, C_6D_6) of **6**.

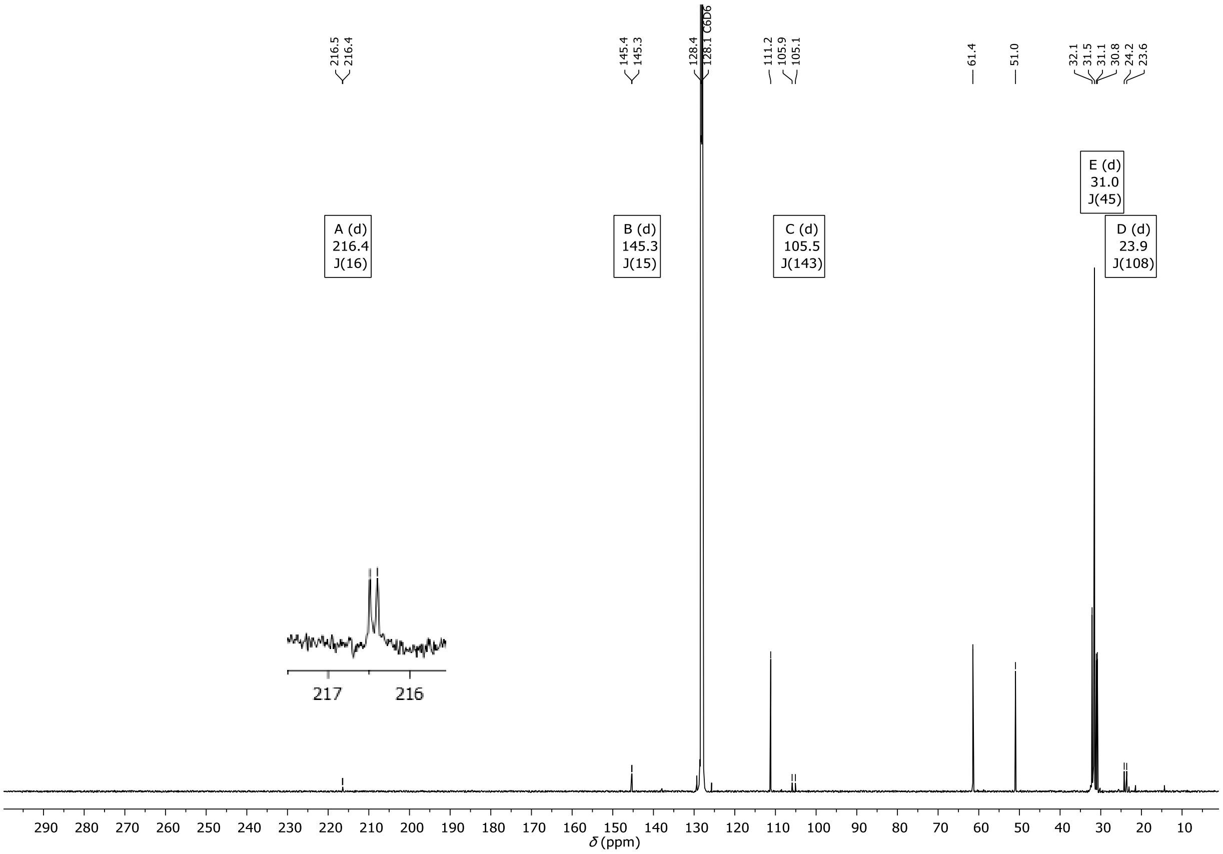

Figure S34: $^{13}C\{^{1}H\}$ NMR spectrum (176 MHz, C_6D_6) of **6**. Signal at δ = 128.4 ppm corresponds to C_6D_5H.

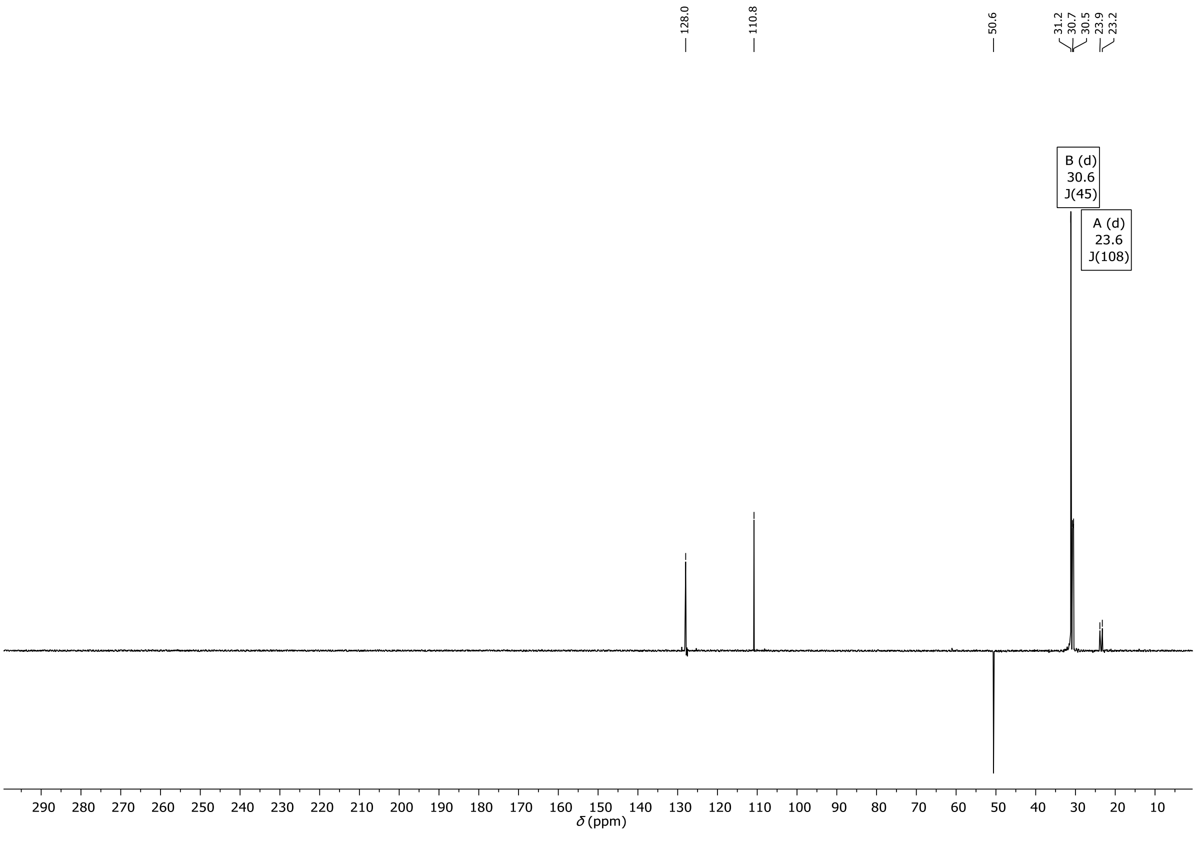

Figure S35: ^{13}C DEPT-135 spectrum (176 MHz, C_6D_6) of **6**. Signal at δ = 128.0 ppm corresponds to C_6D_5H.

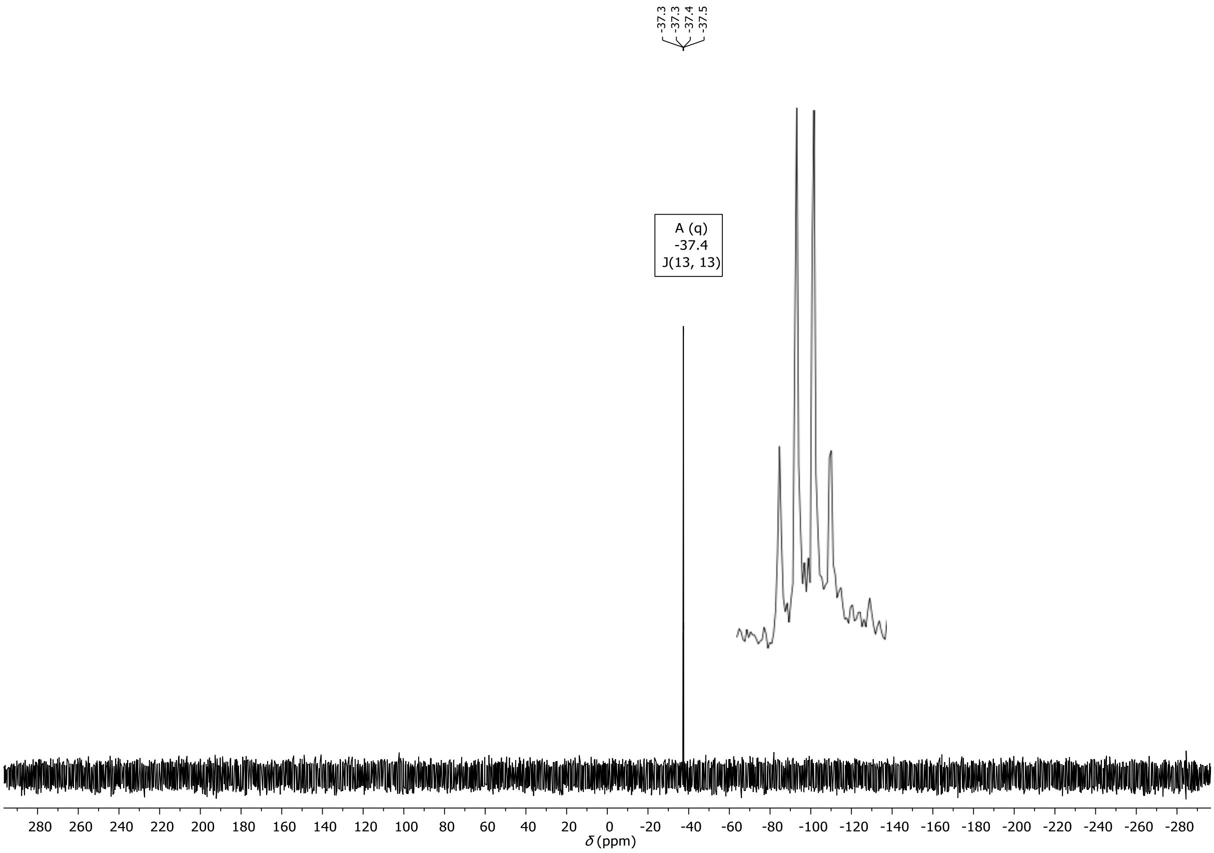

Figure S36: ^{31}P NMR spectrum (162 MHz, C_6D_6) of **6**.

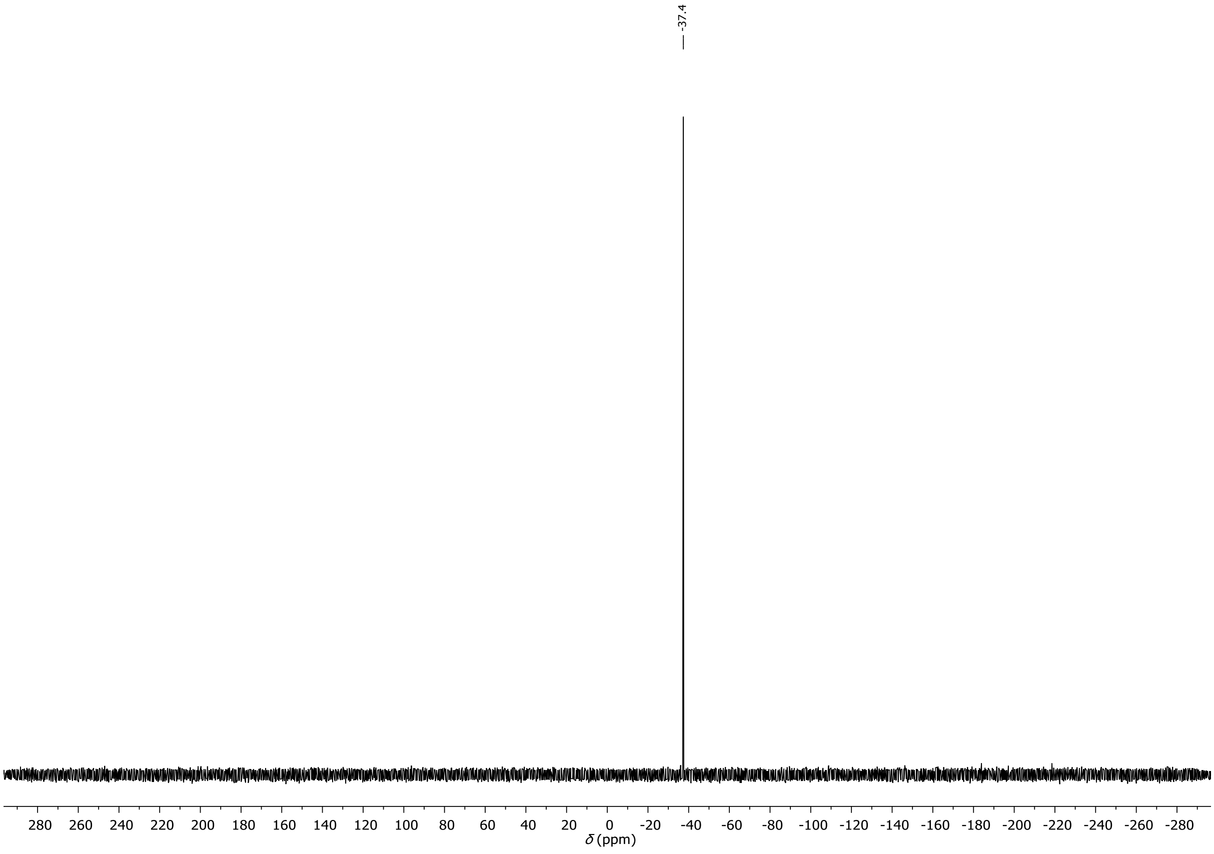

Figure S37: $^{31}P\{^1H\}$ NMR spectrum(162 MHz, C_6D_6) of **6**.

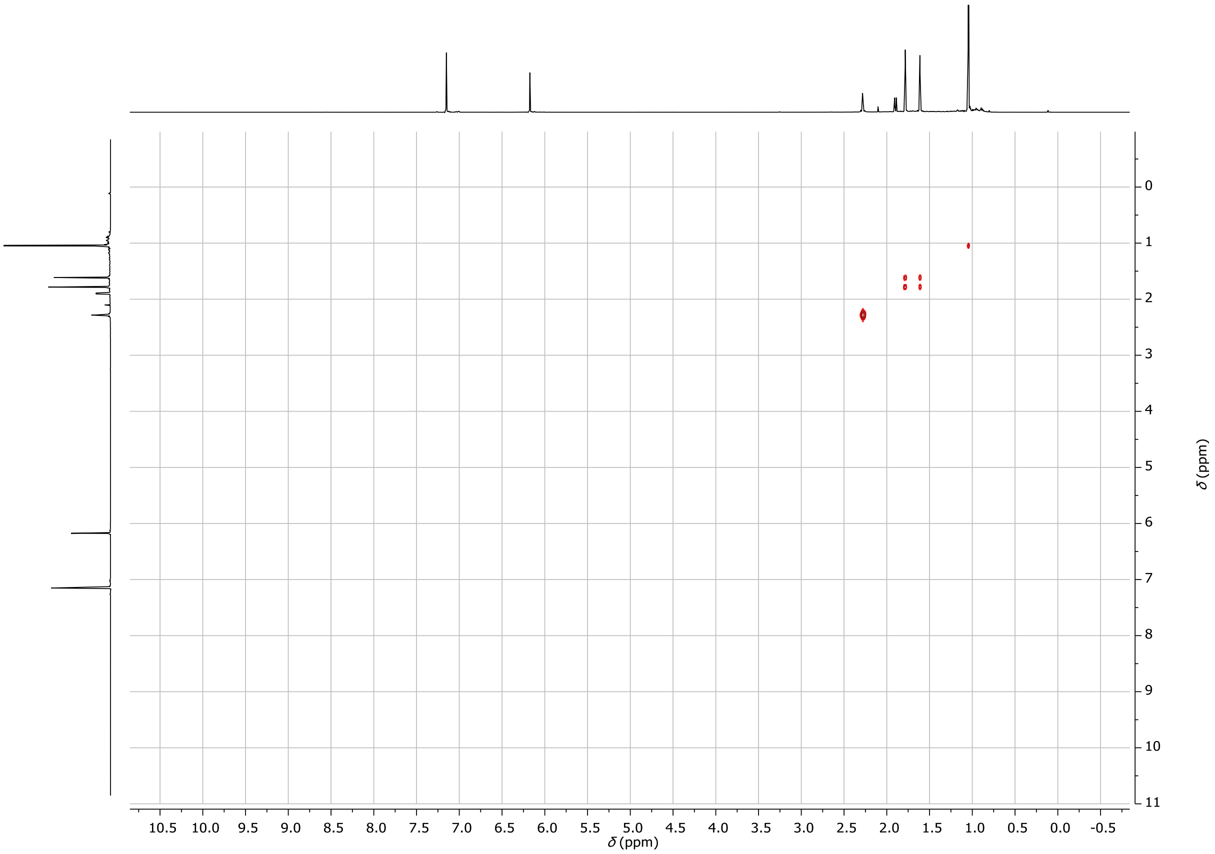

Figure S38: COSY (^{1}H, ^{1}H) NMR spectrum (700 MHz, 700 MHz, C_6D_6) of **6**.

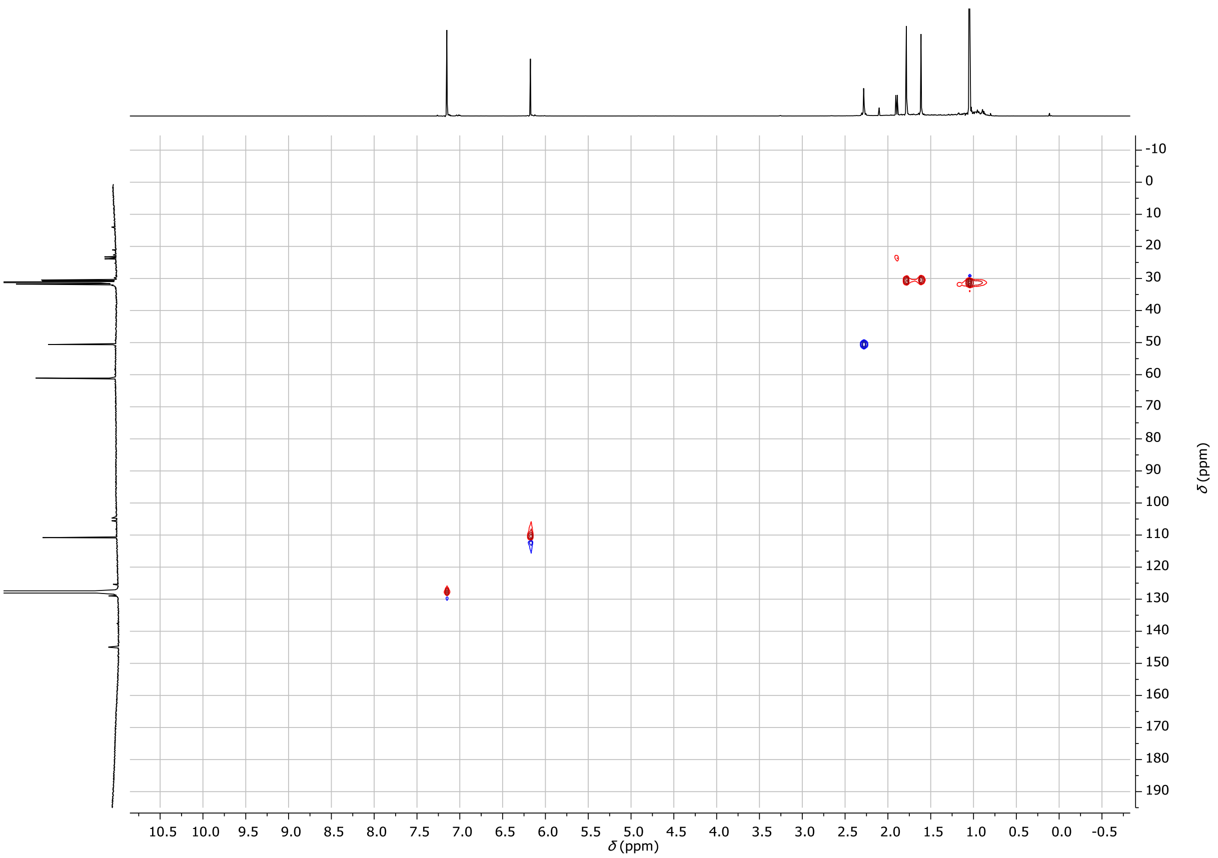

Figure S39: HSQC (^1H, ^{13}C) NMR spectrum (700 MHz, 176 MHz, C_6D_6) of **6**.

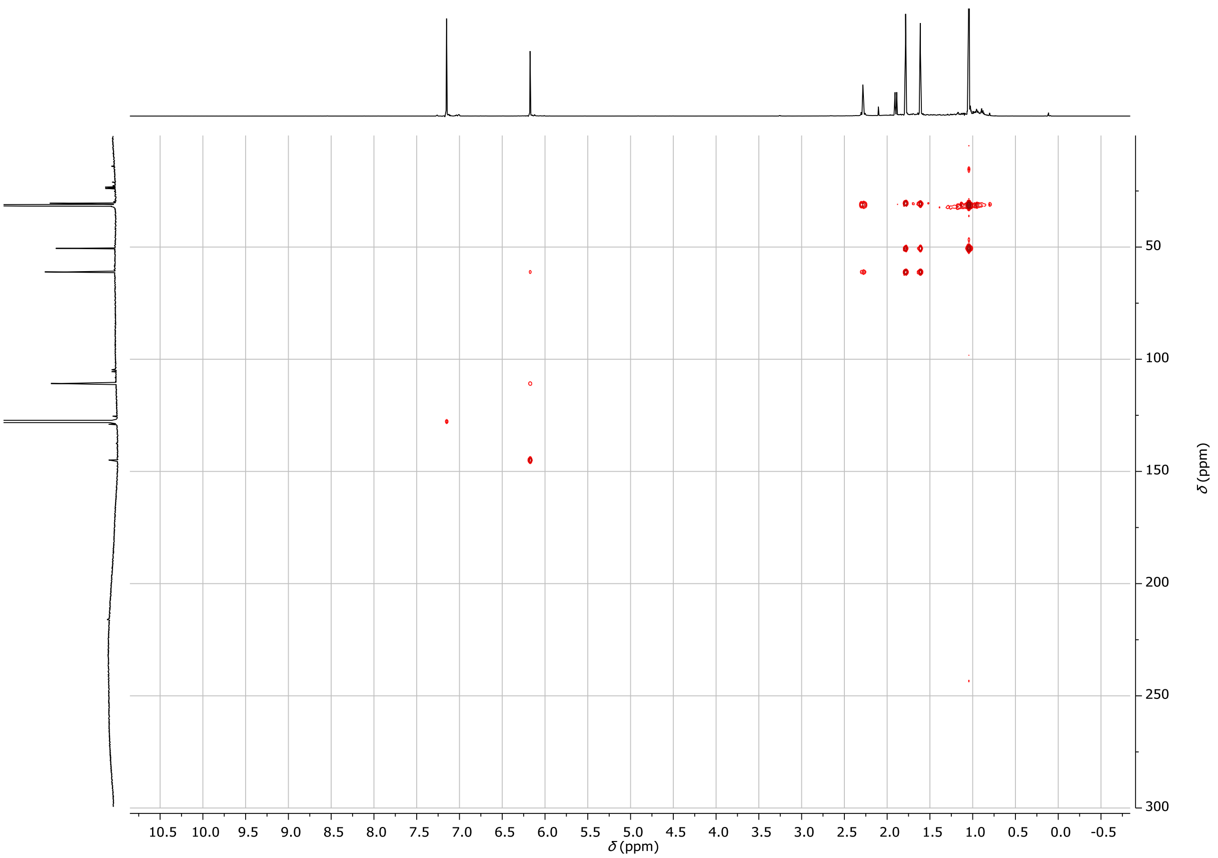

Figure S40: HMBC (^1H, ^{13}C) NMR spectrum (700 MHz, 176 MHz, C_6D_6) of **6**.

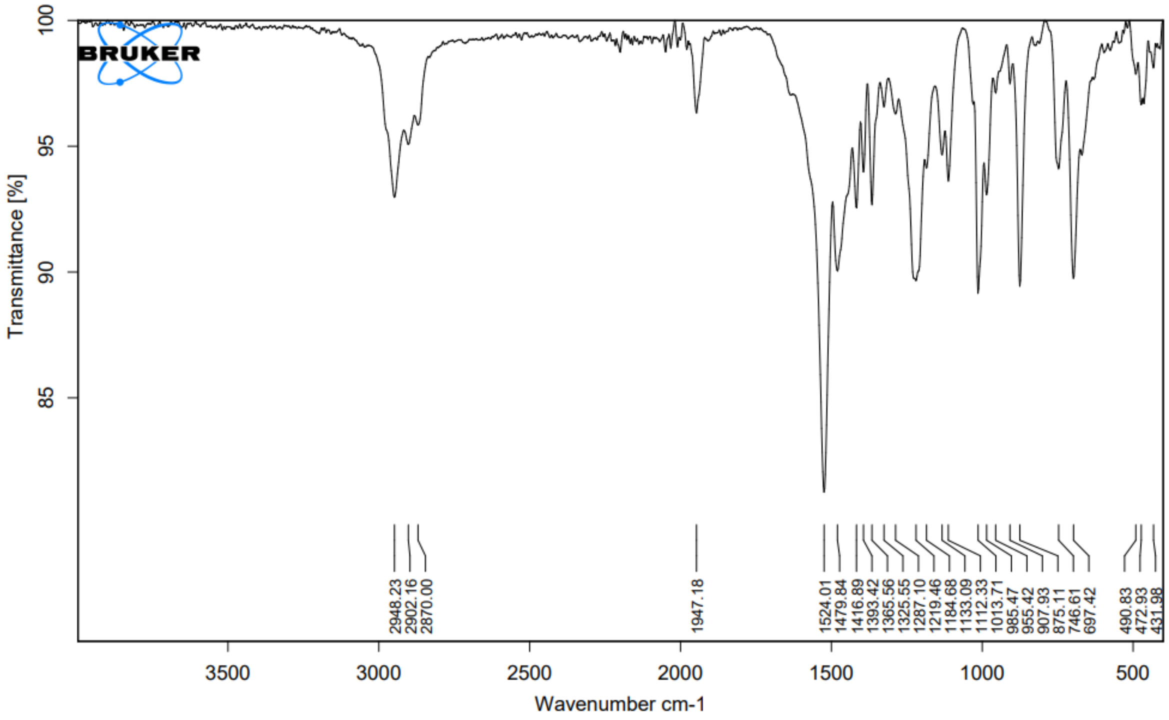

Figure S41: IR (neat) of **6**.

<u>PCl(NIdipp)*t*Bu **7**</u>

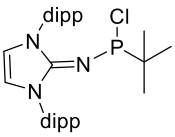

A solution of the silylated N-heterocyclic imine TMSNIdipp (2.000 g, 4.203 mmol, 1.00 eq.) in toluene (10 mL) was slowly added *via* cannula transfer to a stirred solution of *t*BuPCl$_2$ in toluene (15.8 mL, 0.271 M, 4.287 mmol, 1.02 eq.) at −78 °C. The reaction mixture was stirred for 30 minutes while keeping it at -78 °C, then allowed to warm to room temperature and stirred for an additional 16 h. All volatiles including excess *t*BuPCl$_2$ were removed *in vacuo* (60 °C at 10^{-3} mbar) affording the title compound as a beige/white powder (2.078 g; 3.949 mmol, 94%).

^{1}H NMR (400 MHz, C_6D_6) δ (ppm) = 7.26 - 7.22 (m, Ar-H (para)), 7.15 - 7.11(m, 4H, Ar-H (meta)), 5.98 (s, 2H, NCHCHN), 3.16 (sept, $^3J_{HH}$ = 6.9 Hz, 2H, CH (*i*Pr)), 2.98 (sept, $^3J_{HH}$ = 6.9 Hz, 2H, CH (*i*Pr)), 1.49 (d, $^3J_{HH}$ = 6.9 Hz, 6H, CH$_3$ (*i*Pr)), 1.43 (d, $^3J_{HH}$ = 6.8 Hz, 6H, CH$_3$ (*i*Pr)), 1.15 (d, $^3J_{HH}$ = 2.3 Hz, 6H, CH$_3$ (*i*Pr)), 1.13 (d, $^3J_{HH}$ = 2.3 Hz, 6H, CH$_3$ (*i*Pr)), 0.96 (d, $^3J_{HP}$ = 12 Hz, 9H, CH$_3$ (*t*Bu)).

^{1}H{^{31}P} NMR (300 MHz, C_6D_6) δ (ppm) = 7.28 - 7.23 (m, 2H, Ar-H (para)), 7.17 - 7.12 (m, 4H, Ar-H (meta)), 5.99 (s, 2H, NCHCHN), 3.17 (sept, $^3J_{HH}$ = 6.9 Hz, 2H, CH (*i*Pr)), 2.99 (sept, $^3J_{HH}$ = 6.9 Hz, 2H, CH (*i*Pr)), 1.50 (d, $^3J_{HH}$ = 6.9 Hz, 6H, CH$_3$ (*i*Pr)), 1.44 (d, $^3J_{HH}$ = 6.9 Hz, 6H, CH$_3$ (*i*Pr)), 1.17 (d, $^3J_{HH}$ = 1.8 Hz, 6H, CH$_3$ (*i*Pr)), 1.14 (d, $^3J_{HH}$ = 1.8 Hz, 6H, CH$_3$ (*i*Pr)), 0.97 (s, 9H, CH$_3$ (*t*Bu)).

^{13}C{^{1}H} NMR (C_6D_6, 101 MHz) δ (ppm) = 150.4 (d, $^2J_{CP}$ = 13 Hz, NCN), 147.7 (s, qC (Ar-*i*Pr)), 147.6 (s, qC (Ar- *i*Pr)), 147.1, (s, ipso-C (Ar-H)), 133.6, (s, ortho-C (Ar))), 130.3 (s, para-C (Ar-H)), 124.3 (s, meta-C (Ar-H)), 124.0 (s, meta-C (Ar-H)), 116.3 (s, NCHCHN), 36.7 (d, $^1J_{CP}$ = 24 Hz, qC (*t*Bu)), 29.3 (s, CH (*i*Pr)), 29.3 (s, CH (*i*Pr)), 29.2 (s, CH (*i*Pr)), 25.1 (s, CH$_3$ (*i*Pr)), 24.9 (s, CH$_3$ (*i*Pr)), 24.8 (d, $^2J_{CP}$ = 18 Hz, CH$_3$ (*t*Bu)), 23.3 (s, CH$_3$ (*i*Pr)), 22.9 (s, CH$_3$ (*i*Pr)), 22.8 (s, CH$_3$ (*i*Pr)).

^{31}P NMR (C_6D_6, 162 MHz) δ (ppm) = 157.6 (decet, $^3J_{PH}$ = 13 Hz).

^{31}P{^{1}H} NMR (162 MHz, C_6D_6) δ (ppm) = 157.6 (s).

HRMS: (DART, DCM) m/z = 526.3123 ([M+H]+, calculated: 526.3113).

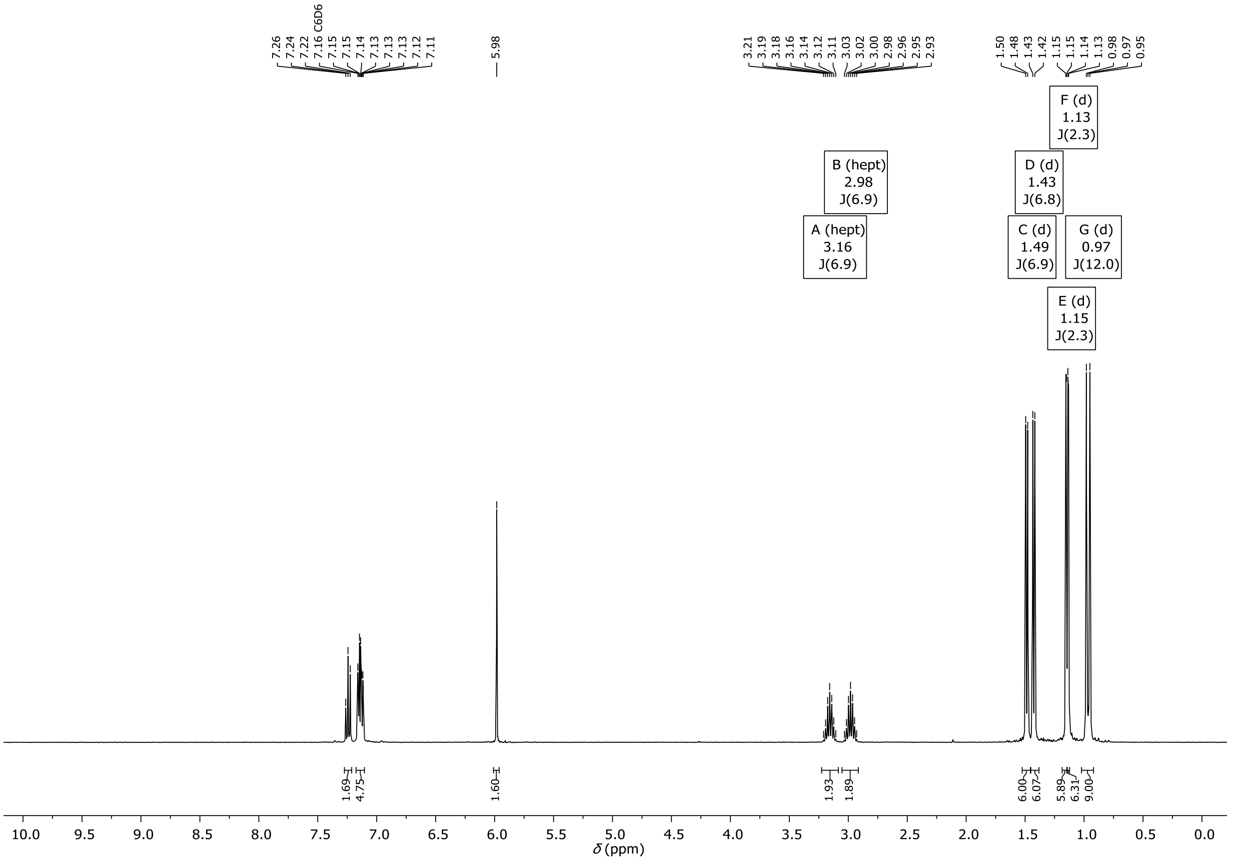

Figure S42: ^{1}H NMR spectrum (400 MHz, C_6D_6) of **7**.

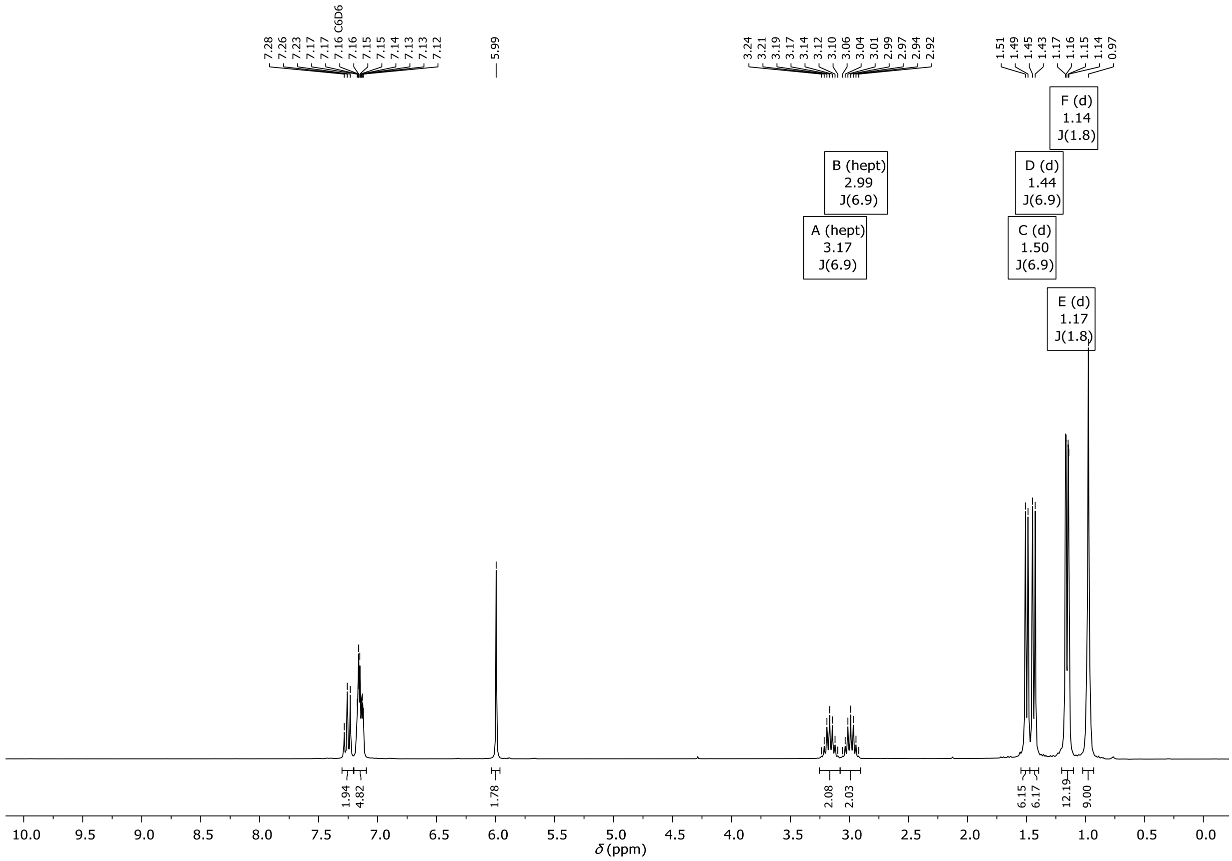

Figure S43: $^{1}H\{^{31}P\}$ NMR spectrum (300 MHz, C_6D_6) of **7**.

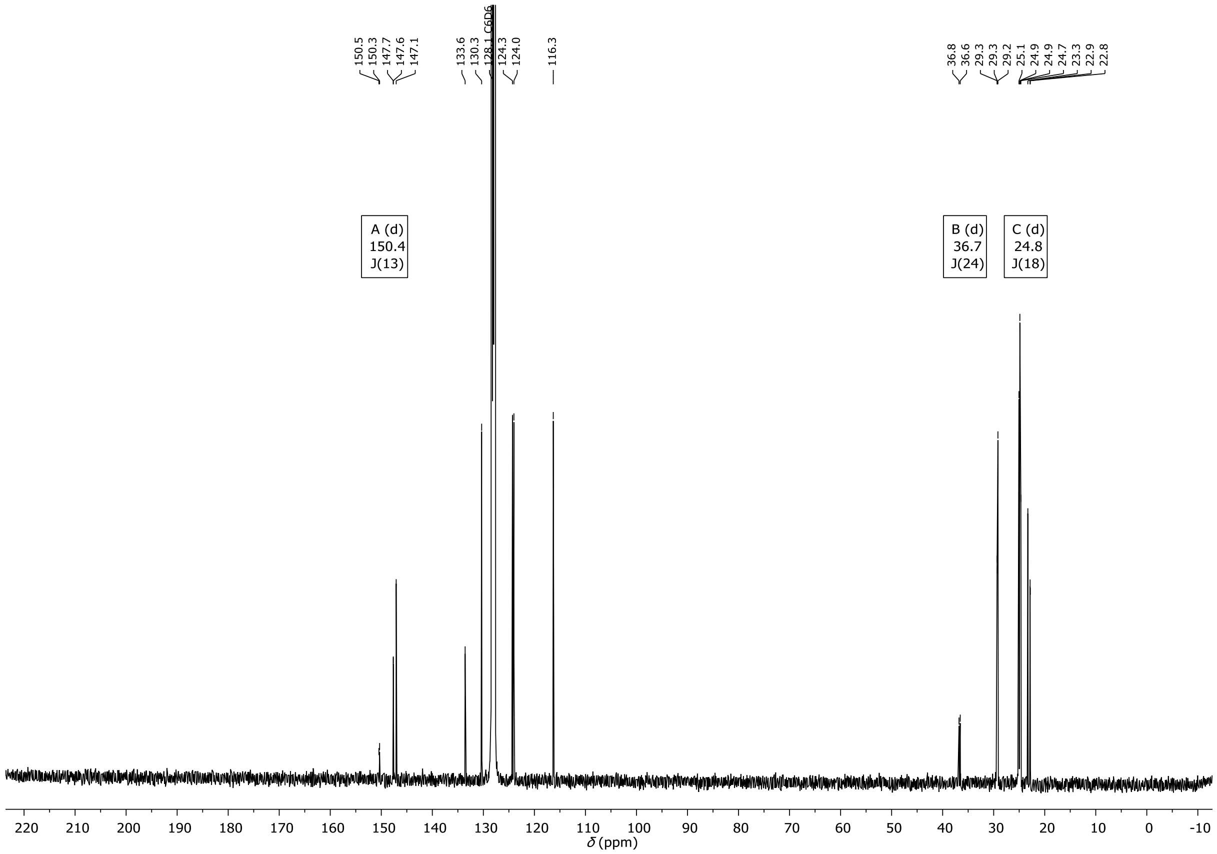

Figure S44: $^{13}C\{^{1}H\}$ NMR spectrum (101 MHz, C_6D_6) of **7**.

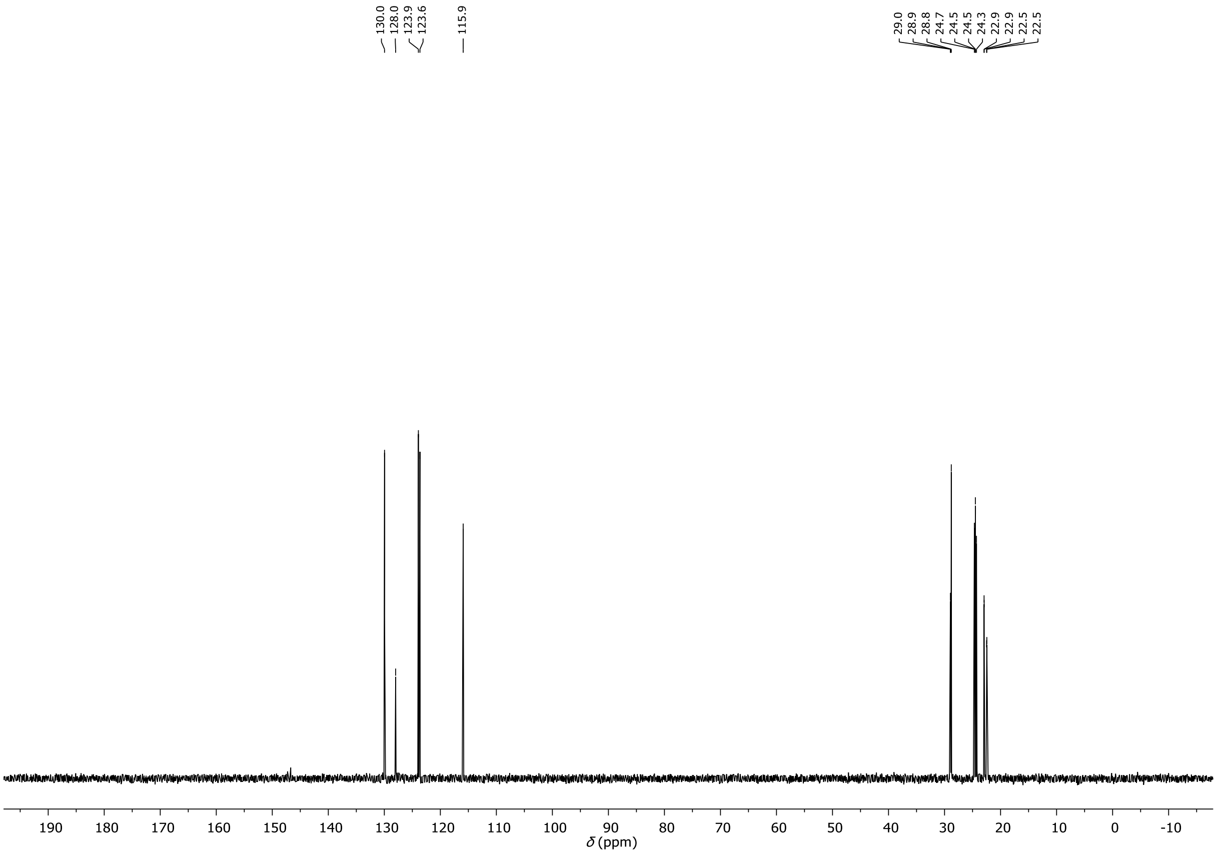

Figure S45: ^{13}C DEPT-135 spectrum (101 MHz, C_6D_6) of **7**.

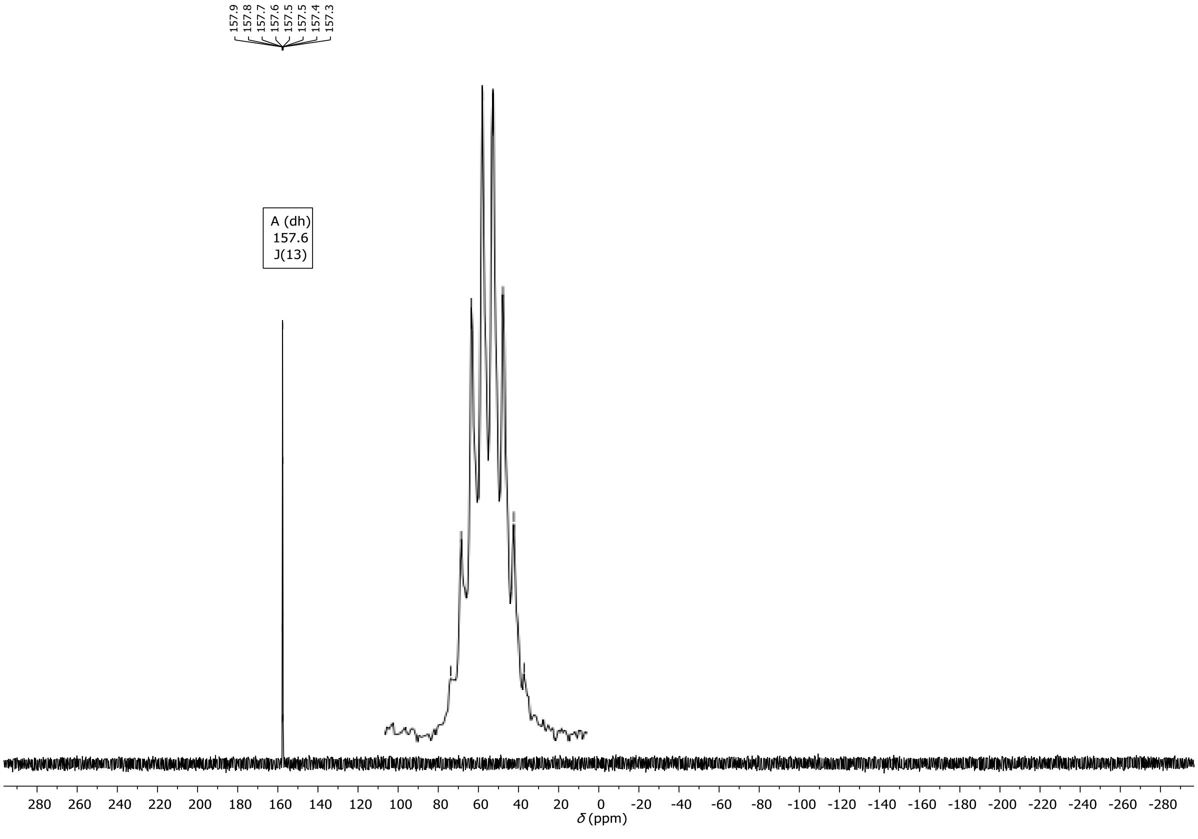

Figure S46: ^{31}P NMR spectrum (162 MHz, C_6D_6) of **7**.

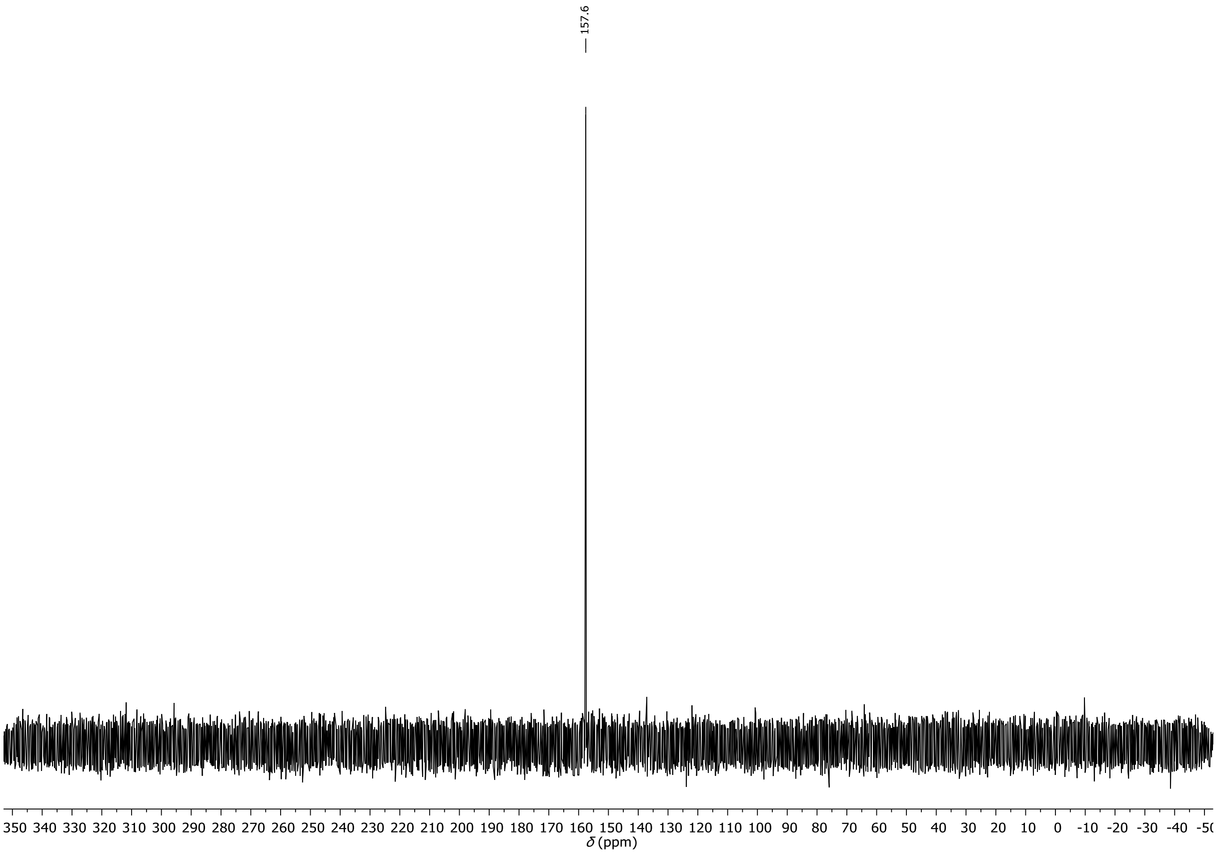

Figure S47: $^{31}P\{^{1}H\}$ NMR spectrum (162 MHz, C_6D_6) of **7**.

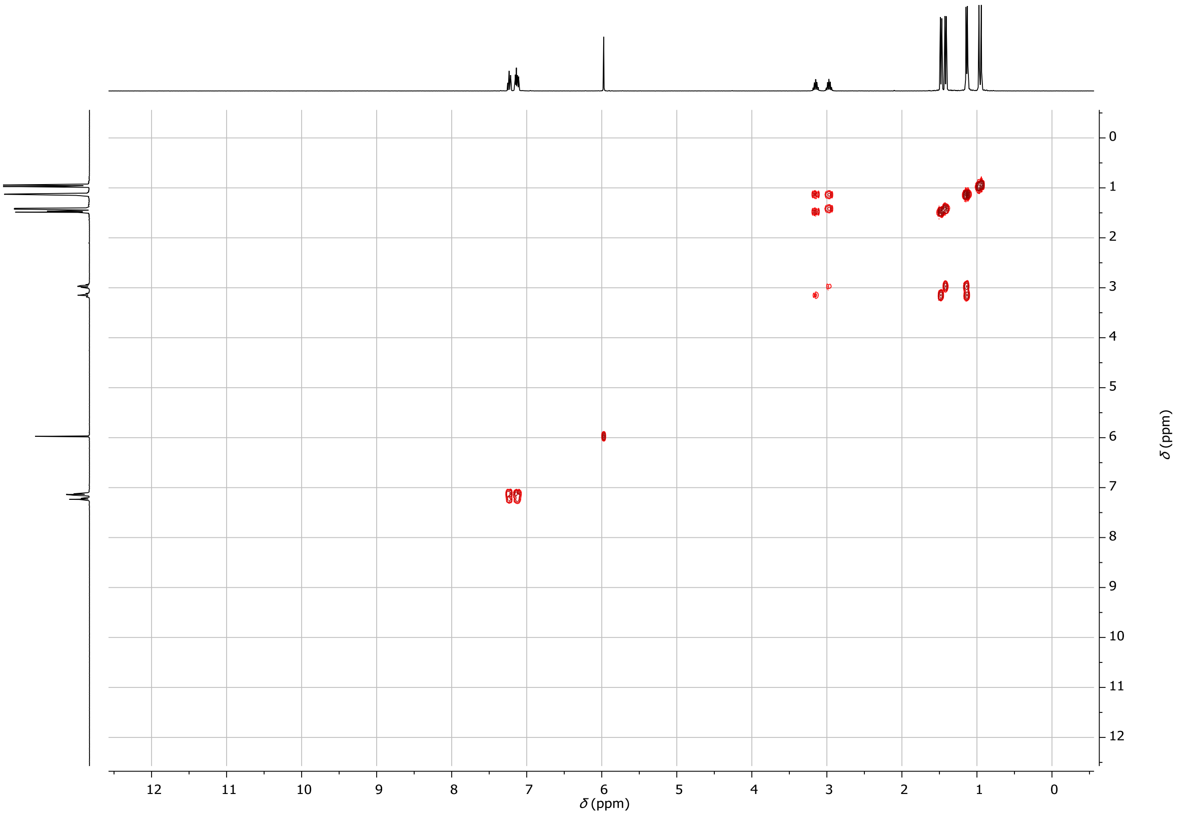

Figure S48: COSY (^{1}H, ^{1}H) NMR spectrum (400 MHz, 400 MHz, C_6D_6) of **7**.

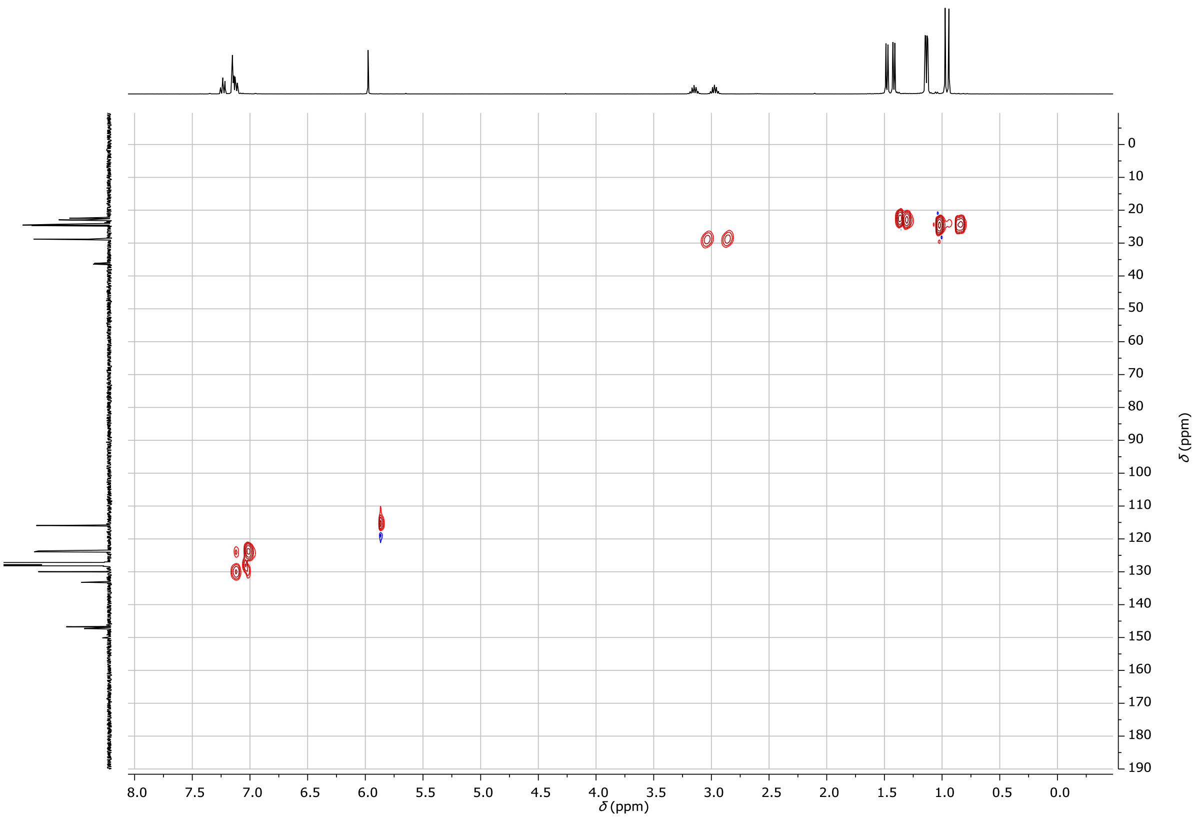

Figure S49: HSQC (^{1}H, ^{13}C) NMR spectrum (400 MHz, 101 MHz, C_6D_6) of **7**.

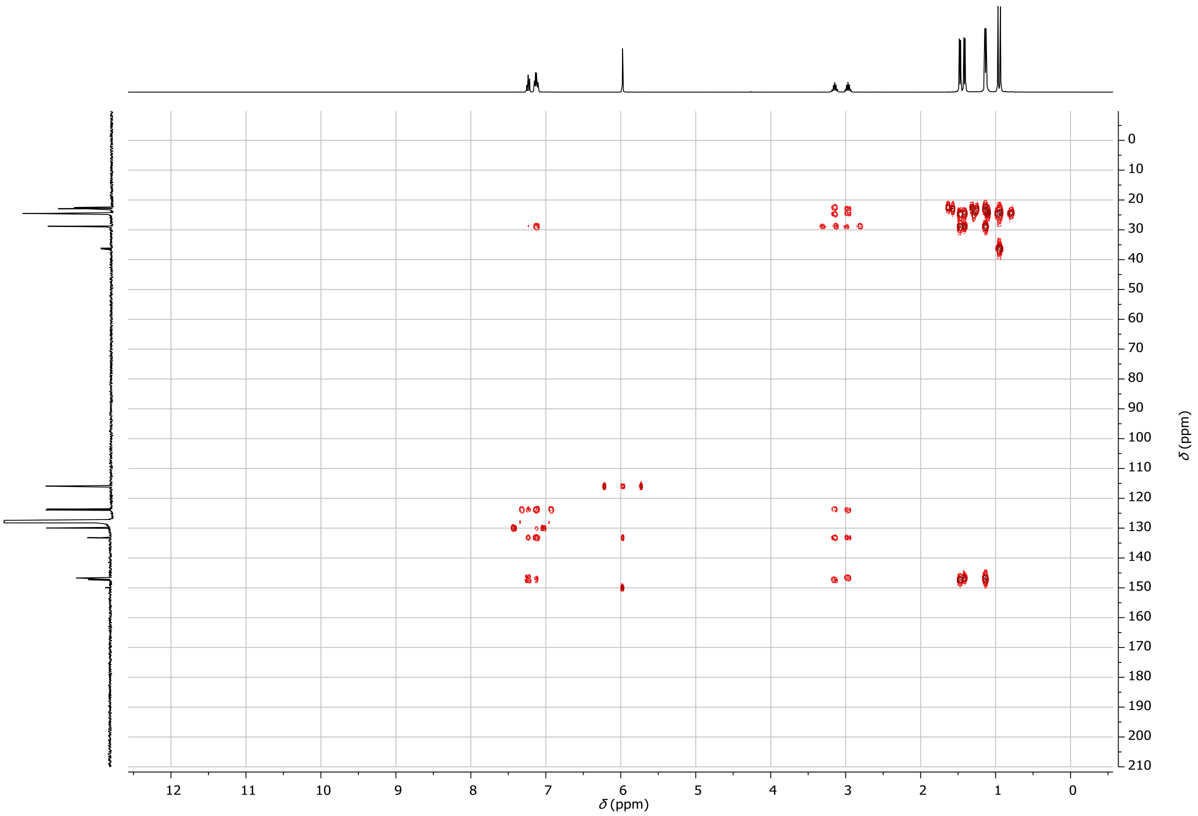

Figure S50: HMBC (^{1}H, ^{13}C) NMR spectrum (400 MHz, 101 MHz, C_6D_6) of **7**.

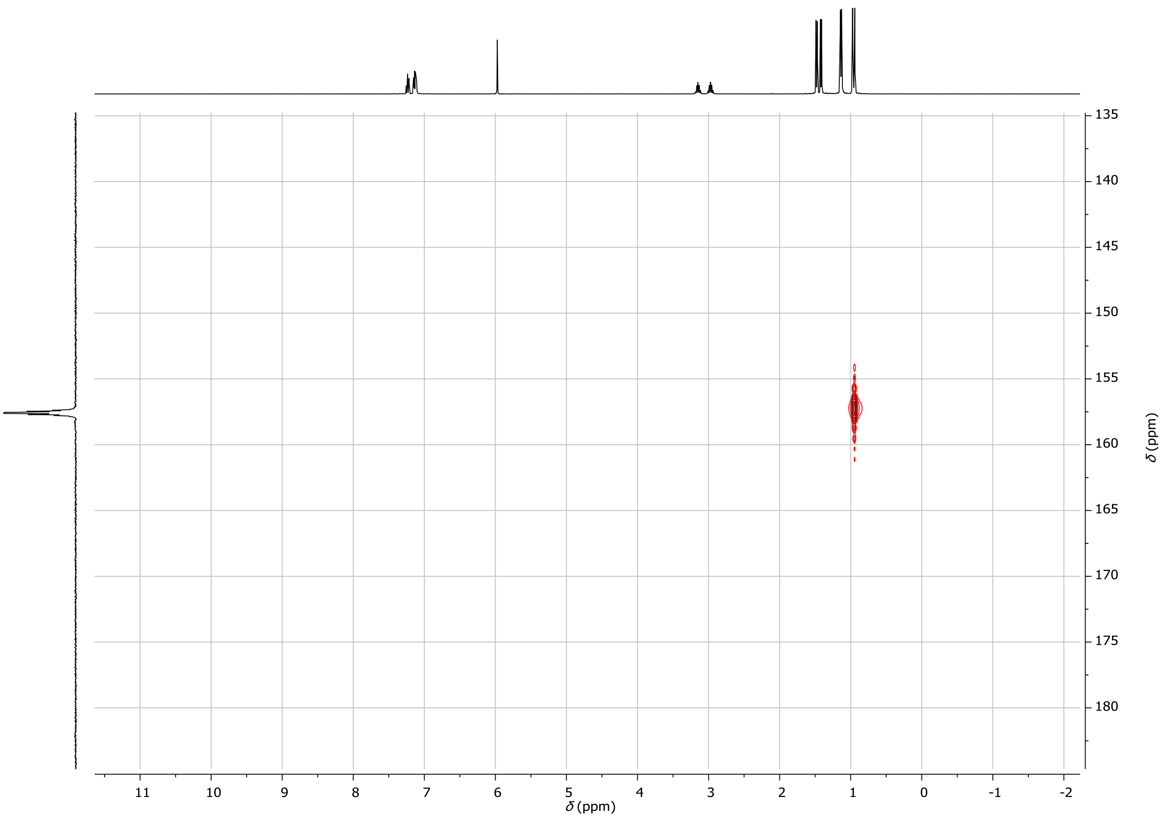

Figure S51: HMBC (^{1}H, ^{31}P) NMR spectrum (300 MHz, 122 MHz, C_6D_6) of **7**.

<u>P(CCH)(NIdipp)*t*Bu **8**</u>

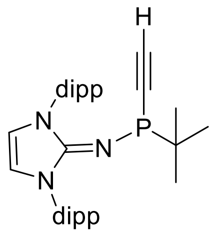

Ethynylmagnesium chloride in THF (8.1 mL, 0.5 M, 4.052 mmol, 2.00 eq.) was added dropwise to **7** (1.066 g, 2.026 mmol, 1.00 eq.) in THF (20 mL) at −78 °C. The reaction mixture was allowed to warm to room temperature over 16 h while stirring. All volatile compounds were removed *in vacuo*. The residue was extracted with hexane (3x 20 mL). All volatiles were removed *in vacuo* and the product was received as a white solid (1.673 g, 0.863 mmol, 83%).

^{1}H NMR (400 MHz, C_6D_6) δ (ppm) = 7.26 - 7.10 (m, 6H, Ar-H), 5.95 (s, 2H, NCHCHN), 3.31 (hept, $^3J_{HH}$ = 6.9 Hz, 2H, CH (*i*Pr)), 3.07 (hept, $^3J_{HH}$ = 6.9 Hz, 2H, CH (*i*Pr)), 2.24 (d, $^3J_{HP}$ = 1.8 Hz, 1H, CCH), 1.50 (d, $^3J_{HH}$ = 6.8 Hz, 6H, CH_3 (*i*Pr)), 1.41 (d, $^3J_{HH}$ = 6.9 Hz, 6H, CH_3 (*i*Pr)), 1.19 (d, $^3J_{HH}$ = 6.9 Hz, 12H, CH_3 (*i*Pr)), 1.02 ((d, $^3J_{PH}$ = 13.4 Hz, 9H, CH_3 (*t*Bu)).

^{1}H{^{31}P} NMR (400 MHz, C_6D_6) δ (ppm) = 7.26 -7.10 (m, 6H, Ar-H), 5.95 (s, 2H, NCHCHN), 3.31 (hept, $^3J_{HH}$ = 6.9 Hz, 2H, CH (*i*Pr)), 3.07 (hept, $^3J_{HH}$ = 6.9 Hz, 2H, CH (*i*Pr)), 2.24 (s, 1H, CCH), 1.50 (d, $^3J_{HH}$ = 6.8 Hz, 6H, CH_3 (*i*Pr)), 1.41 (d, $^3J_{HH}$ = 6.9 Hz, 6H, CH_3 (*i*Pr)), 1.19 (dd, $^3J_{HH}$ = 6.9 Hz, $^3J_{HH}$ = 1.0 Hz, 12H, CH_3 (*i*Pr)), 1.02 (s, 9H, CH_3 (*t*Bu)).

^{13}C{^{1}H} NMR (101 MHz, C_6D_6) δ (ppm) = 148.8 (d, $^2J_{CP}$ = 18 Hz, NCN), 148.5 (d, J_{CP} = 2 Hz, q-C (Ar-*i*Pr)), 147.4 (s, C (Ar-H)), 134.5 (s, ipso-C (Ar)), 129.9 (s, C (Ar-H)), 124.0 (s, C (Ar-H)), 123.9 (s, C (Ar-H)), 115.5 (s, NCHCHN), 89.3 (d, $^2J_{CP}$ = 9 Hz, βC (H-CC)), 89.2 (d, $^1J_{CP}$ = 54 Hz, αC (P-CC)), 32.5 (d, $^1J_{CP}$ = 3 Hz, q-C (*t*Bu)), 29.3 (d, J_{CP} = 3 Hz, CH (*i*Pr)), 29.0 (s, CH (*i*Pr)), 25.8 (d, $^2J_{CP}$ = 16 Hz, CH_3 (*t*Bu)), 25.1 (s, CH_3 (*i*Pr)), 24.8 (s, CH_3 (*i*Pr)), 23.6 (s, CH_3 (*i*Pr)), 22.8 (d, J_{CP} = 4 Hz, CH_3 (*i*Pr)).

^{31}P NMR (162 MHz, C_6D_6) δ (ppm) = 34.4 (dh, $^3J_{HP}$ = 27 Hz, $^3J_{HP}$ = 14 Hz).

^{31}P{^{1}H} NMR (162 MHz, C_6D_6) δ (ppm) = 34.4 (s).

Elemental analysis Calculated for $C_{33}H_{46}N_3P$: C 76.86 %, H 8.99 %, N 8.15 %; found: C 76.20 %, H 8.992 %, N 8.01 %.

HRMS (ESI, CH_3CN) m/z = 516.3483 ([M+H]$^+$, calculated: 516.3502).

IR (neat): ν_{max}/cm^{-1} = 3294.54 (*H-C*≡C), 2960.16, 2934.01, 2867.86, 1607.11, 1587.53, 1566.73, 1468.70, 1457.93, 1409.46, 1383.37, 1361.27, 1330.32, 1297.91, 1257.47, 1230.07, 1180.35, 1125.20, 1105.58, 1061.01, 1011.59, 926.84, 905.20, 885.70, 799.57, 771.91, 757.64, 673.53, 661.37, 640.37, 601.43, 583.08, 538.53, 492.61, 438.09, 406.08.

IR (in DCM): ν_{max}/cm^{-1} = 3293.03 (*H-C*≡C), 2962.61, 2936.42, 2868.90, 1609.50, 1587.40, 1568.10, 1468.10, 1409.70, 1383.89, 1362.13, 1330.66, 1297.97, 1264.64, 1230.10, 1181.74, 1125.70, 1060.24, 1010.67, 926.23, 905.50, 803.34, 772.74, 760.04, 737.64, 705.25, 667.51, 609.18, 539.91, 438.16.

Note that DFT calculations showed that the intensity of the (C≡C) stretching band is very low, therefore not observed in IR (see Figure S166).

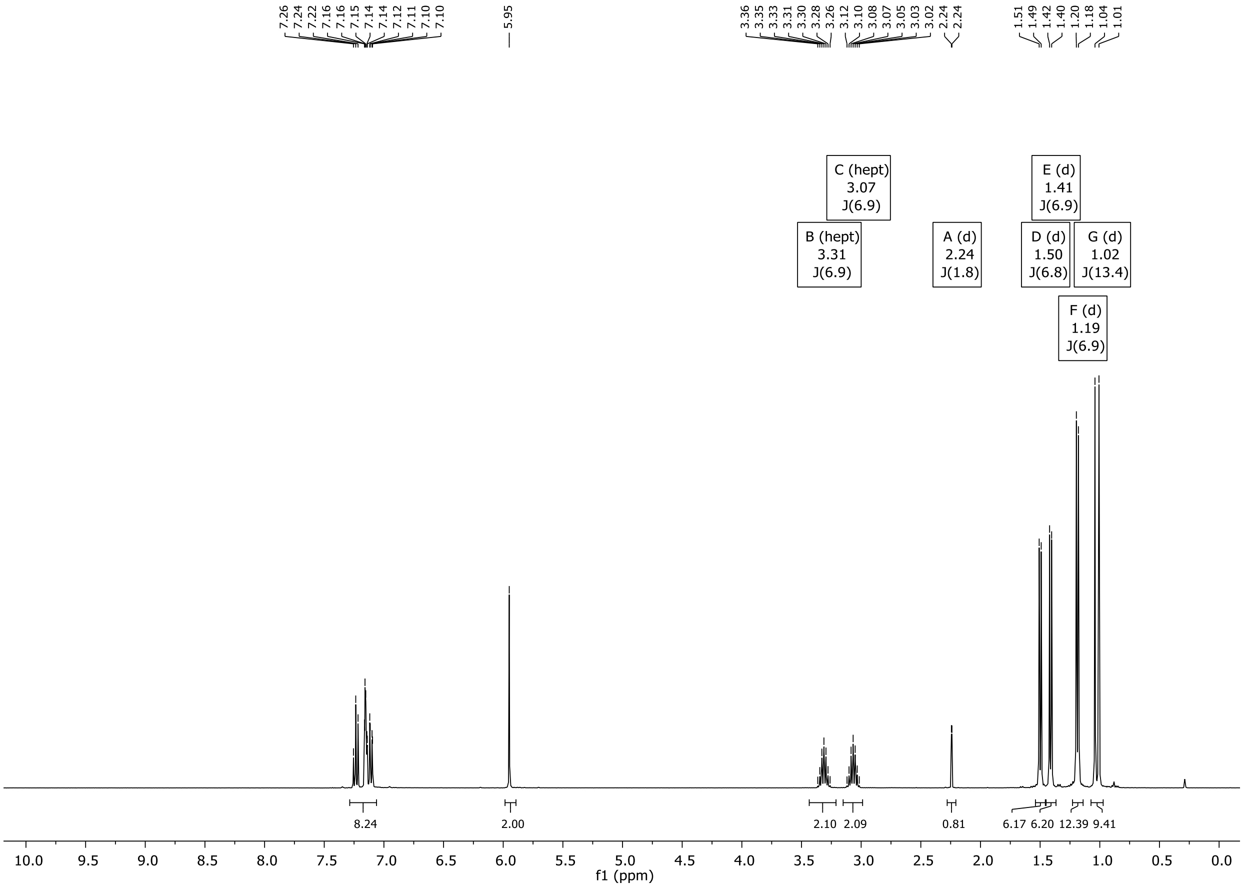

Figure S52: ^{1}H NMR spectrum (400 MHz, C_6D_6) of **8**.

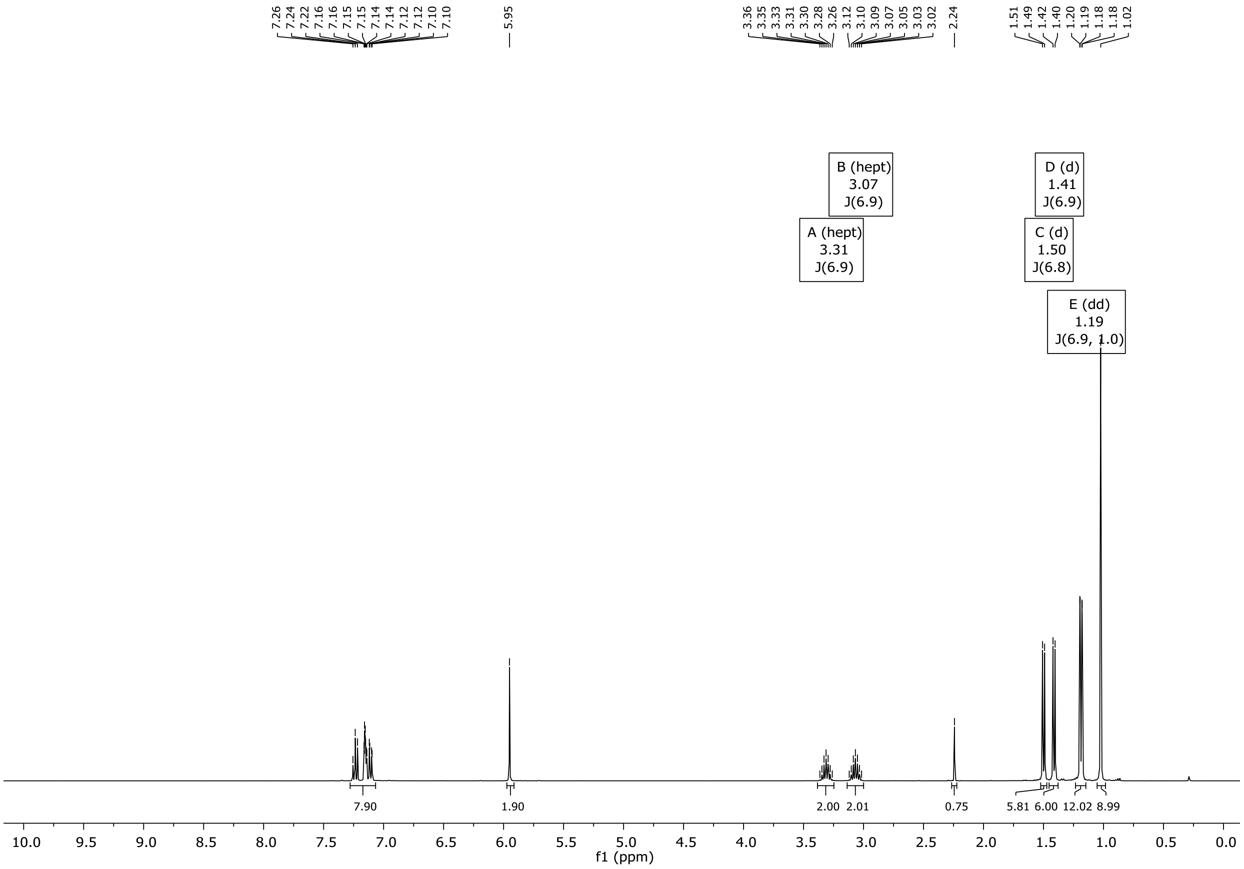

Figure S53: $^{1}H\{^{31}P\}$ NMR spectrum (400 MHz, C_6D_6) of **8**.

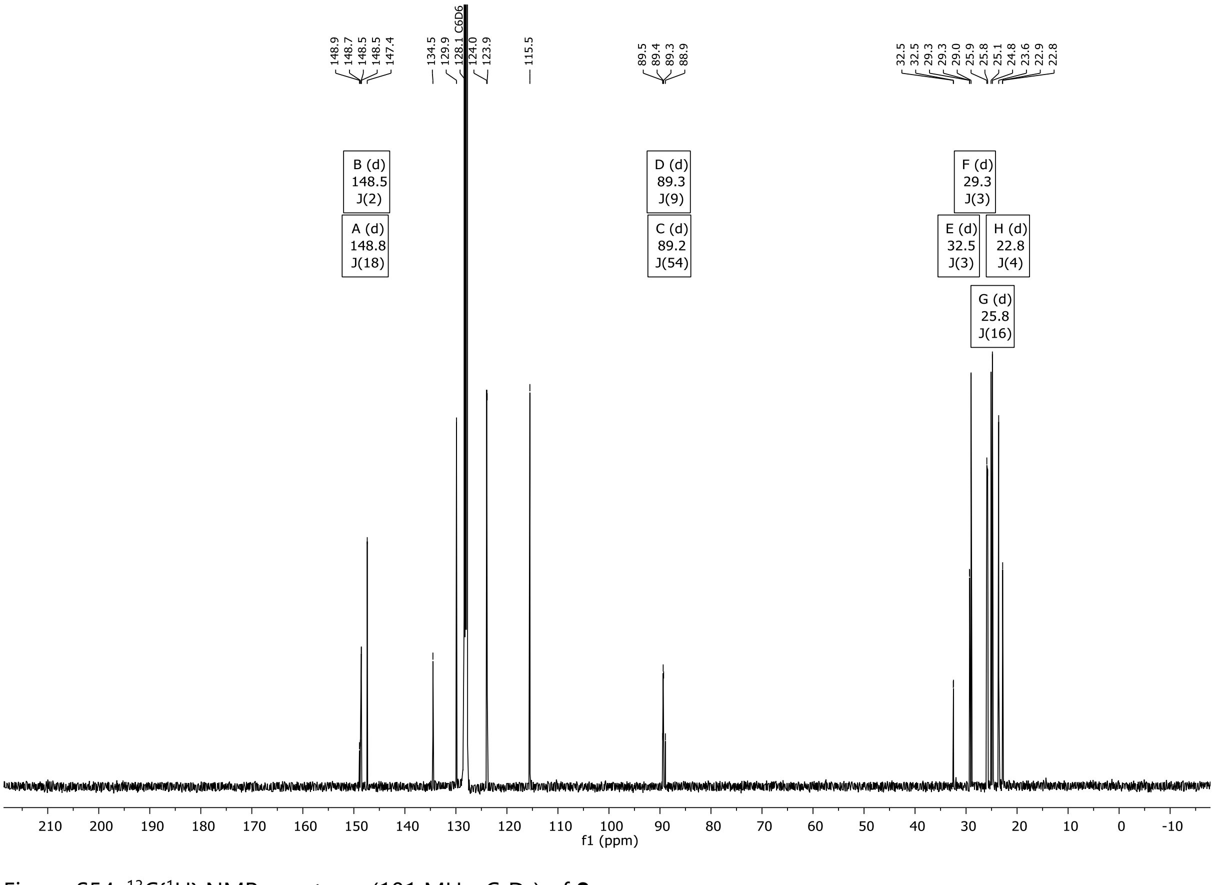

Figure S54: $^{13}C\{^{1}H\}$ NMR spectrum (101 MHz, C_6D_6) of **8**.

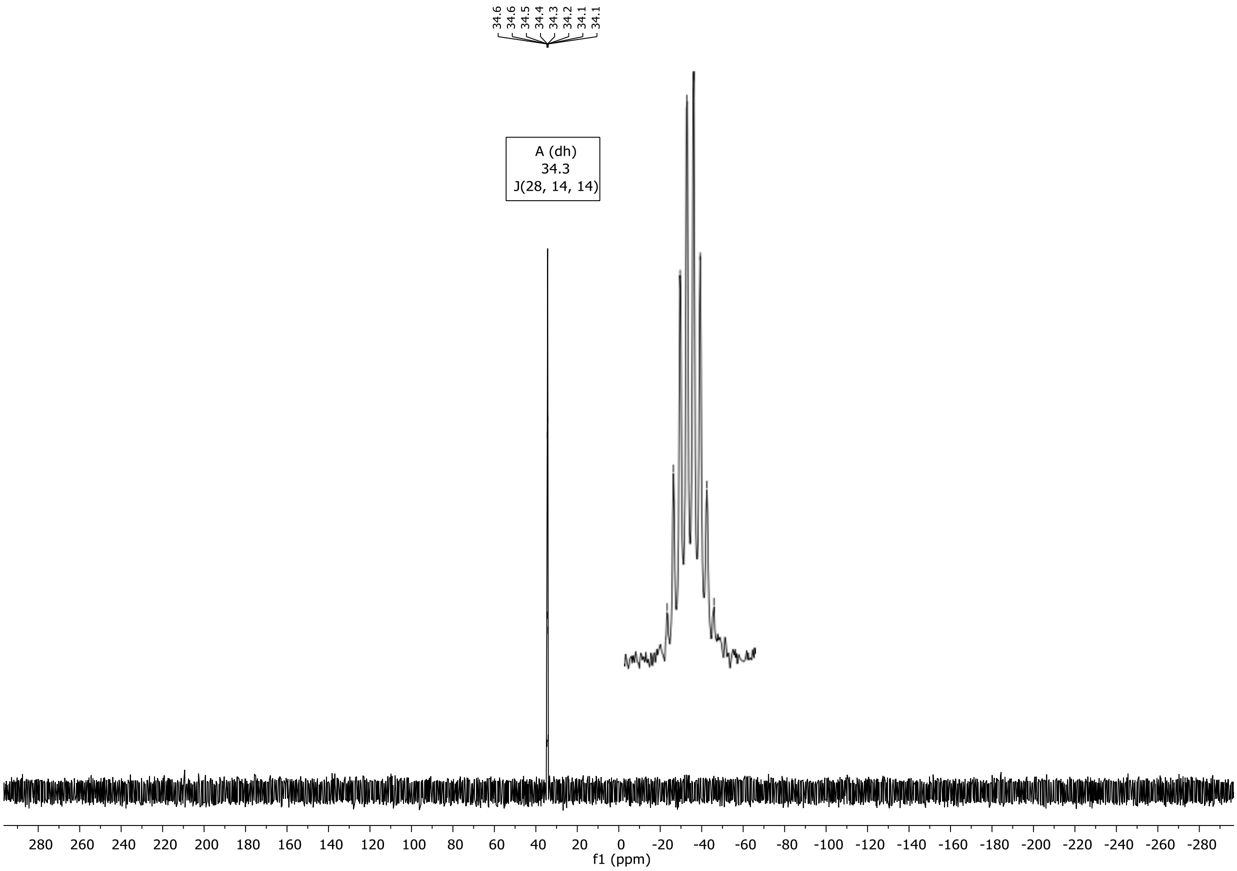

Figure S55: ^{31}P NMR spectrum (162 MHz, C_6D_6) of **8**.

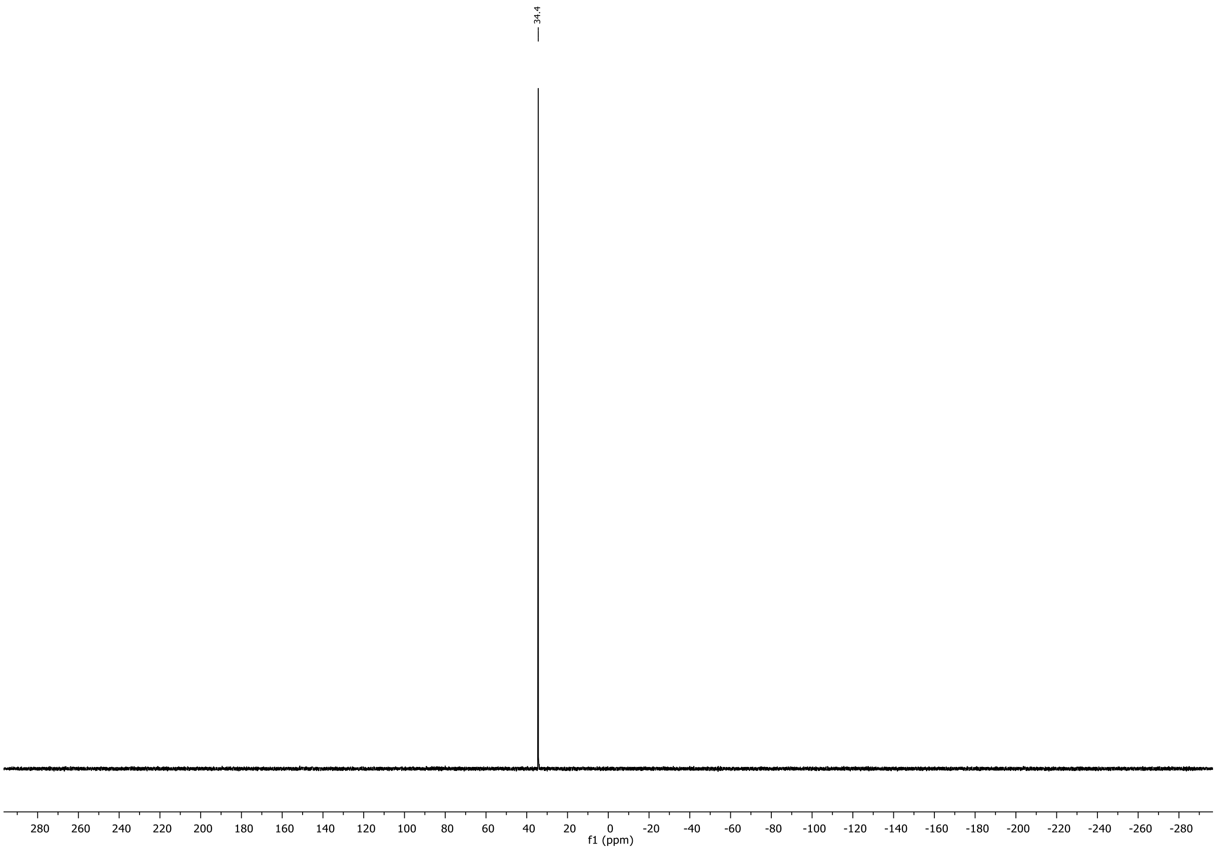

Figure S56: $^{31}P\{^1H\}$ NMR spectrum (162 MHz, C_6D_6) of **8**.

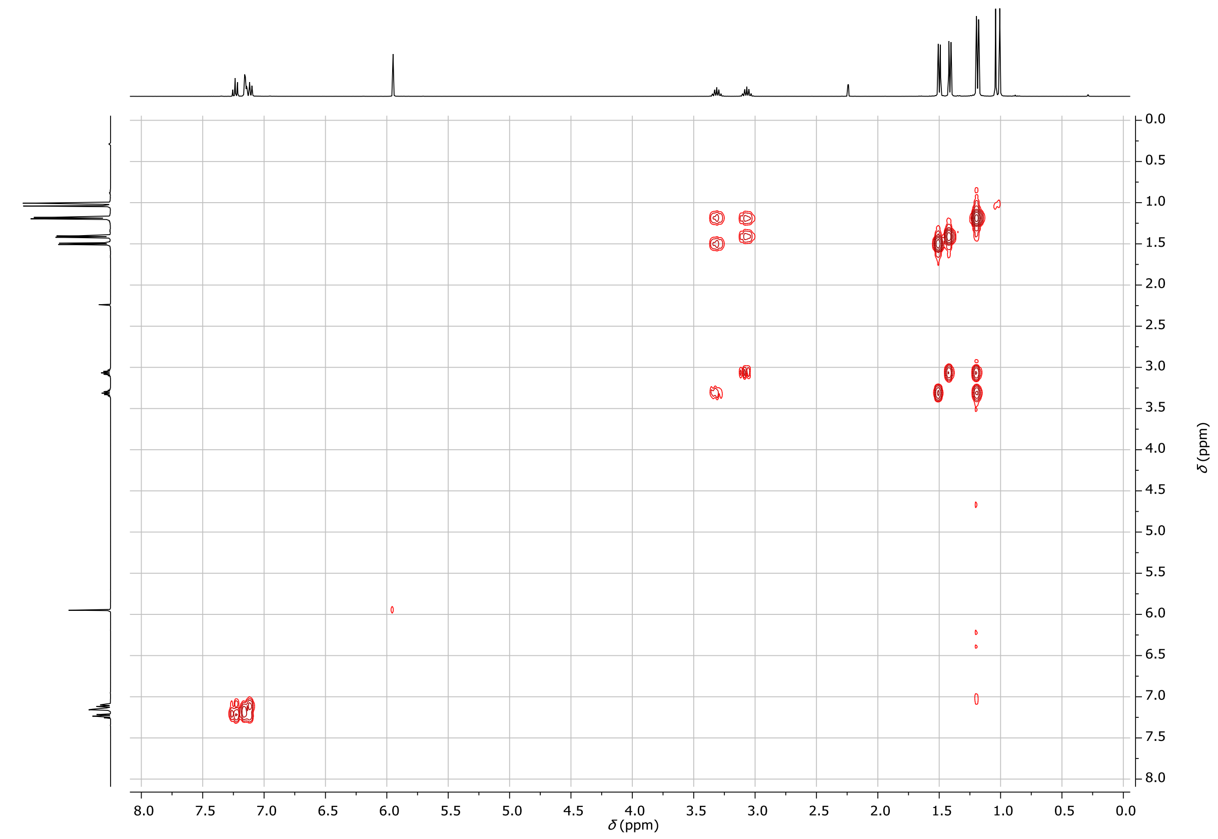

Figure S57: COSY (^1H, ^1H) NMR spectrum (400 MHz, 400 MHz, C_6D_6) of **8**.

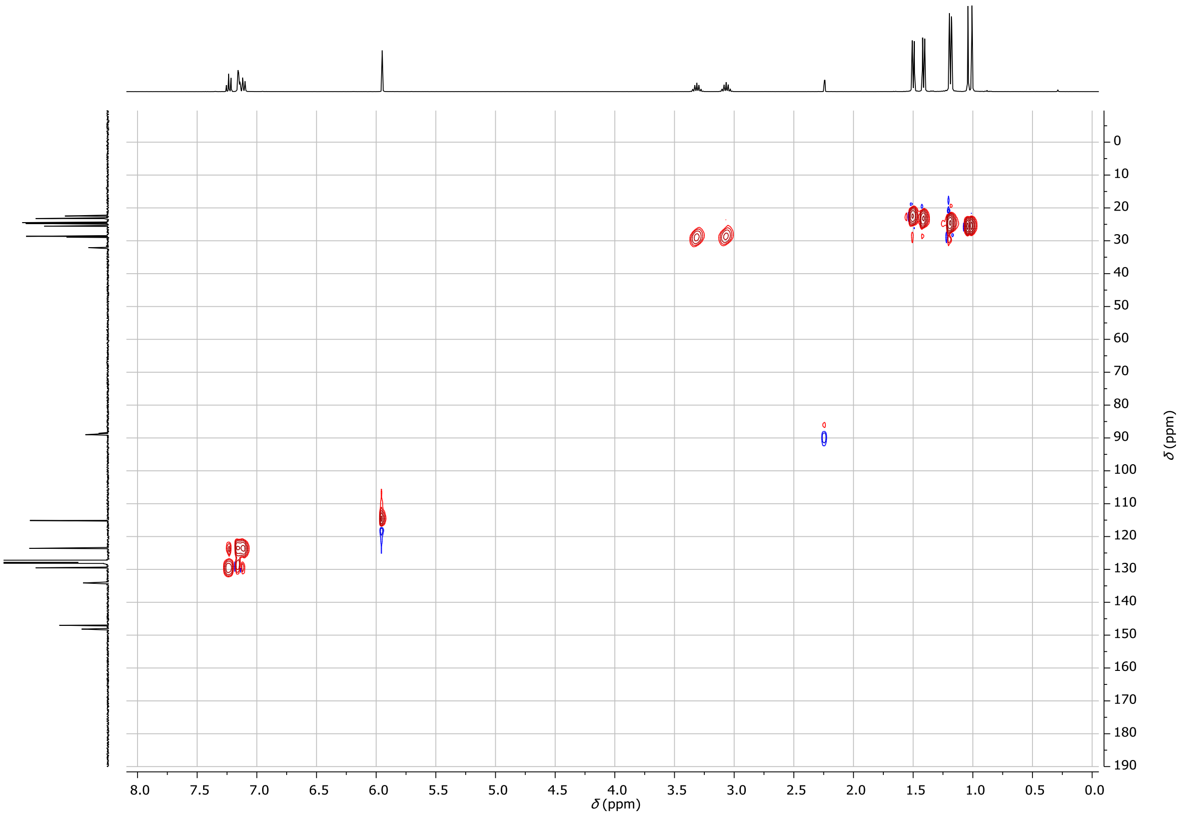

Figure S58: HSQC (^1H, ^{13}C) NMR spectrum (400 MHz, 101 MHz, C_6D_6) of **8**.

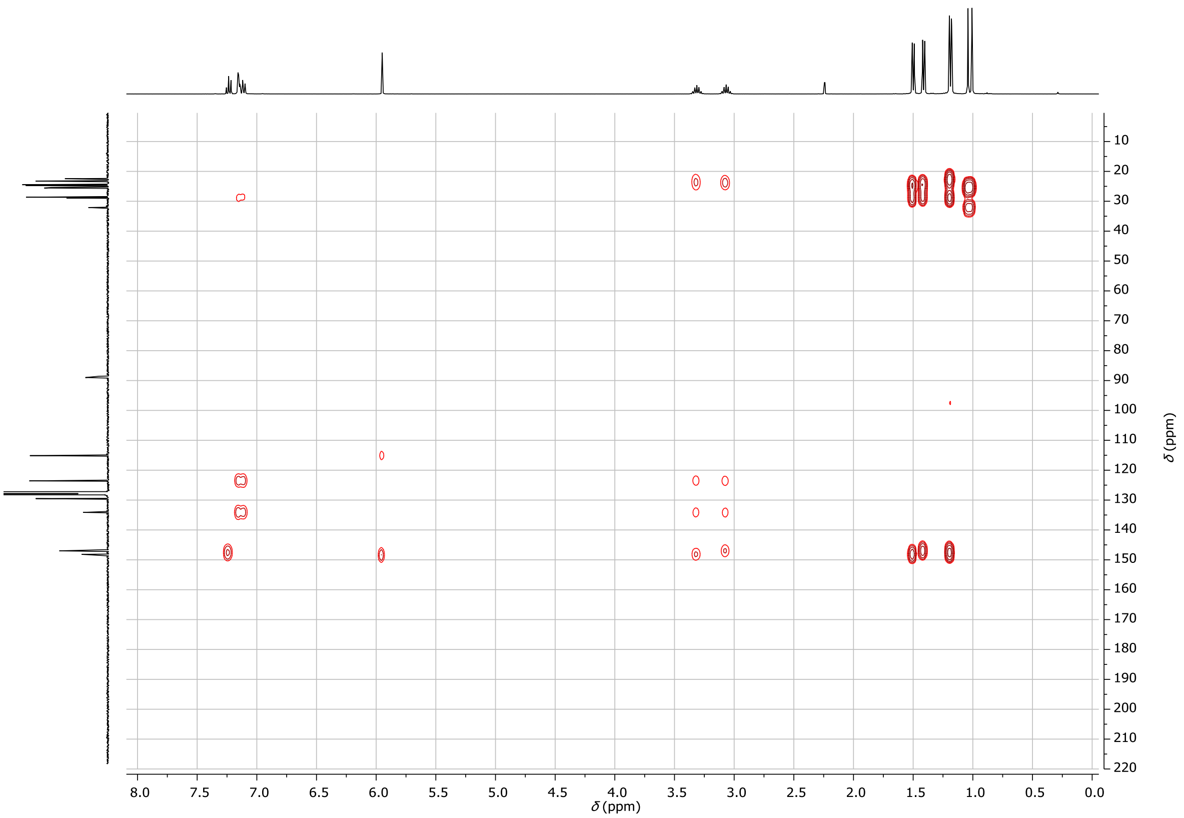

Figure S59: HMBC (^{1}H, ^{13}C) NMR spectrum (400 MHz, 101 MHz, C_6D_6) of **8**.

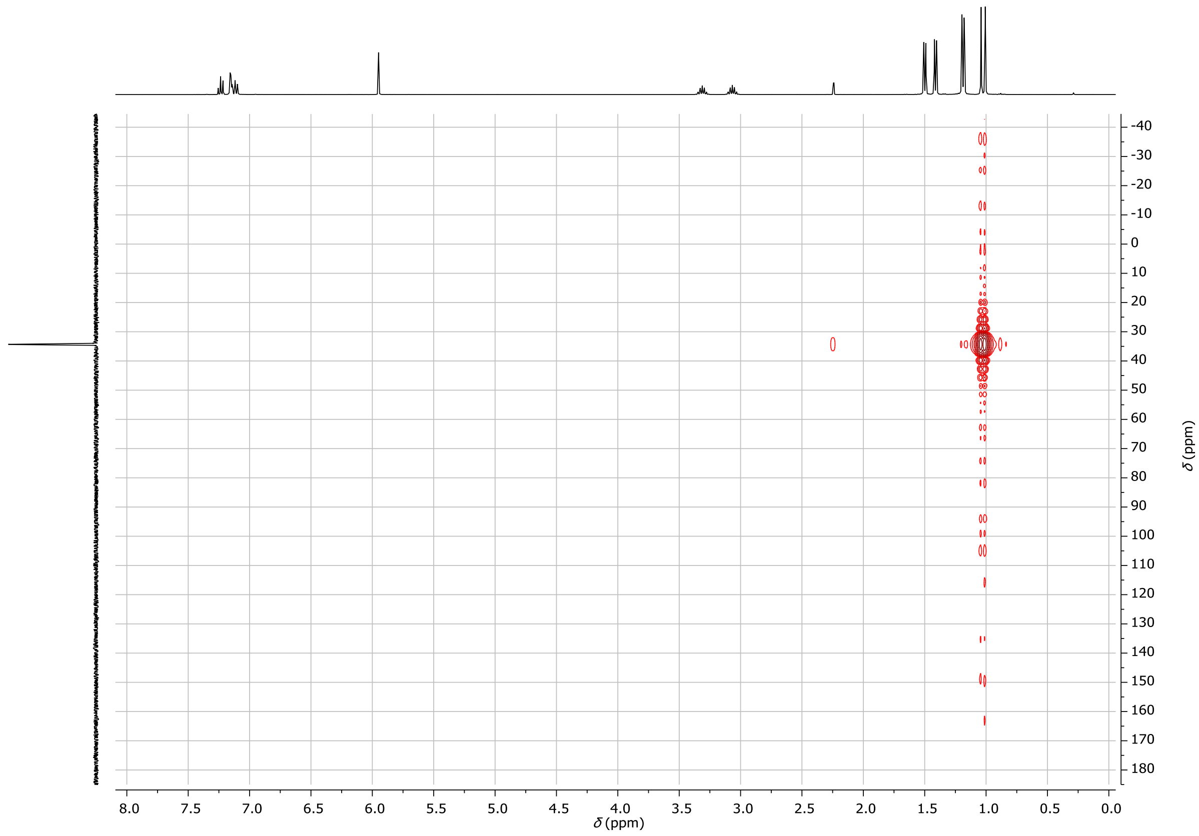

Figure S60: HMBC (^{1}H, ^{31}P) NMR spectrum (400 MHz, 162 MHz, C_6D_6) of **8**.

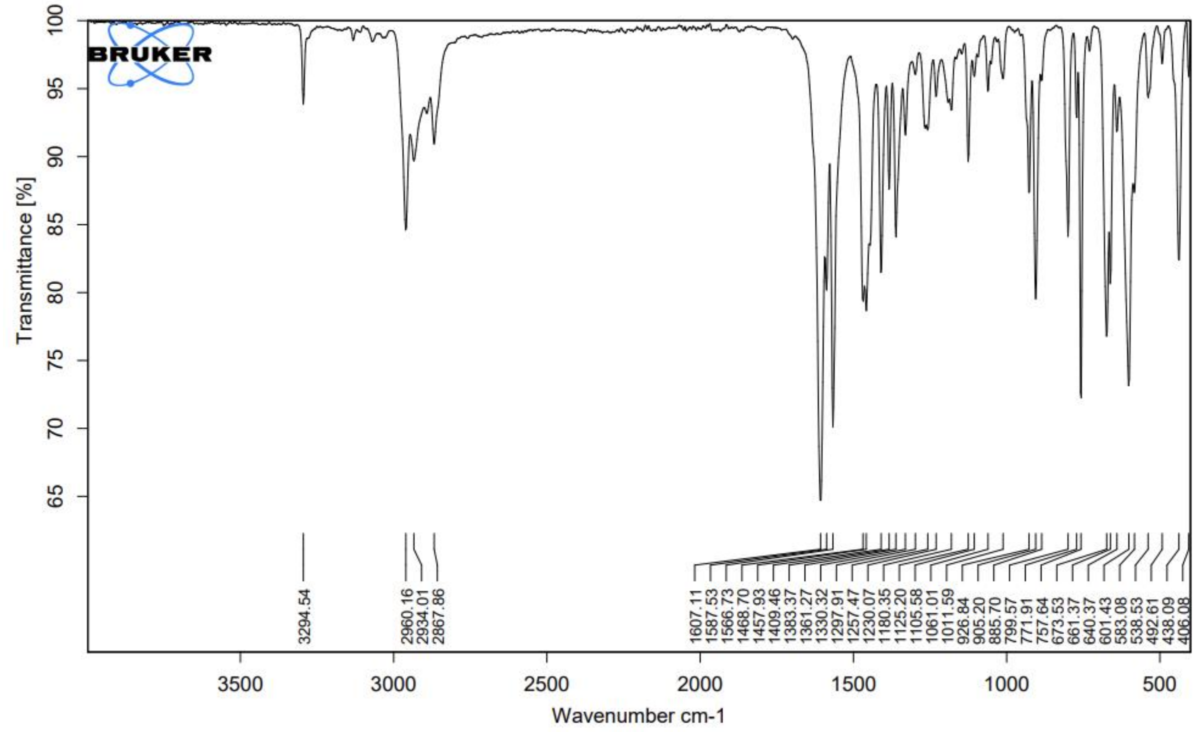

Figure S61: IR spectrum (neat) of **8**.

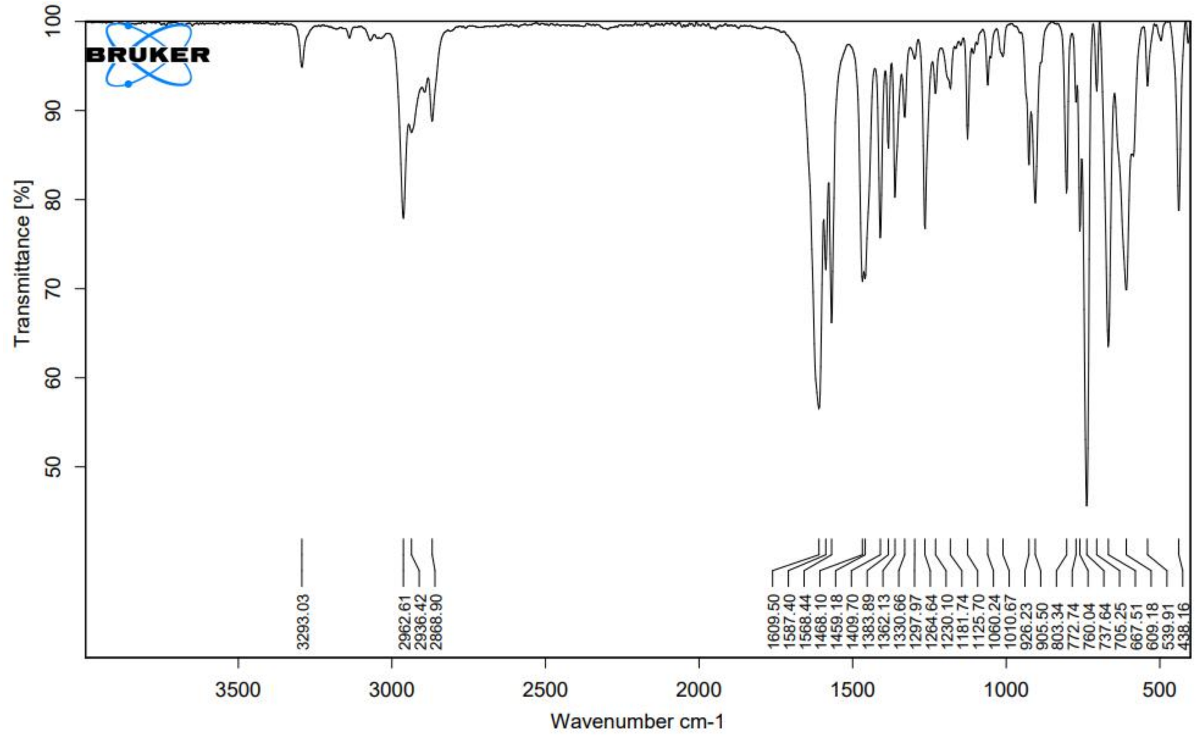

Figure S62: IR spectrum (in DCM) of **8**.

[P(CCH)Me(NIdipp)*t*Bu][I] **9**

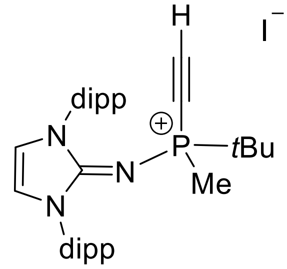

Methyl iodide in toluene (0.35 mL, 0.803 M, 0.281 mmol, 1.45 eq.) was added dropwise to a solution of **8** (100 mg, 0.194 mmol, 1.00 eq.) in toluene (20 mL). The reaction mixture was stirred overnight at ambient temperature. The resulting suspension was filtered, and the residue was washed with *n*-hexane (20 mL). The residue was dried *in vacuo* to afford the product **9** as a white solid (110 mg, 0.167 mmol, 86%).

^{1}H NMR (400 MHz, CD_3CN) δ (ppm) = 7.59 - 7.40 (m, 6H, Ar-H), 7.20 (s, 2H, NCHCHN), 3.69 (d, $^{3}J_{HP}$ = 9.7 Hz, 1H, CCH), 2.79 - 2.61 (m, 4H, CH (*i*Pr)), 1.32 (dd, J_{HH} = 6.8 Hz, J_{HH} = 12.2 Hz, 12H, CH_3 (*i*Pr)), 1.20 (t, J_{HH} = 6.9 Hz, 12H, CH_3 (*i*Pr)), 0.90 (d, $^{2}J_{HP}$ = 13.1 Hz, 3H, CH_3 (Me)), 0.76 ((d, $^{3}J_{PH}$ = 19.4 Hz, 9H, CH_3 (*t*Bu)).

^{1}H{^{31}P} NMR (400 MHz, CD_3CN) δ (ppm) = 7.59 - 7.40 (m, 6H, Ar-H), 7.20 (s, 2H, NCHCHN), 3.67 (s, 1H, CCH), 2.80 - 2.61 (m, 4H, CH (*i*Pr)), 1.32 (dd, J_{HH} = 6.9 Hz, J_{HH} = 12.1 Hz, 12H, CH_3 (*i*Pr)), 1.20 (t, J_{HH} = 6.8 Hz, 12H, CH_3 (*i*Pr)), 0.90 (s, 3H, CH_3 (Me)), 0.76 ((s, 9H, CH_3 (*t*Bu)).

^{13}C{^{1}H} NMR (101 MHz, CD_3CN) δ (ppm) = 147.7 (d, $^{2}J_{CP}$ = 13 Hz, NCN), 146.4 (s, q-C (Ar-*i*Pr)), 132.3 (s, C (Ar-H)), 132.1 (s, ipso-C (Ar)), 125.7 (s, C (Ar-H)), 125.6 (s, C (Ar-H)), 120.0 (s, NCHCHN), 98.9 (d, $^{2}J_{CP}$ = 21 Hz, βC (H-CC)), 72.8 (d, $^{1}J_{CP}$ = 136 Hz, αC (P-CC)), 33.5 (d, $^{1}J_{CP}$ = 95 Hz, q-C (*t*Bu)), 29.7 (d, J_{CP} = 3 Hz, CH (*i*Pr)), 25.4 (s, CH_3 (*i*Pr)), 25.4 (s, CH_3 (*i*Pr)), 23.1 (s, CH_3 (*i*Pr)), 22.9 (s, CH_3 (*i*Pr)), 22.6 (d, $^{3}J_{CP}$ = 1 Hz, CH_3 (*t*Bu)), 12.7 (d, $^{1}J_{CP}$ = 71 Hz, CH_3 (Me)).

^{31}P NMR (162 MHz, CD_3CN) δ (ppm) = 11.6 - 10.8 (m).

^{31}P{^{1}H} NMR (162 MHz, CD_3CN) δ (ppm) = 11.2 (s).

Elemental analysis Calculated for $C_{34}H_{49}IN_3P$: C 62.09 %, H 7.51 %, N 6.39 %; found: C 61.74 %, H 7.503 %, N 6.25 %.

HRMS (ESI, CH_3CN) m/z = 530.3641 ([M-I]$^{+}$, calculated: 530.3659).

IR (neat): ν_{max}/cm^{-1} = 3051.58 (*H-C*≡C), 2965.16, 2930.05, 2867.86, 2037.72 (C≡C), 1600.63, 1580.22, 1558.16, 1466.50, 1430.89, 1390.69, 1366.24, 1328.60, 1298.03, 1281.25, 1265.60, 1215.48, 1134.58, 1104.73, 1074.50, 1062.86, 1044.49, 980.77, 936.07, 891.83, 878.93, 818.53, 802.40, 791.83, 768.27, 755.41, 732.96, 709.32, 680.36, 600.36, 448.01, 415.89.

IR (in DCM): ν_{max}/cm^{-1} = 3050.08 (*H-C*≡C), 2964.91, 2930.01, 2868.63, 2037.14 (C≡C), 1599.98, 1579.40, 1465.23, 1430.10, 1389.30, 1365.67, 1328.27, 1297.82, 1268.43, 1214.75, 1103.74, 1073.98, 1061.68, 1044.20, 979.98, 935.89, 890.43, 878.11, 817.84, 802.51, 791.03, 768.47, 755.38, 742.13, 708.21, 695.13. 679.13, 599.97, 446.26, 415.09.

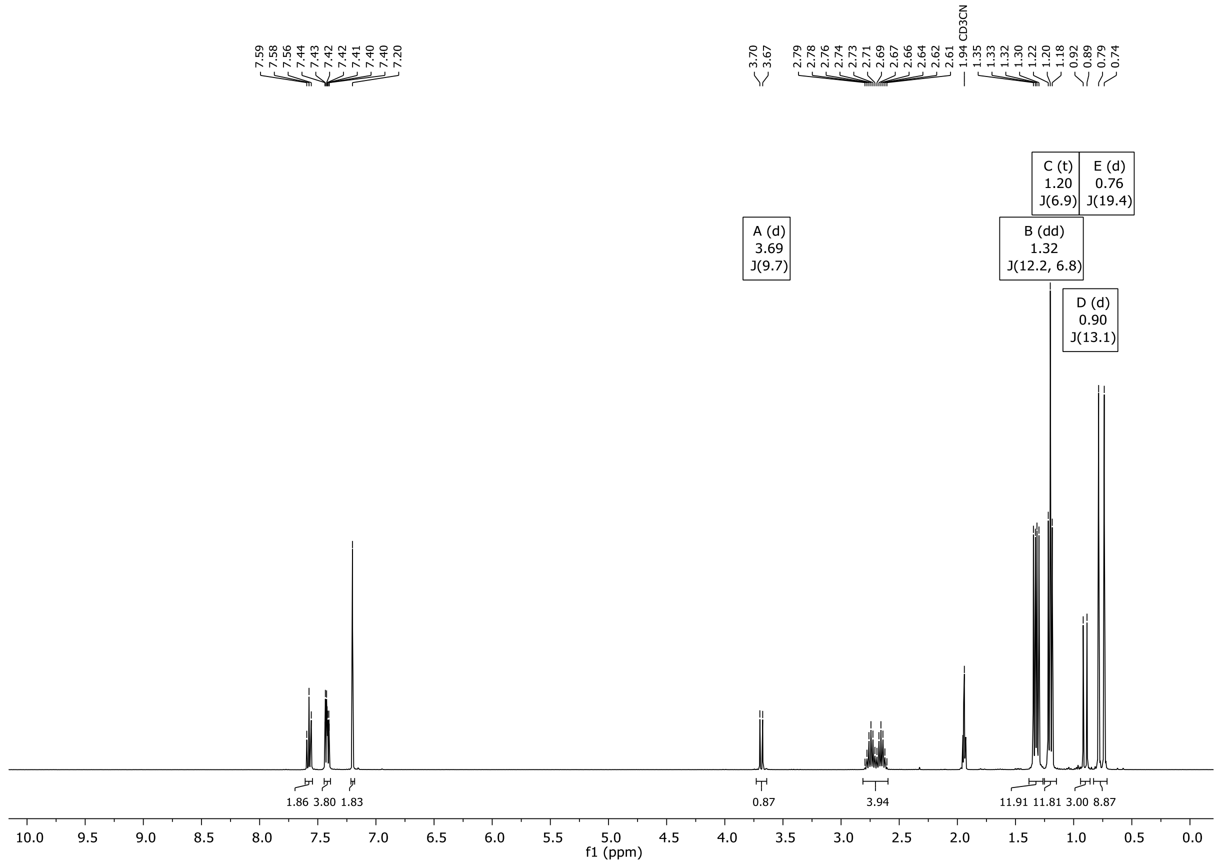

Figure S63: ^{1}H NMR spectrum (400 MHz, CD_3CN) of **9**.

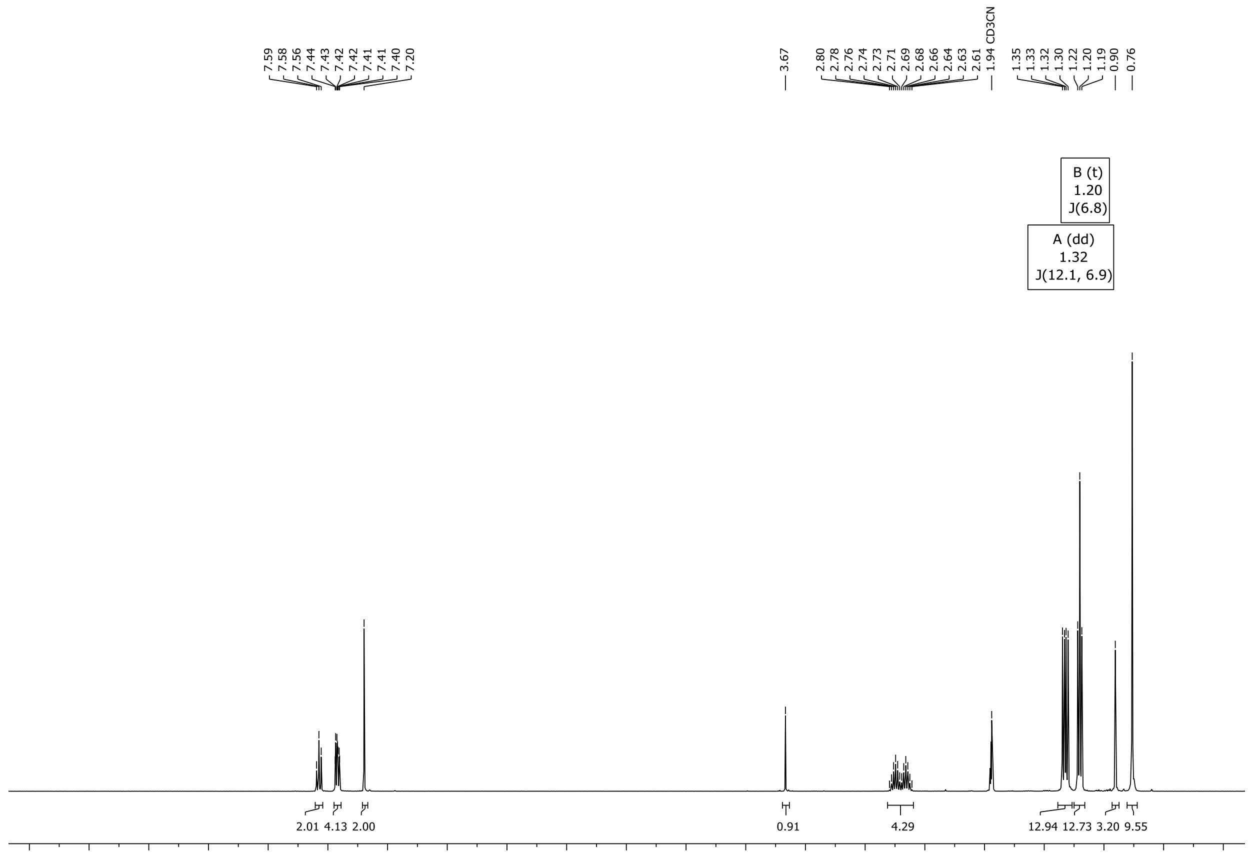

Figure S64: $^{1}H\{^{31}P\}$ NMR spectrum (400 MHz, CD_3CN) of **9**.

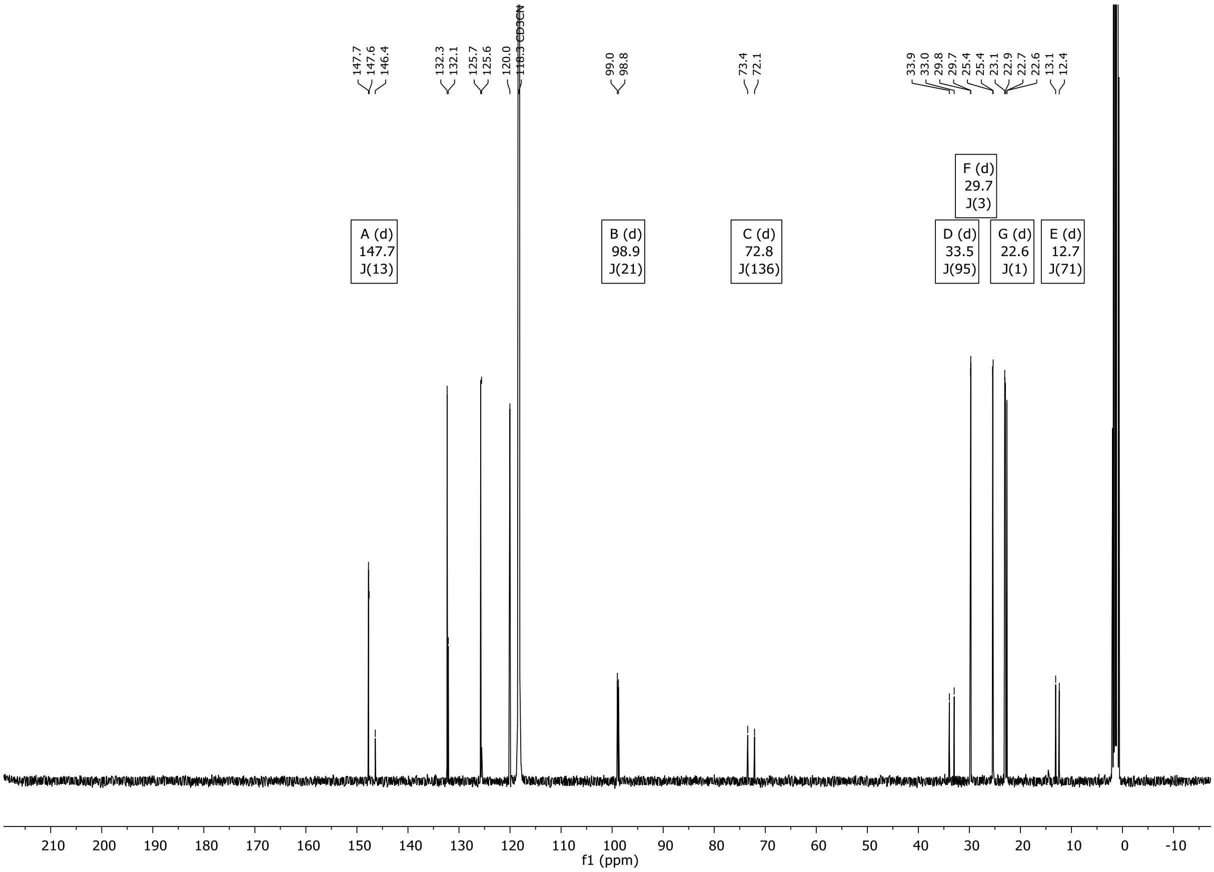

Figure S65: $^{13}C\{^1H\}$ NMR spectrum (101 MHz, CD_3CN) of **9**.

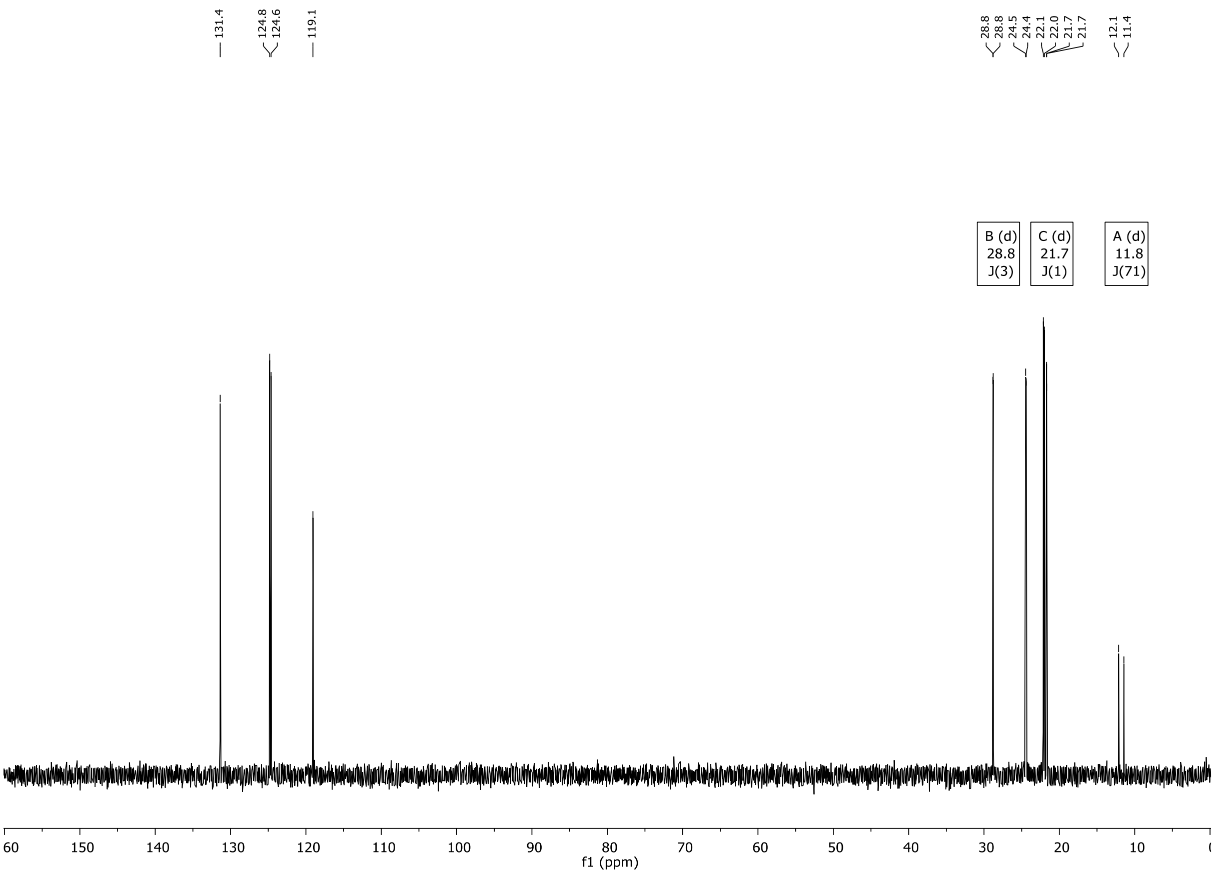

Figure S66: ^{13}C DEPT-135 spectrum (101 MHz, CD_3CN) of **9**.

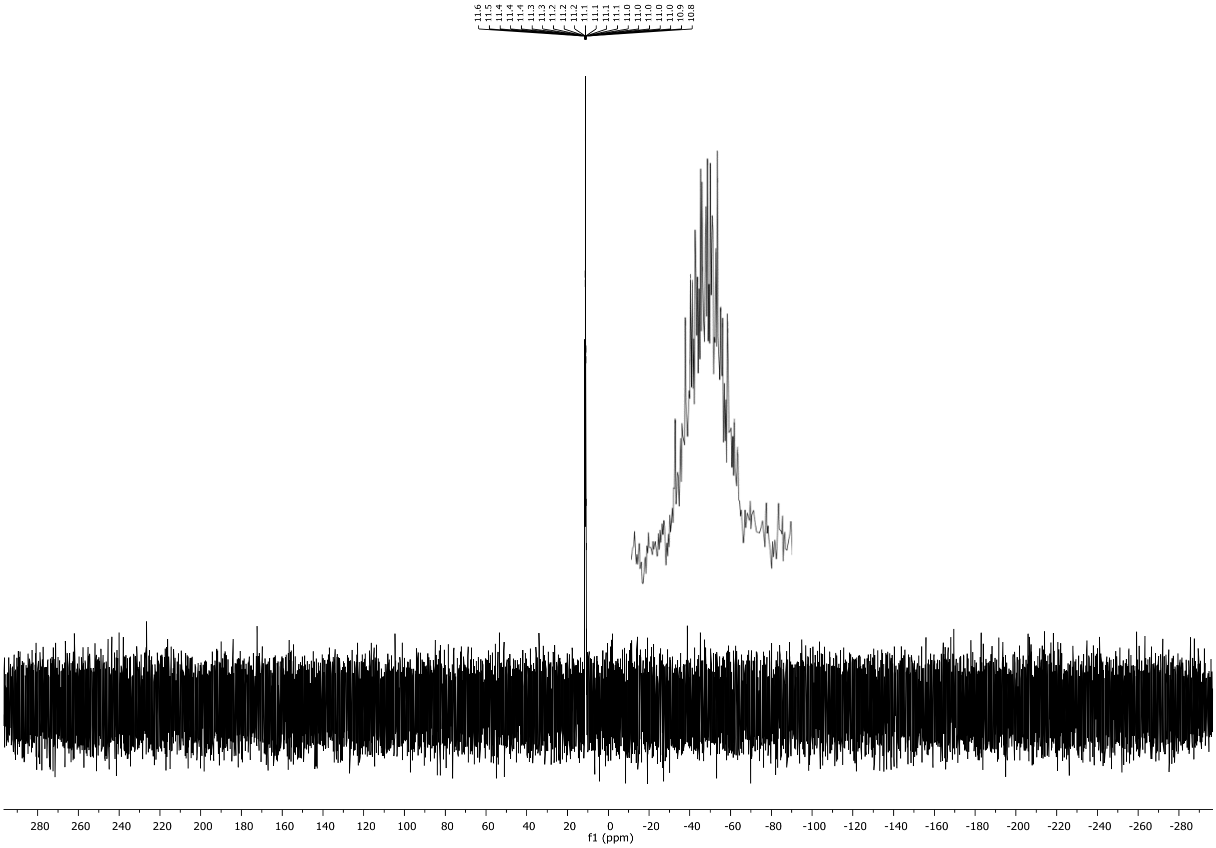

Figure S67: ^{31}P NMR spectrum (162 MHz, CD_3CN) of **9**.

Figure S68: $^{31}P\{^1H\}$ NMR spectrum (162 MHz, CD_3CN) of **9**.

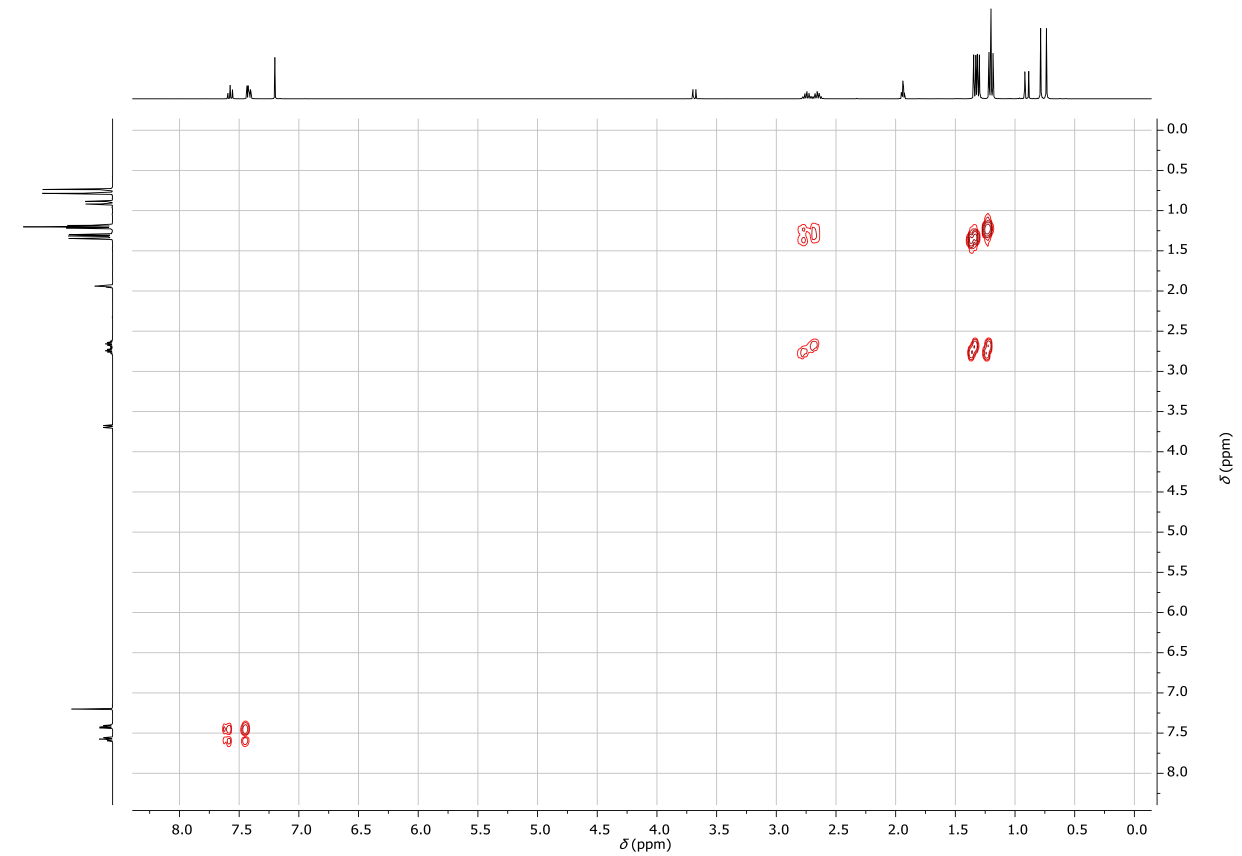

Figure S69: COSY (^{1}H, ^{1}H) NMR spectrum (400 MHz, 400 MHz, CD_3CN) of **9**.

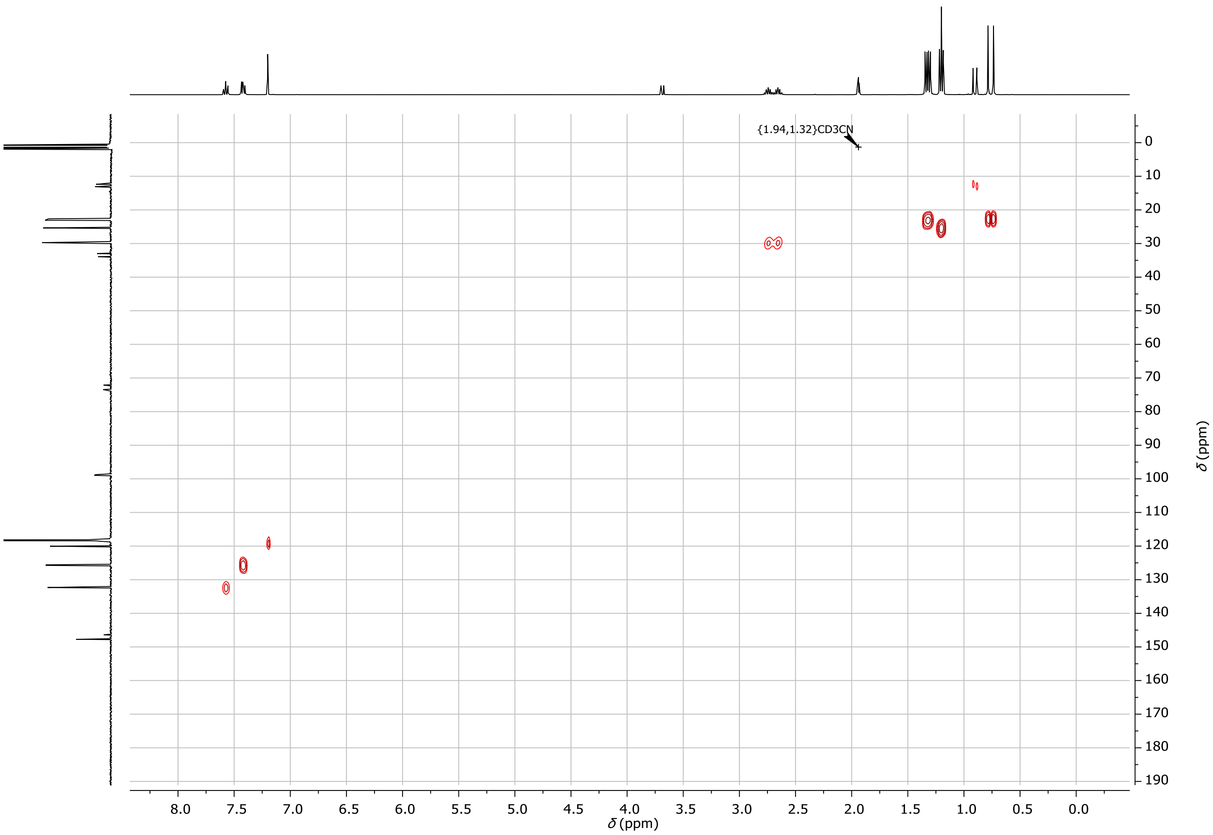

Figure S70: HSQC (^{1}H, ^{13}C) NMR spectrum (400 MHz, 101 MHz, CD_3CN) of **9**.

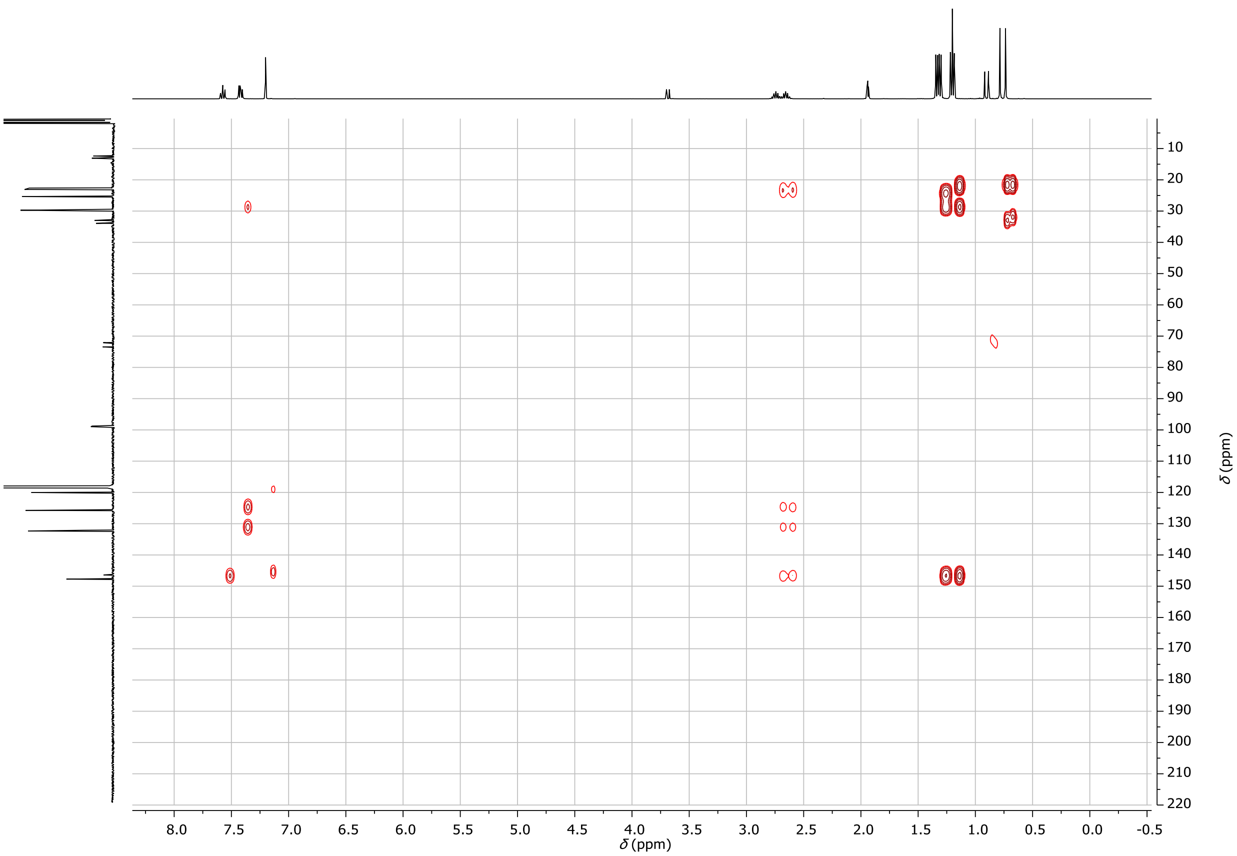

Figure S71: HMBC (^1H, ^{13}C) NMR spectrum (400 MHz, 101 MHz, CD_3CN) of **9**.

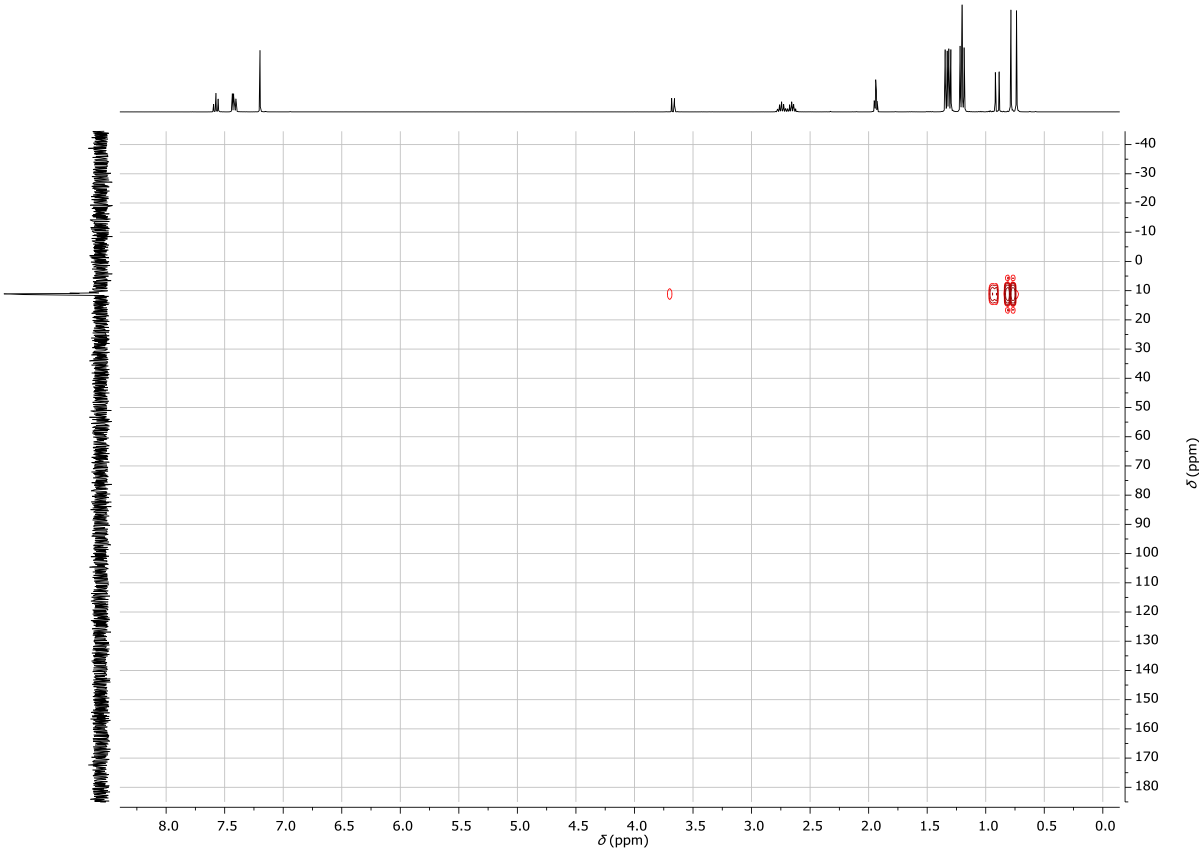

Figure S72: HMBC (^1H, ^{31}P) NMR spectrum (400 MHz, 162 MHz, CD_3CN) of **9**.

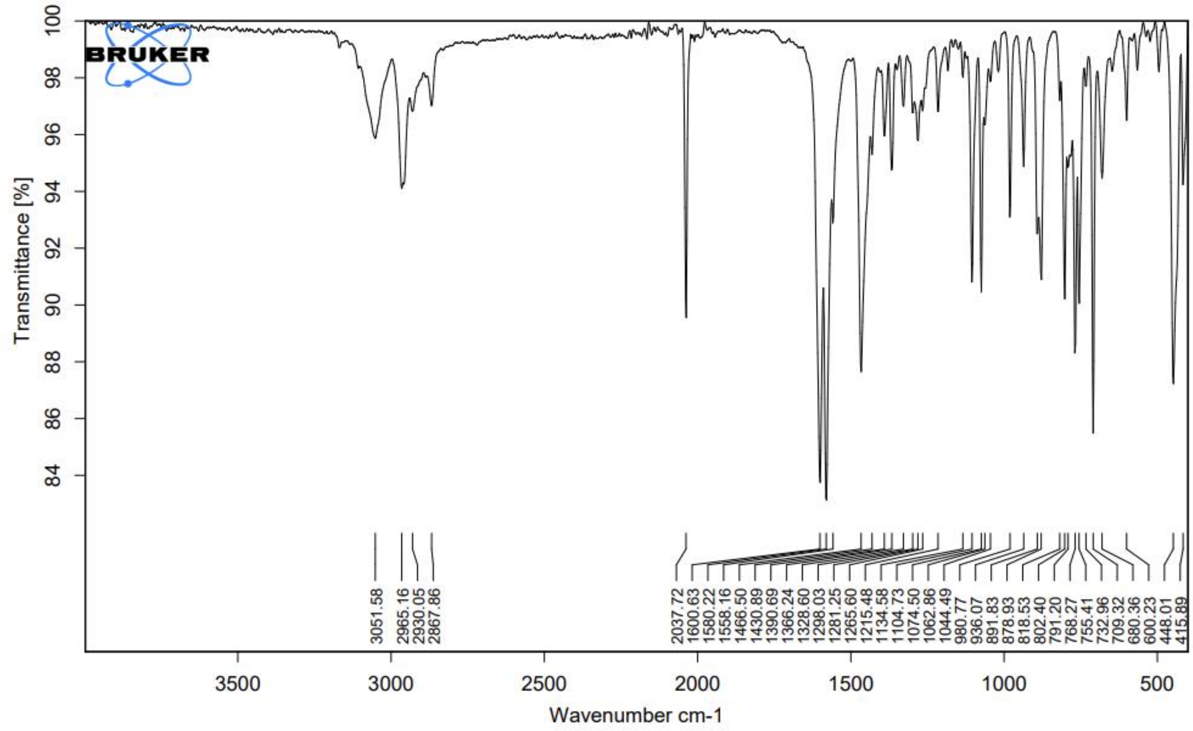

Figure S73: IR spectrum (neat) of **9**.

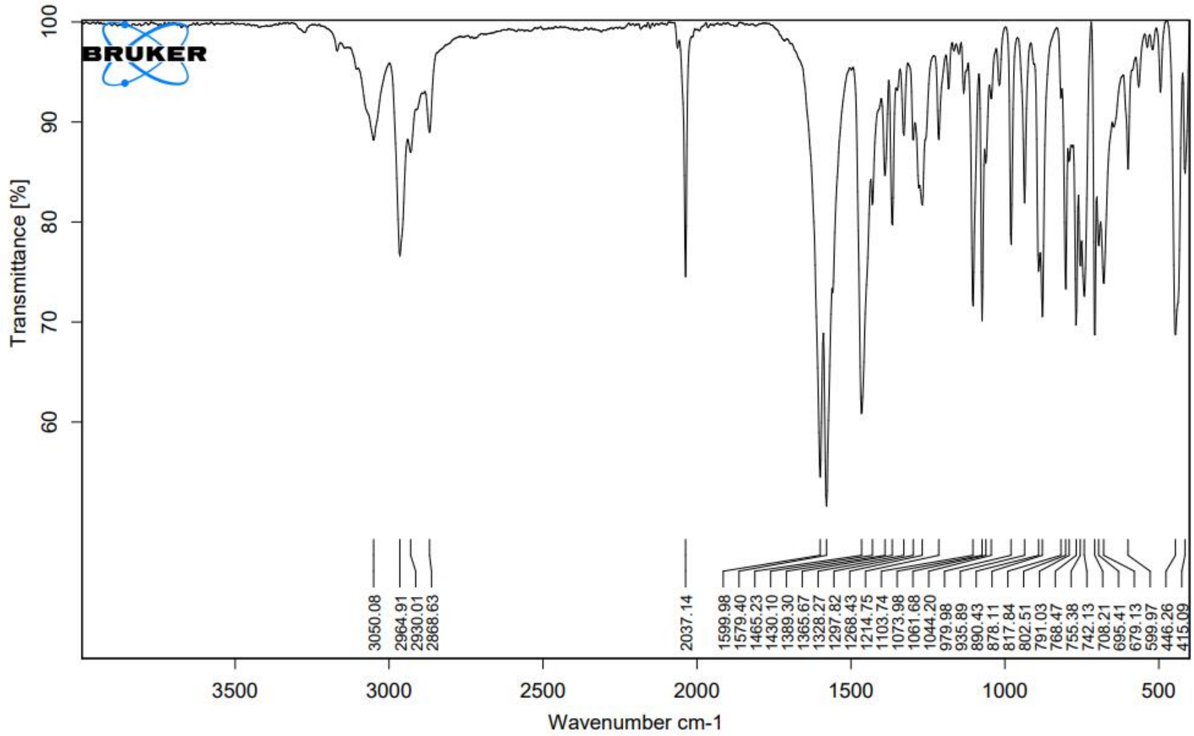

Figure S74: IR spectrum (in DCM) of **9**.

P(CC)Me(NIdipp)*t*Bu **10**

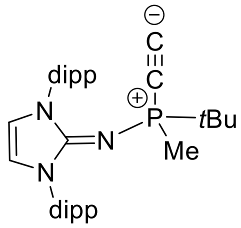

A solution of KHMDS (62 mg, 0.311 mmol, 1.02 eq.) in THF (2 mL) was added to a stirred suspension of **9** (200 mg, 0.304 mmol, 1.00 eq.) in THF (5 mL) at −40 °C. Immediately after the addition, the volatiles were removed *in vacuo* while warming up room temperature. The residue was washed with cold Et_2O (2 mL, −40 °C) to remove excess KHMDS and avoid dissolving of the product.$ The residue was extracted with toluene (3x 5 mL) to remove formed potassium iodide and the solvent was removed *in vacuo* to afford phosphonioacetylide **10** as an off-white powder (125 mg, 0.236 mmol, 78%).

$Notably, if **10** gets dissolved during washing with Et_2O, it can be conveniently recovered by recrystallization from the filtrate at −40°C.

^{1}H NMR (600 MHz, C_6D_6) δ (ppm) = 7.20 - 7.07 (m, 6H, Ar-H), 6.19 (s, 2H, NCHCHN), 3.38 (hept, $^3J_{HH}$ = 6.8 Hz, 2H, CH (*i*Pr)), 3.09 (hept, $^3J_{HH}$ = 6.8 Hz, 2H, CH (*i*Pr)), 1.60 (d, $^3J_{HH}$ = 6.7 Hz, 6H, CH_3 (*i*Pr)), 1.48 (d, $^3J_{HH}$ = 6.7 Hz, 6H, CH_3 (*i*Pr)), 1.09 (d, $^3J_{HH}$ = 6.9 Hz, 6H, CH_3 (*i*Pr)), 1.07 (d, $^3J_{HH}$ = 7.0 Hz, 6H, CH_3 (*i*Pr)), 0.75 (d, $^2J_{HP}$ = 17.1 Hz, 9H, CH_3 (*t*Bu)), 0.31 ((d, $^3J_{PH}$ = 12.6 Hz, 3H, CH_3 (Me)).

^{1}H{^{31}P} NMR (400 MHz, C_6D_6) δ (ppm) = 7.20 - 7.06 (m, 6H, Ar-H), 6.19 (s, 2H, NCHCHN), 3.38 (hept, $^3J_{HH}$ = 6.8 Hz, 2H, CH (*i*Pr)), 3.09 (hept, $^3J_{HH}$ = 6.9 Hz, 2H, CH (*i*Pr)), 1.60 (d, $^3J_{HH}$ = 6.7 Hz, 6H, CH_3 (*i*Pr)), 1.48 (d, $^3J_{HH}$ = 6.7 Hz, 6H, CH_3 (*i*Pr)), 1.09 (d, $^3J_{HH}$ = 6.9 Hz, 6H, CH_3 (*i*Pr)), 1.07 (d, $^3J_{HH}$ = 6.9 Hz, 6H, CH_3 (*i*Pr)), 0.75 (s, 9H, CH_3 (*t*Bu)), 0.31 ((s, 3H, CH_3 (Me)).

^{13}C{^{1}H} NMR (151 MHz, C_6D_6) δ (ppm) = 231.8 (d, $^2J_{CP}$ = 17 Hz, βC (CC)), 149.3 (s, q-C (Ar-*i*Pr)), 147.9 (d, $^2J_{CP}$ = 42 Hz, NCN), 132.9 (s, ipso-C (Ar-H)), 130.17 (s, C (Ar)), 128.3 (s, C (Ar-H)), 124.7 (s, C (Ar-H)), 124.5 (s, C (Ar-H)), 117.3 (s, NCHCHN), 90.8 (d, $^1J_{CP}$ = 112 Hz, αC (P-CC)), 32.4 (d, $^1J_{CP}$ = 94 Hz, q-C (*t*Bu)), 29.1 (s, CH (*i*Pr)), 28.9 (s, CH (*i*Pr)), 25.8 (s, CH_3 (*i*Pr)), 25.6 (s, CH_3 (*i*Pr)), 23.9 (s, CH_3 (*i*Pr)), 23.6 (d, $^2J_{CP}$ = 3 Hz, CH_3 (*t*Bu)), 23.4 (s, CH_3 (*i*Pr)), 13.5 (d, $^1J_{CP}$ = 72 Hz, CH_3 (Me)).

^{31}P NMR (162 MHz, C_6D_6) δ (ppm) = −2.4 - −2.8 (m).

^{31}P{^{1}H} NMR (162 MHz, C_6D_6) δ (ppm) = −2.6 (s).

HRMS (ESI, THF) m/z = 530.3650 ([M+H]$^+$, calculated: 530.3659).

IR (neat): ν_{max}/cm^{-1} = 2961.83, 2927.21, 2867.71, 1936.67 (C≡C), 1606.24, 1582.94, 1562.32, 1464.09, 1418.98, 1386.58, 1363.70, 1328.92, 1287.94, 1256.79, 1125.55, 1098.21, 1080.38, 1064.43, 1041.49, 1015.08, 967.43, 932.12, 887.29, 873.67, 813.38, 768.83, 732.39, 712.48, 696.07, 623.23, 601.05, 439.68, 411.63.

IR (in DCM): ν_{max}/cm^{-1} = 2966.69, 2928.80, 2869.53, 1952.10 (C≡C), 1605.08, 1582.91, 1567.76, 1466.97, 1421.91, 1386.61, 1364.83, 1275.19, 1258.05, 1210.33, 1129.26, 1091.51, 1064.31, 1040.89, 967.77, 933.22, 889.44, 876.13, 805.38, 770.97, 764.13, 739.55, 722.82, 712.96, 691.56, 602.96, 439.26, 416.89.

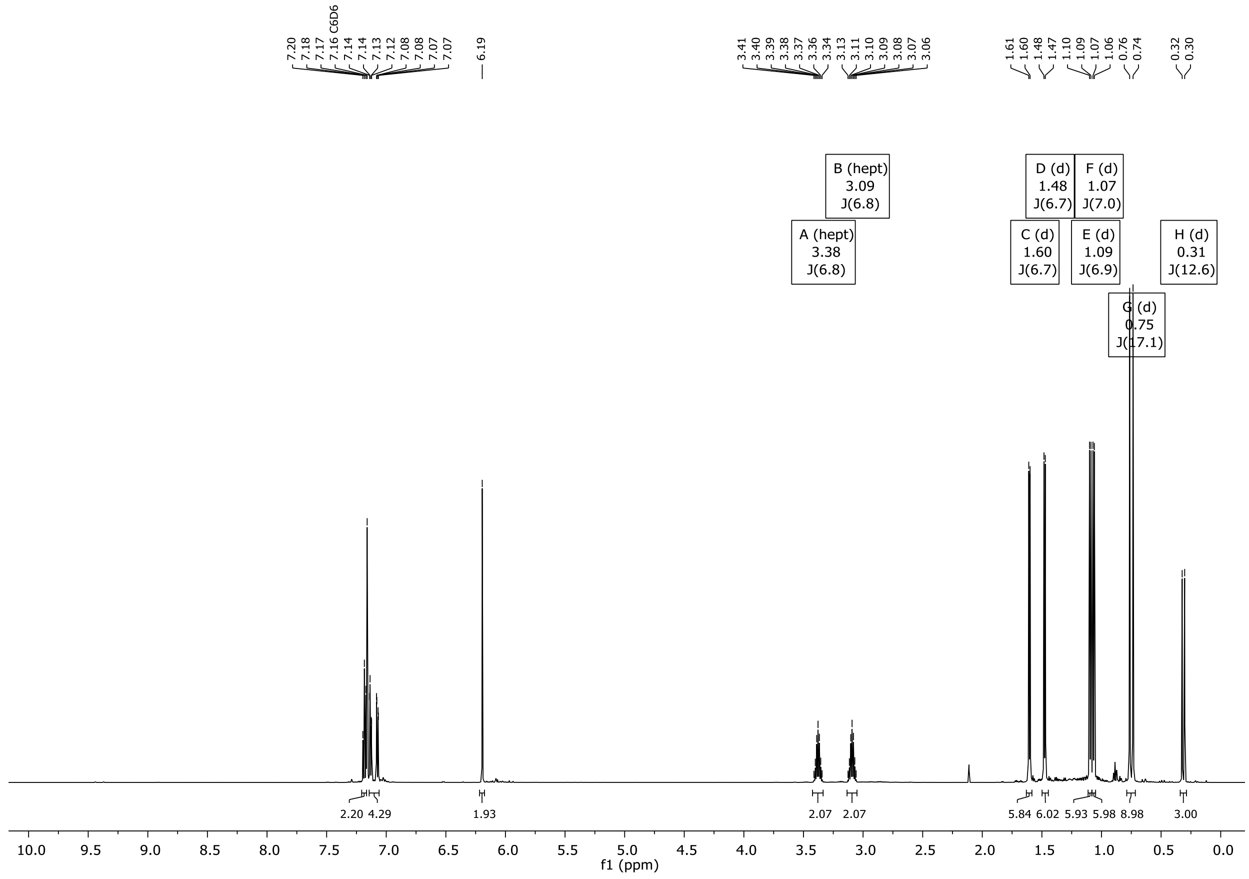

Figure S75: ^{1}H NMR spectrum (600 MHz, C_6D_6) of **10**.

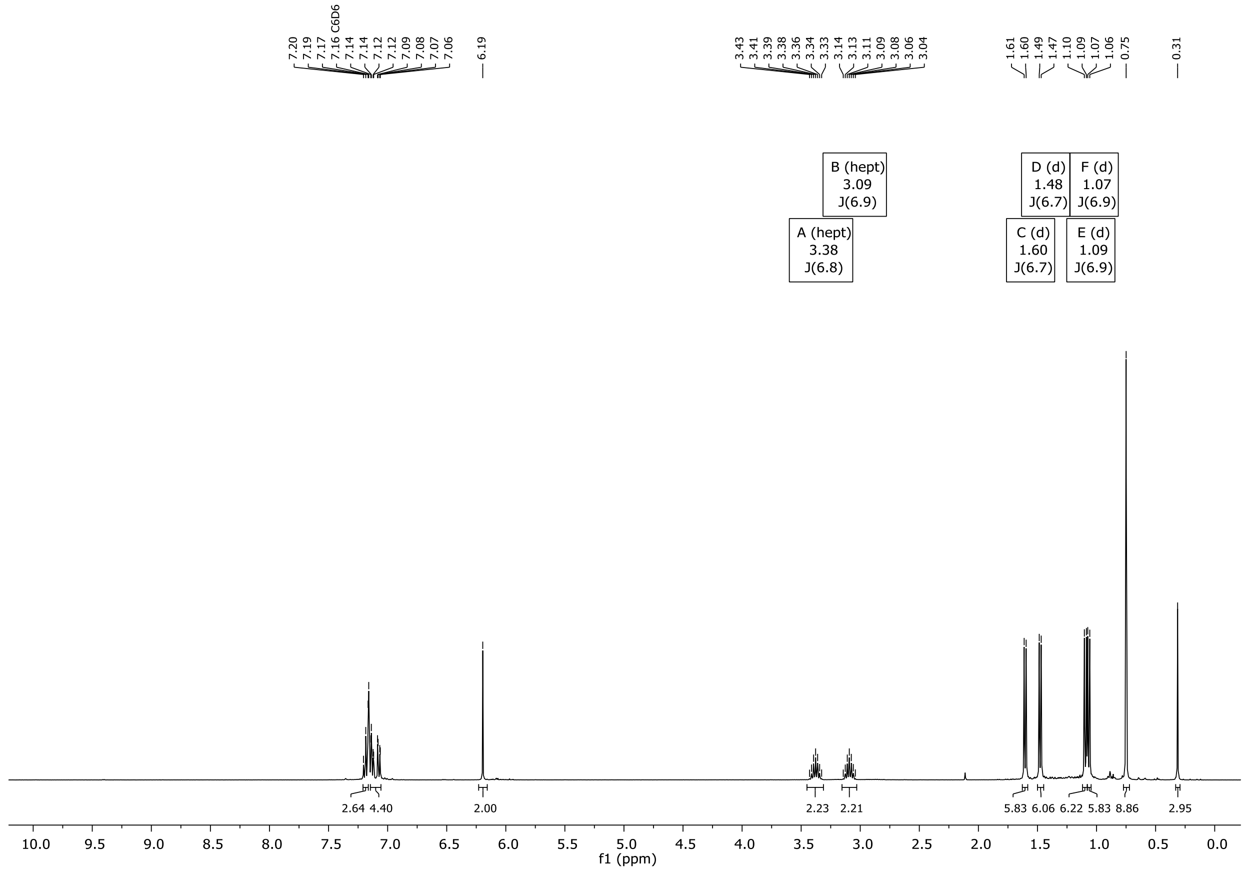

Figure S76: ^{1}H{^{31}P} NMR spectrum (400 MHz, C_6D_6) of **10**.

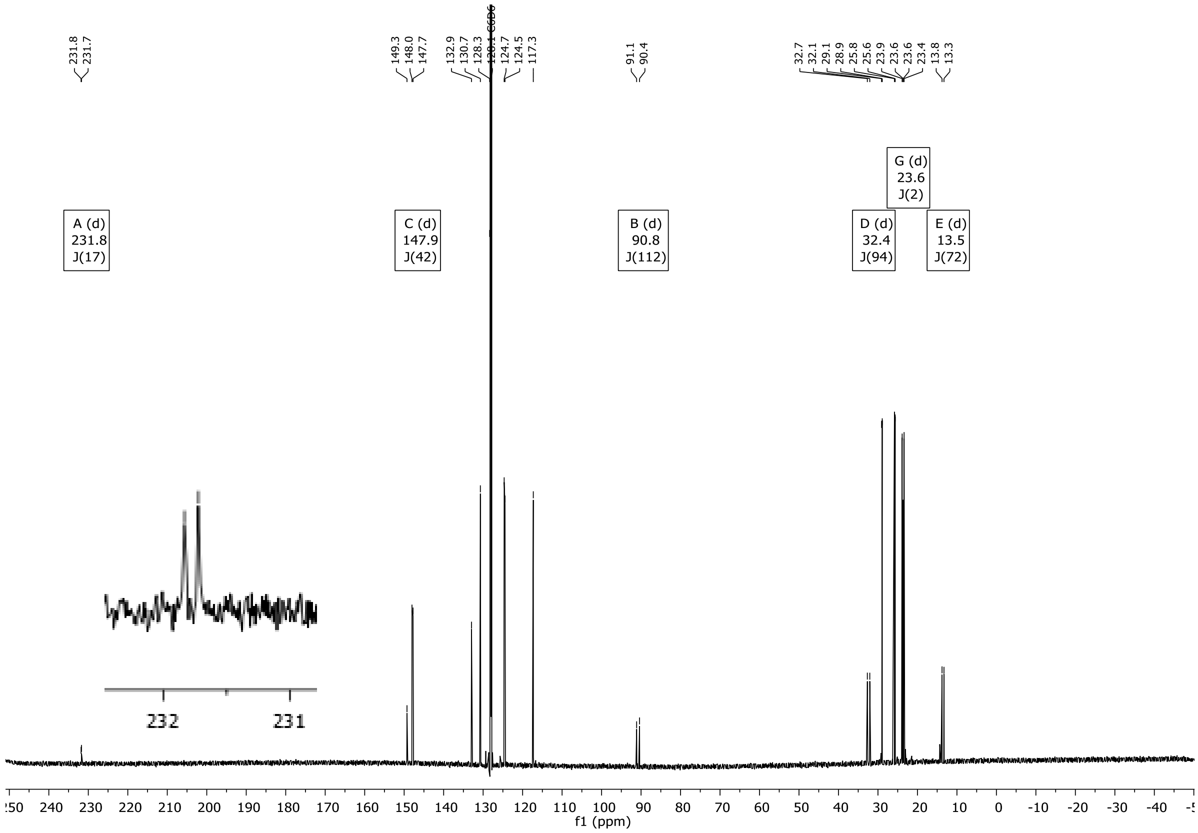

Figure S77: $^{13}C\{^{1}H\}$ NMR spectrum (151 MHz, C_6D_6) of **10**.

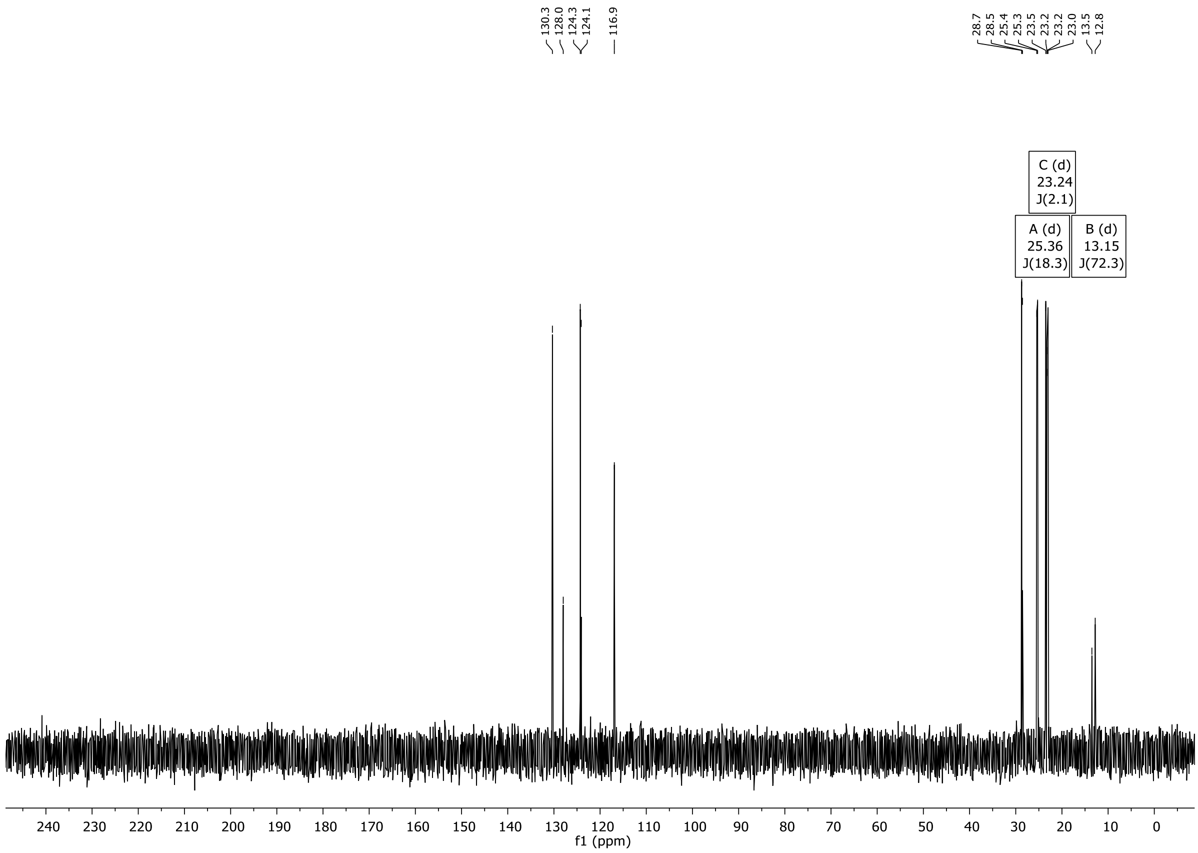

Figure S78: ^{13}C DEPT-135 spectrum (101 MHz, C_6D_6) of **10**.

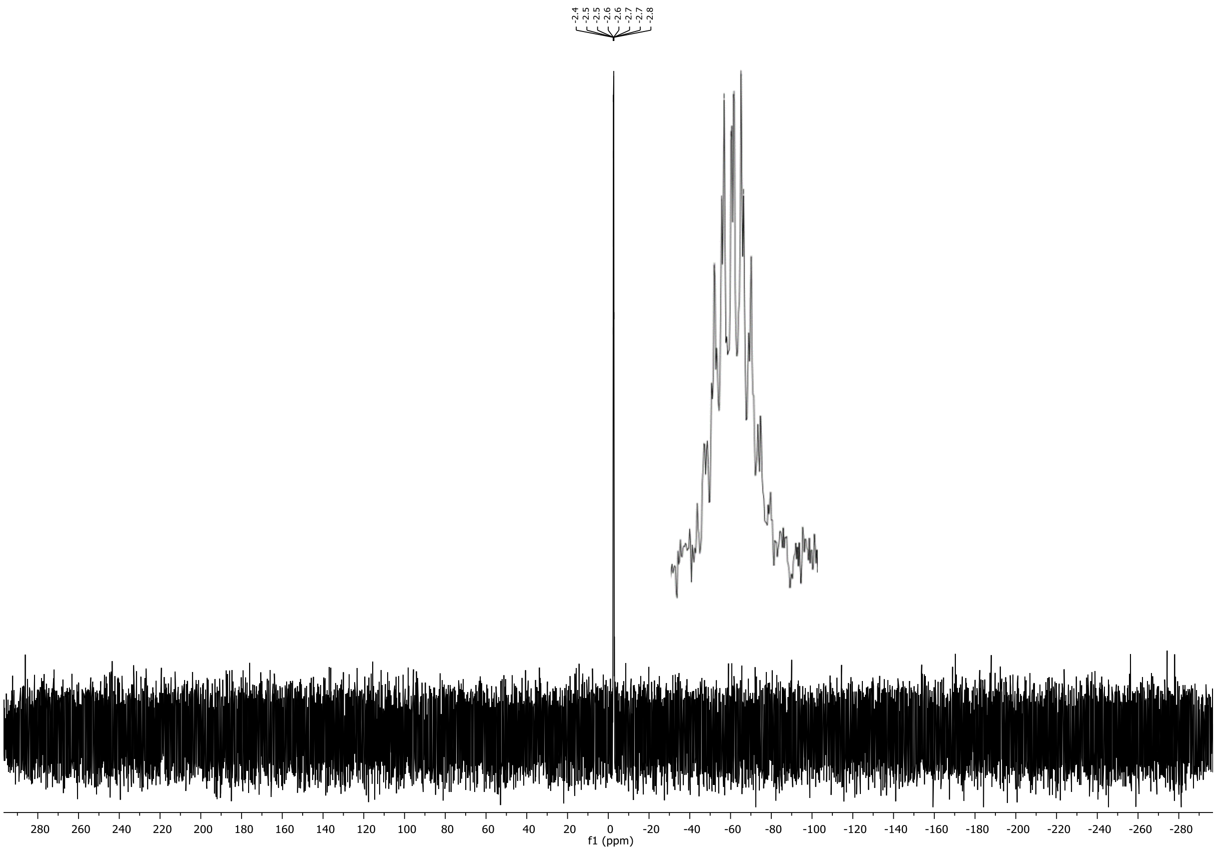

Figure S79: ^{31}P NMR spectrum (162 MHz, C_6D_6) of **10**.

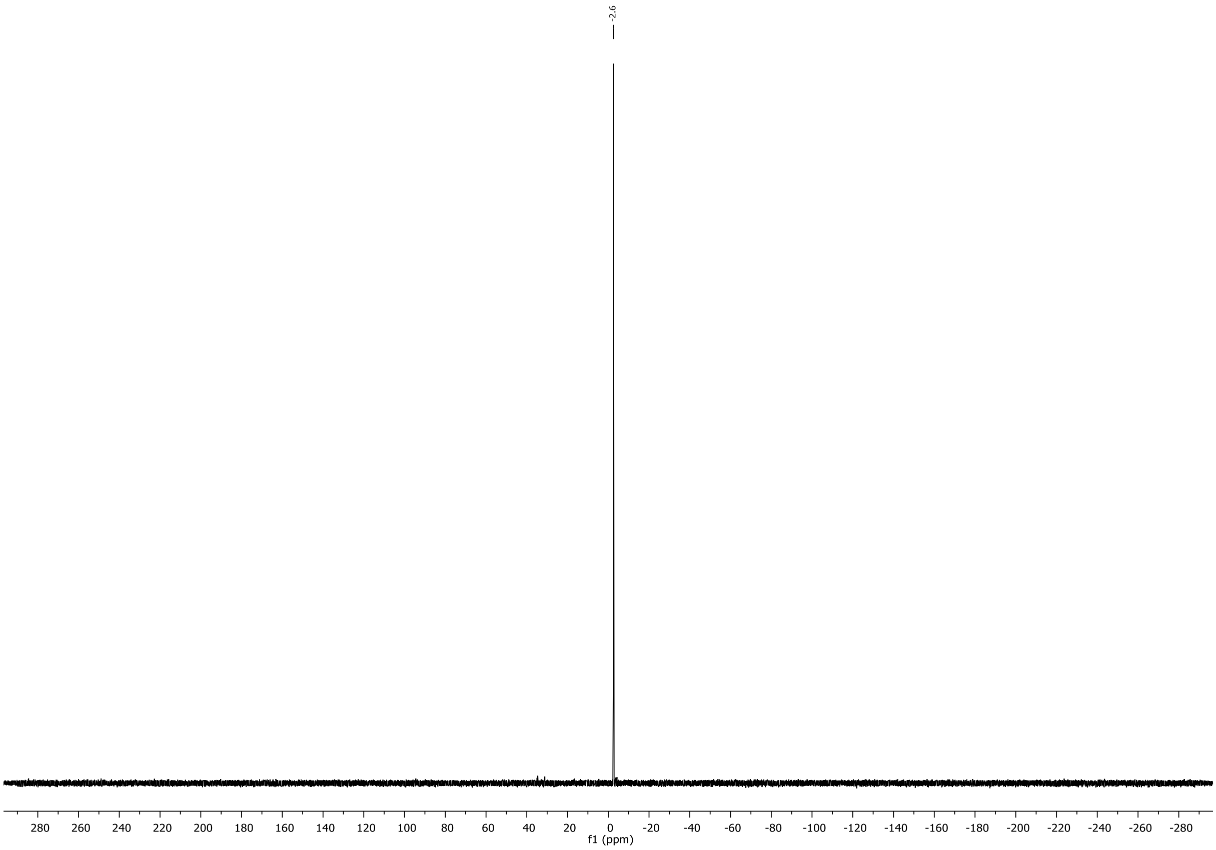

Figure S80: $^{31}P\{^1H\}$ NMR spectrum (162 MHz, C_6D_6) of **10**.

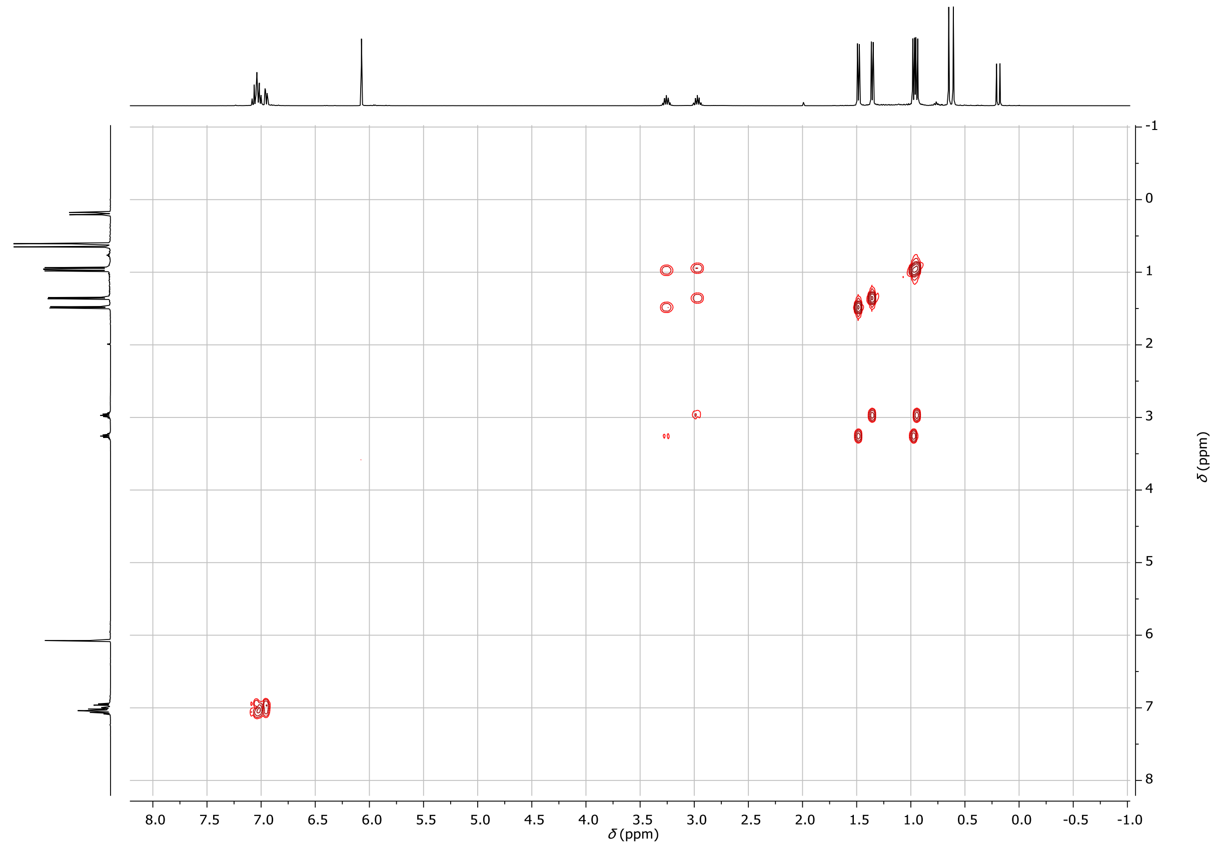

Figure S81: COSY (^{1}H, ^{1}H) NMR spectrum (400 MHz, 400 MHz, C_6D_6) of **10**.

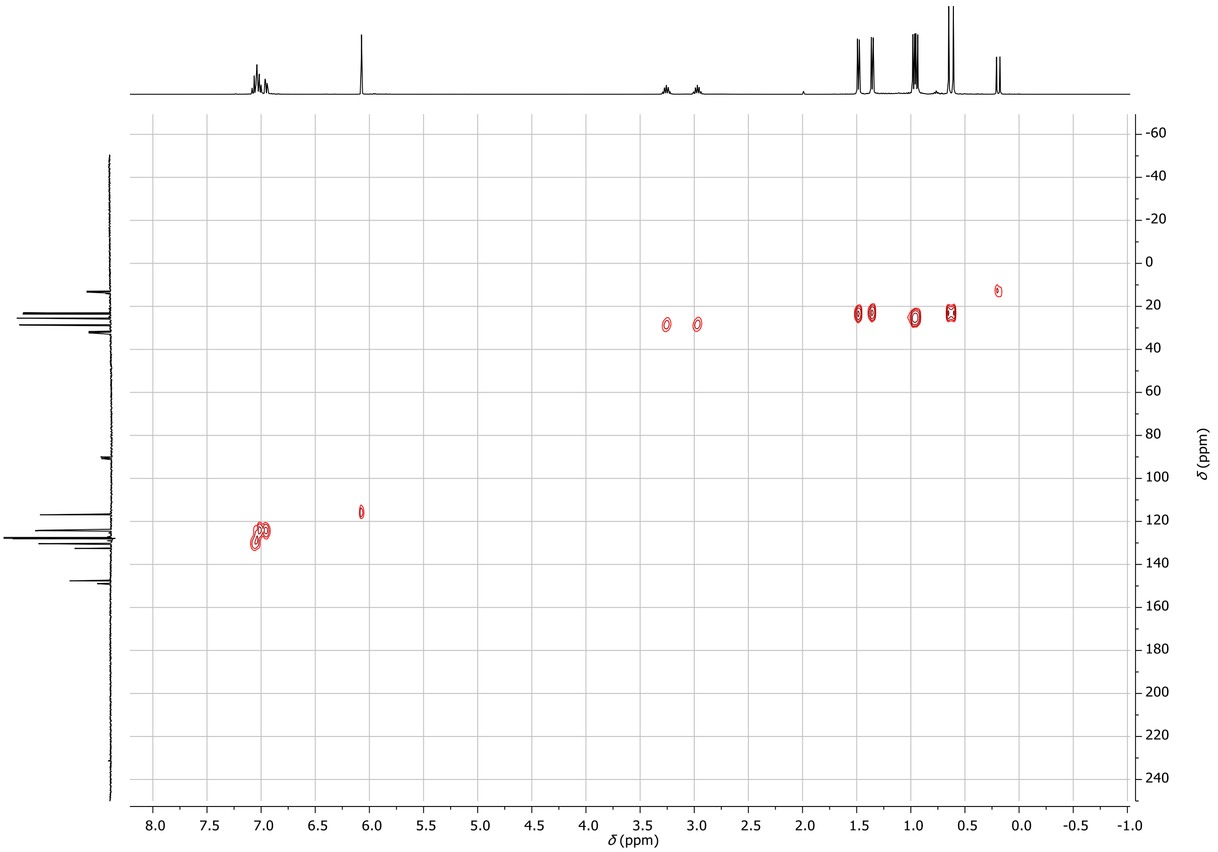

Figure S82: HSQC (^{1}H, ^{13}C) NMR spectrum (400 MHz, 101 MHz, C_6D_6) of **10**.

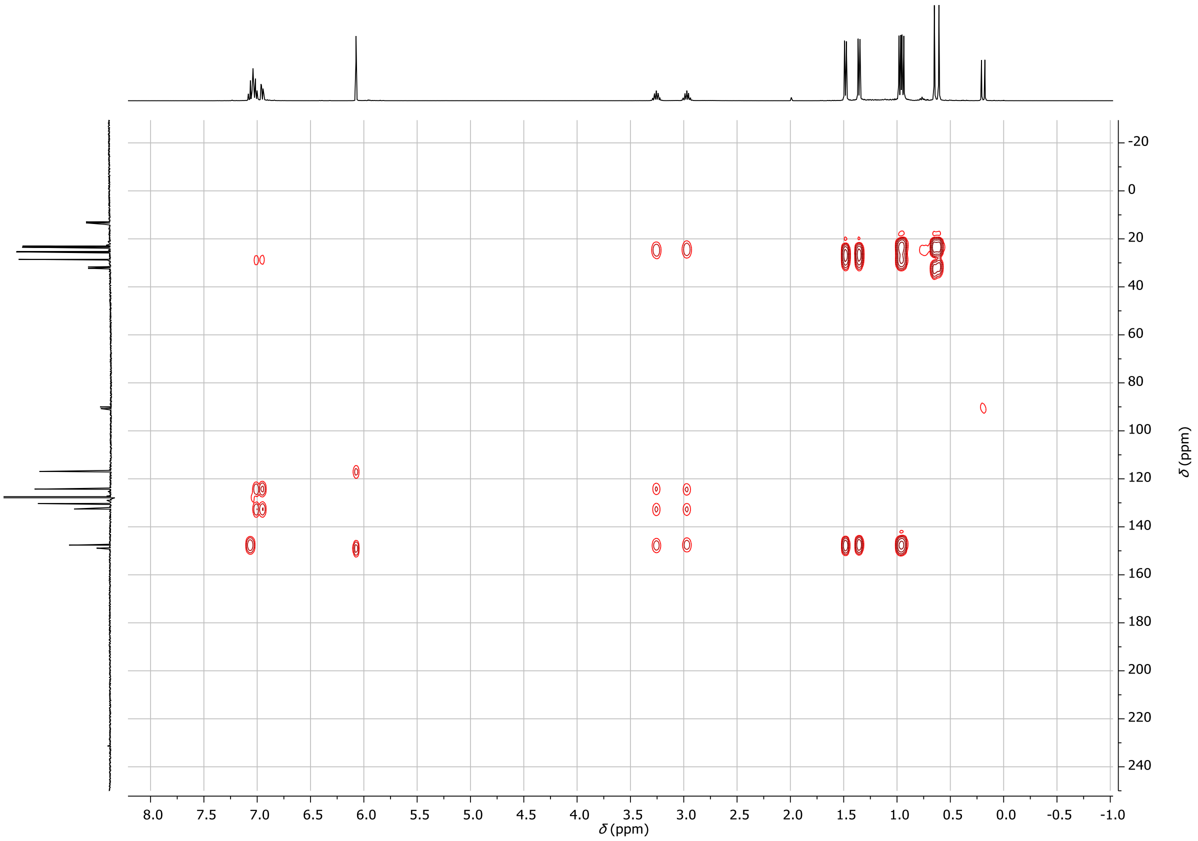

Figure S83: HMBC (^{1}H, ^{13}C) NMR spectrum (400 MHz, 101 MHz, C_6D_6) of **10**.

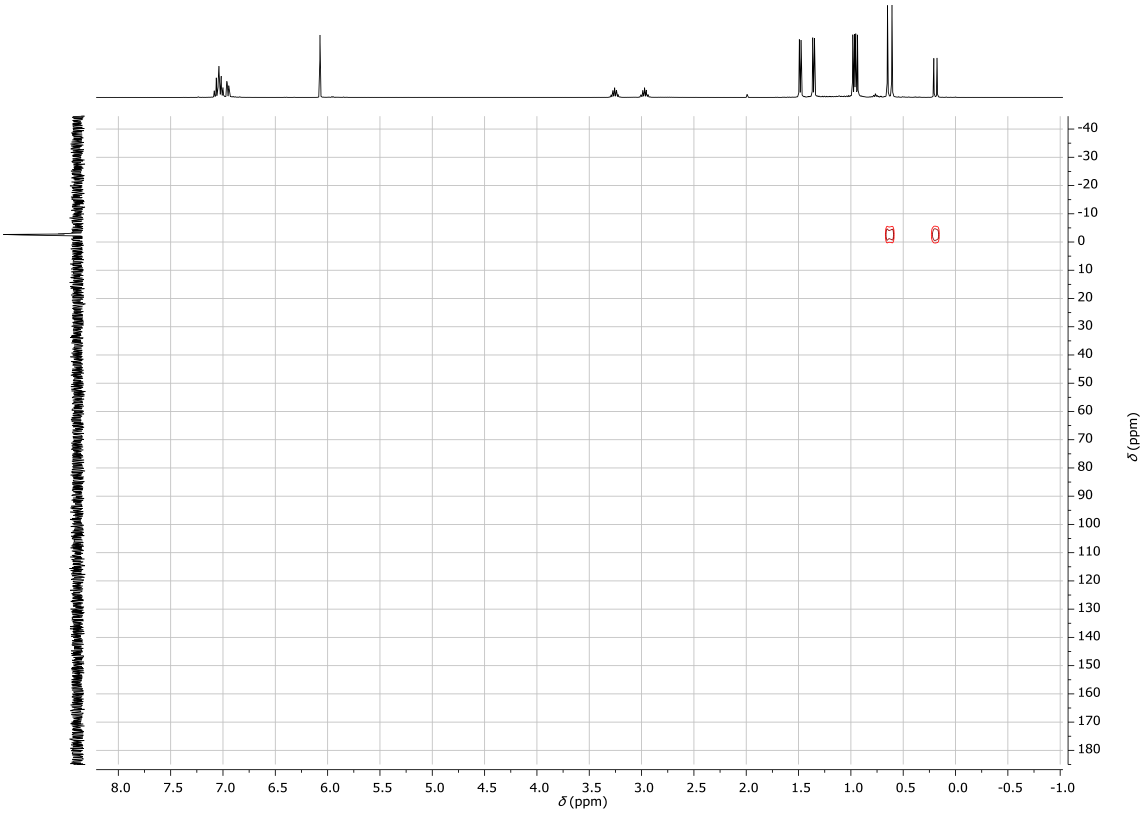

Figure S84: HMBC (^{1}H, ^{31}P) NMR spectrum (400 MHz, 162 MHz, C_6D_6) of **10**.

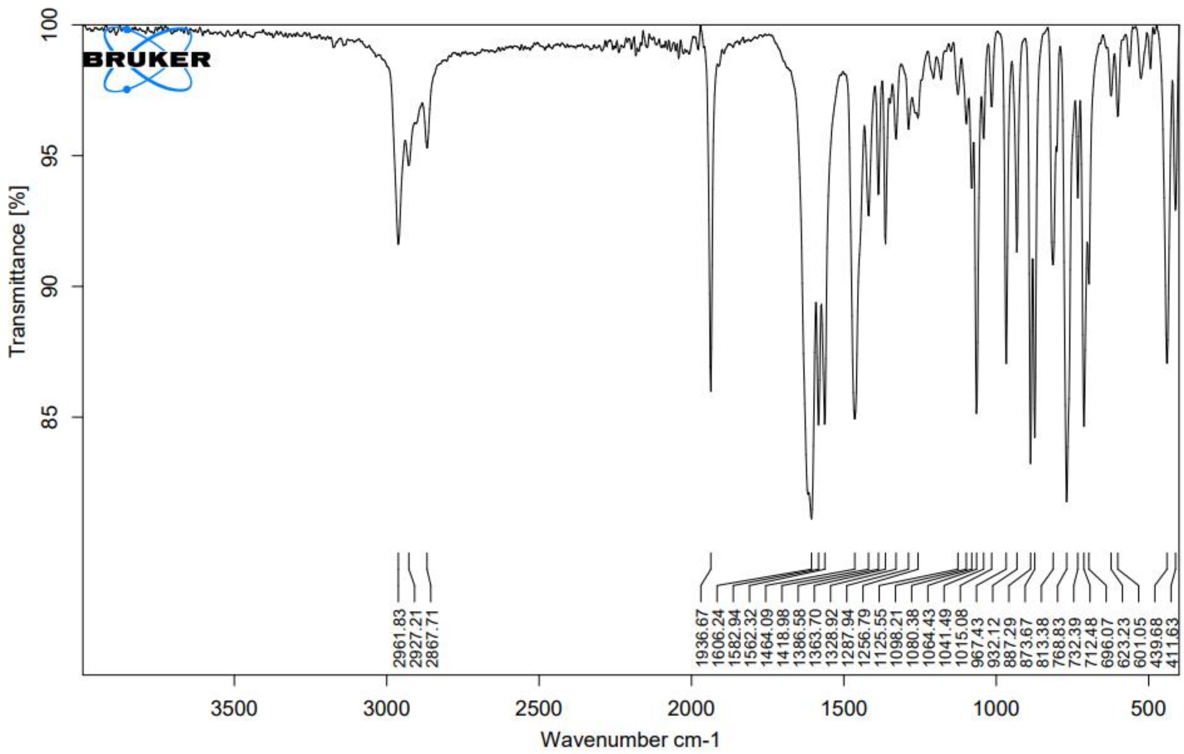

Figure S85: IR spectrum (neat) of **10**.

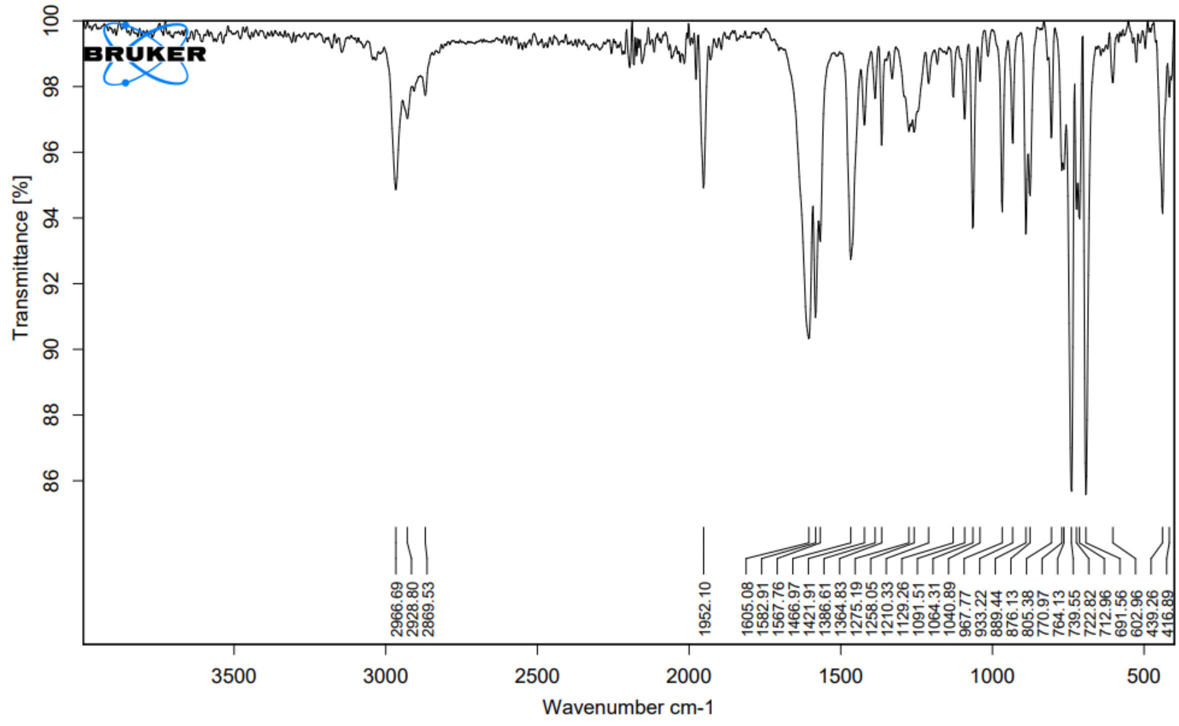

Figure S86: IR spectrum (in DCM) of **10**.

PMe(NIdipp)tBu **11**

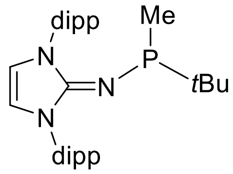

Methylmagnesium chloride in THF (0.07 mL, 3.0 M, 0.210 mmol, 1.10 eq.) was added dropwise to **7** (100 mg, 0.190 mmol, 1.00 eq.) in THF (5 mL) at −78 °C. The reaction mixture was allowed to warm to room temperature while stirring over 16 h. All volatile compounds were removed *in vacuo*. The residue was extracted with *n*-hexane (2x 25 mL). All volatiles were removed *in vacuo* and the product **11** was received as a white solid (50 mg, 0.099 mmol, 52%).

^{1}H NMR (400 MHz, C_6D_6) δ (ppm) = 7.26 - 7.22 (m, 2H, Ar-H (para)), 7.17 - 7.11 (m, 4H, Ar-H (meta)), 5.96 (s, 2H, NCHCHN), 3.42 (sept, $^{3}J_{HH}$ = 6.9 Hz, 2H, CH (*i*Pr)), 3.07 (sept, $^{3}J_{HH}$ = 7.0 Hz, 2H, CH (*i*Pr)), 1.52 (d, $^{3}J_{HH}$ = 6.9 Hz, 6H, CH_3 (*i*Pr)), 1.36 (d, $^{3}J_{HH}$ = 6.9 Hz, 6H, CH_3 (*i*Pr)), 1.19 (dd, $^{3}J_{HH}$ = 7.9 Hz, $^{3}J_{HH}$ = 6.9 Hz, 12H, CH_3 (*i*Pr)), 0.84 (d, $^{3}J_{HP}$ = 11.7 Hz, 9H, CH_3 (*t*Bu)), 0.51 (d, $^{3}J_{HP}$ = 5.2 Hz, 3H, CH_3 (Me)).

^{1}H{^{31}P} NMR (400 MHz, C_6D_6) δ (ppm) = 7.26 - 7.22 (m, 2H, Ar-H (para)), 7.17 - 7.10 (m, 4H, Ar-H (meta)), 5.97 (s, 2H, NCHCHN), 3.42 (sept, $^{3}J_{HH}$ = 6.9 Hz, 2H, CH (*i*Pr)), 3.07 (sept, $^{3}J_{HH}$ = 7.0 Hz, 2H, CH (*i*Pr)), 1.52 (d, $^{3}J_{HH}$ = 6.8 Hz, 6H, CH_3 (*i*Pr)), 1.36 (d, $^{3}J_{HH}$ = 6.9 Hz, 6H, CH_3 (*i*Pr)), 1.19 (dd, $^{3}J_{HH}$ = 7.9 Hz, $^{3}J_{HH}$ = 6.9 Hz, 12H, CH_3 (*i*Pr)), 0.84 (s, 9H, CH_3 (*t*Bu)), 0.51 (s, 3H, CH_3 (Me)).

^{13}C{^{1}H} NMR (C_6D_6, 101 MHz) δ (ppm) = 148.9 (s, q-C (Ar- *i*Pr)), 148.9 (s, ipso-C (Ar-H)), 147.3 (s, q-C (Ar-*i*Pr)), 147.0 (d, $^{2}J_{CP}$ = 16 Hz, NCN), 135.1, (s, ortho-C (Ar))), 129.6 (s, para-C (Ar-H)), 123.8 (s, meta-C (Ar-H)), 115.1 (s, NCHCHN), 30.4 (d, $^{1}J_{CP}$ = 6 Hz, q-C (*t*Bu)), 29.4 (s, CH (*i*Pr)), 29.4 (s, CH (*i*Pr)), 28.9 (s, CH (*i*Pr)), 25.7 (s, $^{2}J_{CP}$ = 16 Hz, CH_3 (*t*Bu)), 25.2 (s, CH_3 (*i*Pr)), 25.0 (s, CH_3 (*i*Pr)), 23.3 (s, CH_3 (*i*Pr)), 22.6 (s, CH_3 (*i*Pr)), 22.6 (s, CH_3 (*i*Pr)), 14.2 (d, $^{1}J_{CP}$ = 13 Hz, CH_3 (Me)).

^{31}P NMR (C_6D_6, 162 MHz) δ (ppm) = 42.1 (m).

^{31}P{^{1}H} NMR (162 MHz, C_6D_6) δ (ppm) = 42.1 (s).

HRMS (ESI, CH_3CN) m/z = 506.3655 ($[M+H]^+$, calculated: 506.3659).

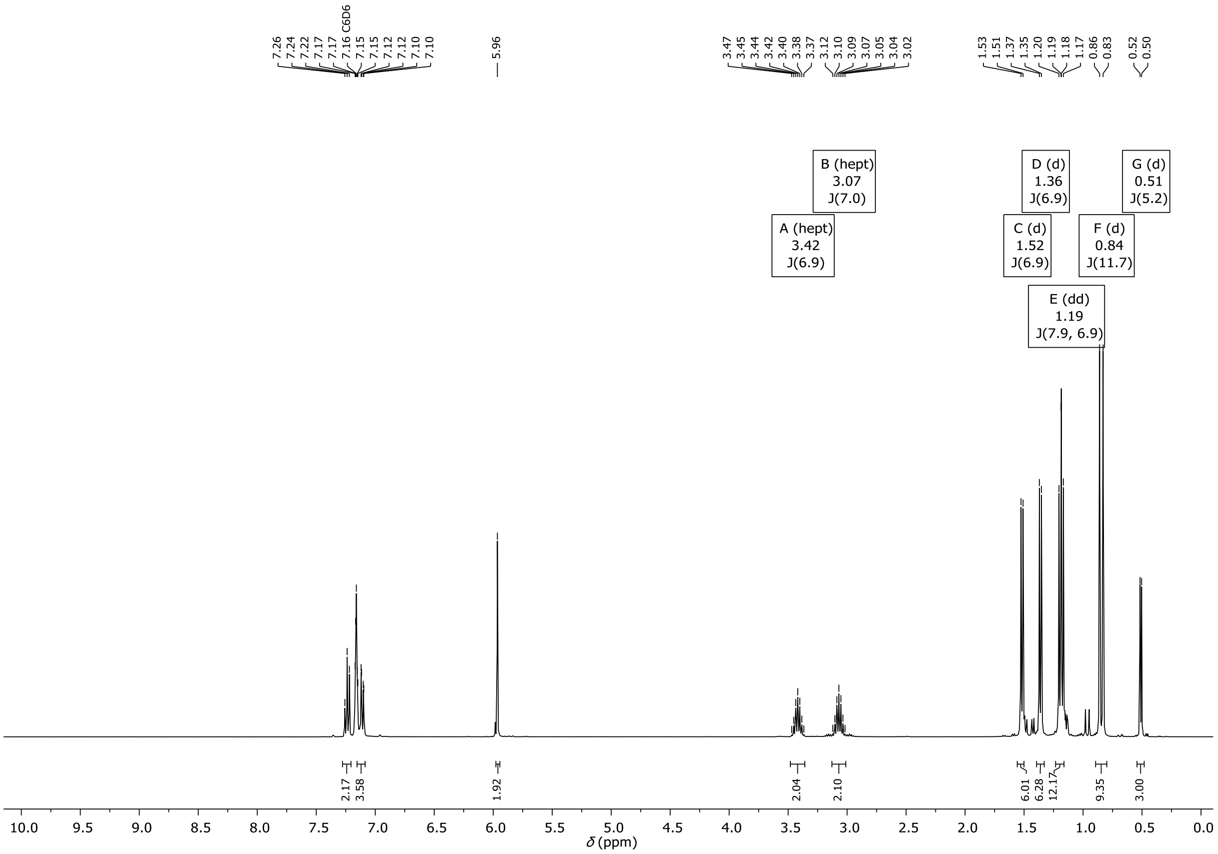

Figure S87: ^1H NMR spectrum (400 MHz, C_6D_6) of **11**.

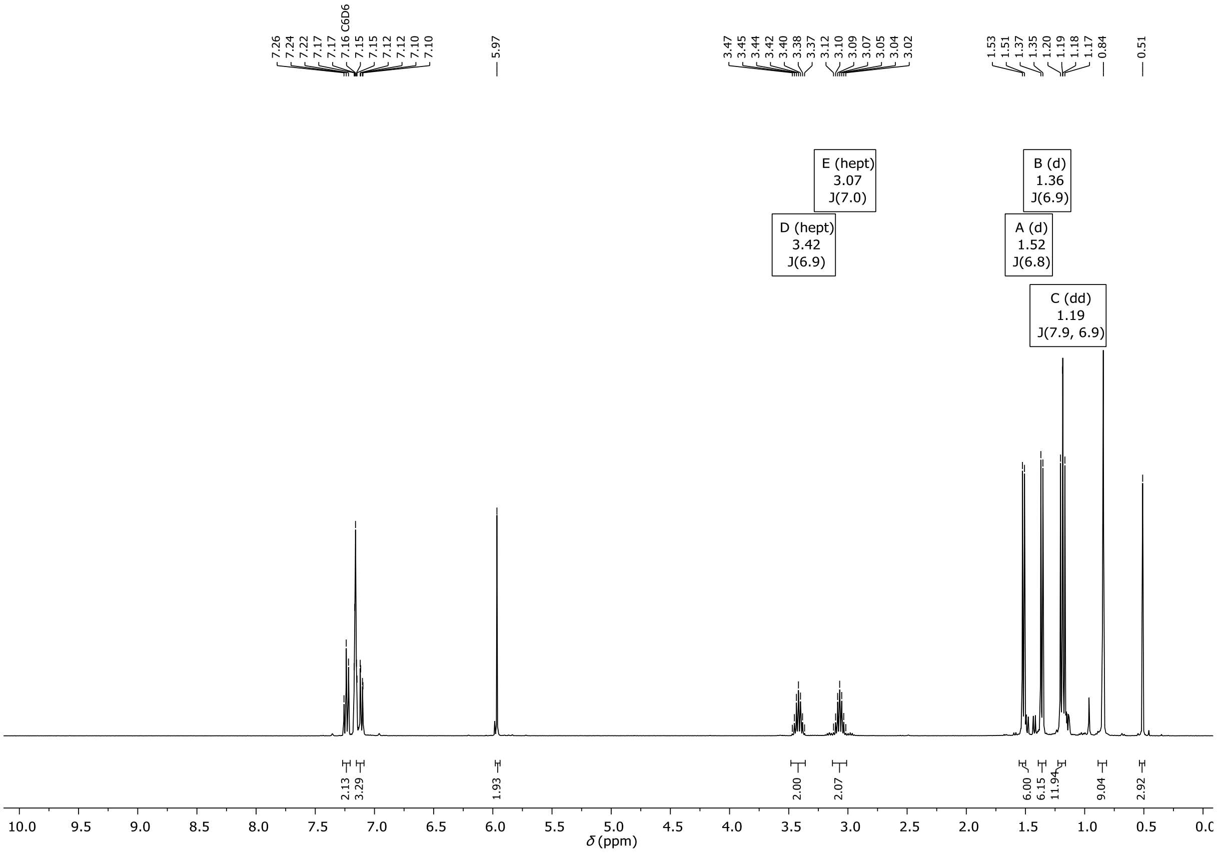

Figure S88: $^1H\{^{31}P\}$ NMR spectrum (400 MHz, C_6D_6) of **11**.

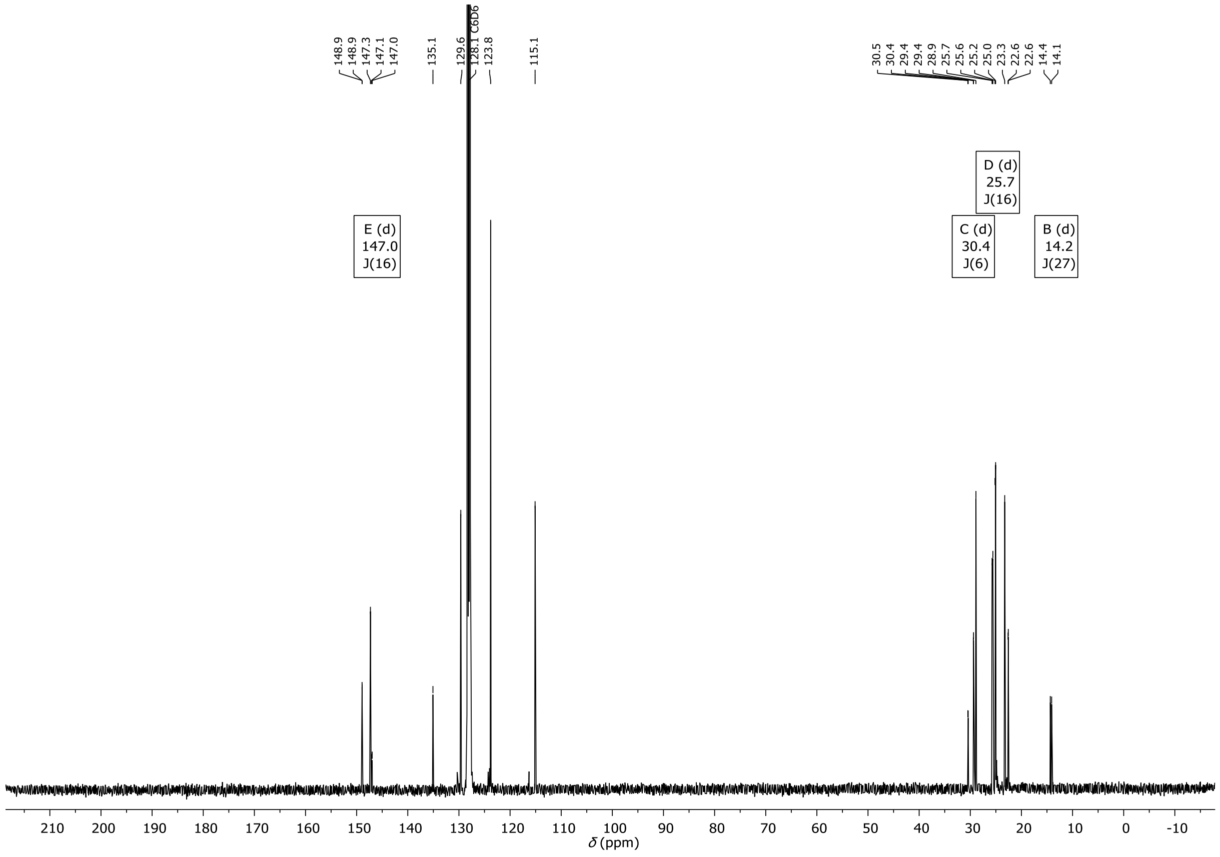

Figure S89: $^{13}C\{^{1}H\}$ NMR spectrum (101 MHz, C_6D_6) of **11**.

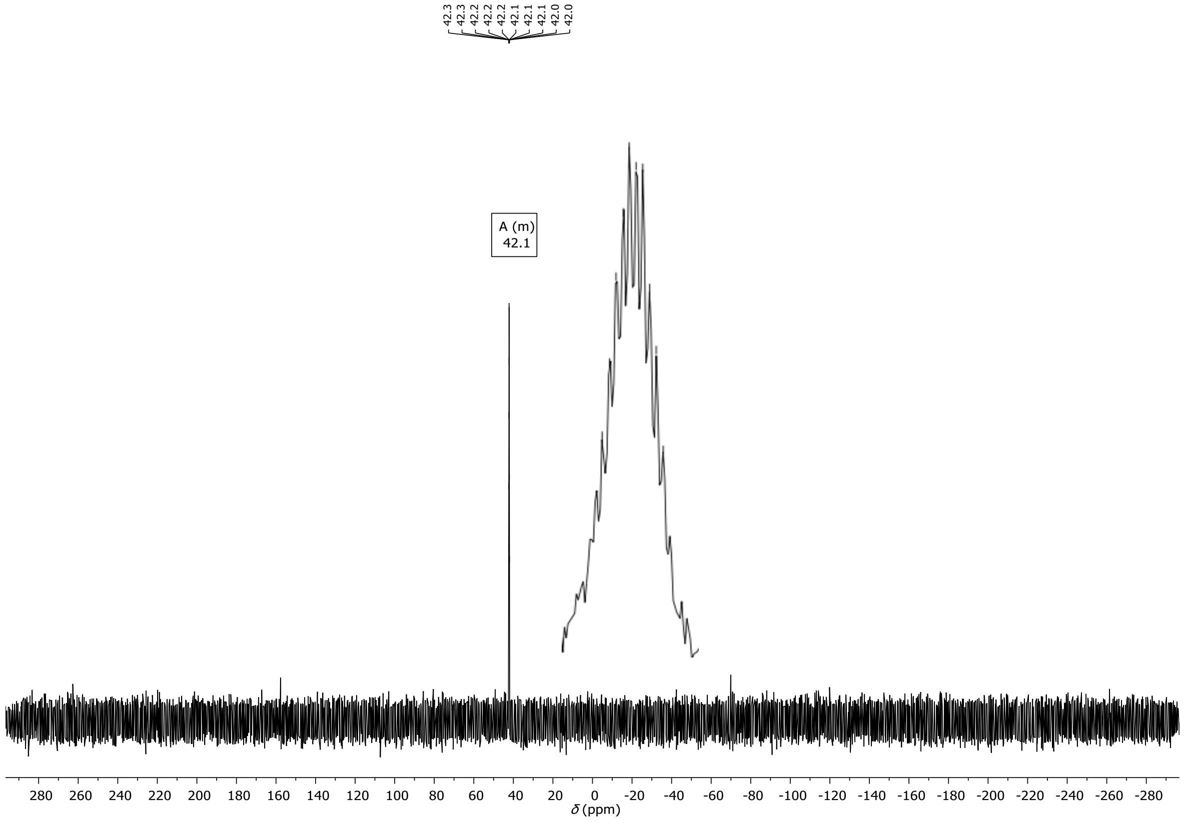

Figure S90: ^{31}P NMR spectrum (162 MHz, C_6D_6) of **11**.

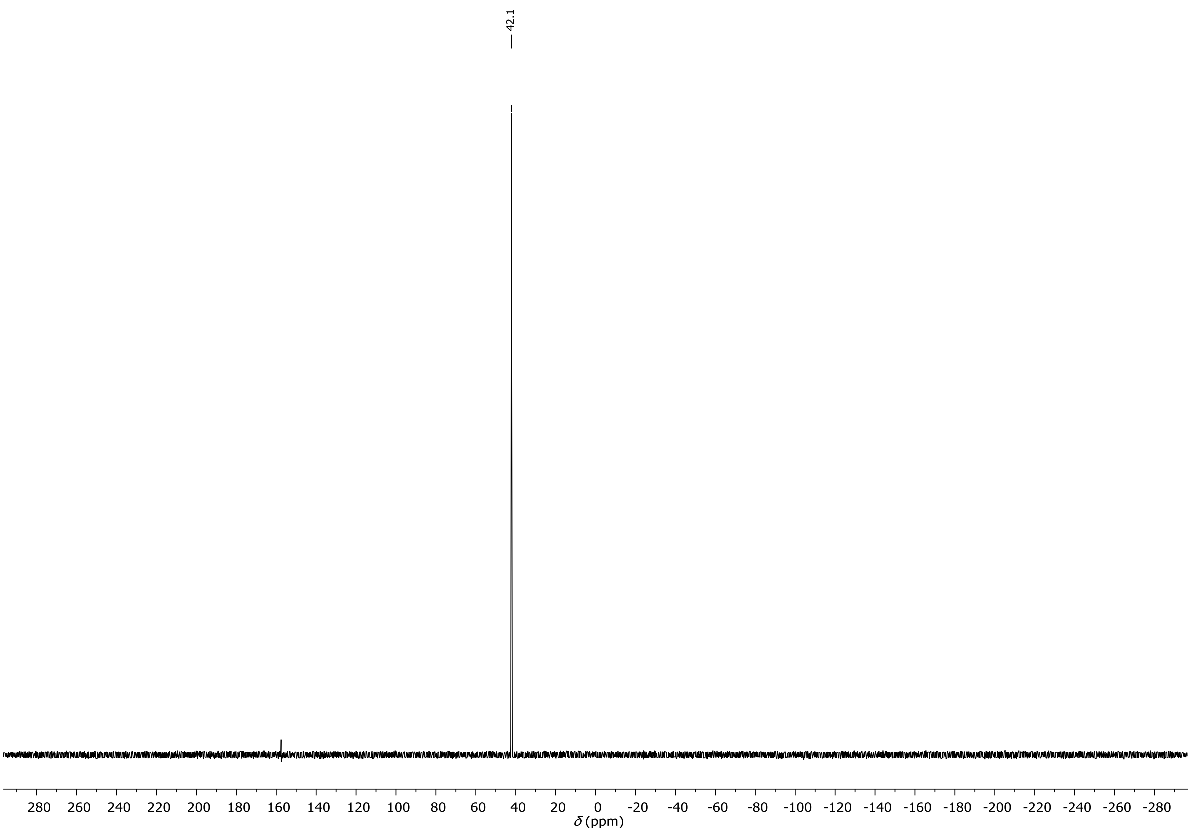

Figure S91: $^{31}P\{^{1}H\}$ NMR spectrum (162 MHz, C_6D_6) of **11**.

OPMe(NIdipp)tBu **12**

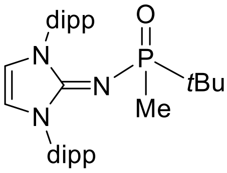

In a NMR tube with a gas cap, a solution of **11** (12 mg, 0.0237 mmol, 1.00 eq.) in benzene (0.6 mL) was frozen and subsequently the argon atmosphere was removed *in vacuo*. After warming the reaction mixture to room temperature, N_2O (1.5 bar) was pressured on the reaction vessel. The reaction was monitored by ^{31}P NMR, revealing the formation of a transient species at 42.1 ppm presumably the phosphine-N_2O-adduct, which reacts over time to the reaction product **12** at 31.1 ppm. The reaction mixture was stirred overnight under N_2O atmosphere to complete the reaction. After removal of all volatiles *in vacuo*, the phosphine oxide **12** was received as a white powder (14 mg, 0.0230 mmol, 97%).

^{1}H NMR (400 MHz, C_6D_6) *δ* (ppm) = 7.25 - 7.17 (m, 4H, Ar-H), 7.11 - 7.09 (m, 4H, Ar-H), 6.06 (s, 2H, NCHCHN), 3.33 (sept, $^{3}J_{HH}$ = 6.9 Hz, 2H, CH (*i*Pr)), 2.94 (sept, $^{3}J_{HH}$ = 6.9 Hz, 2H, CH (*i*Pr)), 1.57 (d, $^{3}J_{HH}$ = 6.8 Hz, 6H, CH_3 (*i*Pr)), 1.38 (d, $^{3}J_{HH}$ = 6.9 Hz, 6H, CH_3 (*i*Pr)), 1.14 (t, $^{3}J_{HH}$ = 7.2 Hz, 12H, CH_3 (*i*Pr)), 0.88 (d, $^{3}J_{HP}$ = 14.5 Hz, 9H, CH_3 (*t*Bu)), 0.48 (d, $^{3}J_{HP}$ = 12.6 Hz, 3H, CH_3 (Me)).

$^{1}H\{^{31}P\}$ NMR (400 MHz, C_6D_6) *δ* (ppm) = 7.25 - 7.17 (m, 4H, Ar-H), 7.11 - 7.09 (m, 4H, Ar-H), 6.06 (s, 2H, NCHCHN), 3.33 (sept, $^{3}J_{HH}$ = 6.9 Hz, 2H, CH (*i*Pr)), 2.94 (sept, $^{3}J_{HH}$ = 6.9 Hz, 2H, CH (*i*Pr)), 1.57 (d, $^{3}J_{HH}$ = 6.8 Hz, 6H, CH_3 (*i*Pr)), 1.38 (d, $^{3}J_{HH}$ = 6.8 Hz, 6H, CH_3 (*i*Pr)), 1.14 (t, $^{3}J_{HH}$ = 7.2 Hz, 12H, CH_3 (*i*Pr)), 0.88 (s, 9H, CH_3 (*t*Bu)), 0.48 (s, 3H, CH_3 (Me)).

^{13}C{^{1}H} NMR (C_6D_6, 101 MHz) δ (ppm) = 148.4 (s, C (Ar-H)), 147.5 (d, $^{2}J_{CP}$ = 10 Hz, NCN), 147.4 (s, q-C (Ar-*i*Pr)), 134.1 (s, ipso-C (Ar)), 130.1 (s, C (Ar-H)), 124.2 (s, C (Ar-H)), 124.1 (s, C (Ar-H)), 115.9 (s, NCHCHN), 33.1 (d, $^{1}J_{CP}$ = 106 Hz, q-C (*t*Bu)), 29.4 (s, CH (*i*Pr)), 29.0 (s, CH (*i*Pr)), 25.4 (s, CH_3 (*i*Pr)), 25.1 (s, CH_3 (*t*Bu)), 25.0 (s, CH_3 (*i*Pr)), 23.2 (s, CH_3 (*i*Pr)), 23.0 (s, CH_3 (*i*Pr)), 12.8 (d, $^{1}J_{CP}$ = 79 Hz, CH_3 (Me)).

^{31}P NMR (C_6D_6, 162 MHz) δ (ppm) = 31.4 - 30.9 (m).

^{31}P{^{1}H} NMR (162 MHz, C_6D_6) δ (ppm) = 31.1 (s).

HRMS (ESI, CH_3CN) m/z = 522.3602 ($[M+H]^+$, calculated: 522.3608).

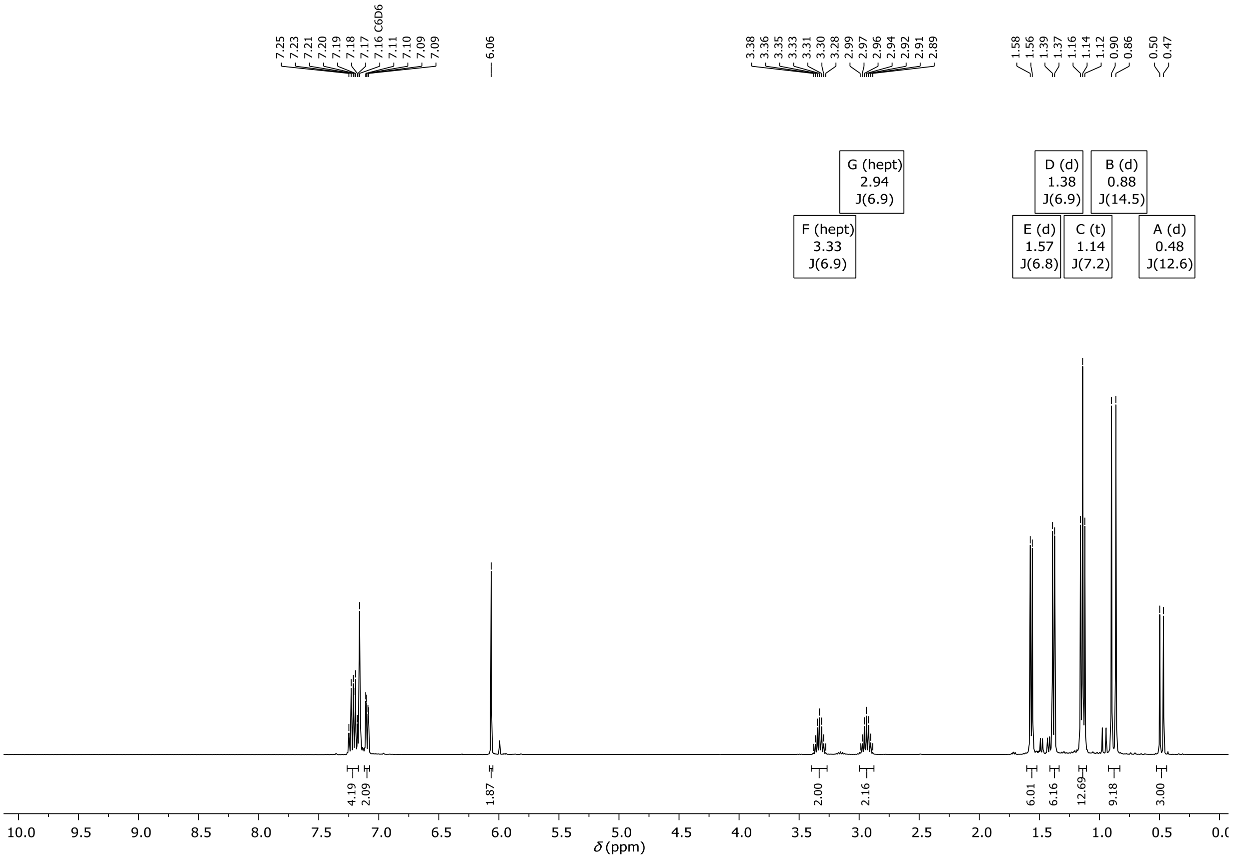

Figure S92: ^{1}H NMR spectrum (400 MHz, C_6D_6) of **12**.

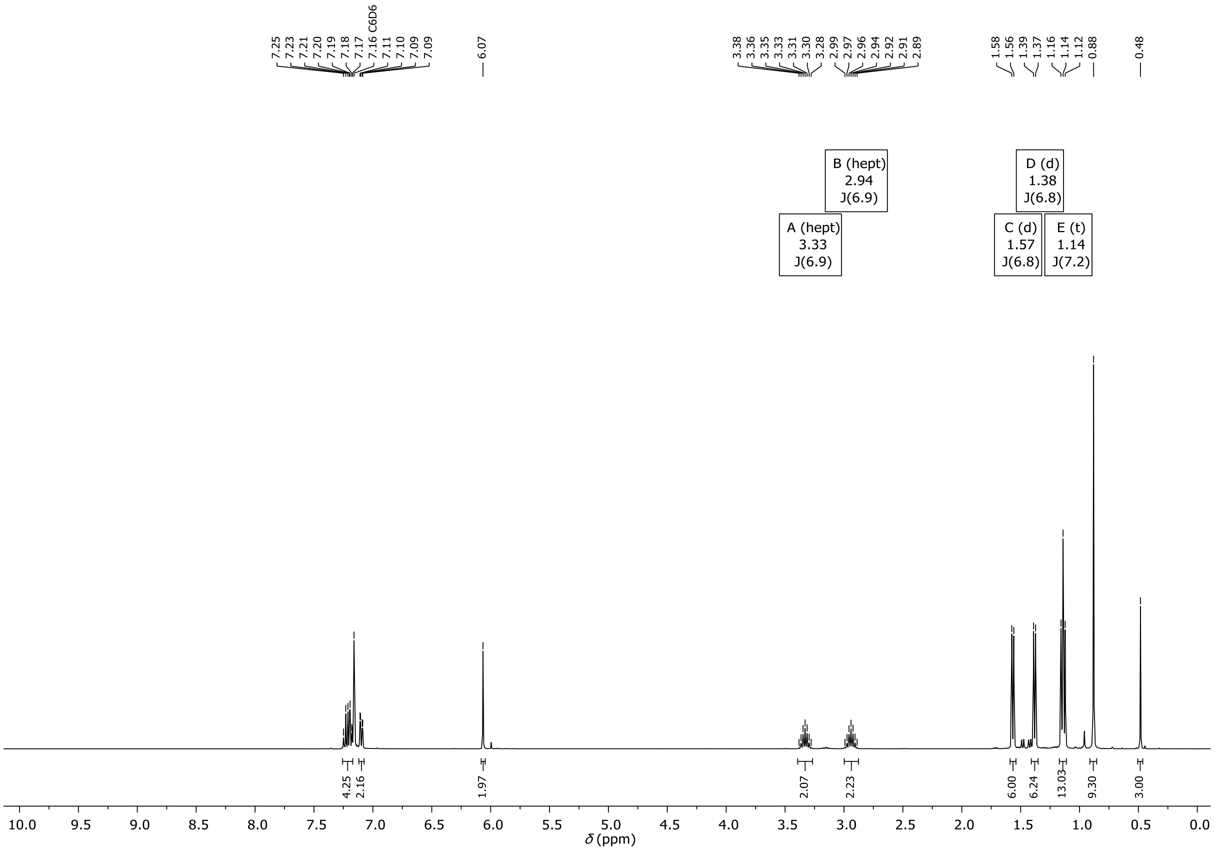

Figure S93: $^{1}H\{^{31}P\}$ NMR spectrum (400 MHz, C_6D_6) of **12**.

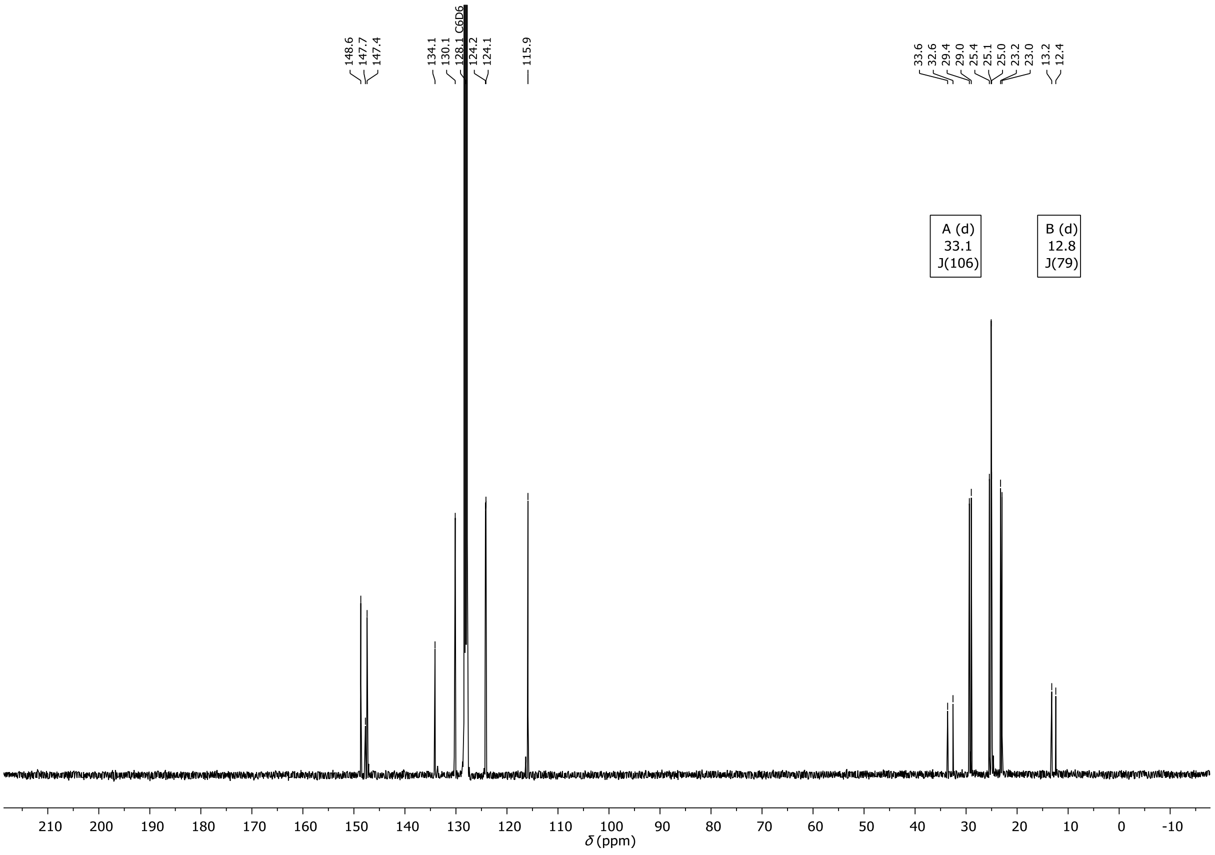

Figure S94: $^{13}C\{^{1}H\}$ NMR spectrum (101 MHz, C_6D_6) of **12**.

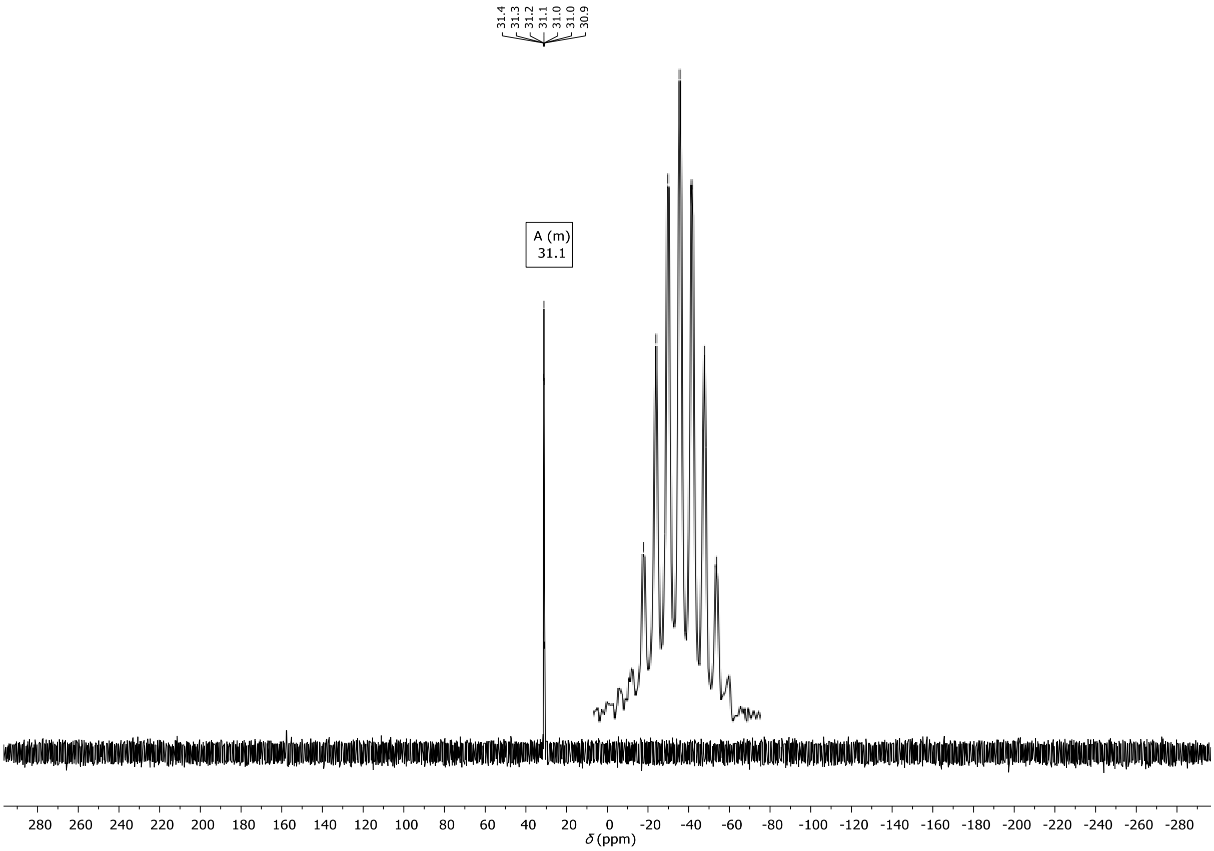

Figure S95: ^{31}P NMR spectrum (101 MHz, C_6D_6) of **12**.

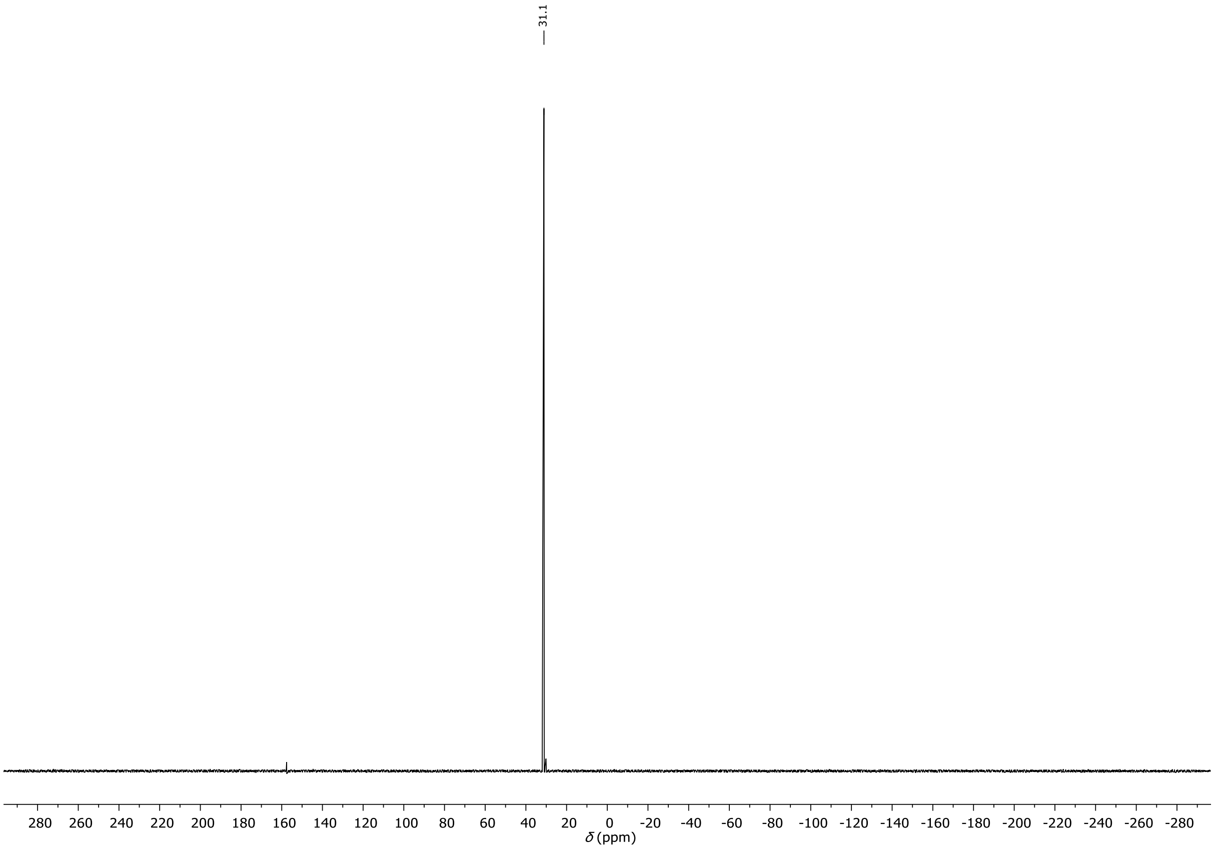

Figure S96: $^{31}P\{^1H\}$ NMR spectrum (101 MHz, C_6D_6) of **12**.

Light and temperature stability

6:

6 (8 mg, 0.012 mmol) was dissolved in C_6D_6 (0.6 mL). The solution was irradiated at 365 nm and 280 nm for intervals of 5, 15 and 60 min. Note that the used LED array at 280 nm heats the NMR tube to 80 °C despite cooling with a fan. ^{1}H and ^{31}P NMR spectroscopy did not show significant decomposition.

The solution was heated to 90 °C. The reaction was controlled by ^{1}H and ^{31}P NMR spectroscopy in one hour intervals. Within 3 h, all starting material was decomposed. The ^{1}H and ^{31}P NMR spectroscopy of the reaction mixture showed that the ^{31}P signal is decreasing (see Figure S98) while the ^{1}H signals are broadening (see Figure S97).

10:

10 (15 mg, 0.028 mmol) was dissolved in THF-d_8 (0.6 mL). The solution was irradiated at 365 nm and 280 nm for intervals of 5, 15 and 60 min. Note that the used LED array at 280 nm produces a temperature of 80 °C despite cooling. ^{1}H and ^{31}P NMR spectroscopy did not show significant decomposition.

10 (10 mg, 0.019 mmol) was dissolved in C_6D_6 (0.6 mL). The solution was heated 1 h at 60 °C, 2 h at 70 °C and 2h at 90 °C. The reaction was controlled by ^{1}H and ^{31}P NMR spectroscopy in one hour intervals. The ^{1}H and ^{31}P NMR spectroscopy of the reaction mixture showed the decomposition into multiple ^{31}P species (see Figure S100) while the ^{1}H signals are broadening (see Figure S99). As main component, phosphine **11** can be identified in the reaction mixture by ^{1}H (see Figure S101) and ^{31}P NMR (see Figure S102).

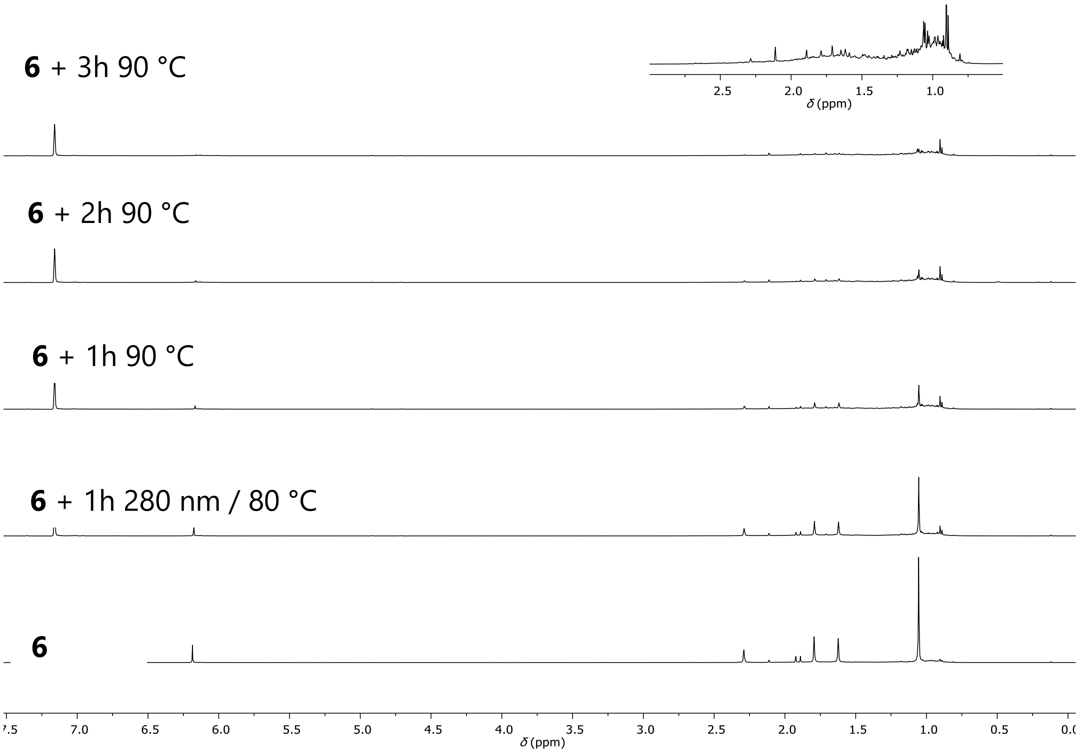

Figure S97: Stacked ^{1}H NMR spectra (400 MHz, C_6D_6) of **6** after irradiation or heating for indicated times and temperatures.

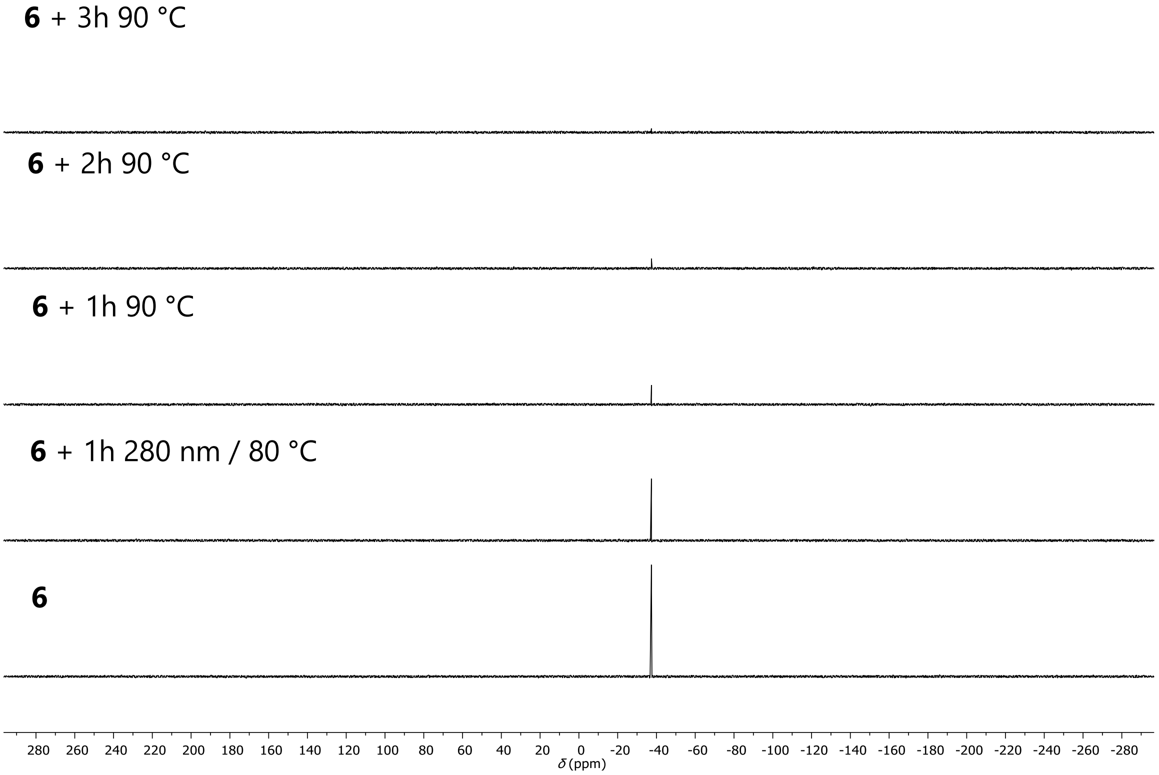

Figure S98: Stacked $^{31}P\{^{1}H\}$ NMR spectra (162 MHz, C_6D_6) of **6** after irradiation or heating for indicated times and temperatures.

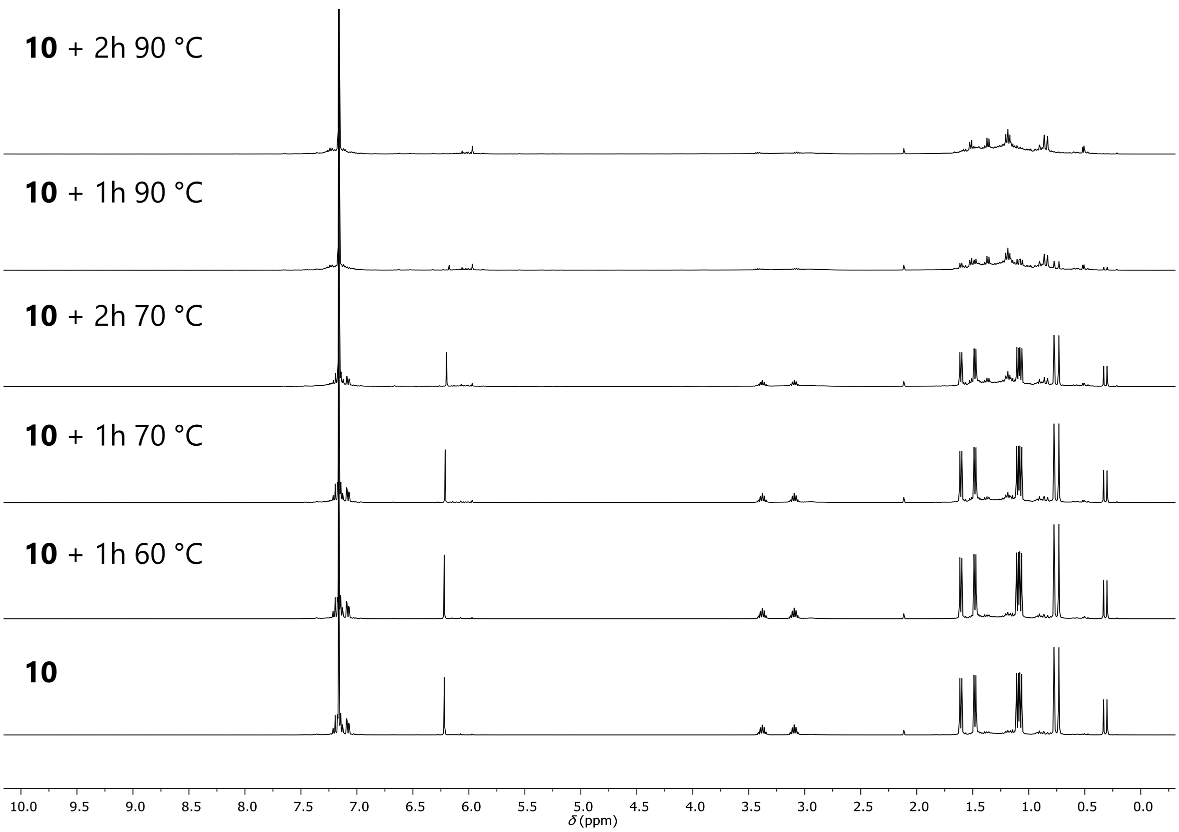

Figure S99: Stacked ^{1}H NMR spectra (400 MHz, C_6D_6) of **10** after irradiation or heating for indicated times and temperatures.

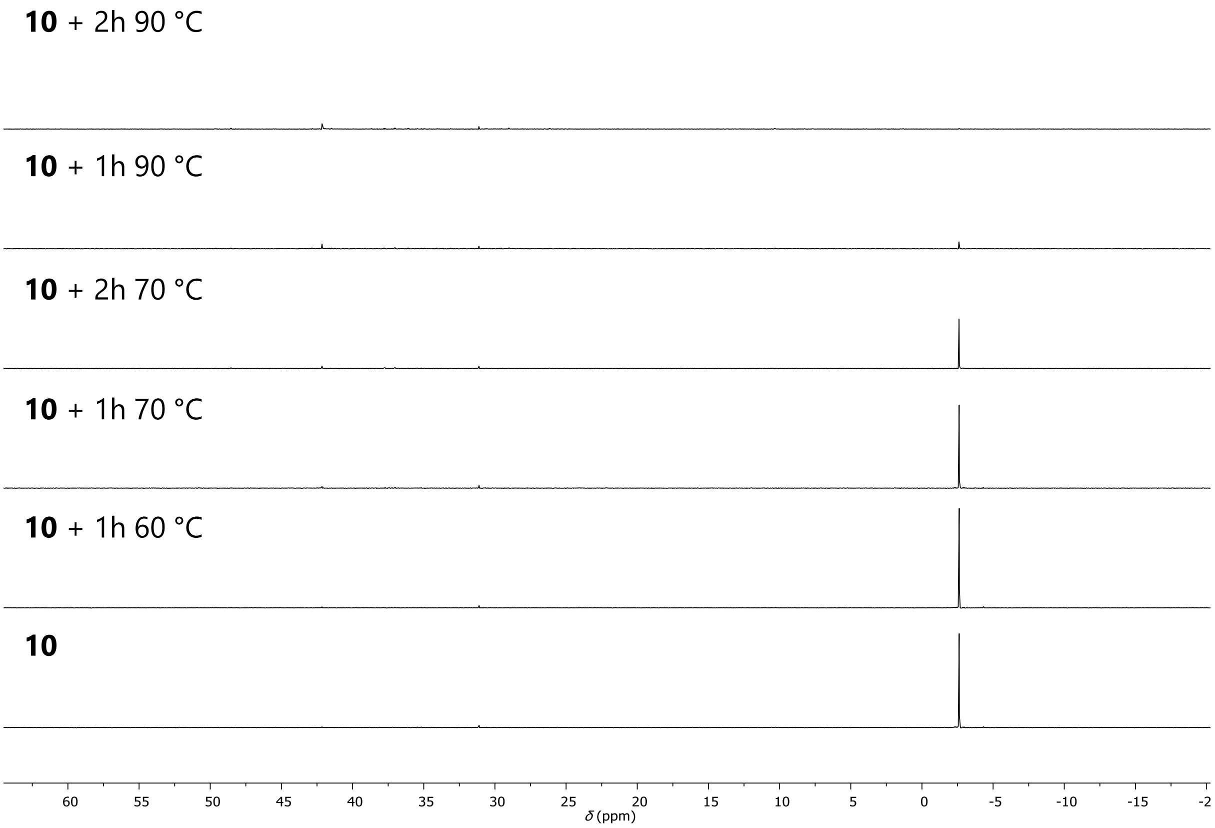

Figure S100: Stacked $^{31}P\{^{1}H\}$ NMR spectra (162 MHz, C_6D_6) of **10** after irradiation or heating for indicated times and temperatures.

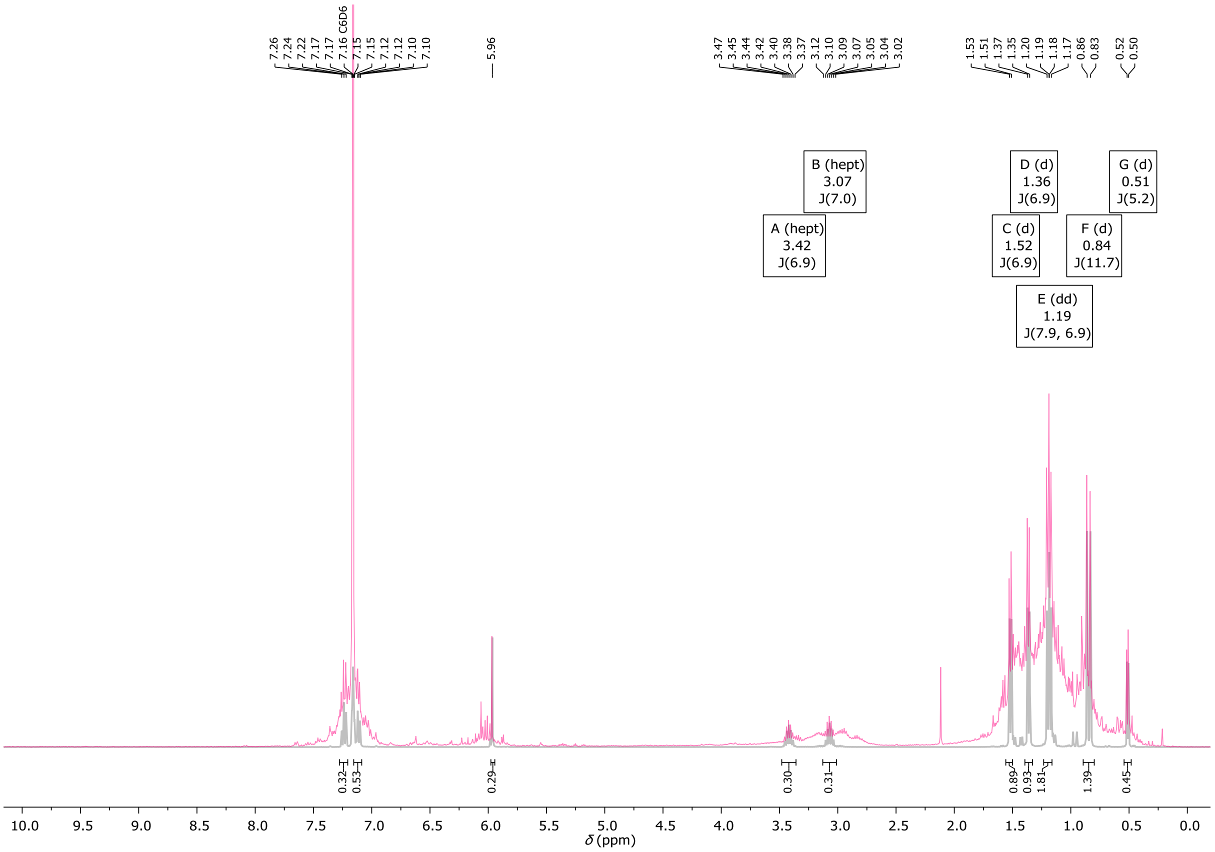

Figure S101: Superimposed ^{1}H NMR spectra (400 MHz, C_6D_6) of **10** after heating for 1 h at 60 °C, 2 h at 70 °C and 2h at 90 °C in total (pink) and **11** (grey).

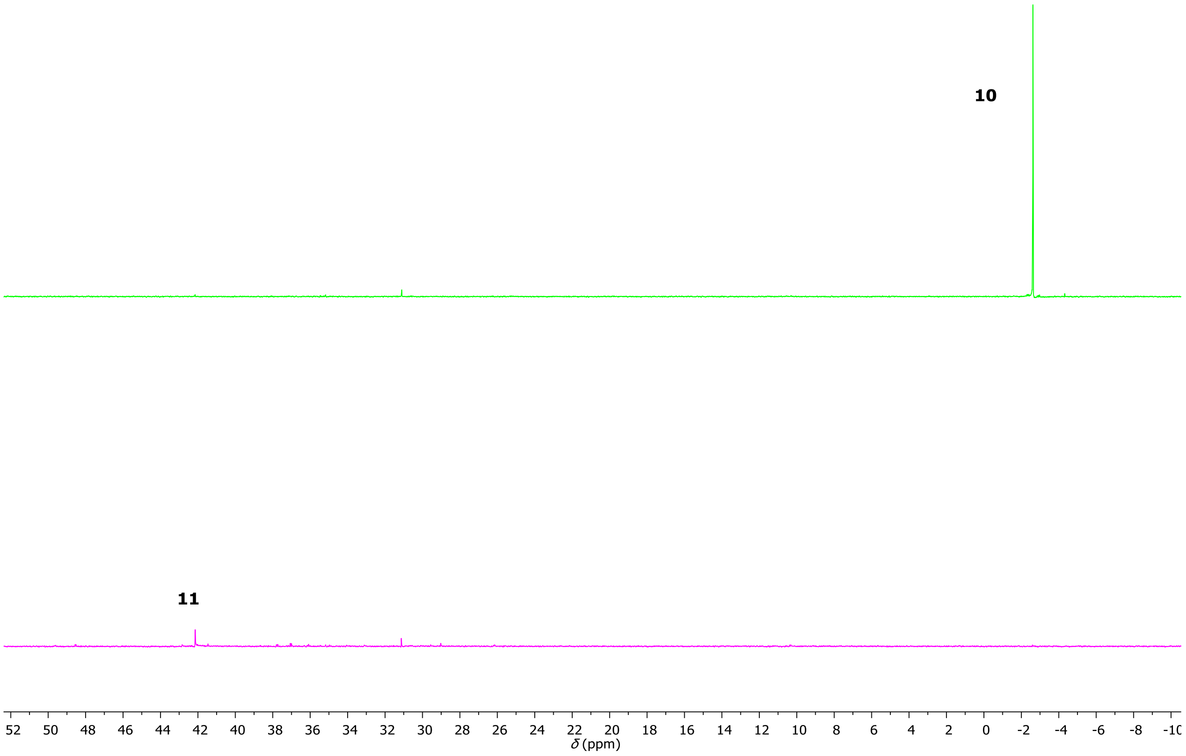

Figure S102: Stacked $^{31}P\{^{1}H\}$ NMR spectra (400 MHz, C_6D_6) of **10** before (green) and after heating for 1 h at 60 °C, 2 h at 70 °C and 2h at 90 °C in total (pink).

Reactivity with water

6: H_2O (1.0 µL, 0.0566 mmol, 2.00 eq.) was added to a solution of **6** (19 mg, 0.0283 mmol, 1.00 eq.) in THF (0.7 mL). The reaction mixture was left overnight. The reaction mixture was analyzed by ^{31}P NMR spectroscopy, which indicates full consumption of **6** into several phosphorus containing species with no discernible main product (see Figure S103). Due to the large number of detected compounds, quantitative ^{31}P NMR was no longer sufficiently meaningful. ^{13}C NMR spectroscopy did not reveal acetylene (73.5 ppm) in the reaction mixture (see Figure S104).

10: H_2O (1.0 µL, 0.0566 mmol, 2.00 eq.) was added to a solution of **10** (15 mg, 0.0283 mmol, 1.00 eq.) in deuterated THF (0.5 mL). The reaction mixture was left overnight. The reaction mixture was analyzed by quantitative ^{31}P NMR spectroscopy, which indicates full consumption of **10** with 65% conversion to **12** (see Figure S106). ^{13}C NMR spectroscopy revealed acetylene (73.5 ppm) in the reaction mixture (see Figure S108).

The reaction was repeated with D_2O. D_2O (1.0 µL, 0.0566 mmol, 2.00 eq.) was added to a solution of **10** (15 mg, 0.0283 mmol, 1.00 eq.) in deuterated THF (0.5 mL). The reaction mixture was left over night. Similar to H_2O quantitative ^{31}P NMR spectroscopy indicates full consumption of **10** with 72% conversion to **12** (see Figure S110) and ^{13}C NMR spectroscopy revealed acetylene (73.5 ppm) in the reaction mixture (see Figure S112). ^{1}H NMR spectroscopy revealed a decrease of detected ^{1}H at the methyl group which probably corresponds to H/D exchange with the residual D_2O. A proton signal corresponding to the acetylene was not identified for both experiments.

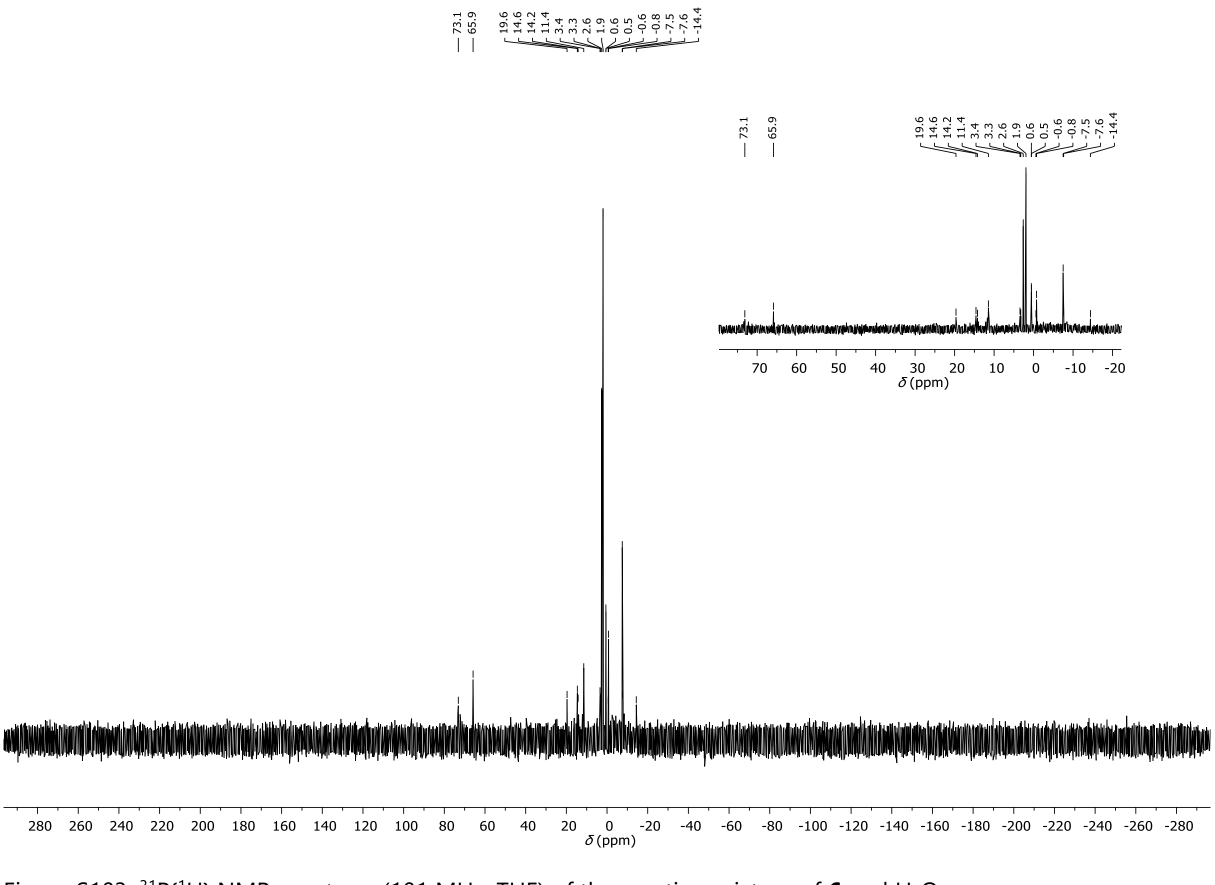

Figure S103: ^{31}P{^{1}H} NMR spectrum (101 MHz, THF) of the reaction mixture of **6** and H_2O.

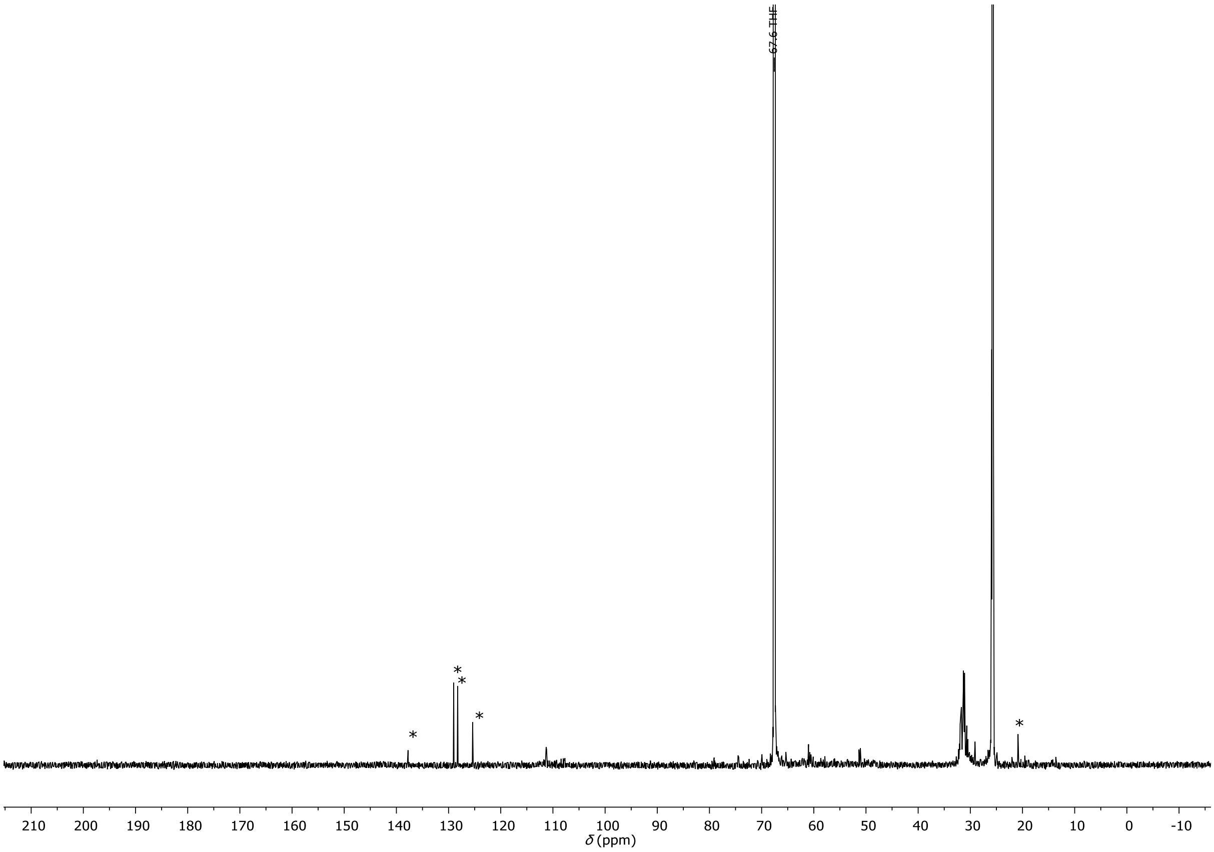

Figure S104: $^{13}C\{^1H\}$ NMR spectrum (101 MHz, THF) of the reaction mixture of **6** and H_2O (* marks residual toluene).

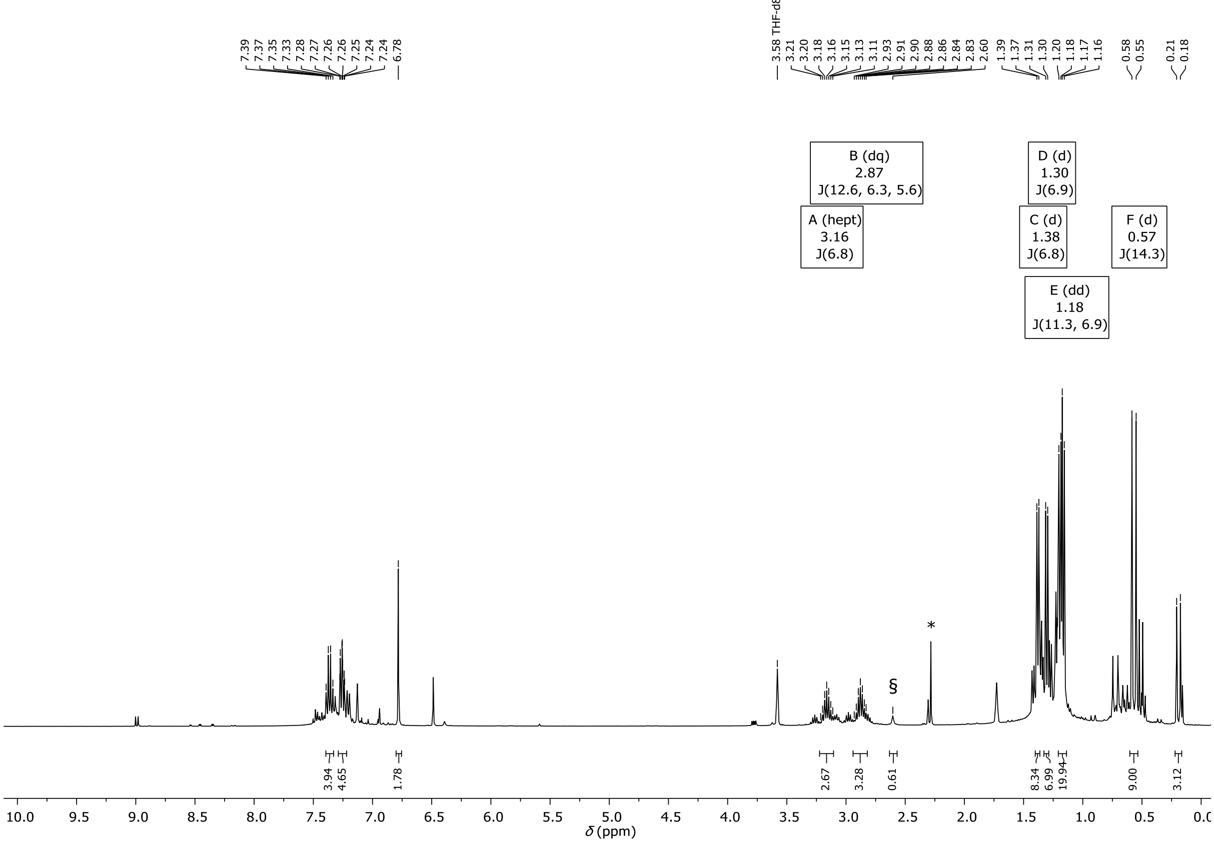

Figure S105: ^1H NMR spectrum (101 MHz, THF) of the reaction mixture of **10** and H_2O (* marks residual toluene, [$] marks residual H2O/DHO).

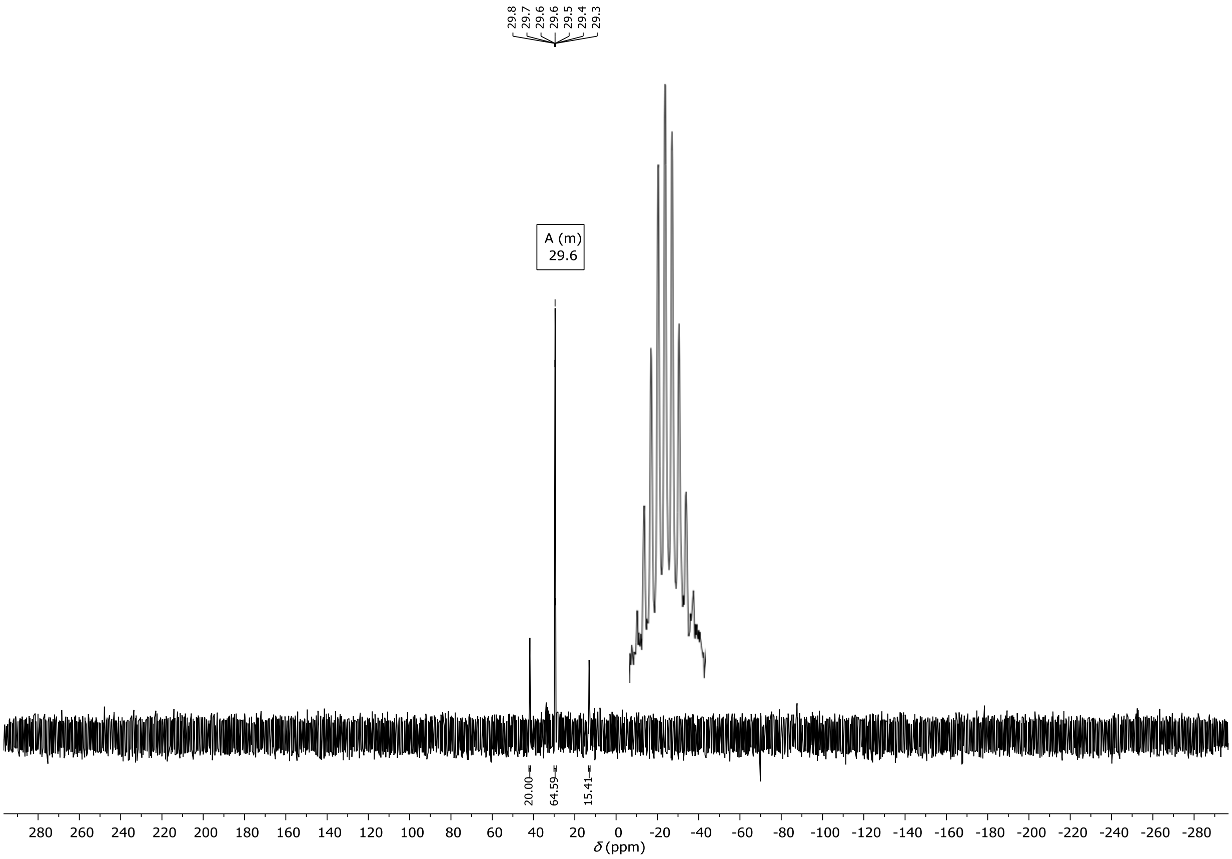

Figure S106: Quantitative ^{31}P NMR spectrum (101 MHz, THF-d_8, relaxation delay 25000, acquisition time 0.5) of the reaction mixture of **10** and H_2O showing the resonance of **12** at 29.6 ppm.

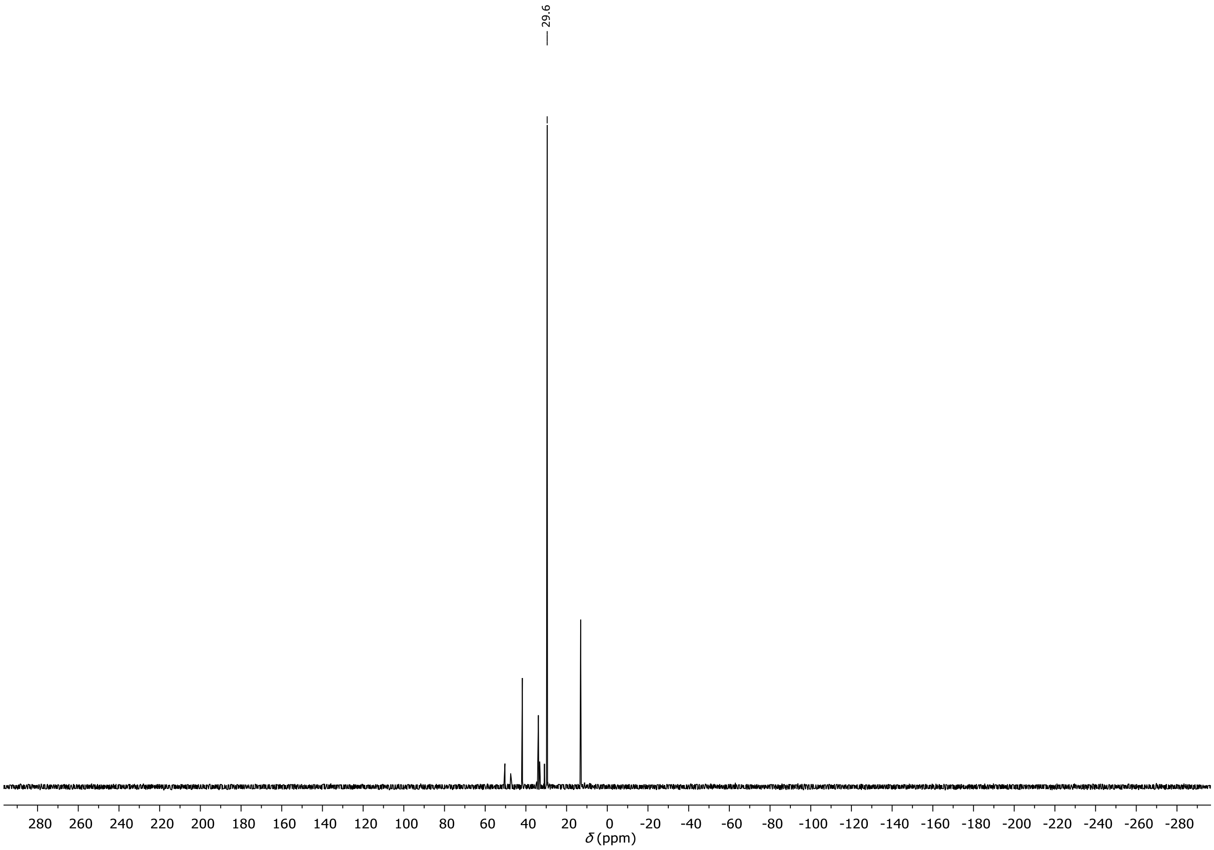

Figure S107: $^{31}P\{^1H\}$ NMR spectrum (101 MHz, THF-d_8) of the reaction mixture of **10** and H_2O showing the resonance of **12** at 29.6 ppm.

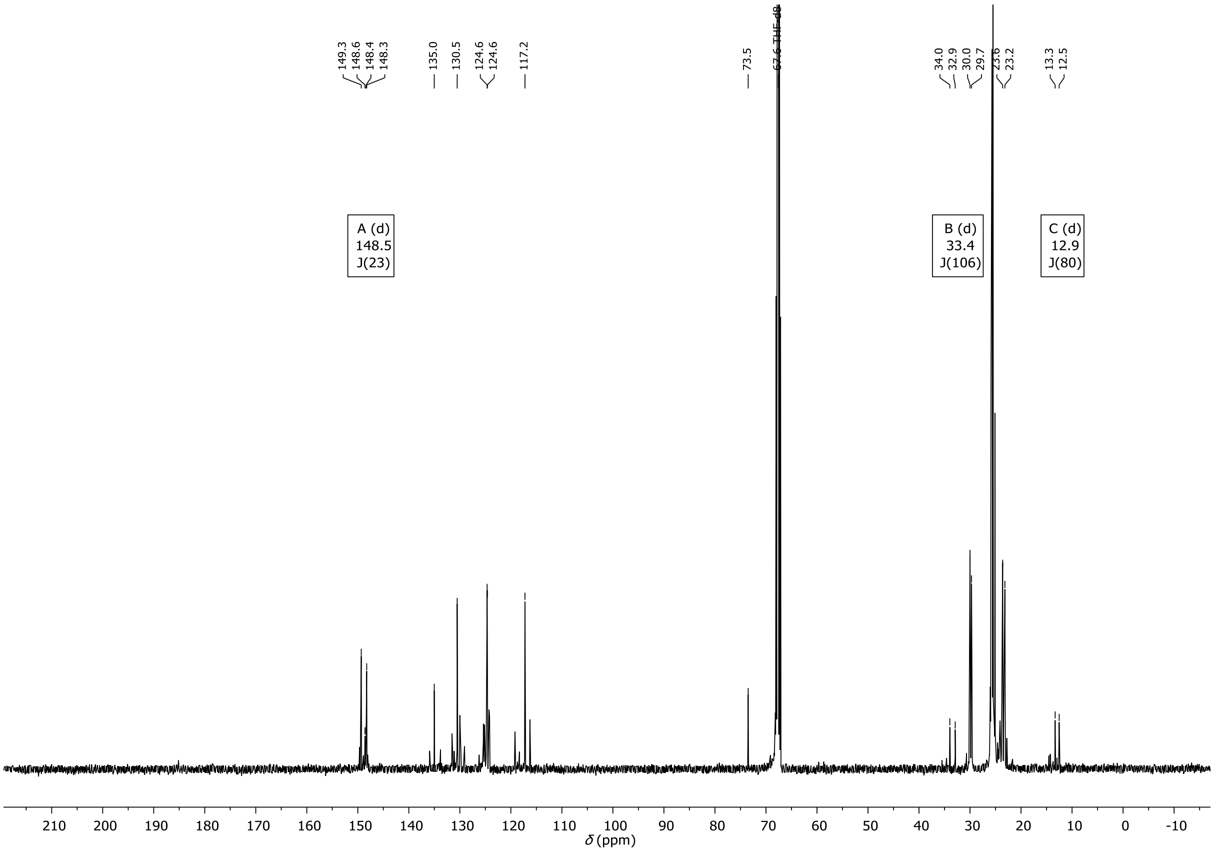

Figure S108: $^{13}C\{^{1}H\}$ NMR spectrum (101 MHz, THF-d_8) of the reaction mixture of **10** and H_2O.

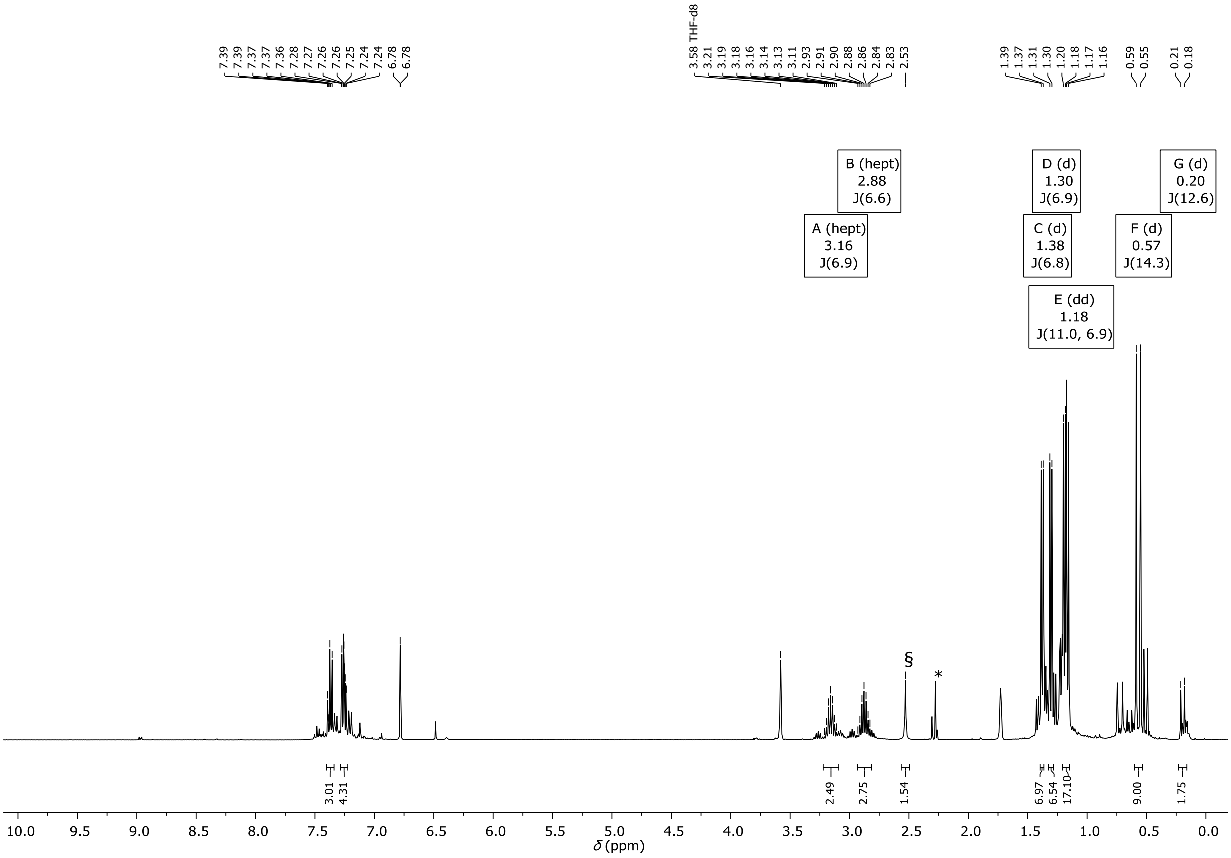

Figure S109: ^{1}H NMR spectrum (101 MHz, THF) of the reaction mixture of **10** and D_2O (* marks residual toluene, $^{\$}$ marks residual H_2O/DHO).

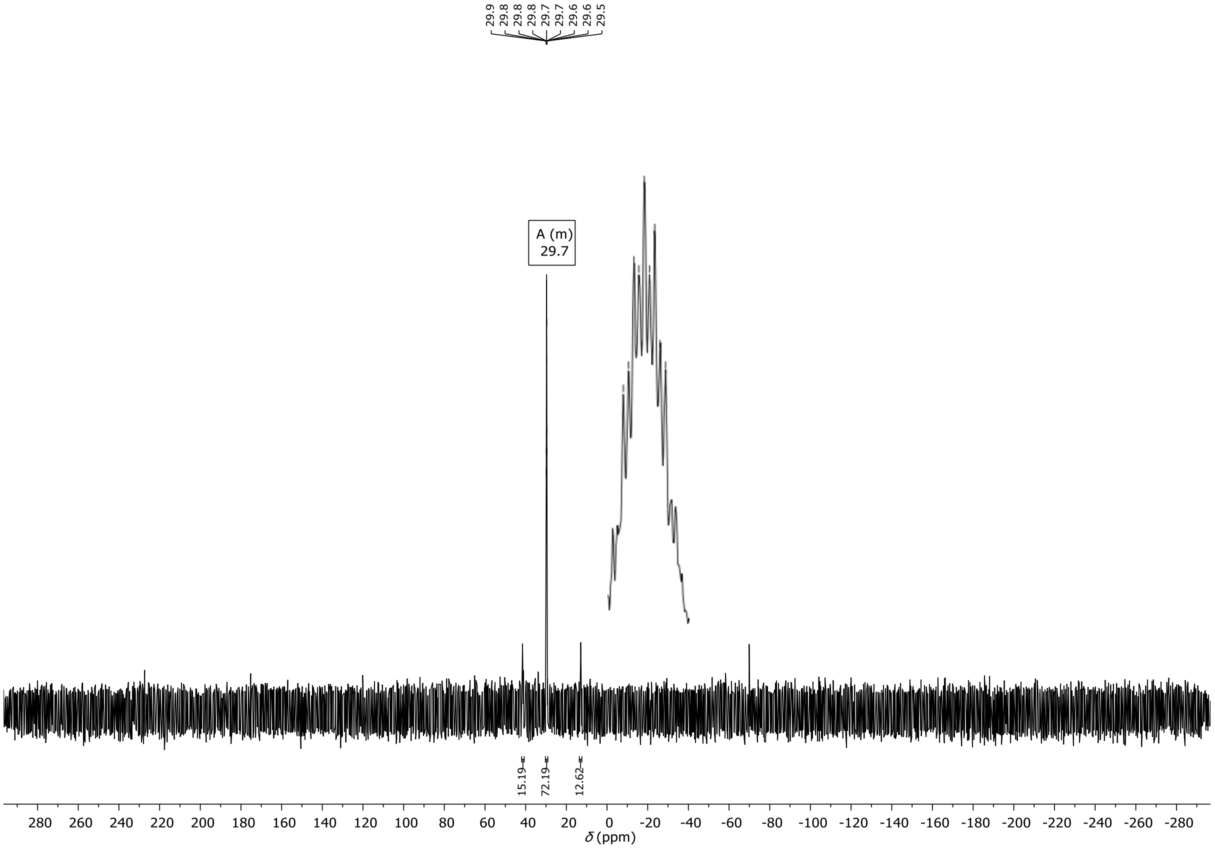

Figure S110: Quantitative ^{31}P NMR spectrum (101 MHz, THF-d_8, relaxation delay 25000, acquisition time 0.5) of the reaction mixture of **10** and D_2O showing the resonance of **12** at 29.7 ppm.

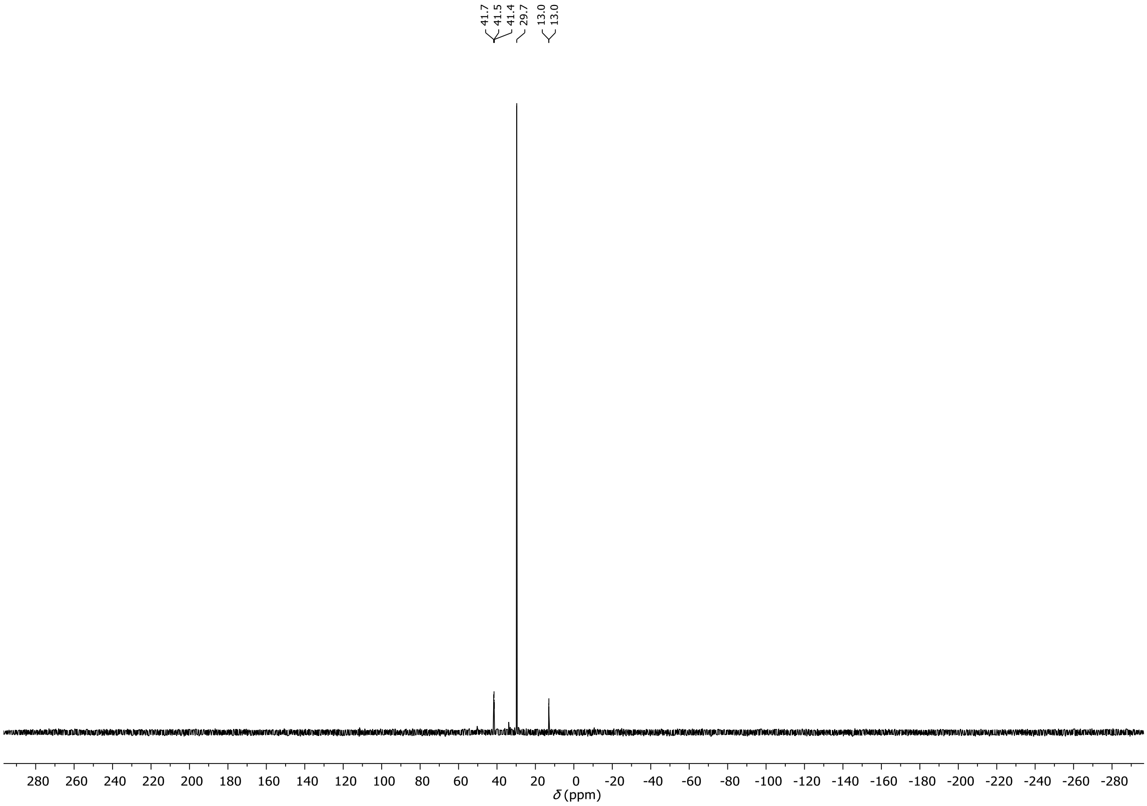

Figure S111: ^{31}P{^{1}H} NMR spectrum (101 MHz, THF-d_8) of the reaction mixture of **10** and D_2O showing the resonance of **12** at 29.7 ppm.

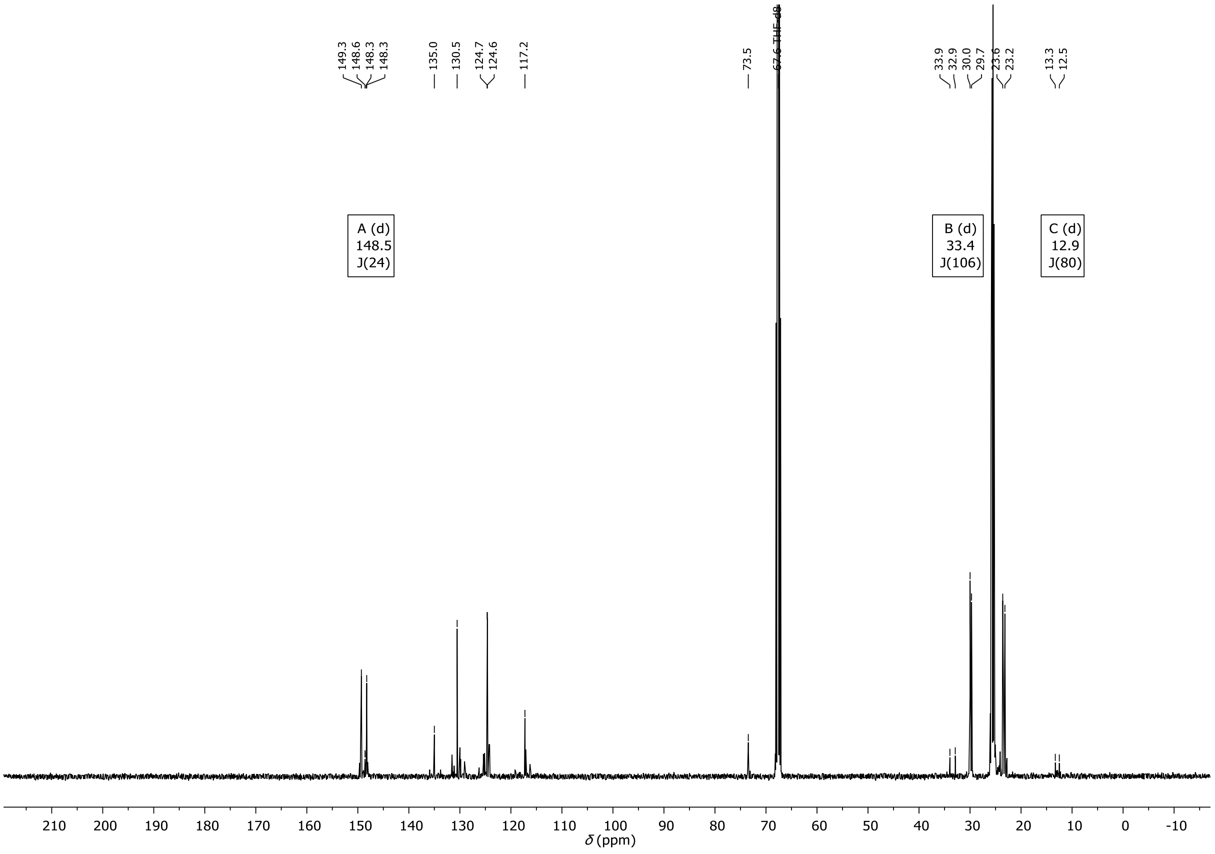

Figure S112: $^{13}C\{^{1}H\}$ NMR spectrum (101 MHz, THF-d_8) of the reaction mixture of **10** and D_2O.

<u>[Au((CC)PMe(NI*t*Oct)$_2$)$_2$][Cl]</u> **13**

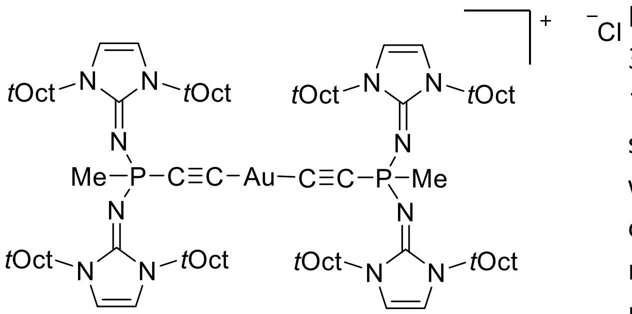

Phosphonioacetylide **6** (31 mg, 0.0312 mmol, 3.00 eq.$^{\$}$) and Au(tht)Cl (5 mg, 0.0156 mmol, 1.00 eq.) were dissolved in toluene (3 mL) and stirred overnight. By ^{31}P NMR full conversion was observed with residual **6**. All volatile compounds were removed *in vacuo*. The residue was washed with diethyl ether to remove the residual **6** (2x 1 mL). The residue was dried *in vacuo*. **13** was obtained as a white solid (5 mg, 0.0031 mmol, 20%).

$^{\$}$Note that when combining 2 equivalents of **6** with 1 equivalent of Au(tht)Cl, the resulting reaction mixture showed the cationic complex **13** (-33.4 ppm) along with a minor resonance at -31.3 ppm (presumably **6**-AuCl), which is likely due to inaccurate stoichiometry (Figure S113). Therefore, another equivalent of **6** was added, resulting in full conversion to **13**.

^{1}H NMR (400 MHz, CD_3CN) δ (ppm) = 6.87 (s, 8H, CH (NCHCHN)), 2.19 - 2.05 (m, 16H, CH_2), 1.77 – 1.72 (m, 54H, $C(CH_3)_2$ + PCH_3), 0.90 (s, 36H, $C(CH_3)_3$).

^{13}C{^{1}H} NMR (101 MHz, CD_3CN) δ (ppm) = 156.9 (dd, $^{2}J_{CP}$ = 20 Hz, $^{4}J_{CP}$ = 3 Hz, βC (Au-CC)), 145.1 (d, $^{2}J_{CP}$ = 16 Hz, NCN), 113.3 (s, CH), 98.4 (d, $^{1}J_{CC}$ = 180 Hz, αC (P-CC)), 62.7 (s, q$C(CH_3)_2$), 51.7 (s, CH_2), 32.5 (s, q$C(CH_3)_3$), 31.6 (s, $(CH_3)_3$), 31.1 (d, $^{5}J_{CP}$ = 18 Hz, $(CH_3)_2$), 23.1 (d, $^{1}J_{CP}$ = 112 Hz, PCH_3).

^{31}P NMR (162 MHz, CD_3CN) δ (ppm) = −33.5 (q, $^3J_{PH}$ = 13 Hz).

$^{31}P\{^1H\}$ NMR (162 MHz, CD_3CN) δ (ppm) = −33.5.

HRMS (ESI, CH_3CN) m/z = 1562.1220 ($[M-Cl]^+$, calculated: 1562.1242).

IR (neat): ν_{max}/cm^{-1} = 2949.13, 2902.19, 2868.54, 2047.48 (C≡C), 1521.63, 1468.38, 1418.37, 1393.83, 1366.25, 1324.31, 1272.57, 1230.48, 1215.76, 1155.56, 1133.65, 1116.61, 1023.03, 992.77, 957.53, 907.01, 876.13, 811.78, 766.76, 729.14, 690.44, 633.67, 582.39, 541.95, 489.91, 471.53, 441.16.

IR (in DCM): ν_{max}/cm^{-1} = 2949.90, 2902.16, 2868.93, 2049.57 (C≡C), 1522.30, 1468.68, 1418.28, 1393.97, 1366.47, 1324.47, 1274.27, 1215.86, 1155.42, 1132.84, 1116.46, 1023.11, 992.63, 958.00, 907.27, 875.96, 811.98, 766.93, 725.37, 689.80, 634.09, 570.84, 542.12, 488.54, 471.29, 445.04.

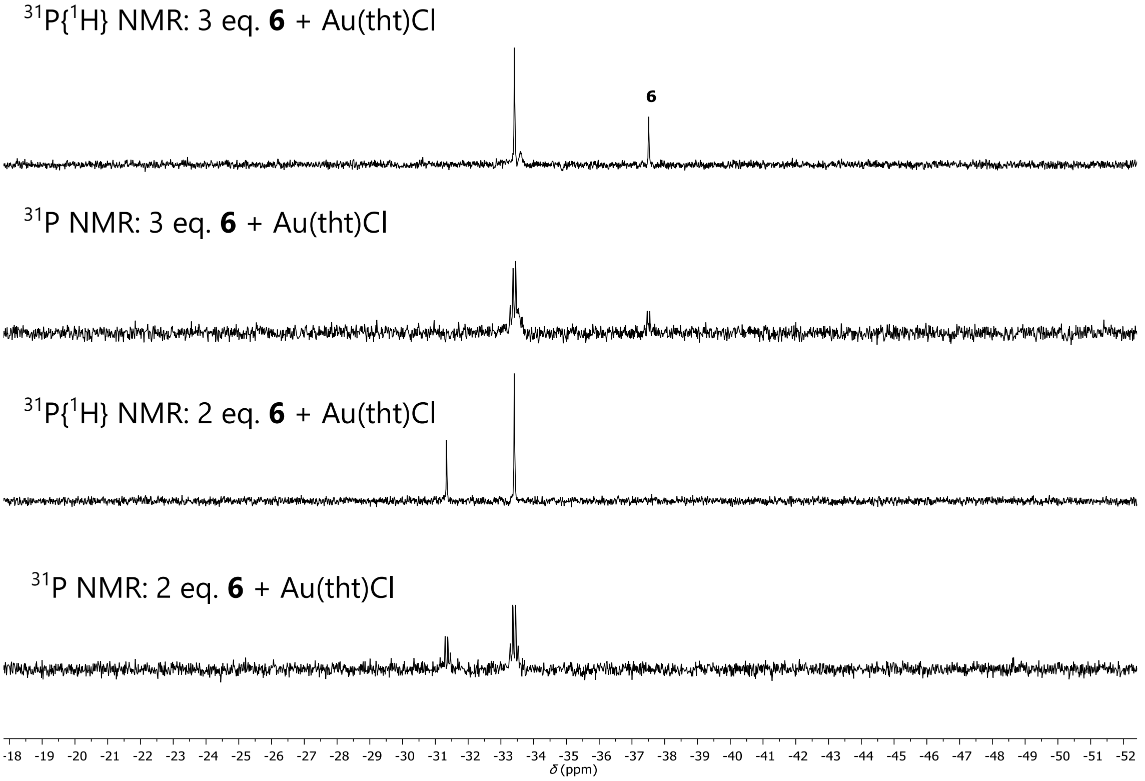

Figure S113: Stacked ^{31}P and $^{31}P\{^1H\}$ NMR spectra (162 MHz, C_6D_6) of the addition of two (bottom) or three (top) equivalents of **6** to Au(tht)Cl.

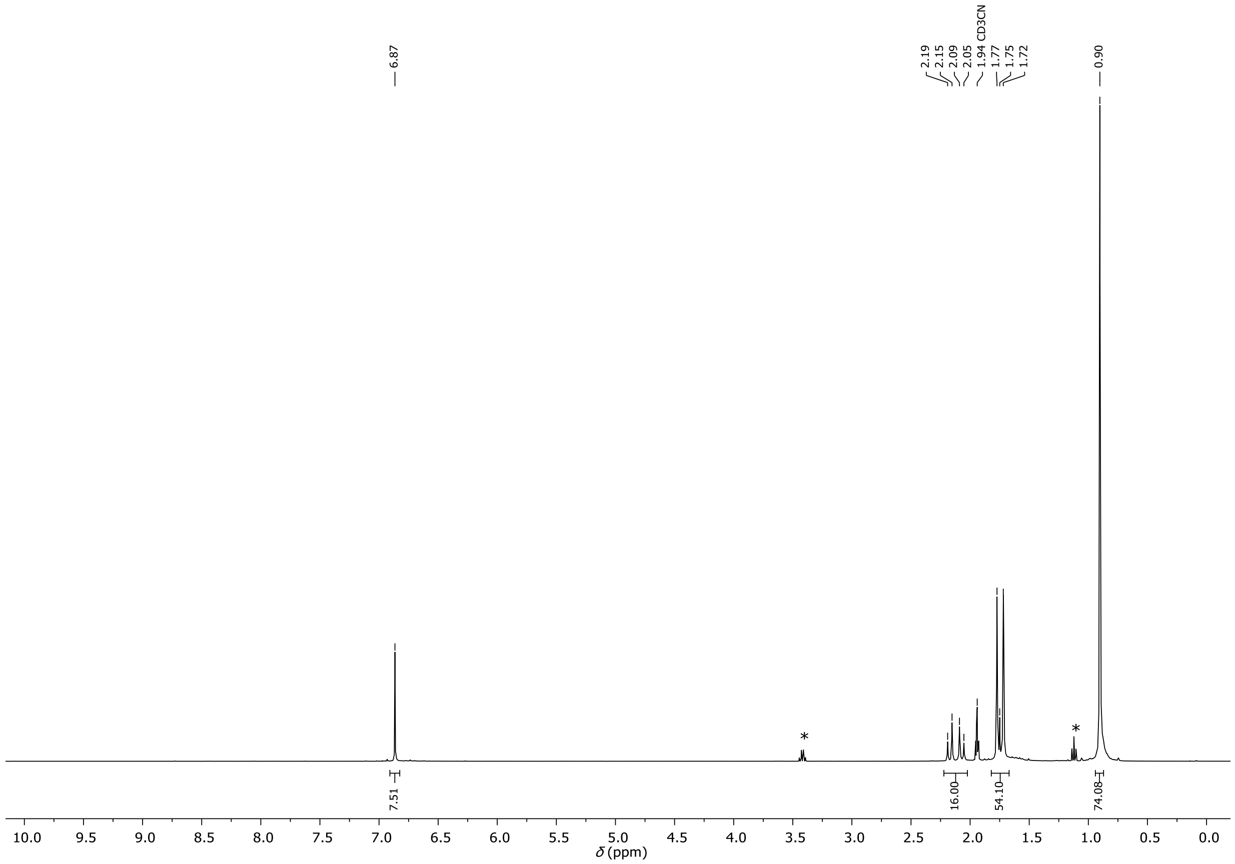

Figure S114: ^{1}H NMR spectrum (400 MHz, CD_3CN) of **13** (* marks residual diethyl ether).

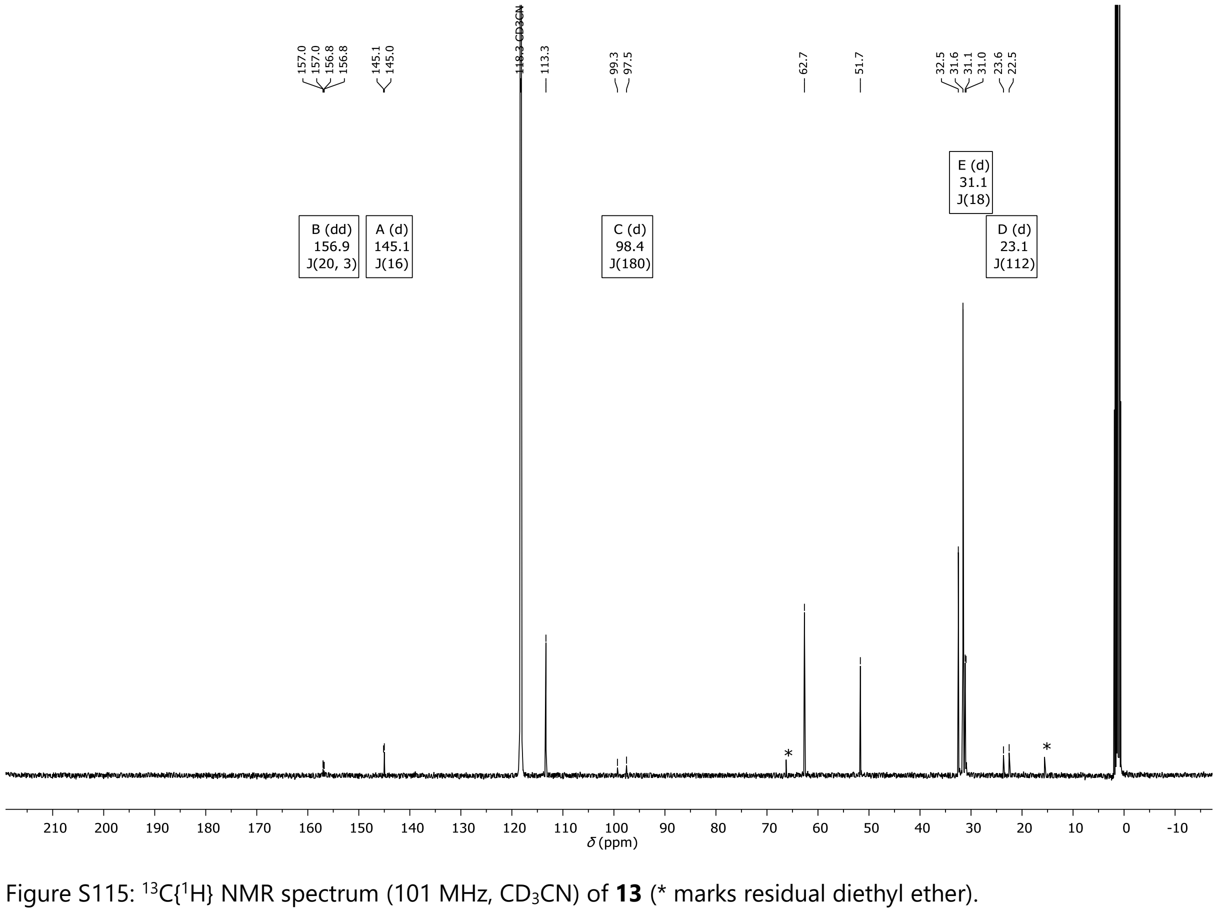

Figure S115: $^{13}C\{^{1}H\}$ NMR spectrum (101 MHz, CD_3CN) of **13** (* marks residual diethyl ether).

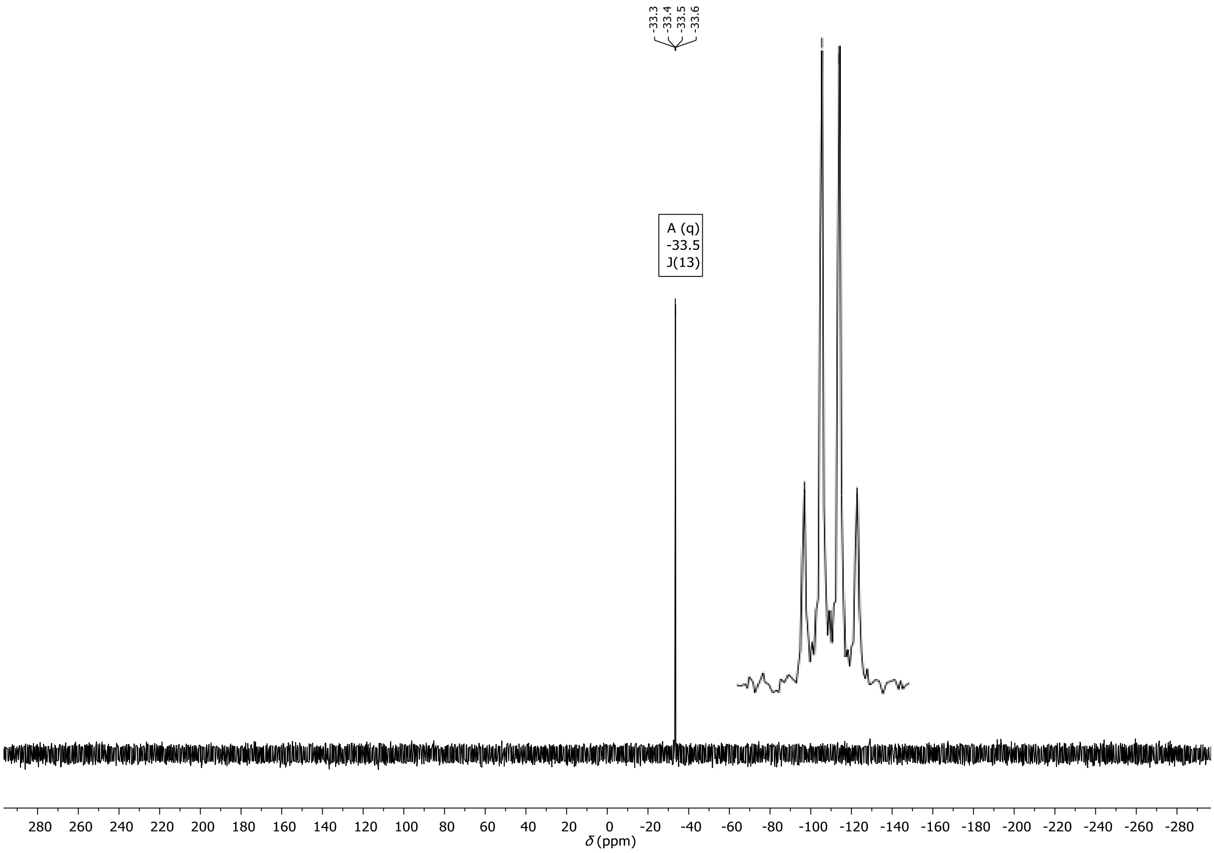

Figure S116: ^{31}P NMR spectrum (162 MHz, CD_3CN) of **13**.

Figure S117: $^{31}P\{^1H\}$ NMR spectrum (162 MHz, CD_3CN) of **13**.

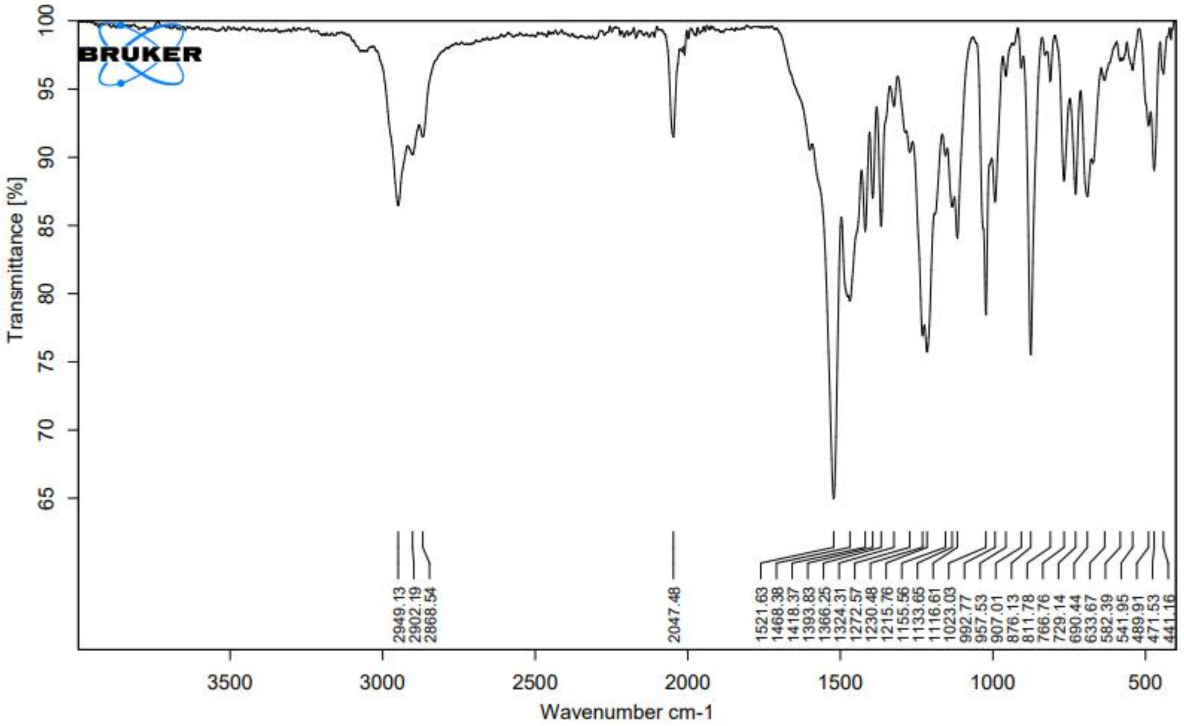

Figure S118: IR spectrum (neat) of **13**.

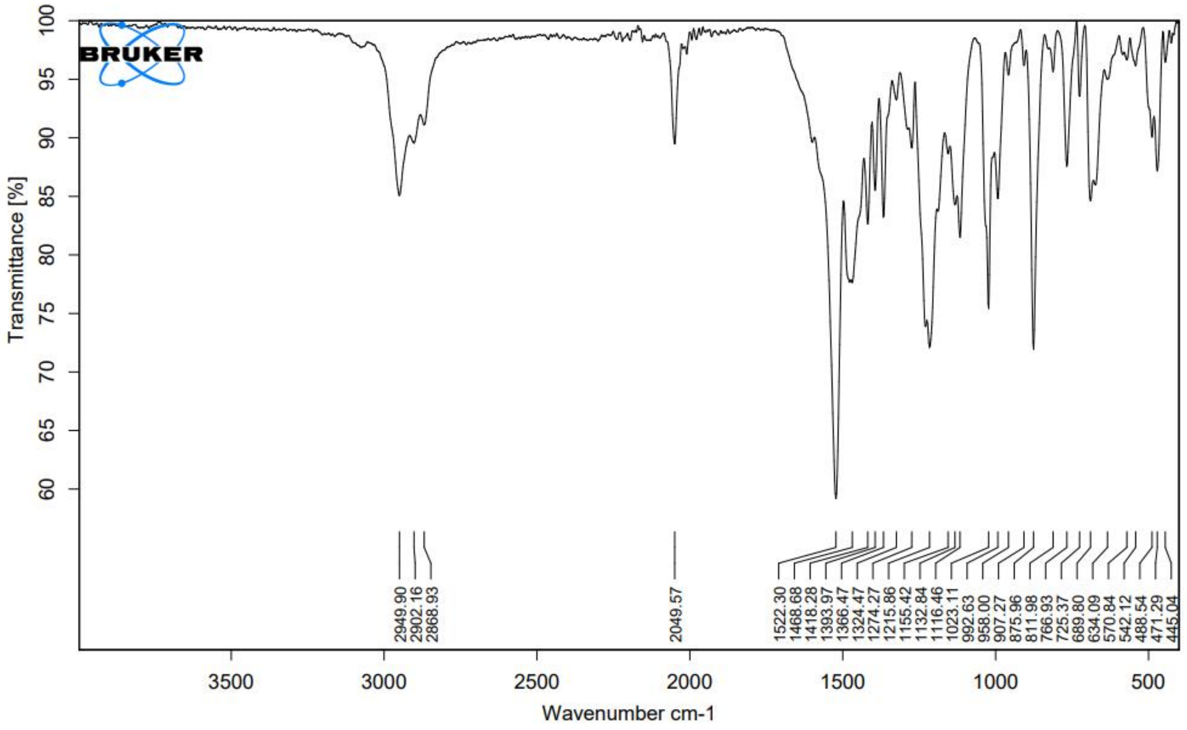

Figure S119: IR spectrum (DCM) of **13**.

[Au((CC)PMe(NIdipp)*t*Bu)$_2$][Cl] **14**

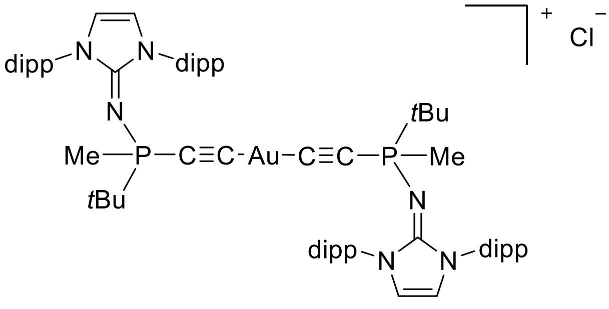

Au(tht)Cl (6 mg, 0.0189 mmol, 1.00 eq.) was added to a solution of **10** (20 mg, 0.0378 mmol, 2.00 eq.) in toluene (3 mL). The reaction mixture was stirred for 1 h, where a suspension was formed. The absence of phosphorus species in the supernatant was confirmed by ^{31}P NMR, indicating a complete reaction. The reaction mixture was filtered, and the residue was washed with hexane (3 mL). The residue was dried *in vacuo*. **14** was obtained as a white solid (15 mg, 0.0116 mmol, 61%).

^{1}H NMR (400 MHz, CD_3CN) δ (ppm) = 7.56 - 7.52 (m, 2H, Ar-H), 7.43 - 7.37 (m, 4H, Ar-H), 7.11 (s, 2H, NCHCHN), 2.96 (hept, J_{HH} = 6.7 Hz, 2H, CH (*i*Pr)), 2.77 (hept, J_{HH} = 6.7 Hz, 2H, CH (*i*Pr)), 1.41 (d, J_{HH} = 6.8 Hz, 6H, CH_3 (*i*Pr)), 1.35 (d, J_{HH} = 6.8 Hz, 6H, CH_3 (*i*Pr)), 1.22 (d, J_{HH} = 6.9 Hz, 6H, CH_3 (*i*Pr)), 1.17 (d, J_{HH} = 6.9 Hz, 6H, CH_3 (*i*Pr)), 0.70 (d, $^3J_{HP}$ = 18.3 Hz, 9H, CH_3 (*t*Bu)), 0.52 (d, $^2J_{HP}$ = 13.0 Hz, 3H, CH_3 (Me)).

^{1}H{^{31}P} NMR (400 MHz, CD_3CN) δ (ppm) = 7.56 - 7.52 (m, 2H, Ar-H), 7.43 - 7.37 (m, 4H, Ar-H), 7.10 (s, 2H, NCHCHN), 2.96 (hept, $^3J_{HH}$ = 6.8 Hz, 2H, CH (*i*Pr)), 2.77 (hept, $^3J_{HH}$ = 6.8 Hz, 2H, CH (*i*Pr)), 1.41 (d, $^3J_{HH}$ = 6.8 Hz, 6H, CH_3 (*i*Pr)), 1.35 (d, $^3J_{HH}$ = 6.8 Hz, 6H, CH_3 (*i*Pr)), 1.22 (d, $^3J_{HH}$ = 6.9 Hz, 6H, CH_3 (*i*Pr)), 1.18 (d, $^3J_{HH}$ = 6.9 Hz, 6H, CH_3 (*i*Pr)), 0.70 (s, 9H, CH_3 (*t*Bu)), 0.52 (s, 3H, CH_3 (Me)).

^{13}C{^{1}H} NMR (101 MHz, CD_3CN) δ (ppm) = 163.5 (dd, $^2J_{CP}$ = 14 Hz, $^4J_{CP}$ = 3 Hz, βC (Au-CC)), 148.0 (d, $^2J_{CP}$ = 24 Hz, NCN), 147.9 (s, q-C (Ar-*i*Pr) (overlays with multiplet of CN, can be proven by NMR in $CDCl_3$)), 132.8 (s, ipso-C (Ar)), 131.9 (s, C (Ar-H)), 125.6 (s, C (Ar-H)), 125.3 (s, C (Ar-H)), 119.5 (s, C (NCHCHN), 86.7 (d, $^1J_{CC}$ = 142 Hz, αC (P-CC)), 33.1 (d, $^1J_{CP}$ = 96 Hz, q-C (*t*Bu)), 29.5 (d, J_{CP} = 8 Hz, CH (*i*Pr)), 25.6 (s, CH_3 (*i*Pr)), 25.3 (s, CH_3 (*i*Pr)), 24.0 (s, CH_3 (*i*Pr)), 23.7 (d, $^3J_{CP}$ = 2 Hz, CH_3 (*t*Bu)), 23.1 (s, J_{CP} = 2 Hz, CH_3 (*i*Pr)), 13.2 (dd, $^1J_{CP}$ = 73 Hz, $^7J_{CP}$ = 2 Hz, CH_3 (Me)).

^{31}P NMR (162 MHz, CD_3CN) δ (ppm) = 8.2 - 7.8 (m).

^{31}P{^{1}H} NMR (162 MHz, CD_3CN) δ (ppm) = 8.0 (s), 8.0 (s).

^{31}P{^{1}H} NMR (162 MHz, $CDCl_3$) δ (ppm) = 7.4 (s).

HRMS (ESI, CH_3CN) m/z = 1255.6807 ([M-Cl]$^+$, calculated: 1255.6832).

IR (neat): ν_{max}/cm^{-1} = 2961.10, 2928.09, 2868.22, 2051.82 (C≡C), 1600.63, 1578.98, 1562.65, 1460.01, 1424.19, 1385.57, 1363.74, 1329.32, 1291.01, 1273.06, 1211.04, 1181.45, 1129.52, 1092.74, 1067.68, 1042.20, 1014.95, 973.75, 931.89, 886.39, 876.99, 800.54, 762.63, 723.75, 633.20, 602.31, 524.30, 495.47, 450.13, 426.94, 410.66.

IR (in DCM): ν_{max}/cm^{-1} = 2964.47, 2929.29, 2869.05, 2054.79 (C≡C), 1604.96, 1581.23, 1565.08, 1465.34, 1426.02, 1385.67, 1364.83, 1348.55, 1329.52, 1292.80, 1273.85, 1254.86, 1213.55, 1180.77, 1129.99, 1094.21, 1069.34, 1042.27, 1014.46, 975.26, 933.97, 885.81, 877.29, 801.42,

789.70, 761.67, 741.12, 723.92, 692.59, 636.99, 602.42, 578.52, 525.83, 495.64, 450.14, 427.19, 412.27.

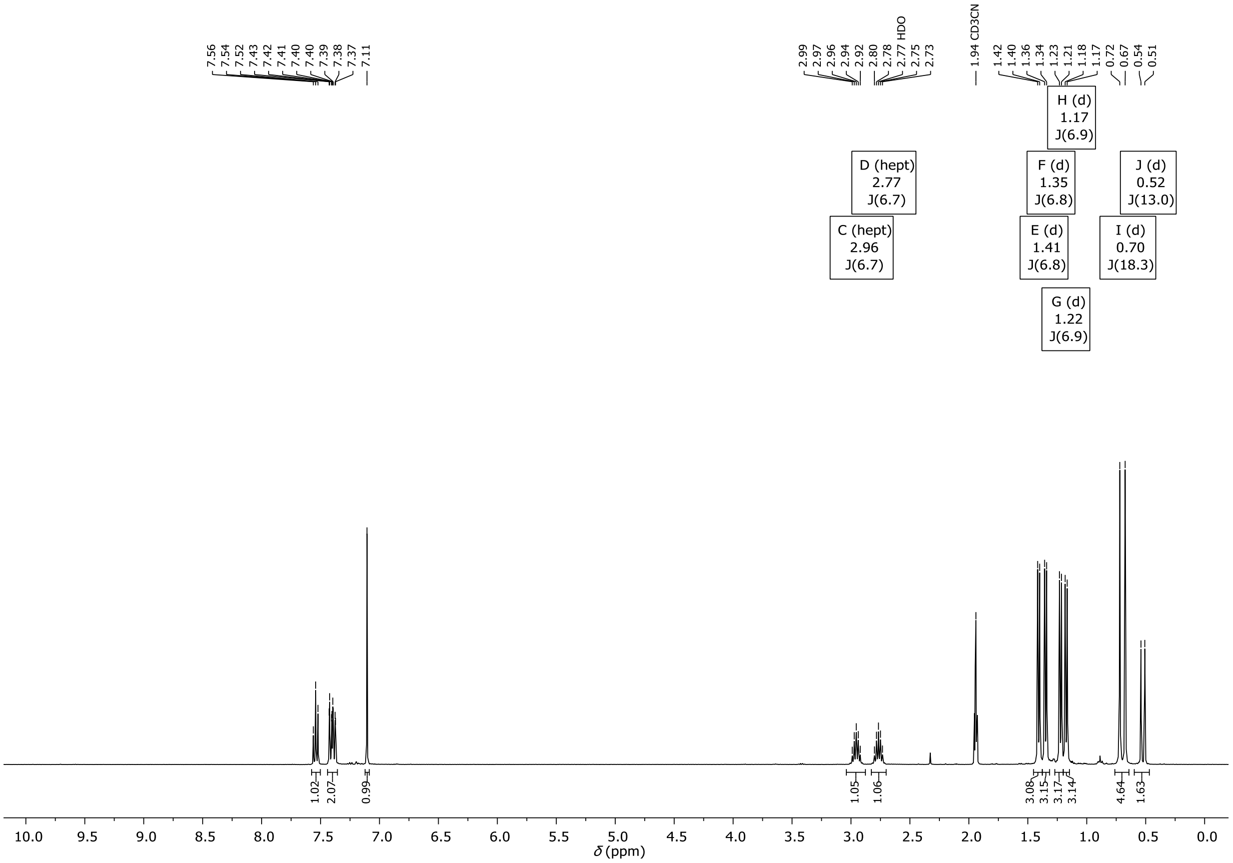

Figure S120: ^{1}H NMR spectrum (400 MHz, CD_3CN) of **14**.

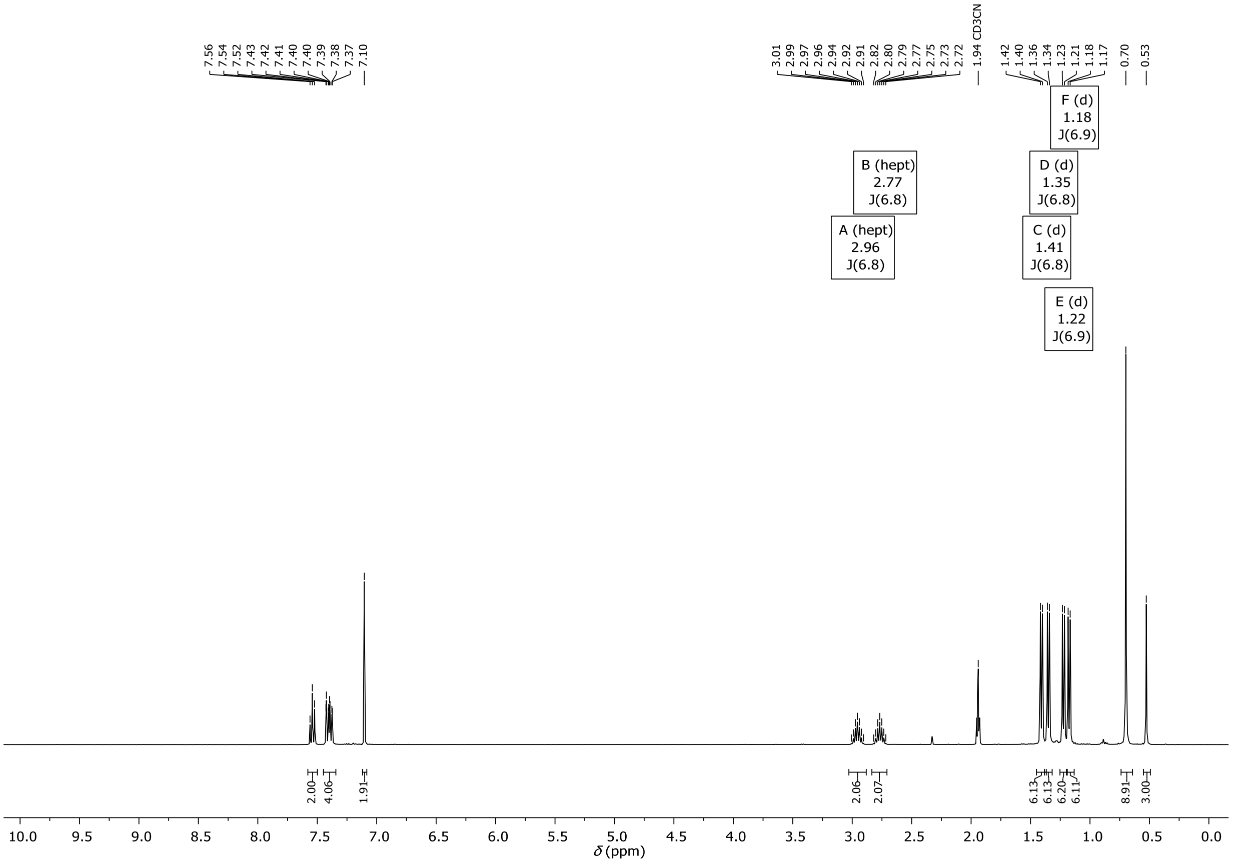

Figure S121: $^{1}H\{^{31}P\}$ NMR spectrum (400 MHz, CD_3CN) of **14**.

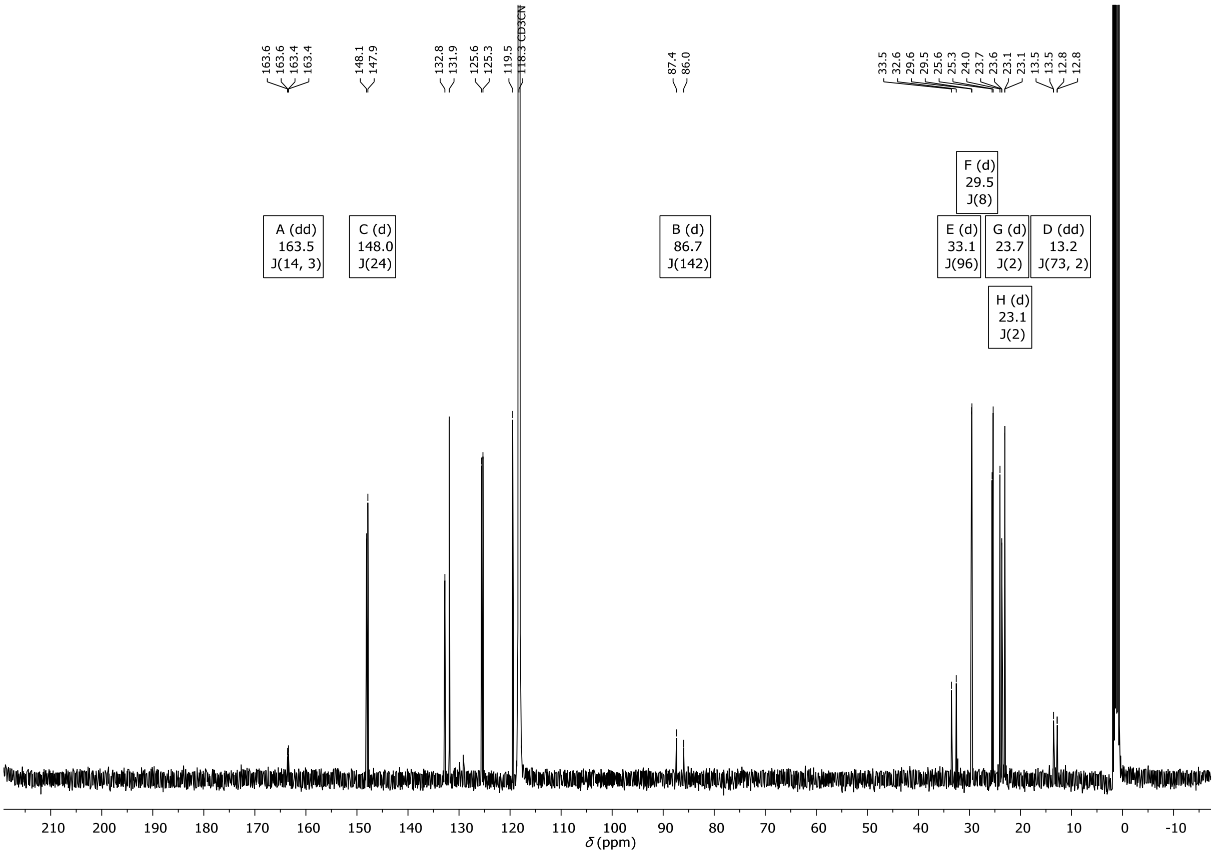

Figure S122: $^{13}C\{^{1}H\}$ NMR spectrum (101 MHz, CD_3CN) of **14**.

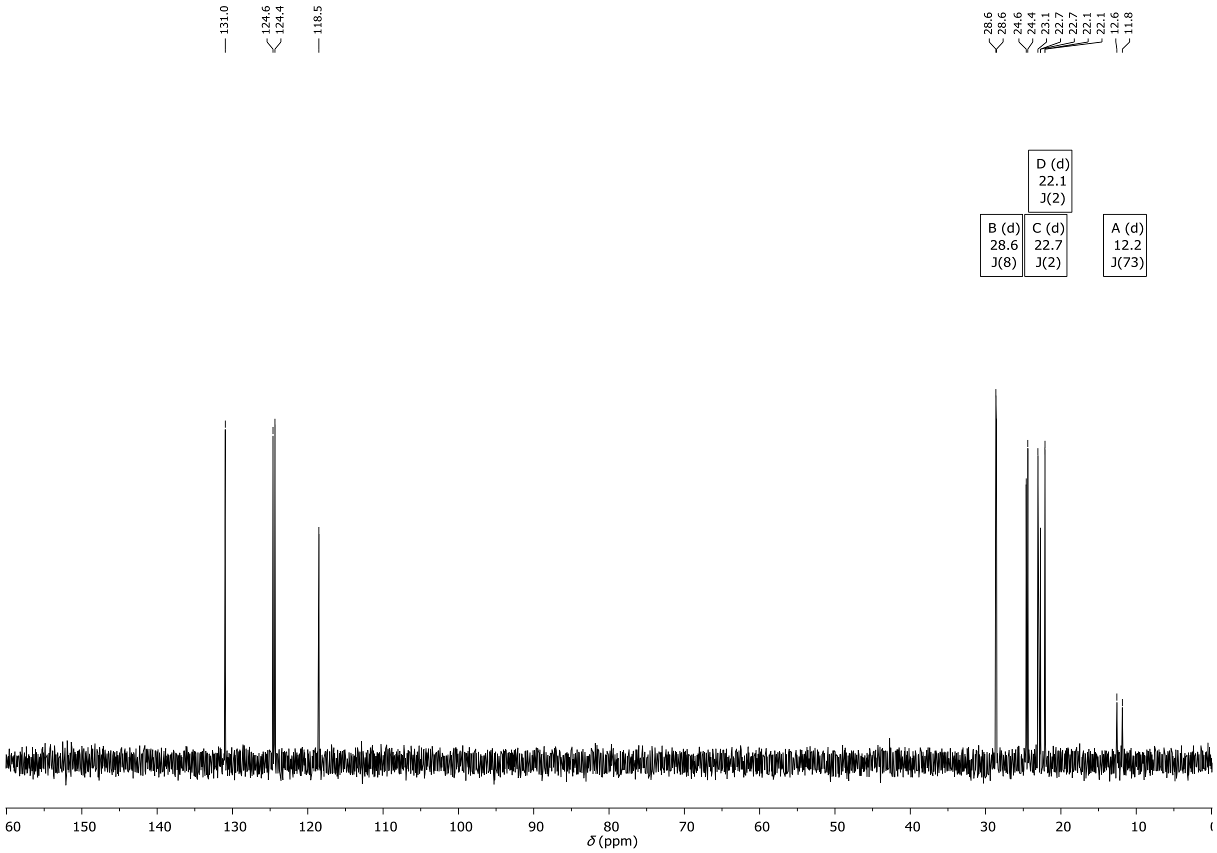

Figure S123: ^{13}C DEPT-135 spectrum (101 MHz, CD_3CN) of **14**.

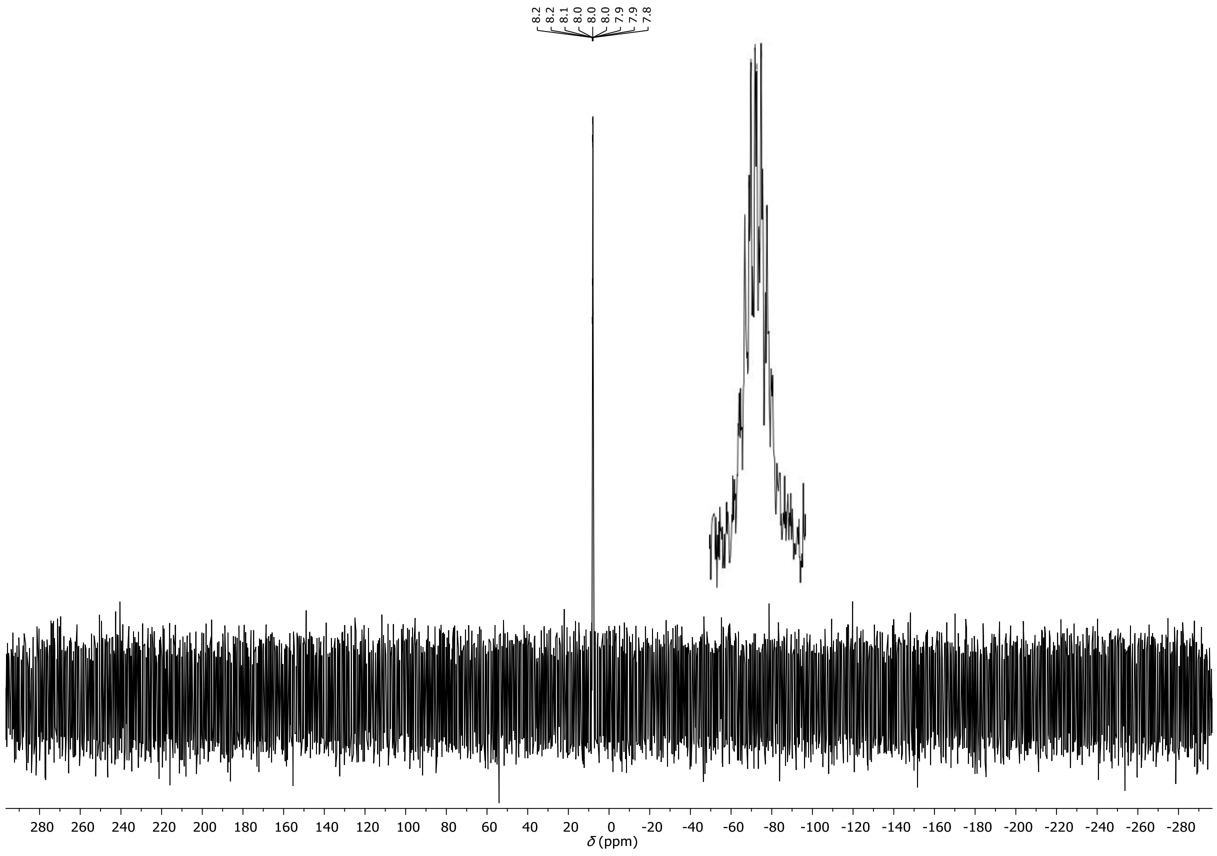

Figure S124: ^{31}P NMR spectrum (162 MHz, CD_3CN) of **14**.

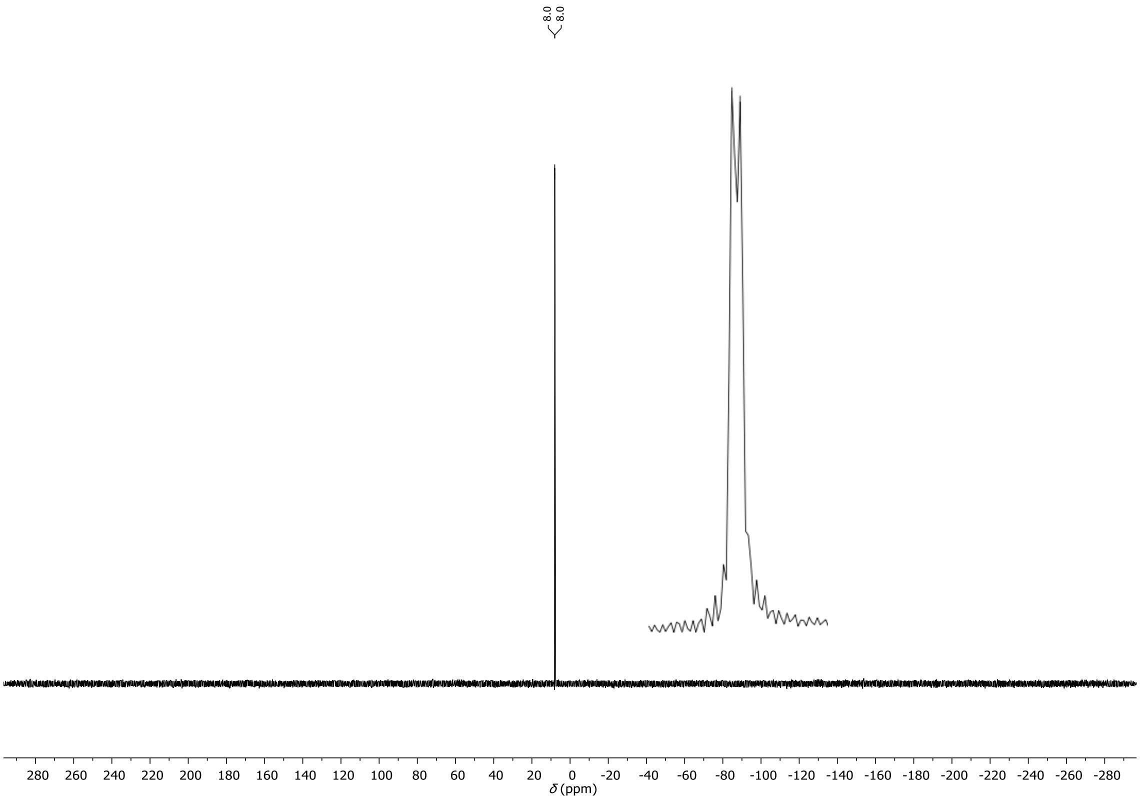

Figure S125: $^{31}P\{^{1}H\}$ NMR spectrum (162 MHz, CD_3CN) of **14**.

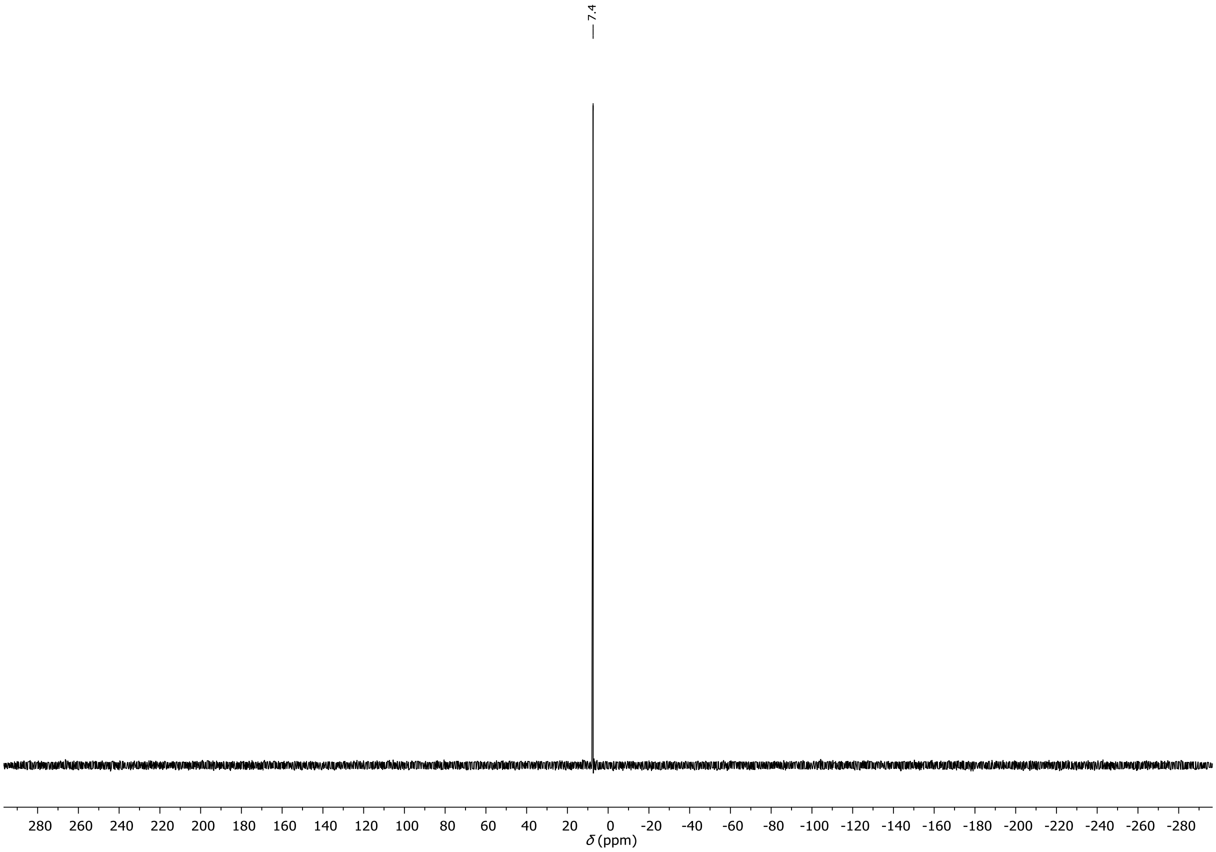

Figure S126: $^{31}P\{^{1}H\}$ NMR spectrum (162 MHz, $CDCl_3$) of **14**.

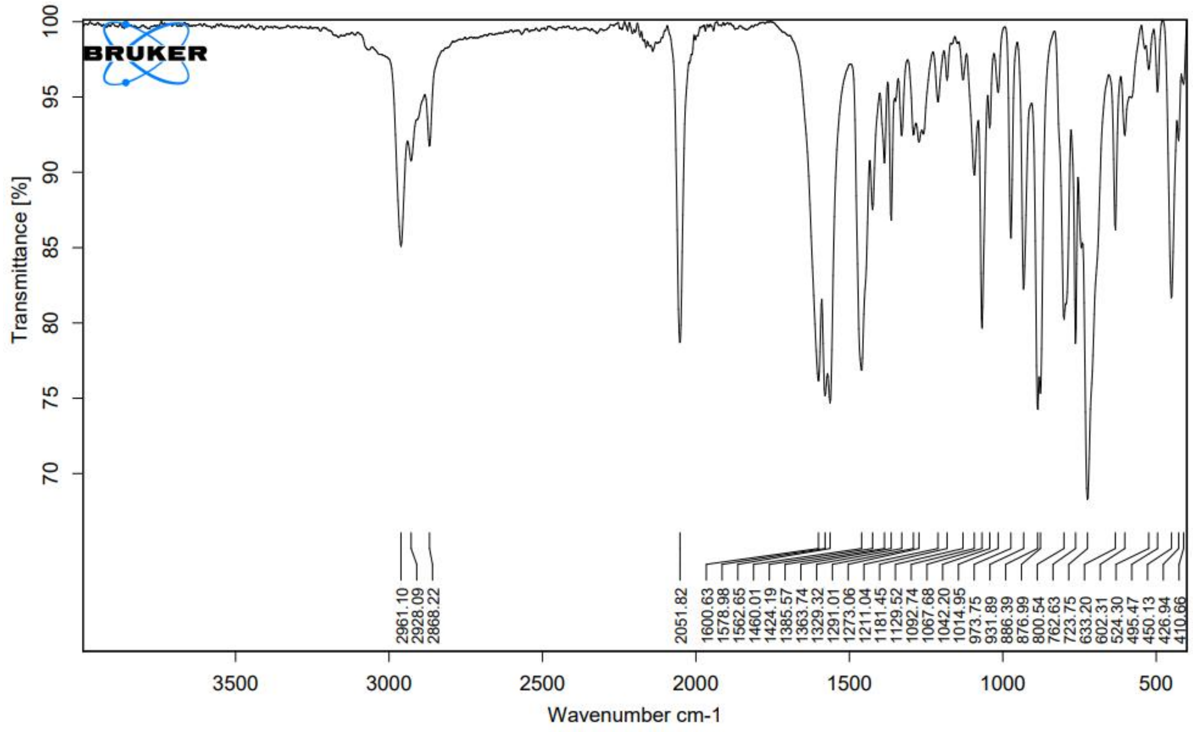

Figure S127: IR spectrum (neat) of **14**.

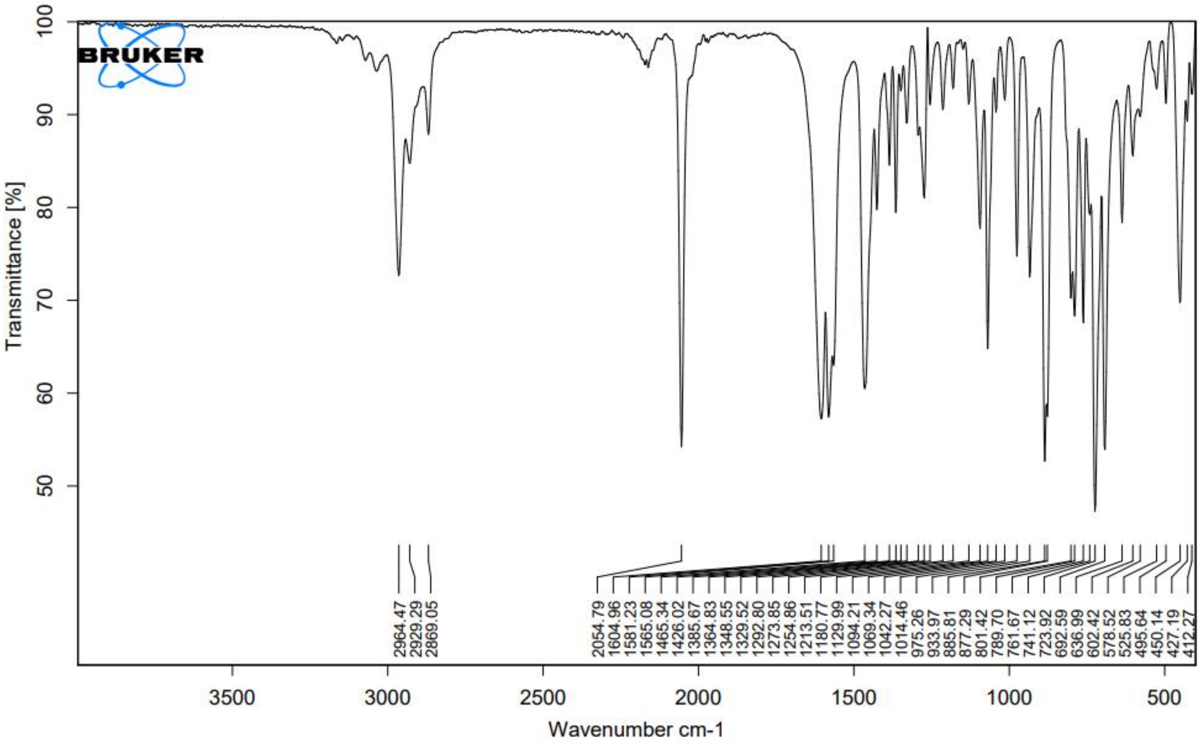

Figure S128: IR spectrum (DCM) of **14**.

Reaction of **6** with [AuCl(*i*Pr$_2$-bimy)]

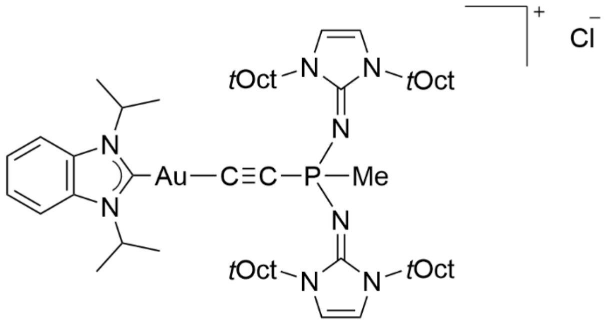

15

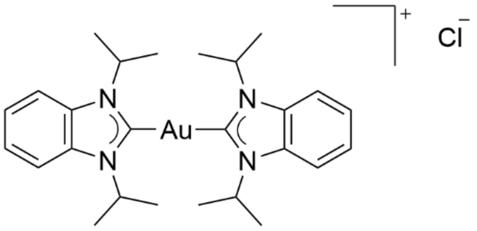

[Au(*i*Pr$_2$-bimy)$_2$][Cl]

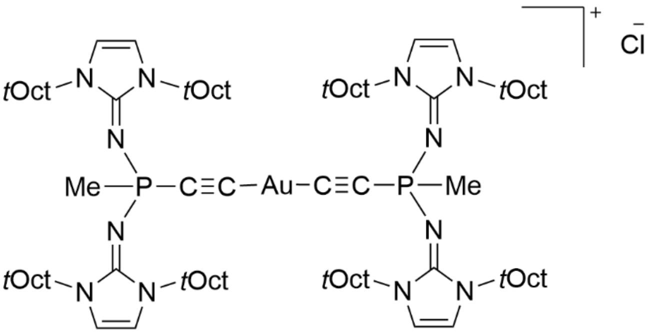

13

Figure S129: Reaction products of the reaction with **6** and [ClAu(*i*Pr$_2$-bimy)].

[ClAu(*i*Pr$_2$-bimy)] (5 mg, 0.0115 mmol, 1 eq.) and **6** (7 mg, 0.0115 mmol, 1 eq.) were dissolved in THF (1 mL). A color change to red was observed instantly. All volatiles were removed *in vacuo*. The residue was examined with ^{1}H, ^{13}C and ^{31}P NMR revealing a mixture of heteroleptic and homoleptic Au(I) complexes (Figure S129).

[Au(*i*Pr$_2$-bimy)$_2$][Cl] was identified by comparison of literature reported [Au(*i*Pr$_2$-bimy)$_2$][Br][4] of the ^{13}C NMR signals. Residual [ClAu(*i*Pr$_2$-bimy)] was identified by comparison of literature reported data.[5]

^{1}H NMR (400 MHz, $CDCl_3$) δ (ppm) = 7.78 - 7.73 (m, 4H, Ar-H (NHC), [Au(*i*Pr$_2$-bimy)$_2$][Cl]), 7.65 - 7.63 (m, 2H+2H, Ar-H (NHC), [ClAu(*i*Pr$_2$-bimy)]+**15**), 7.49 - 7.45 (m, 4H, Ar-H (NHC), [Au(*i*Pr$_2$-bimy)$_2$][Cl]), 7.38 - 7.35 (m, 2H+2H, Ar-H (NHC), [ClAu(*i*Pr$_2$-bimy)]+**15**), 6.78 (s, 4H, NCHCHN, **15**), 6.68 (s, 8H, NCHCHN, **13**), 5.50 (hept, J_{HH} = 7.0 Hz, 2H, CH (*i*Pr (NHC), [ClAu(*i*Pr$_2$-bimy)])), 5.42 (hept, J_{HH} = 7.0 Hz, 4H, CH (*i*Pr (NHC), [Au(*i*Pr$_2$-bimy)$_2$][Cl])), 5.27 (hept, J_{HH} = 7.0 Hz, 2H, CH (*i*Pr (NHC), **15**)), 2.21 - 2.01 (m, 8H+16H, CH_2 (*t*Oct), **13**+**15**), 1.86 (d, J_{HH} = 7.2 Hz, 24H, CH_3 (*i*Pr (NHC), [Au(*i*Pr$_2$-bimy)$_2$][Cl])), 1.77 – 1.71 (m, 12H+54H+40H, CH_3 (*i*Pr (NHC) + $C(CH_3)_2$ (*t*Oct) + PCH_3, [ClAu(*i*Pr$_2$-bimy)]+**13**+**15**), 0.94 (s, 36H, $C(CH_3)_3$ (*t*Oct), **15**), 0.89 (s, 36H, $C(CH_3)_3$ (*t*Oct), **13**).

^{13}C{^{1}H} NMR (101 MHz, $CDCl_3$) δ (ppm) = 186.9 (s, $C_{Carbene}$ (NHC), [Au(*i*Pr$_2$-bimy)$_2$][Cl]), 143.9 (d, $^{2}J_{CP}$ = 16 Hz, NCN (NHI), **13**), 132.8 (s, C (Ar, NHC), [Au(*i*Pr$_2$-bimy)$_2$][Cl]), 125.0 (s, C (Ar, NHC), [Au(*i*Pr$_2$-bimy)$_2$][Cl]), 124.3 (s, C (Ar, NHC), **15**), 124.0 (s, C (Ar, NHC), [ClAu(*i*Pr$_2$-bimy)]), 113.3 (s, C (Ar, NHC), [Au(*i*Pr$_2$-bimy)$_2$][Cl]), 113.2 (s, C (Ar, NHC), **15**), 112.9 (s, C (Ar, NHC), [ClAu(*i*Pr$_2$-

bimy)]), 112.3 (s, C (NCHCHN (NHI), **15**), 112.0 (s, C (NCHCHN (NHI), **13**), 62.1 (s, q$C(CH_3)_2$) (*t*Oct), **15**), 61.9 (s, q$C(CH_3)_2$ (*t*Oct), **13**), 54.5 (s, CH (*i*Pr, NHC),), [ClAu(*i*Pr_2-bimy)]+**15**), 54.0 (s, CH (*i*Pr, NHC), [Au(*i*Pr_2-bimy)$_2$][Cl]), 51.4 (s, CH_2 (*t*Oct), **15**), 51.2 (s, CH_2 (*t*Oct)), **13**), 32.1 (s, q$C(CH_3)_3$ (*t*Oct), **15**), 32.0 (s, q$C(CH_3)_3$) (*t*Oct), **13**), 31.4 (s, $(CH_3)_3$ (*t*Oct), **13**), 31.4 (s, $(CH_3)_3$ (*t*Oct), **15**), 30.8 (d, $^5J_{CP}$ = 19 Hz, $(CH_3)_2$ (*t*Oct), **13**), 23.6 (s, CH_3 (*i*Pr, NHC), **15**), 22.8 (s, CH_3 (*i*Pr, NHC), [Au(*i*Pr_2-bimy)$_2$][Cl]), 22.1 (d, $^1J_{CP}$ = 64 Hz, CH_3 (Me), **13**), 21.5 (s, CH_3 (*i*Pr, NHC), [ClAu(*i*Pr_2-bimy)]).

Note that some of the quaternary carbons are not found in ^{13}C NMR due to low concentration in the mixture and coupling to phosphorus.

^{31}P NMR (162 MHz, $CDCl_3$) δ (ppm) = −34.4 (q, $^2J_{HP}$ = 13 Hz, **13**), −35.2 (q, $^2J_{HP}$ = 14 Hz, **15**).

^{31}P{^{1}H} NMR (162 MHz, $CDCl_3$) δ (ppm) = −34.4 (s, **13**), −35.2 (s, **15**).

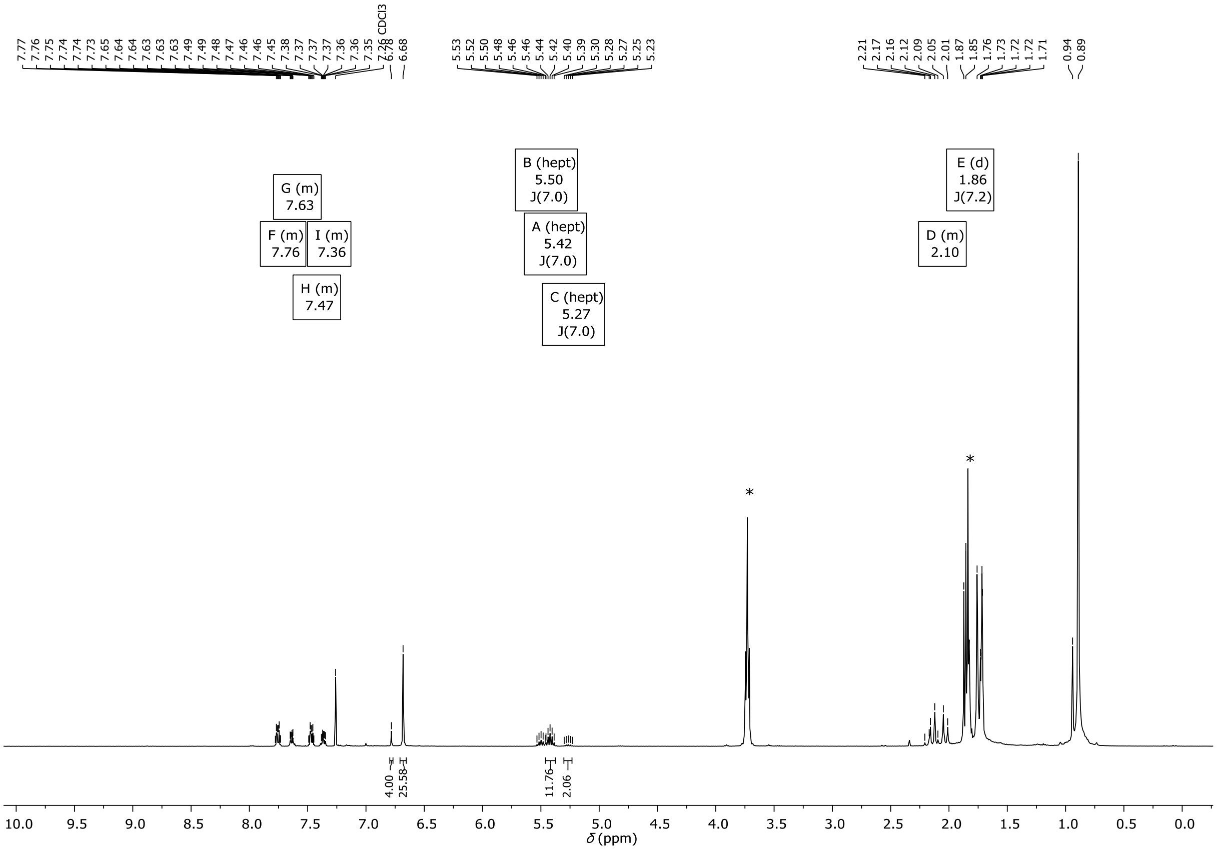

Figure S130: ^{1}H NMR spectrum (400 MHz, $CDCl_3$) of the reaction with **6** and [ClAu(*i*Pr_2-bimy)]. * marks residual THF.

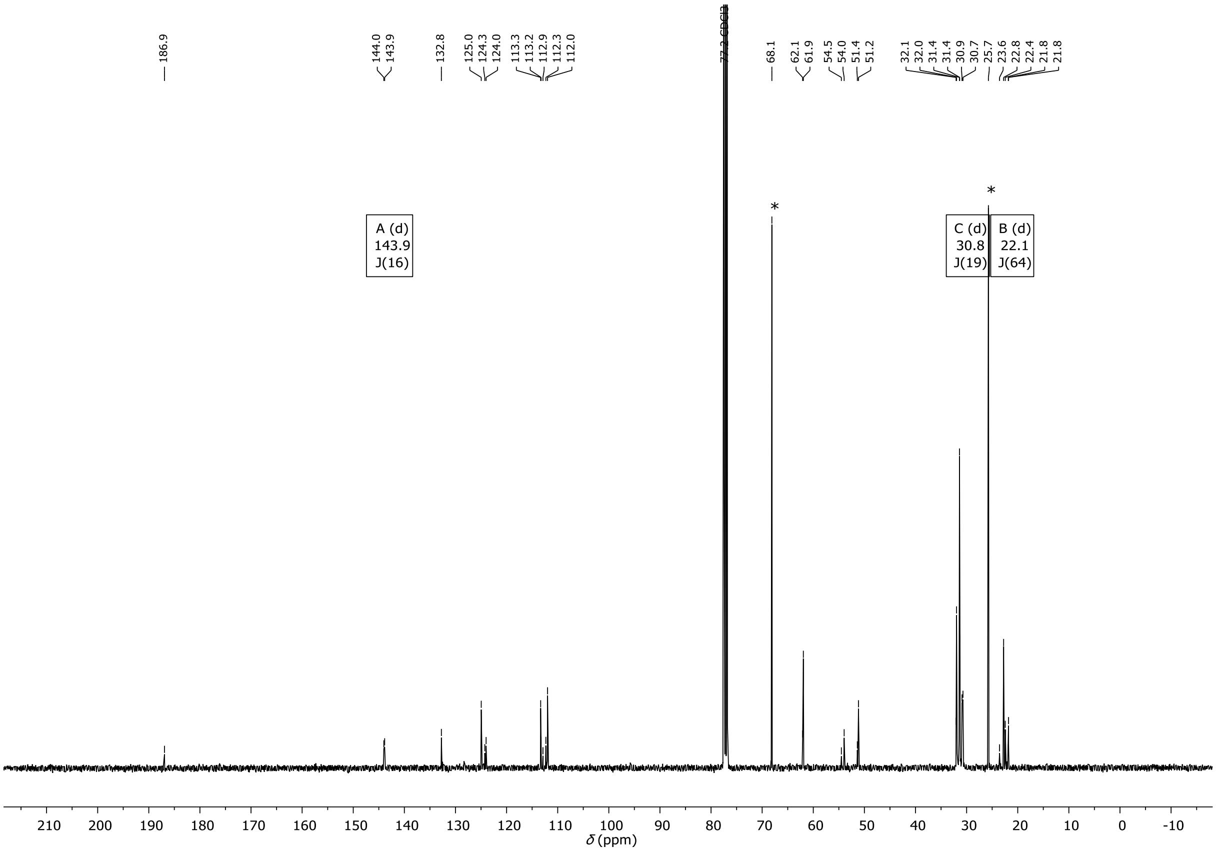

Figure S131: $^{13}C\{^{1}H\}$ NMR spectrum (101 MHz, $CDCl_3$) of the reaction with **6** and [ClAu(*i*Pr_2-bimy)]. * marks residual THF.

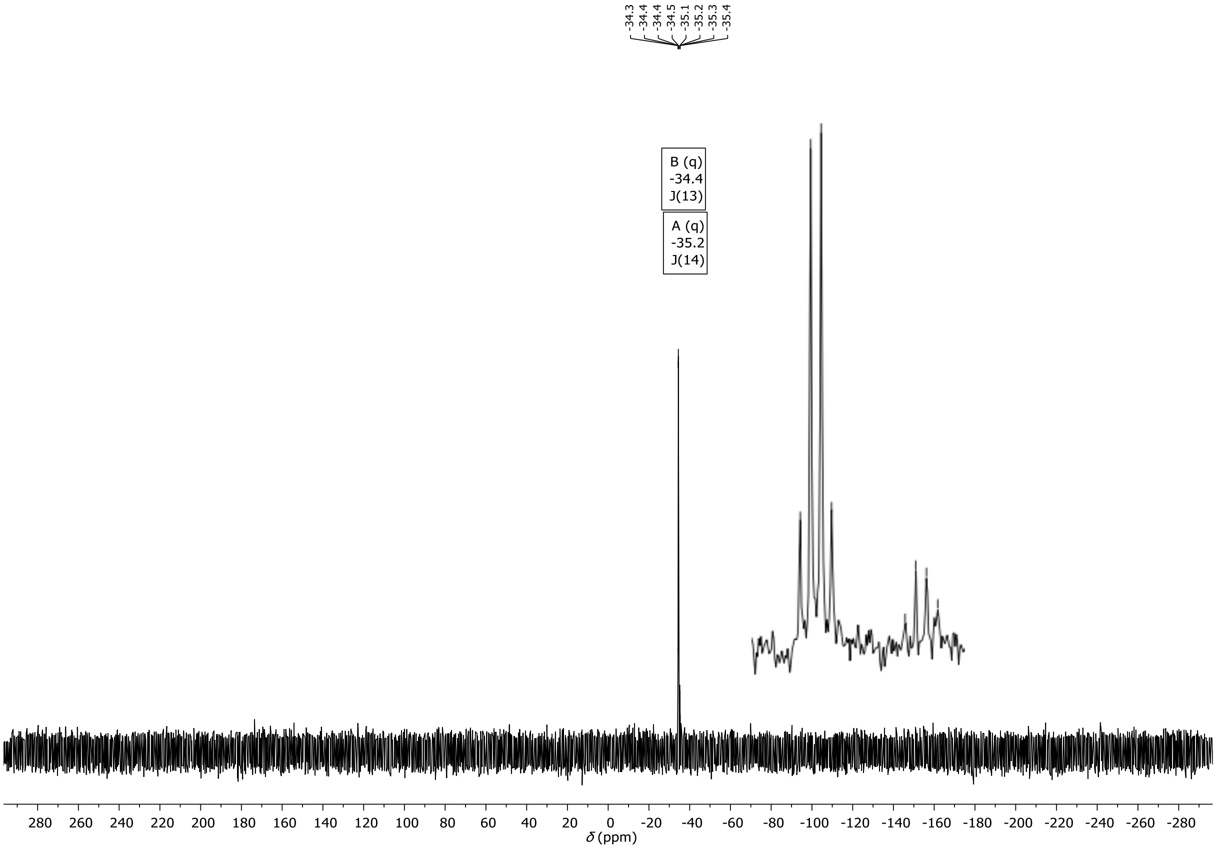

Figure S132: ^{31}P NMR spectrum (162 MHz, $CDCl_3$) of the reaction with **6** and [ClAu(*i*Pr_2-bimy)].

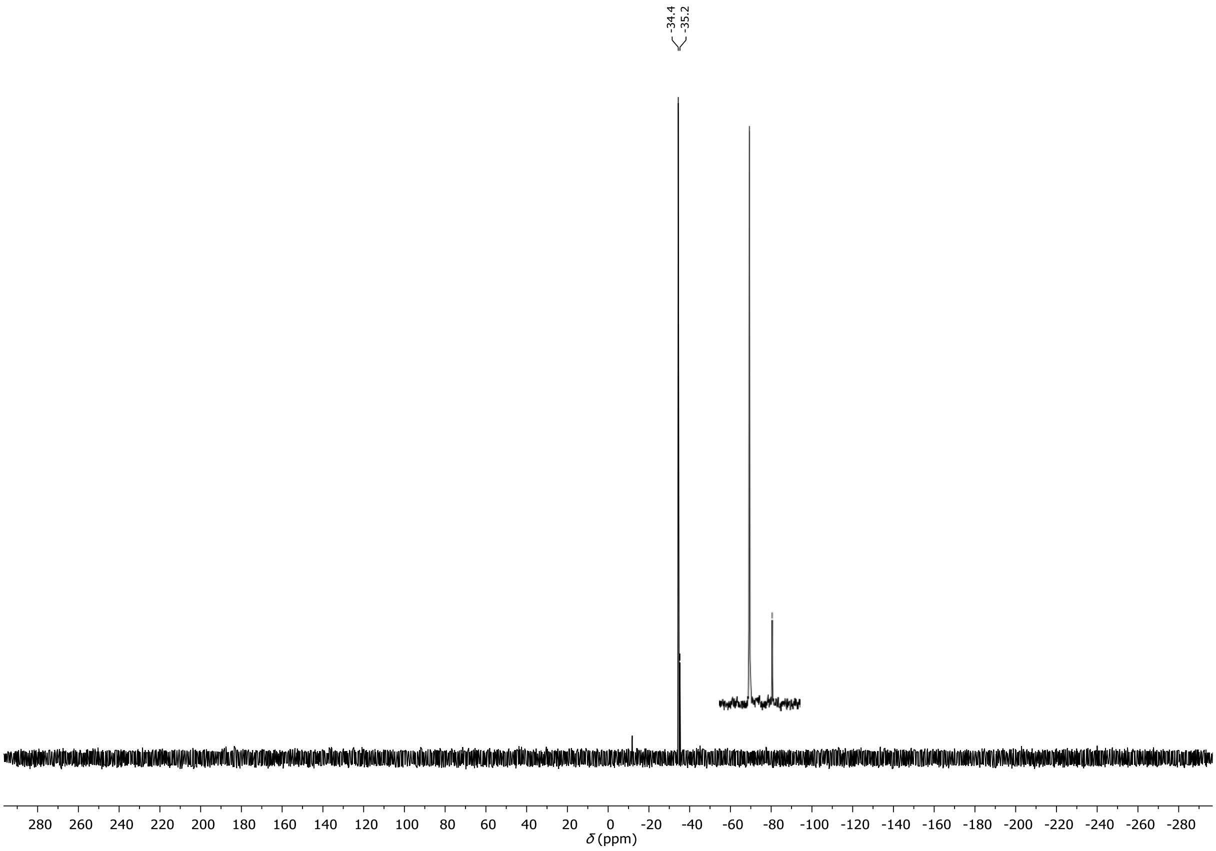

Figure S133: $^{31}P\{^1H\}$ NMR spectrum (162 MHz, $CDCl_3$) of the reaction with **6** and [ClAu(*i*Pr_2-bimy)].

Determination of the Huynh Electronic Parameter (HEP) for phosphonioacetlyide **10**: Reaction of **10** with [AuCl(*i*Pr_2-bimy)]

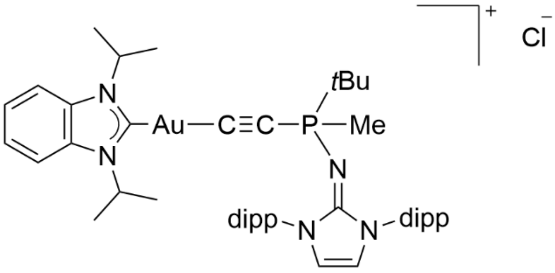

16

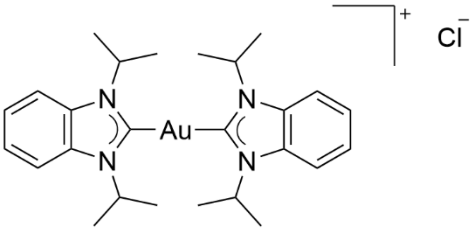

[Au(*i*Pr$_2$-bimy)$_2$][Cl]

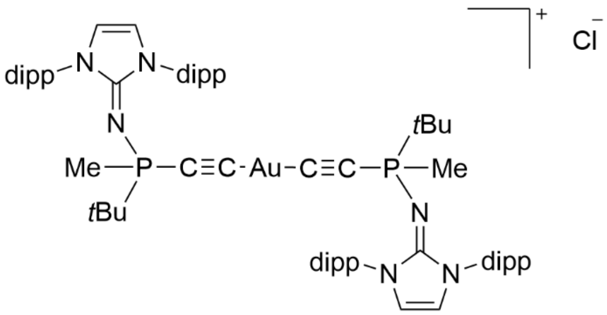

14

Figure S134: Reaction products of the reaction with **10** and [ClAu(*i*Pr_2-bimy)].

[ClAu(iPr$_2$-bimy)] (9 mg, 0.0207 mmol, 1 eq.) and **10** (11 mg, 0.0207 mmol, 1 eq.) were dissolved in THF (1 mL). A white precipitation formed instantly. The supernatant was decanted and the residue was dried *in vacuo*. The residue was examined with ^{1}H, ^{13}C and ^{31}P NMR, which confirms the formation of the gold(I) complexes [Au(**10**)$_2$]Cl (**14**), [Au(**10**)(iPr$_2$-bimy)]Cl (**16**) and [Au(iPr$_2$-bimy)$_2$]Cl[4] according to their indicative ^{1}H and ^{13}C NMR resonances (Figure S134). The ^{31}P{^{1}H} NMR spectrum further confirms the formation of **14** and **16**. (Note that the two diastereomers of **14** appear as two separate resonances in CD_3CN (8.0 ppm, 8.0 ppm) and as one signal in $CDCl_3$ (7.4 ppm); see Figure S139 and S140). Note that upon exchange of chloride with PF_6^- counteranions using either KPF_6 or $TlPF_6$, the same product mixture was obtained. Attempts to separate the complexes by column chromatography (alumina, CH_2Cl_2/MeOH) or recrystallization (THF) were unsuccessful. However, single crystals suitable for an SCXRD analysis were obtained, which reveal the literature-known homoleptic complex [Au(iPr$_2$-bimy)$_2$]PF$_6$ and the desired complex [Au(**10**)(iPr$_2$-bimy)]PF$_6$ **16'**.

See above for the NMR data for the isolated complex **14**. The NMR data for complex [Au(iPr$_2$-bimy)$_2$]Br is reported in reference[4]. Assignment of the resonances of **14**, **16** and [Au(iPr$_2$-bimy)$_2$]Cl in the mixture:

^{1}H NMR (400 MHz, $CDCl_3$) δ (ppm) = 7.77 - 7.74 (m, 4H, Ar-H (NHC), [Au(iPr$_2$-bimy)$_2$][Cl]), 7.68 - 7.66 (m, 4H, Ar-H (NHC), **16**), 7.54 - 7.29 (m, 4H+6H+4H, Ar-H (NHC+NHI), **14**+**16**+[Au(iPr$_2$-bimy)$_2$][Cl]), 6.94 (s, 2H, NCHCHN, **16**), 6.86 (s, 2H, NCHCHN, **14**), 5.41 (hept, J_{HH} = 7.0 Hz, 4H, CH (iPr (NHC), [Au(iPr$_2$-bimy)$_2$][Cl])), 5.32 (hept, J_{HH} = 7.0 Hz, 2H, CH (iPr (NHC), **16**)), 2.96 (hept, J_{HH} = 7.1 Hz, 2H+1H, CH (iPr (NHI), **14**+**16**)), 2.77 (hept, J_{HH} = 6.9 Hz, 2H+1H, CH (iPr (NHI), **14**+**16**)), 1.86 (d, J_{HH} = 6.9 Hz, 24H, CH_3 (iPr (NHC), [Au(iPr$_2$-bimy)$_2$][Cl])), 1.77 (d, J_{HH} = 7.0 Hz, 12H, CH_3 (iPr (NHC), **16**)), 1.43 (d, J_{HH} = 6.8 Hz, 3H, CH_3 (iPr (NHI), **16**)), 1.41 (d, J_{HH} = 6.6 Hz, 6H, CH_3 (iPr (NHI), **16**)), 1.37 (d, J_{HH} = 6.8 Hz, 3H, CH_3 (iPr (NHI), **16**)), 1.34 (d, J_{HH} = 6.8 Hz, 6H, CH_3 (iPr (NHI), **14**)), 1.24 (m, 6H+3H, CH_3 (iPr (NHI), **14**+**16**)), 1.19 (m, 6H+3H, CH_3 (iPr (NHI), **14**+**16**)), 0.77 (d, $^{3}J_{HP}$ = 18.5 Hz, 9H, CH_3 (tBu, **16**)), 0.70 (d, $^{3}J_{HP}$ = 18.3 Hz, 9H, CH_3 (tBu, **14**)), 0.58 (d, $^{2}J_{HP}$ = 12.9 Hz, 3H, CH_3 (Me, **16**)), 0.49 (d, $^{2}J_{HP}$ = 12.9 Hz, 3H, CH_3 (Me, **14**)).

^{1}H NMR (400 MHz, CD_3CN) δ (ppm) = 7.87 - 7.85 (m, 4H, Ar-H (NHC), [Au(iPr$_2$-bimy)$_2$][Cl]), 7.80 - 7.78 (m, 4H, Ar-H (NHC), **16**), 7.57 - 7.37 (m, 4H+6H+4H, Ar-H (NHC+NHI), **14**+**16**+[Au(iPr$_2$-bimy)$_2$][Cl]), 7.11 (s, 2H, NCHCHN, **16**), 7.11 (s, 2H, NCHCHN, **14**), 5.41 (hept, J_{HH} = 6.9 Hz, 4H, CH (iPr (NHC), [Au(iPr$_2$-bimy)$_2$][Cl])), 5.30 (hept, J_{HH} = 7.0 Hz, 2H, CH (iPr (NHC), **16**)), 2.97 (hept, J_{HH} = 6.9 Hz, 2H+1H, CH (iPr (NHI), **14**+**16**)), 2.79 (hept, J_{HH} = 6.9 Hz, 2H+1H, CH (iPr (NHI), **14**+**16**)), 1.81 (d, J_{HH} = 7.0 Hz, 24H, CH_3 (iPr (NHC), [Au(iPr$_2$-bimy)$_2$][Cl])), 1.74 (d, J_{HH} = 6.9 Hz, 12H, CH_3 (iPr (NHC), **14**)), 1.44 (d, J_{HH} = 6.8 Hz, 3H, CH_3 (iPr (NHI), **16**)), 1.40 (m, 6H+3H, CH_3 (iPr (NHI), **14**+**16**)), 1.35 (d, J_{HH} = 6.8 Hz, 6H, CH_3 (iPr (NHI), **14**)), 1.22 (m, 6H+3H, CH_3 (iPr (NHI), **14**+**16**)), 1.18 (m, 6H+3H, CH_3 (iPr (NHI), **14**+**16**)), 0.75 (d, $^{3}J_{HP}$ = 18.5 Hz, 9H, CH_3 (tBu, **16**)), 0.70 (d, $^{3}J_{HP}$ = 18.4 Hz, 9H, CH_3 (tBu, **14**)), 0.62 (d, $^{2}J_{HP}$ = 13.0 Hz, 3H, CH_3 (Me, **16**)), 0.52 (d, $^{2}J_{HP}$ = 13.0 Hz, 3H, CH_3 (Me, **14**)).

^{13}C{^{1}H} NMR (101 MHz, $CDCl_3$) δ (ppm) = 188.9 (d, $^{4}J_{CP}$ = 3 Hz, $C_{Carbene}$ (NHC), **16**), 186.9 (s, $C_{Carbene}$ (NHC), [Au(iPr$_2$-bimy)$_2$][Cl]), 148.0 (m, NCN + q-C (Ar-iPr, NHI), **14**+**16**), 132.7 (s, C (Ar, NHC), [Au(iPr$_2$-bimy)$_2$][Cl]), 132.7 (s, C (Ar, NHC), **16**), 131.7 (s, ipso-C (Ar, NHI), **14**), 131.7 (s,

ipso-C (Ar, NHI), **16**), 131.4 (s, C (Ar-H, NHI), **16**), 131.3 (s, C (Ar-H, NHI), **14**), 125.0 (s, C (Ar, NHC), [Au(*i*Pr_2-bimy)$_2$][Cl]), 124.8 (s, C (Ar, NHC), **16**), 124.8 (s, C (Ar-H, NHI), **14**), 124.7 (s, C (Ar-H, NHI), **16**), 124.6 (s, C (Ar-H, NHI), **14**), 124.5 (s, C (Ar-H, NHI), **16**), 118.5 (s, C (NCHCHN), **16**), 118.2 (s, C (NCHCHN, **14**), 113.3 (s, C (Ar, NHC), [Au(*i*Pr_2-bimy)$_2$][Cl]), 113.2 (s, C (Ar, NHC), **16**), 54.0 (s, CH (*i*Pr, NHC), [Au(*i*Pr_2-bimy)$_2$][Cl]), 53.8 (s, CH (*i*Pr, NHC), **16**), 32.7 (d, $^1J_{CP}$ = 95 Hz, q-C (*t*Bu), **14**+**16**), 28.9 (m, J_{CP} = 8 Hz, CH (*i*Pr, NHI), **14**+**16**), 25.7 (s, CH_3 (*i*Pr, NHI), **14**+**16**), 25.3 (s, CH_3 (*i*Pr, NHI), **14**+**16**), 23.8 (s, CH_3 (*i*Pr, NHI), **14**+**16**), 23.5 (s, J_{CP} = 2 Hz, CH_3 (*i*Pr, NHI), **14**+**16**), 23.1 (d, $^3J_{CP}$ = 2 Hz, CH_3 (*t*Bu), **16**), 23.0 (s, CH_3 (*t*Bu), **14**), 23.1 (s, CH_3 (*i*Pr, NHC), [Au(*i*Pr_2-bimy)$_2$][Cl]), 22.4 (s, CH_3 (*i*Pr, NHC), **16**), 12.8 (d, $^1J_{CP}$ = 73 Hz, CH_3 (Me), **16**).

Note that some of the quaternary carbons are not found in ^{13}C NMR due to low concentration in the mixture and coupling to phosphorus.

^{31}P NMR (162 MHz, $CDCl_3$) δ (ppm) = 7.5 (m, **14**+**16**).

$^{31}P\{^1H\}$ NMR (162 MHz, $CDCl_3$) δ (ppm) = 7.5 (s, **16**), 7.4 (s, **14**).

$^{31}P\{^1H\}$ NMR (162 MHz, CD_3CN) δ (ppm) = 8.0 (s, **14**), 8.0 (s, **14**), 8.0 (s, **16**).

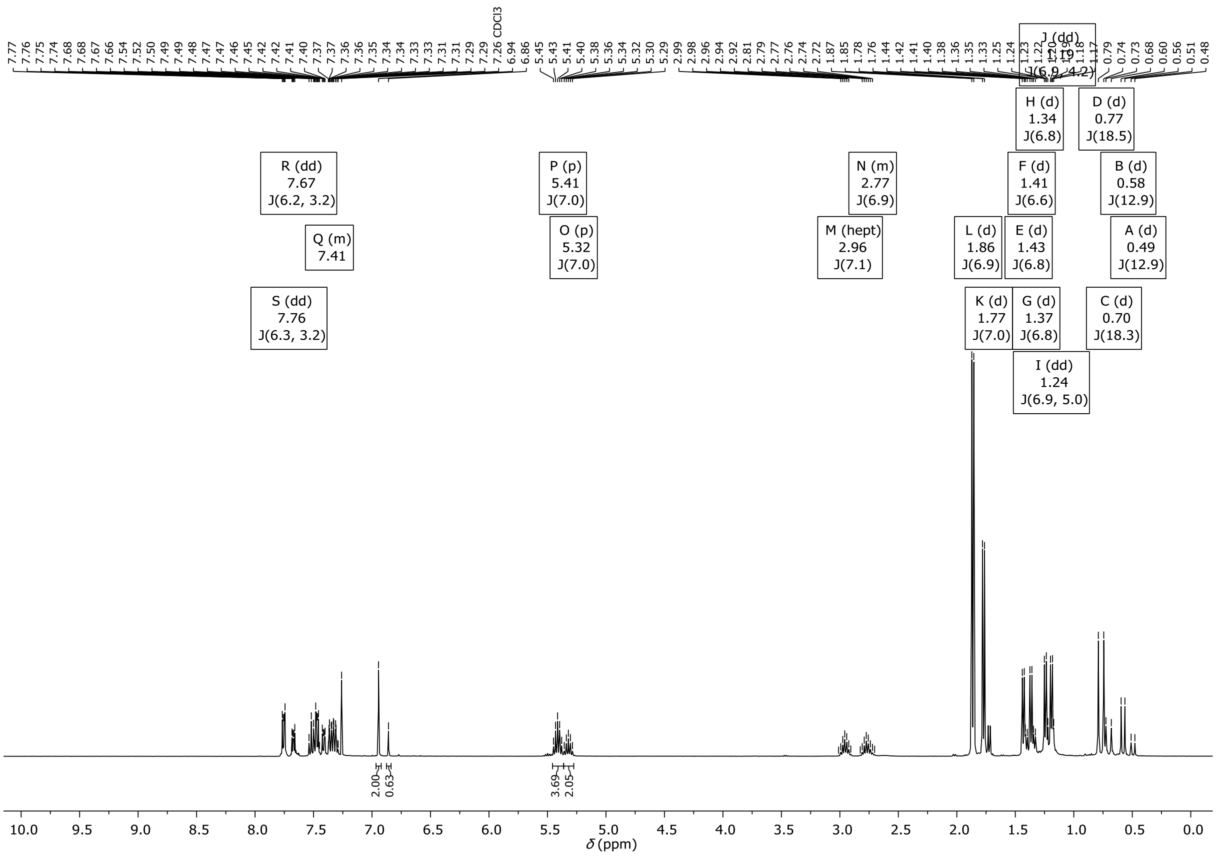

Figure S135: ^1H NMR spectrum (400 MHz, $CDCl_3$) of the reaction with **10** and [ClAu(*i*Pr_2-bimy)].

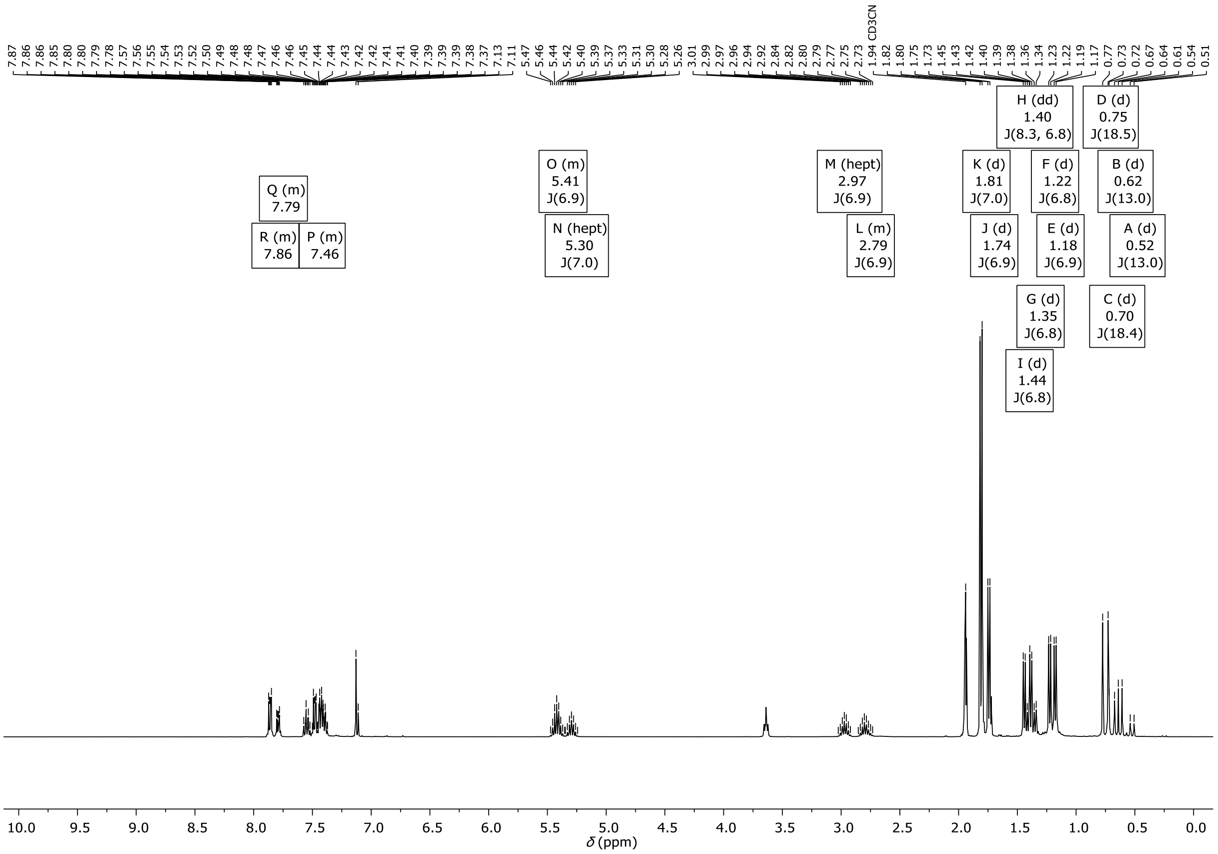

Figure S136: ^{1}H NMR spectrum (400 MHz, CD_3CN) of the reaction with **10** and [ClAu(*i*Pr_2-bimy)].

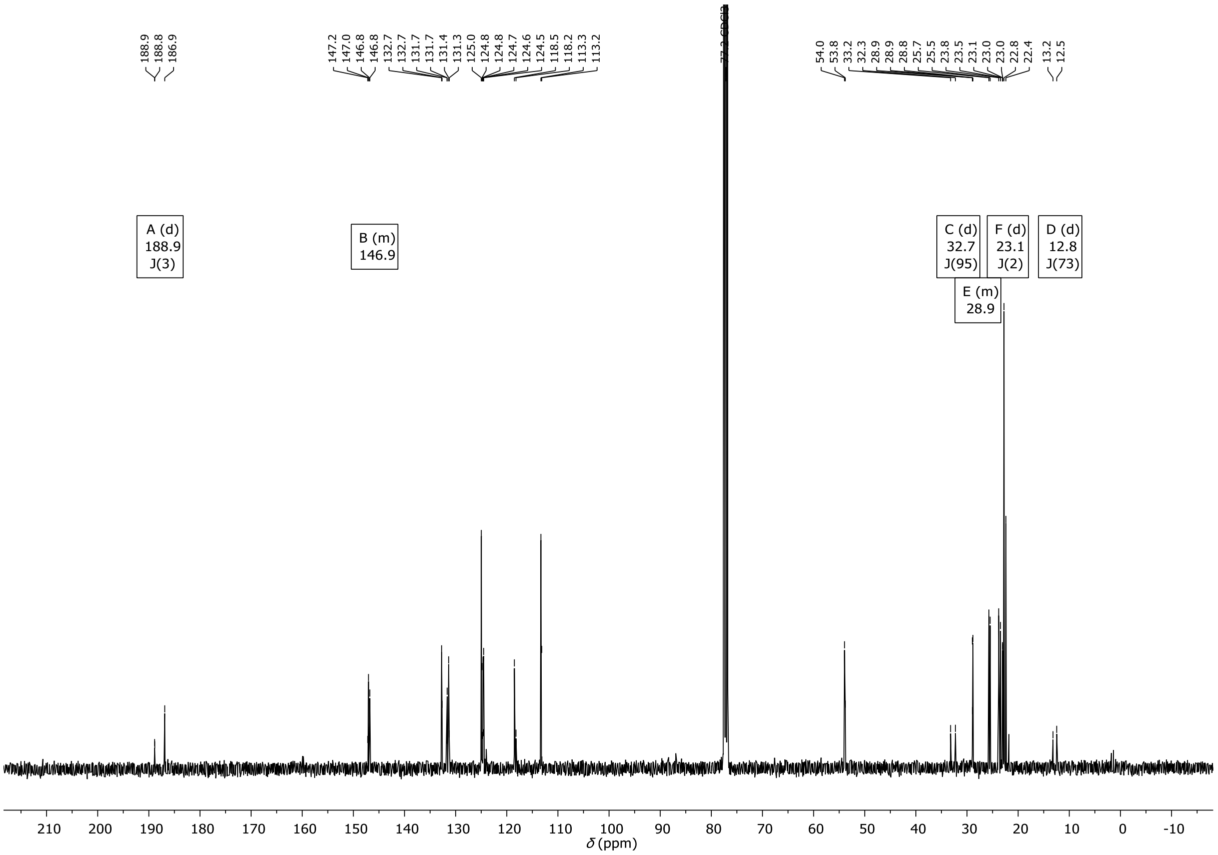

Figure S137: $^{13}C\{^{1}H\}$ NMR spectrum (101 MHz, $CDCl_3$) of the reaction with **10** and [ClAu(*i*Pr_2-bimy)].

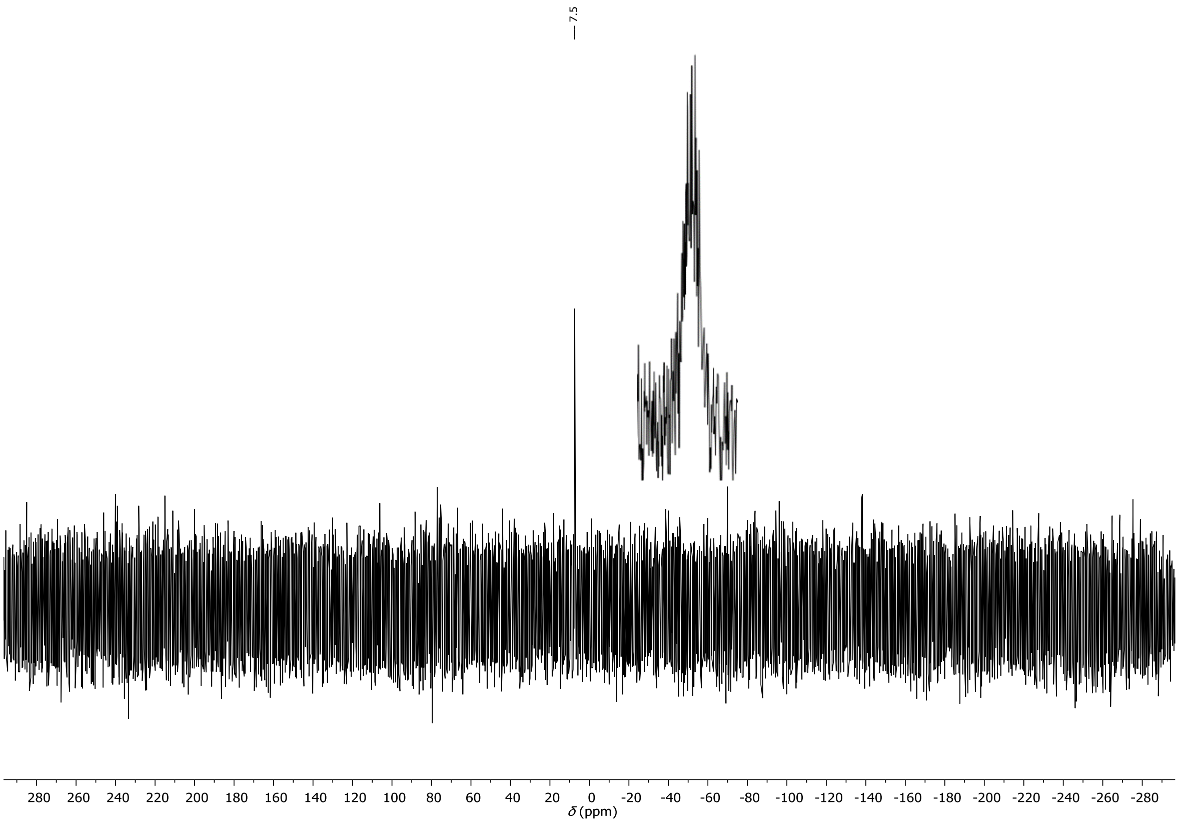

Figure S138: ^{31}P NMR spectrum (162 MHz, $CDCl_3$) of the reaction with **10** and [ClAu(*i*Pr_2-bimy)].

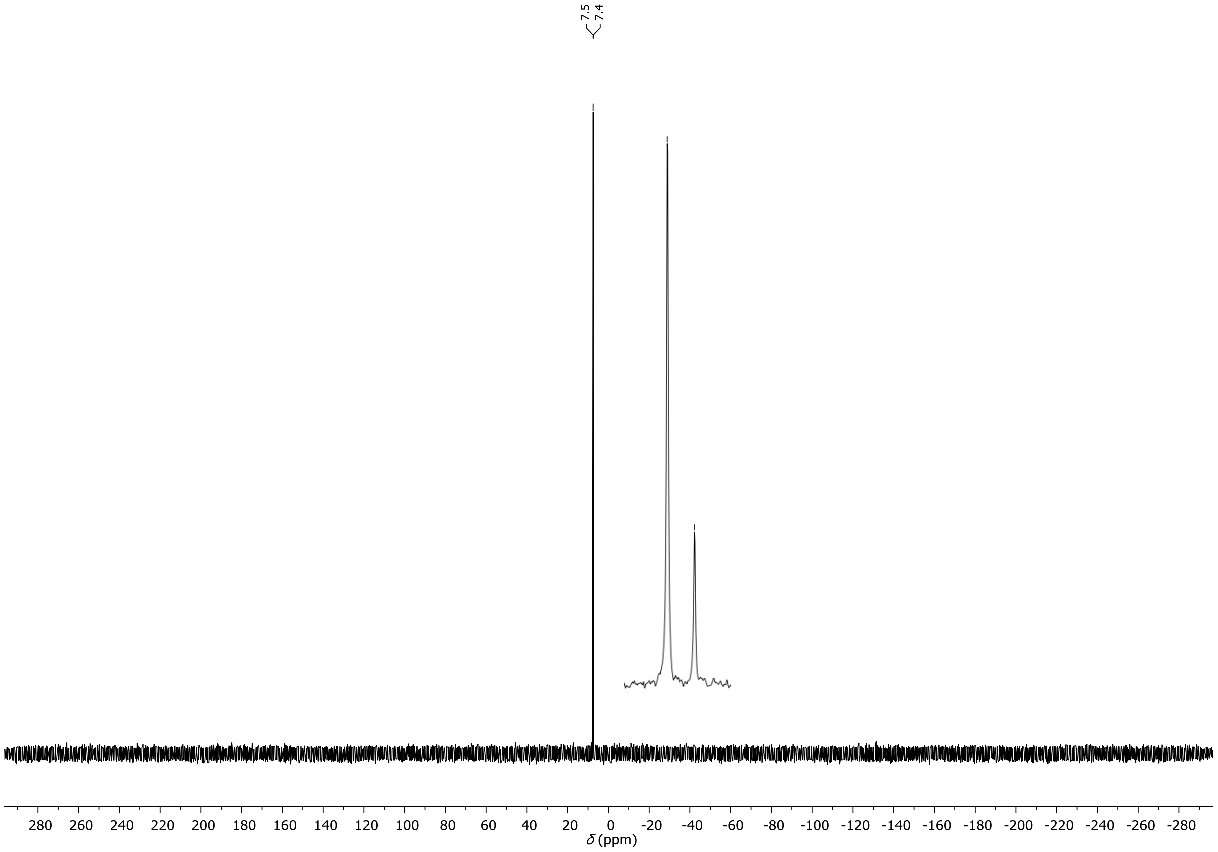

Figure S139: $^{31}P\{^1H\}$ NMR spectrum (162 MHz, $CDCl_3$) of the reaction with **10** and [ClAu(*i*Pr_2-bimy)].

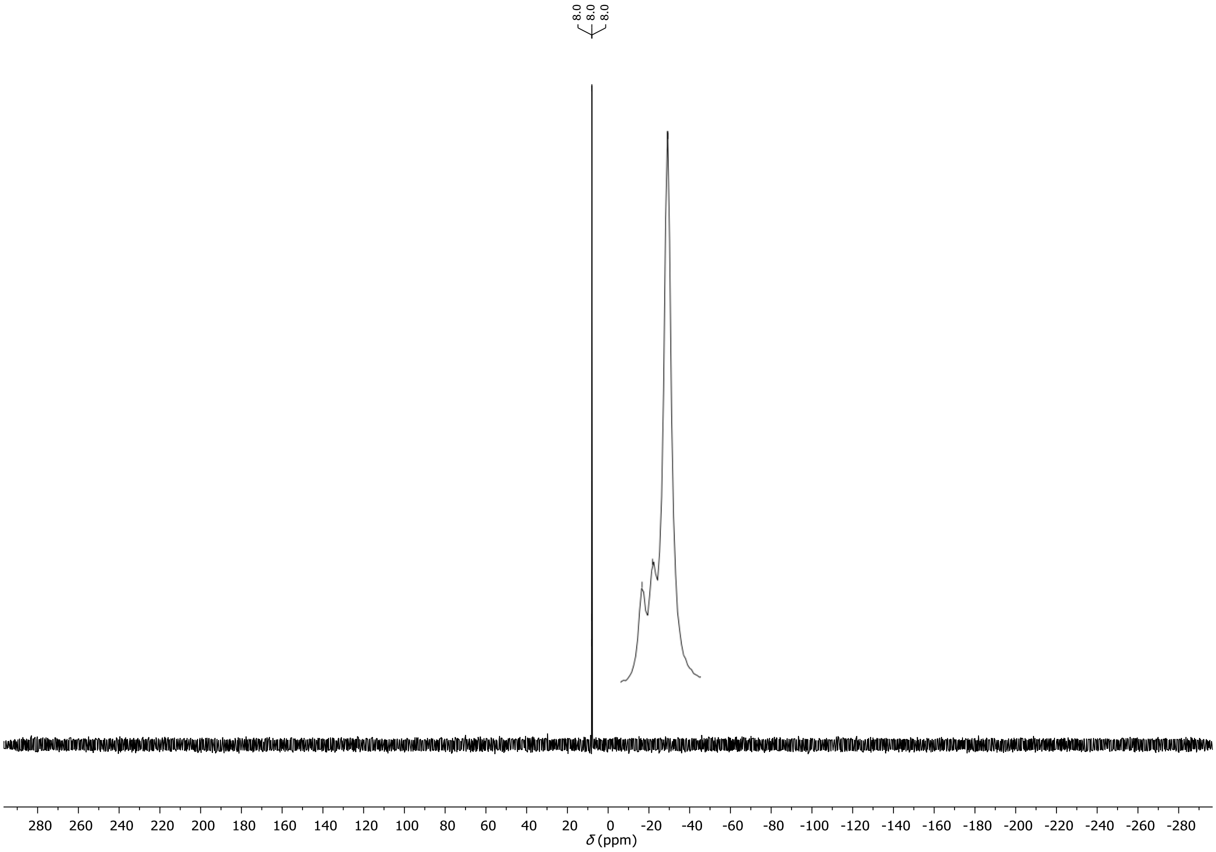

Figure S140: $^{31}P\{^1H\}$ NMR spectrum (162 MHz, CD_3CN) of the reaction with **10** and [ClAu(*i*Pr_2-bimy)].

X-ray diffraction studies

General

The crystals were measured at a steady temperature of 133 K (**6**, **10**), 150 K (**4**, **8**, **12**), 153 K (**5**, **16'**) or 173 K (**1**-**3**, **9**, **14**) on a Bruker D8 Quest diffractometer equipped with a Photon III detector (**1**-**6**, **8**-**10**, **12**, **14**, **16'**). A multi-scan absorption correction was performed for all compounds. The structures were solved with ShelXS/ShelXTL[6] (**1**,**2**) using direct methods or with ShelXT[7] (**3**-**6**, **8**-**10**, **12**, **14**, **16'**) using dual methods with Olex2[8] as a graphical interface. The structure models were refined with ShelXL[9] using full matrix least square minimization on F^2. All non-hydrogen atoms were refined anisotropically. Hydrogen atom positions were calculated geometrically and refined using the riding model. The figures depicting the molecular structures were generated using Diamond Version 4.6.8.

Single-crystal X-ray structure analysis of 1

Single crystals were obtained from a saturated solution of **1** in hexane at −40 °C. **1** crystallizes in the monoclinic space group $P2_1/c$. The asymmetric unit contains one molecule of **1**.

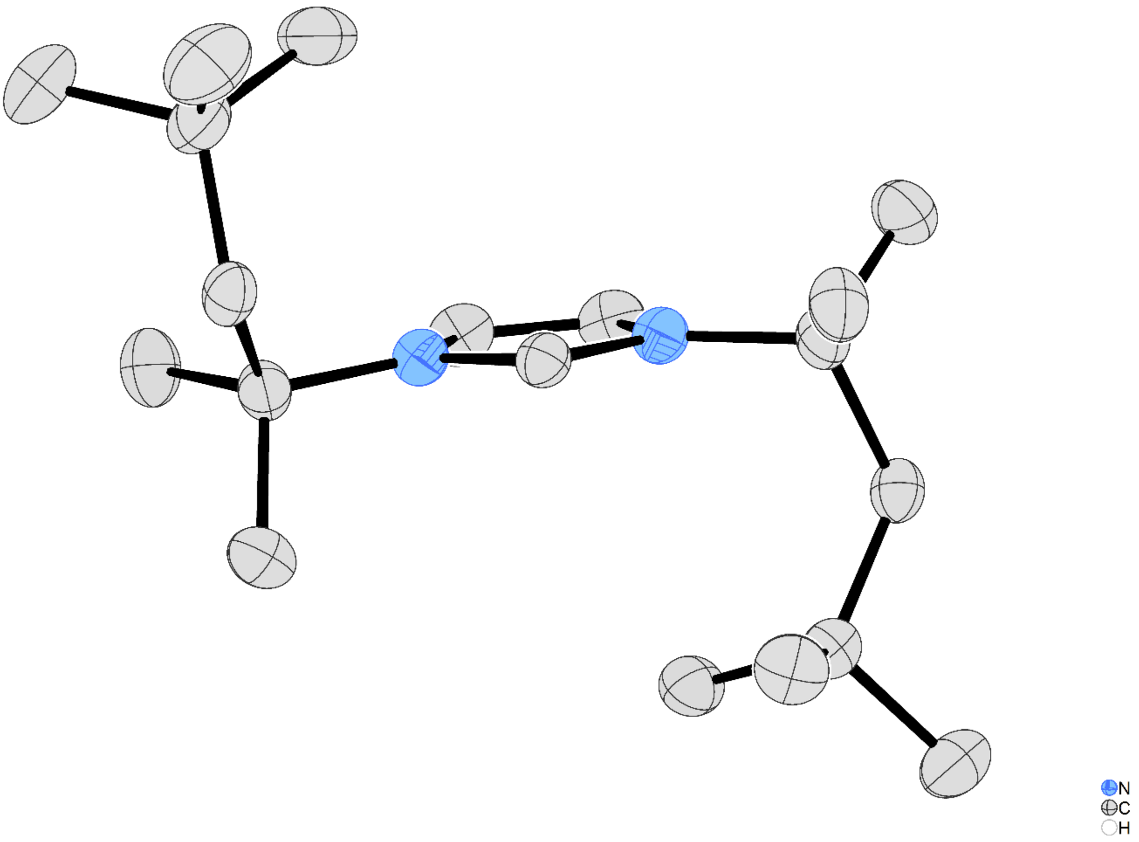

Figure S141: Asymmetric unit of **1**. Hydrogen atoms are omitted for clarity. Ellipsoids are displayed at 50% probability.

CCDC number	2531290	ρ_{calc}g/cm^3	1.019
Empirical formula	$C_{19}H_{36}N_2$	μ/mm^{-1}	0.059
Formula weight	292.50	F(000)	656.0

Temperature/K	173.00	Crystal size/mm^3	0.18 × 0.13 × 0.08
Crystal system	monoclinic	Radiation	MoKα (λ = 0.71073)
Space group	$P2_1/c$	2Θ range for data collection/°	4.55 to 51.988
a/Å	12.0766(4)	Index ranges	-14 ≤ h ≤ 14, -16 ≤ k ≤ 16, -14 ≤ l ≤ 13
b/Å	13.3479(5)	Reflections collected	47893
c/Å	11.8337(3)	Independent reflections	3743 [R_{int} = 0.0421, R_{sigma} = 0.0183]
α/°	90	Data/restraints/parameters	3743/0/205
β/°	91.5427(11)	Goodness-of-fit on F^2	1.076
γ/°	90	Final R indexes [I>=2σ (I)]	R_1 = 0.0377, wR_2 = 0.0994
Volume/Å^3	1906.87(11)	Final R indexes [all data]	R_1 = 0.0440, wR_2 = 0.1038
Z	4	Largest diff. peak/hole / e Å^{-3}	0.25/-0.16

Single-crystal X-ray structure analysis of **2**

Single crystals were obtained from a saturated solution of **2** in pentane at −40 °C. **2** crystallizes in the monoclinic space group $P2_1/n$. The asymmetric unit contains one molecule of **2**.

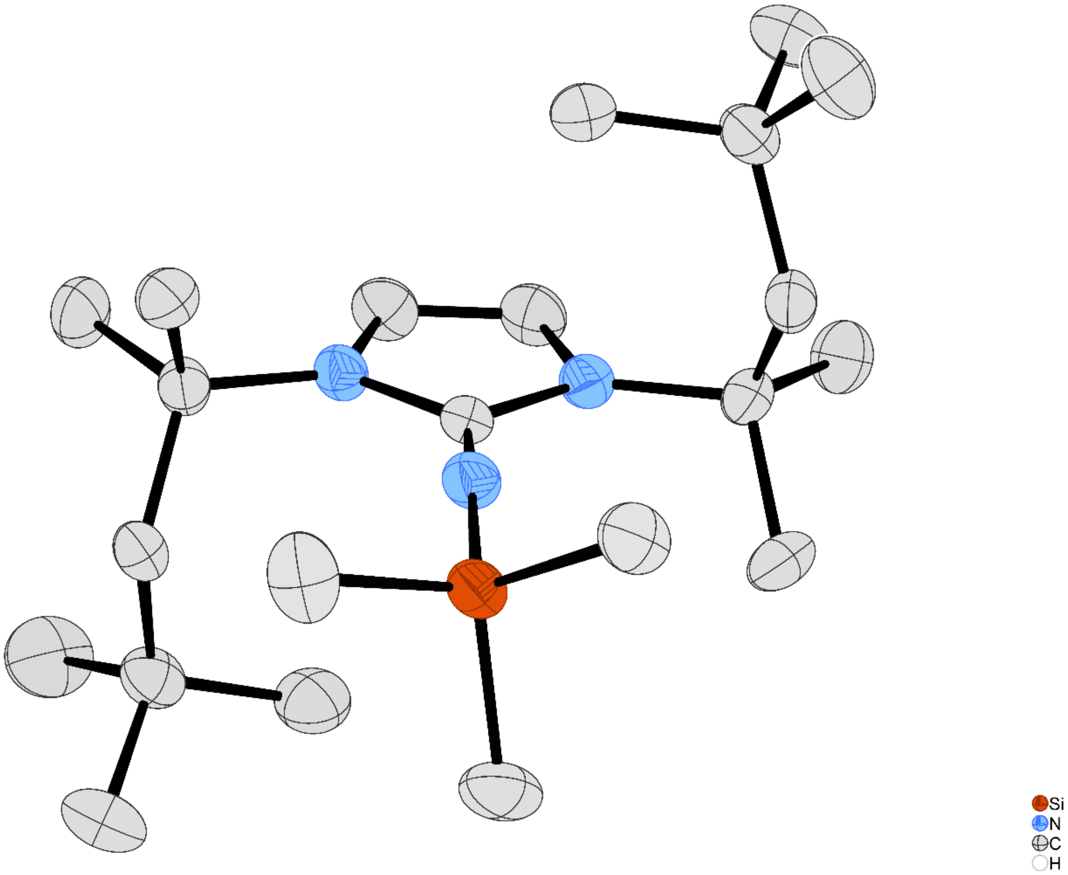

Figure S142: Asymmetric unit of **2**. Hydrogen atoms are omitted for clarity. Ellipsoids are displayed at 50% probability.

CCDC number	2531291	ρ_{calc}g/cm^3	1.034
Empirical formula	$C_{22}H_{45}N_3Si$	μ/mm^{-1}	0.107
Formula weight	379.70	F(000)	848.0
Temperature/K	173.00	Crystal size/mm^3	0.21 × 0.18 × 0.08
Crystal system	monoclinic	Radiation	MoKα (λ = 0.71073)
Space group	P2$_1$/n	2Θ range for data collection/°	3.494 to 56.826
a/Å	8.9025(2)	Index ranges	-11 ≤ h ≤ 11, -20 ≤ k ≤ 20, -23 ≤ l ≤ 23
b/Å	15.5175(5)	Reflections collected	54482
c/Å	17.6520(6)	Independent reflections	6092 [R_{int} = 0.0466, R_{sigma} = 0.0224]
α/°	90	Data/restraints/parameters	6092/0/248
β/°	91.0480(10)	Goodness-of-fit on F^2	1.031
γ/°	90	Final R indexes [I>=2σ (I)]	R_1 = 0.0367, wR_2 = 0.0938
Volume/Å^3	2438.12(13)	Final R indexes [all data]	R_1 = 0.0460, wR_2 = 0.1016
Z	4	Largest diff. peak/hole / e Å^{-3}	0.23/-0.23

Single-crystal X-ray structure analysis of **3**

Single crystals were obtained by solvent evaporation at room temperature from a solution of **3** in deuterated acetonitrile. **3** crystallizes in the monoclinic space group C2/c. The asymmetric unit contains one molecule of **3**. In addition, the asymmetric unit contains one heavily disordered acetonitrile molecule which was treated using a solvent mask. The N-P-N unit is disordered over two positions (occupancy 0.77 : 0.23).

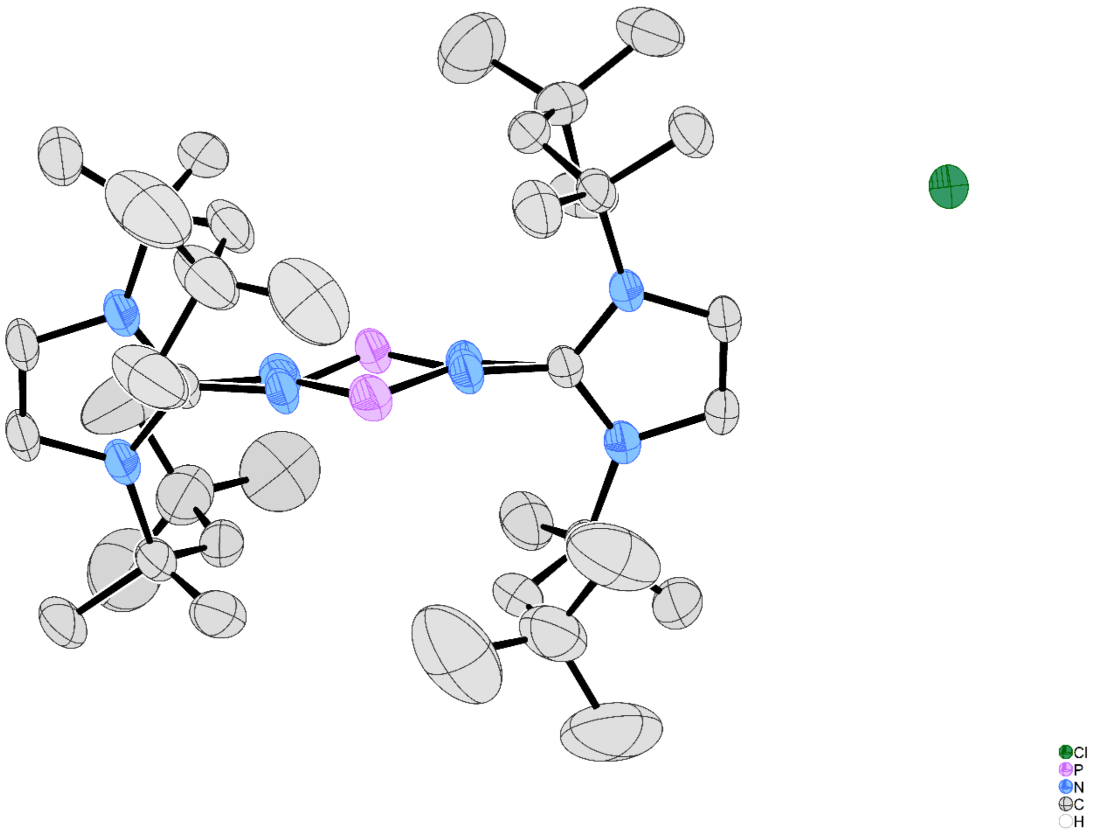

Figure S143: Asymmetric unit of **3**. Hydrogen atoms are omitted for clarity. Ellipsoids are displayed at 50% probability.

Identification code	2531292	ρ_{calc}g/cm^3	1.010
Empirical formula	$C_{40}H_{75}ClN_7P$	μ/mm^{-1}	0.146
Formula weight	720.49	F(000)	3168.0
Temperature/K	173.00	Crystal size/mm^3	0.28 × 0.18 × 0.11
Crystal system	monoclinic	Radiation	MoKα (λ = 0.71073)
Space group	C2/c	2Θ range for data collection/°	4.498 to 51.5
a/Å	21.0979(8)	Index ranges	-25 ≤ h ≤ 25, -27 ≤ k ≤ 27, -25 ≤ l ≤ 24
b/Å	22.1869(8)	Reflections collected	65315
c/Å	20.6699(6)	Independent reflections	9031 [R_{int} = 0.0439, R_{sigma} = 0.0314]
α/°	90	Data/restraints/parameters	9031/1/462
β/°	101.7190(10)	Goodness-of-fit on F^2	1.051
γ/°	90	Final R indexes [I>=2σ (I)]	R_1 = 0.0552, wR_2 = 0.1597
Volume/Å^3	9473.8(6)	Final R indexes [all data]	R_1 = 0.0642, wR_2 = 0.1697
Z	8	Largest diff. peak/hole / e Å^{-3}	0.41/-0.29

Single-crystal X-ray structure analysis of **4**

Single crystals were obtained by solvent evaporation at −40 °C from a pentane solution of **4** with toluene. **4** crystallizes in the monoclinic space group $P2_1/n$. The asymmetric unit contains one molecule of **4**. The N_2PC-unit is partially disordered over two positions (occupancy 0.50 : 0.50).

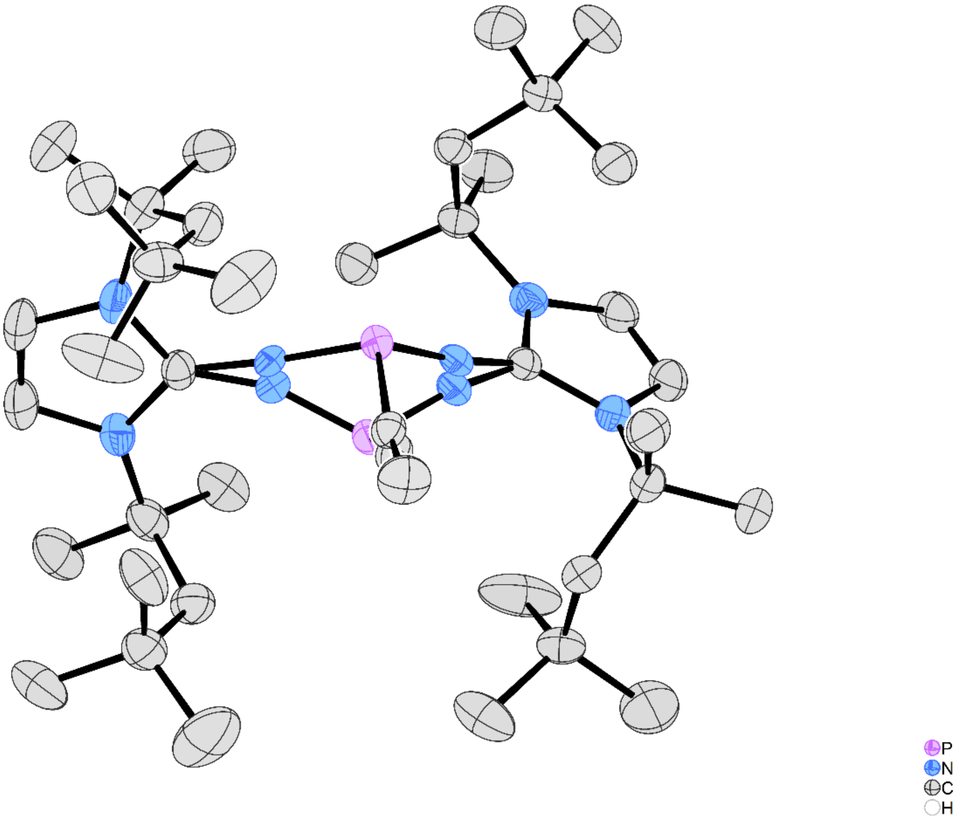

Figure S144: Asymmetric unit of **4**. Hydrogen atoms are omitted for clarity. Ellipsoids are displayed at 50% probability.

CCDC number	2531293	ρ_{calc}g/cm^3	1.071
Empirical formula	$C_{40}H_{73}N_6P$	μ/mm^{-1}	0.100
Formula weight	669.01	F(000)	1480.0
Temperature/K	150.00	Crystal size/mm^3	0.58 × 0.36 × 0.12
Crystal system	monoclinic	Radiation	MoKα (λ = 0.71073)
Space group	$P2_1/n$	2Θ range for data collection/°	4.526 to 54.982
a/Å	12.2517(4)	Index ranges	-15 ≤ h ≤ 15, -26 ≤ k ≤ 26, -21 ≤ l ≤ 21
b/Å	20.4608(8)	Reflections collected	51110
c/Å	16.8211(7)	Independent reflections	9499 [R_{int} = 0.0706, R_{sigma} = 0.0515]
α/°	90	Data/restraints/parameters	9499/2/485

β/°	100.3880(10)	Goodness-of-fit on F^2	1.028
γ/°	90	Final R indexes [I>=2σ (I)]	R_1 = 0.0537, wR_2 = 0.1339
Volume/Å^3	4147.6(3)	Final R indexes [all data]	R_1 = 0.0782, wR_2 = 0.1503
Z	4	Largest diff. peak/hole / e Å^{-3}	0.38/-0.29

Single-crystal X-ray structure analysis of **5**

Single crystals were obtained by solvent evaporation at −40 °C from a saturated solution of **5** in THF with hexane. **5** crystallizes in the monoclinic space group $P2_1$. The asymmetric unit contains one molecule of **5** and two molecules of THF. A *tert*-octyl group is partially disordered over two positions (occupancy 0.63 : 0.37).

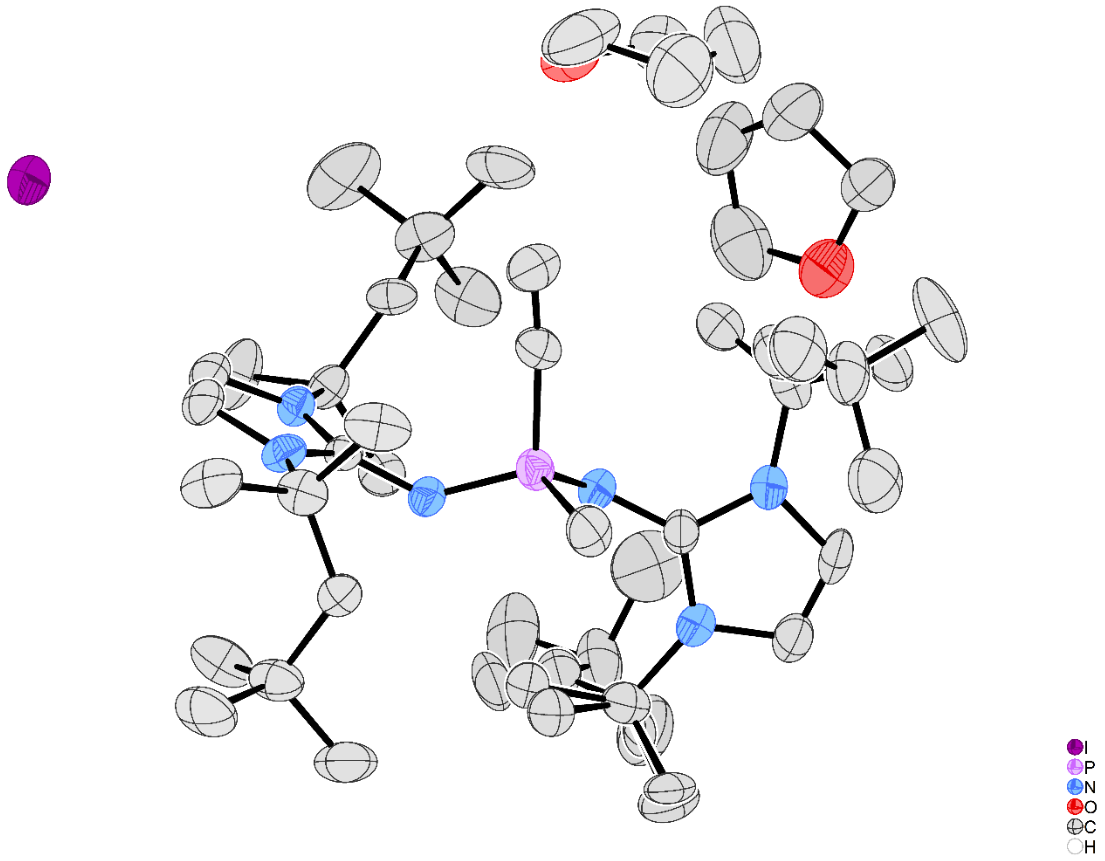

Figure S145: Asymmetric unit of **5**. Hydrogen atoms are omitted for clarity. Ellipsoids are displayed at 50% probability.

CCDC number	2531294	ρ_{calc}g/cm^3	1.173
Empirical formula	$C_{49}H_{92}IN_6O_2P$	μ/mm^{-1}	0.661
Formula weight	955.15	F(000)	1024.0
Temperature/K	153.00	Crystal size/mm^3	0.129 × 0.063 × 0.058
Crystal system	monoclinic	Radiation	MoKα (λ = 0.71073)
Space group	$P2_1$	2Θ range for data collection/°	4.564 to 52.79

a/Å	12.2007(18)	Index ranges	-15 ≤ h ≤ 15, -17 ≤ k ≤ 17, -19 ≤ l ≤ 20
b/Å	13.8818(15)	Reflections collected	29594
c/Å	16.1946(15)	Independent reflections	11027 [R_{int} = 0.0639, R_{sigma} = 0.0703]
α/°	90	Data/restraints/parameters	11027/187/593
β/°	99.567(4)	Goodness-of-fit on F^2	1.060
γ/°	90	Final R indexes [I>=2σ (I)]	R_1 = 0.0502, wR_2 = 0.1172
Volume/Å^3	2704.7(6)	Final R indexes [all data]	R_1 = 0.0645, wR_2 = 0.1252
Z	2	Largest diff. peak/hole / e Å^{-3}	0.71/-0.31

Single-crystal X-ray structure analysis of **6**

Single crystals were obtained by solvent evaporation at room temperature from a solution of **6** in toluene with hexane. **6** crystallizes in the monoclinic space group $P2_1/n$. The asymmetric unit contains one molecule of **6**, 0.4 molecules of toluene and 0.8 hexane. The hexane was located on an inversion center and is heavily disordered, therefore a solvent mask was used. A solvent mask was calculated, and 172 electrons were found in a volume of 526 Å^3 in 2 voids per unit cell. This is consistent with the presence of 0.8 hexane molecules per asymmetric unit which accounts for 160 electrons per unit cell. A *tert*-octyl group is partially disordered over two positions (occupancy 0.81 : 0.19). Toluene is found in two positions.

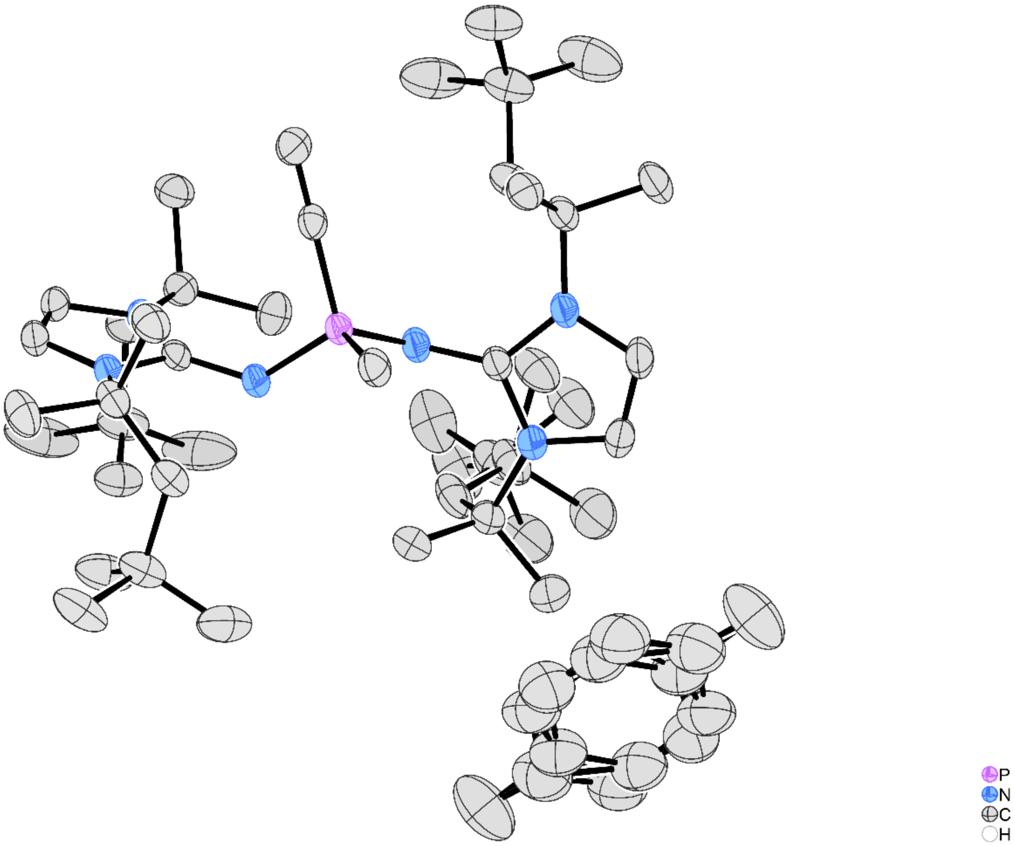

Figure S146: Asymmetric unit of **6**. Hydrogen atoms are omitted for clarity. Ellipsoids are displayed at 50% probability.

CCDC number	2531295	ρ_{calc}g/cm^3	1.095
Empirical formula	$C_{48.6}H_{89.4}N_6P$	μ/mm^{-1}	0.096
Formula weight	788.83	F(000)	1752.0
Temperature/K	133.00	Crystal size/mm^3	0.17 × 0.12 × 0.05
Crystal system	monoclinic	Radiation	MoKα (λ = 0.71073)
Space group	P2$_1$/n	2Θ range for data collection/°	4.472 to 53.506
a/Å	17.7677(8)	Index ranges	-22 ≤ h ≤ 22, -17 ≤ k ≤ 17, -24 ≤ l ≤ 25
b/Å	13.8078(7)	Reflections collected	137036
c/Å	19.7441(12)	Independent reflections	10162 [R_{int} = 0.0672, R_{sigma} = 0.0277]
α/°	90	Data/restraints/parameters	10162/148/558
β/°	99.106(2)	Goodness-of-fit on F^2	1.039
γ/°	90	Final R indexes [I>=2σ (I)]	R_1 = 0.0523, wR_2 = 0.1433
Volume/Å^3	4782.8(4)	Final R indexes [all data]	R_1 = 0.0670, wR_2 = 0.1555
Z	4	Largest diff. peak/hole / e Å^{-3}	0.72/-0.26

Single-crystal X-ray structure analysis of **8**

Single crystals of **8** suitable for X-ray diffraction analysis were obtained from a benzene solution of **8** at room temperature. The single-crystal X-ray structure analysis revealed that **8** crystallizes in the orthorhombic space group Pbca. The asymmetric unit contains two molecules of **8**. One of the molecules is disordered at the (HCC)P*t*Bu moiety over two positions (occupancy 0.80 : 0.20).

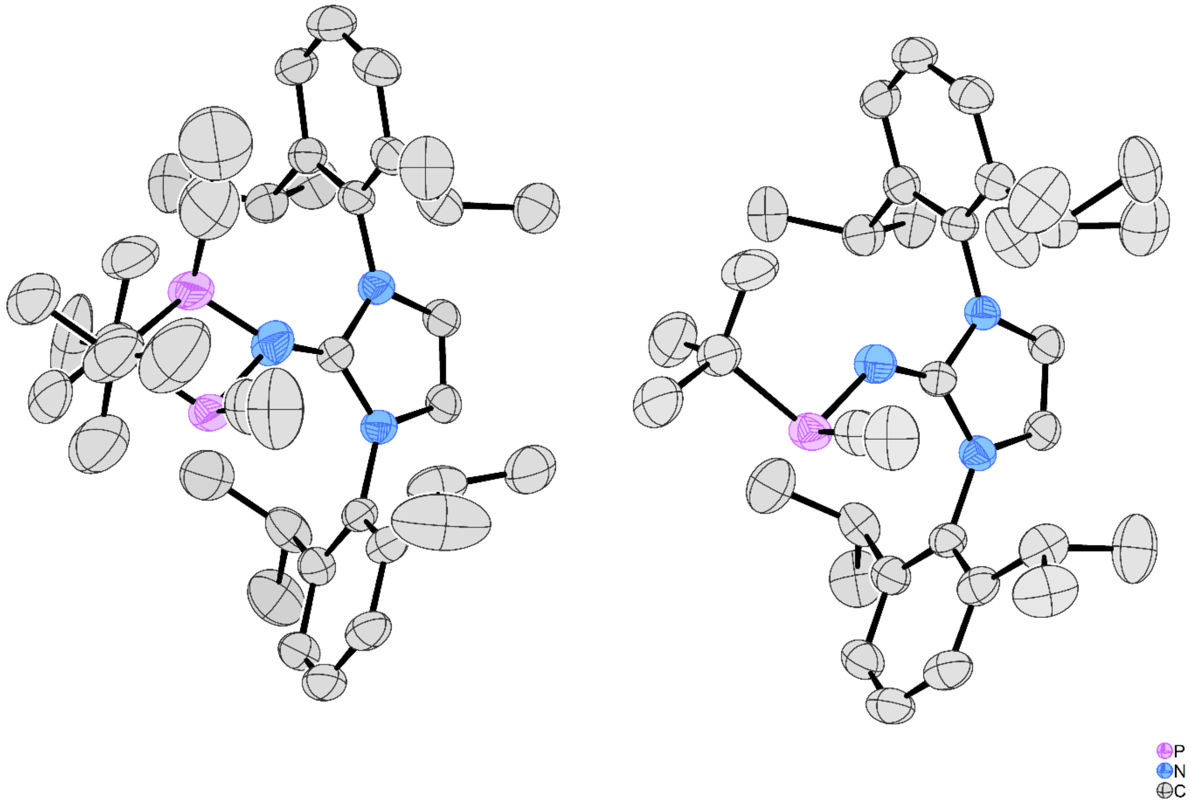

Figure S147: Asymmetric unit of **8**. Hydrogen atoms are omitted for clarity. Ellipsoids are displayed at 50% probability.

CCDC number	2531296	ρ_{calc}g/cm^3	1.078
Empirical formula	$C_{33}H_{46}N_3P$	μ/mm^{-1}	0.110
Formula weight	515.70	F(000)	4480.0
Temperature/K	150.00	Crystal size/mm^3	0.538 × 0.12 × 0.088
Crystal system	orthorhombic	Radiation	MoKα (λ = 0.71073)
Space group	Pbca	2Θ range for data collection/°	3.834 to 50.784
a/Å	19.0683(7)	Index ranges	-23 ≤ h ≤ 22, -17 ≤ k ≤ 19, -32 ≤ l ≤ 48
b/Å	16.5574(6)	Reflections collected	84534
c/Å	40.2705(16)	Independent reflections	11651 [R_{int} = 0.0596, R_{sigma} = 0.0391]

α/°	90	Data/restraints/parameters	11651/154/777
β/°	90	Goodness-of-fit on F^2	1.026
γ/°	90	Final R indexes [I>=2σ (I)]	R_1 = 0.0621, wR_2 = 0.1628
Volume/Å^3	12714.3(8)	Final R indexes [all data]	R_1 = 0.0969, wR_2 = 0.1875
Z	16	Largest diff. peak/hole / e Å^{-3}	0.69/-0.47

Single-crystal X-ray structure analysis of **9**

Single crystals of **9** suitable for X-ray diffraction analysis were obtained from vapor diffusion at −40 °C of a ACN solution of **9** with hexane. The single-crystal X-ray structure analysis revealed that **9** crystallizes in the monoclinic space group C2/c. The asymmetric unit contains one molecule of **9** with the iodine counterion found in two positions (occupancy 0.50 : 0.50) and two molecules of ACN.

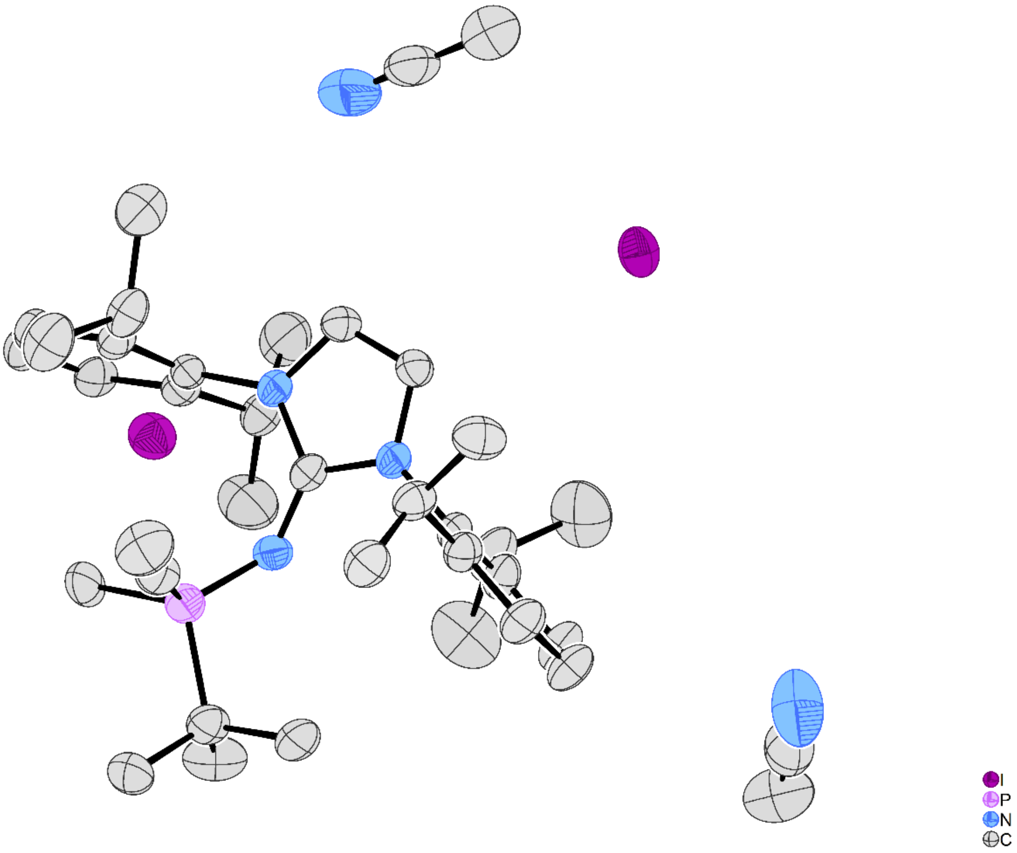

Figure S148: Asymmetric unit of **9**. Hydrogen atoms are omitted for clarity. Ellipsoids are displayed at 50% probability.

CCDC number	2531297	ρ_{calc}g/cm^3	1.220
Empirical formula	$C_{38}H_{55}IN_5P$	μ/mm^{-1}	0.865
Formula weight	739.74	F(000)	3088.0
Temperature/K	173.00	Crystal size/mm^3	0.13 × 0.05 × 0.03

Crystal system	monoclinic	Radiation	MoKα (λ = 0.71073)
Space group	C2/c	2Θ range for data collection/°	4.528 to 49.996
a/Å	22.2117(13)	Index ranges	$-26 \leq h \leq 26$, $-23 \leq k \leq 23$, $-20 \leq l \leq 22$
b/Å	20.1551(12)	Reflections collected	78552
c/Å	18.7037(10)	Independent reflections	7095 [R_{int} = 0.0543, R_{sigma} = 0.0369]
α/°	90	Data/restraints/parameters	7095/0/421
β/°	105.858(2)	Goodness-of-fit on F^2	1.061
γ/°	90	Final R indexes [I>=2σ (I)]	R_1 = 0.0412, wR_2 = 0.1070
Volume/Å^3	8054.6(8)	Final R indexes [all data]	R_1 = 0.0612, wR_2 = 0.1194
Z	8	Largest diff. peak/hole / e Å^{-3}	0.82/-0.93

Single-crystal X-ray structure analysis of **10**

Single crystals of **10** suitable for X-ray diffraction analysis were obtained from vapor diffusion at room temperature from a toluene solution of **10** with *n*-hexane. The single-crystal X-ray structure analysis revealed that **10** crystallizes in the monoclinic space group $P2_1/c$. The asymmetric unit contains two molecules of **10**. The P(CC)(Me)(*t*Bu) moiety is disordered over two positions (occupancy 0.68 : 0.32 / 0.61 : 0.39). The dipp groups are partially disordered over two positions (occupancy 0.64 : 0.36 / 0.63 : 0.37 / 0.85 : 0.15 / 0.57 : 0.43).

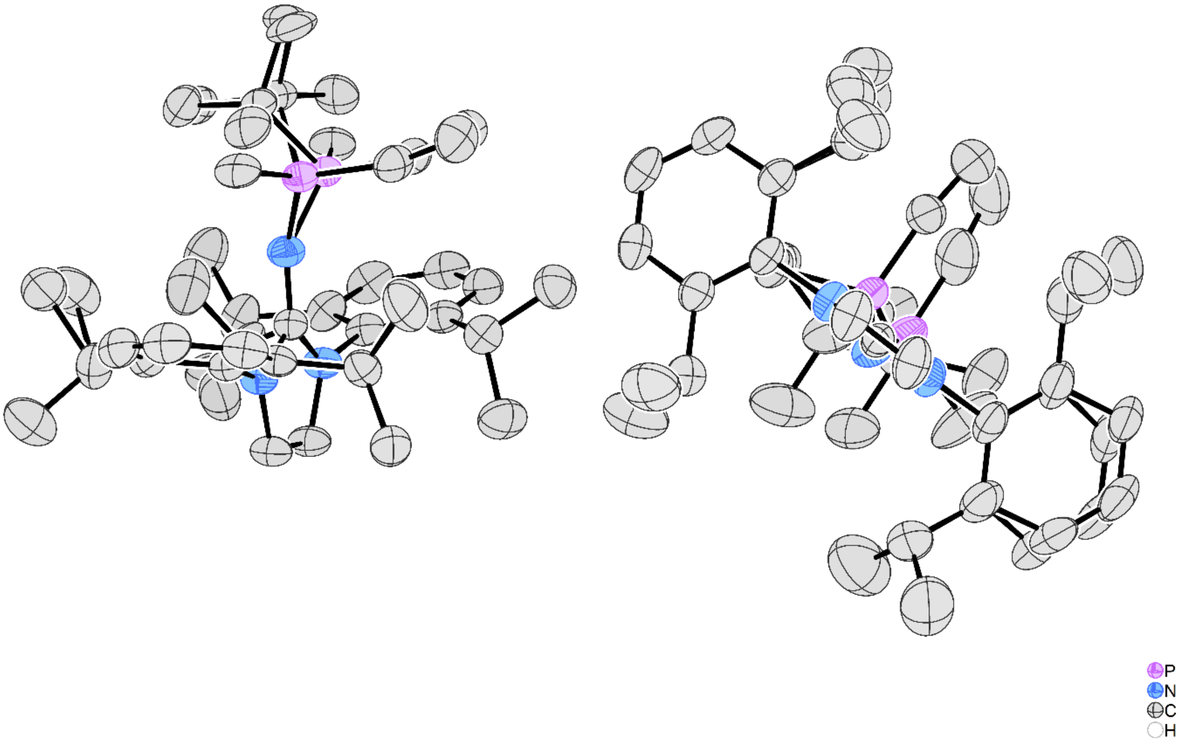

Figure S149: Asymmetric unit of **10**. Hydrogen atoms are omitted for clarity. Ellipsoids are displayed at 50% probability.

CCDC number	2531298	ρ_{calc}g/cm^3	1.095
Empirical formula	$C_{34}H_{48}N_3P$	μ/mm^{-1}	0.111
Formula weight	529.72	F(000)	2304.0
Temperature/K	133.00	Crystal size/mm^3	0.28 × 0.26 × 0.12
Crystal system	monoclinic	Radiation	MoKα (λ = 0.71073)
Space group	P2$_1$/c	2Θ range for data collection/°	4.172 to 58.27
a/Å	20.676(3)	Index ranges	-27 ≤ h ≤ 28, -22 ≤ k ≤ 22, -25 ≤ l ≤ 27
b/Å	16.230(2)	Reflections collected	134352
c/Å	19.909(2)	Independent reflections	17236 [R_{int} = 0.0619, R_{sigma} = 0.0381]
α/°	90	Data/restraints/parameters	17236/315/962
β/°	105.913(4)	Goodness-of-fit on F^2	1.056
γ/°	90	Final R indexes [I>=2σ (I)]	R_1 = 0.0587, wR_2 = 0.1592
Volume/Å^3	6424.8(14)	Final R indexes [all data]	R_1 = 0.0935, wR_2 = 0.1847
Z	8	Largest diff. peak/hole / e Å^{-3}	0.27/-0.25

Single-crystal X-ray structure analysis of **12**

Single crystals of **12** suitable for X-ray diffraction analysis were obtained from vapor diffusion at room temperature of a benzene solution of **12** with *n*-hexane. The single-crystal X-ray structure analysis revealed that **12** crystallizes in the orthorhombic space group $P2_12_12_1$. The asymmetric unit contains one molecule of **12**. The OPMe*t*Bu moiety is disordered over two positions (occupancy 0.53 : 0.47) and the *iso*-propyl groups are each disordered over two positions (occupancy 0.56 : 0.44 / 0.7 : 0.3 / 0.52 : 0.48 / 0.76 : 0.24).

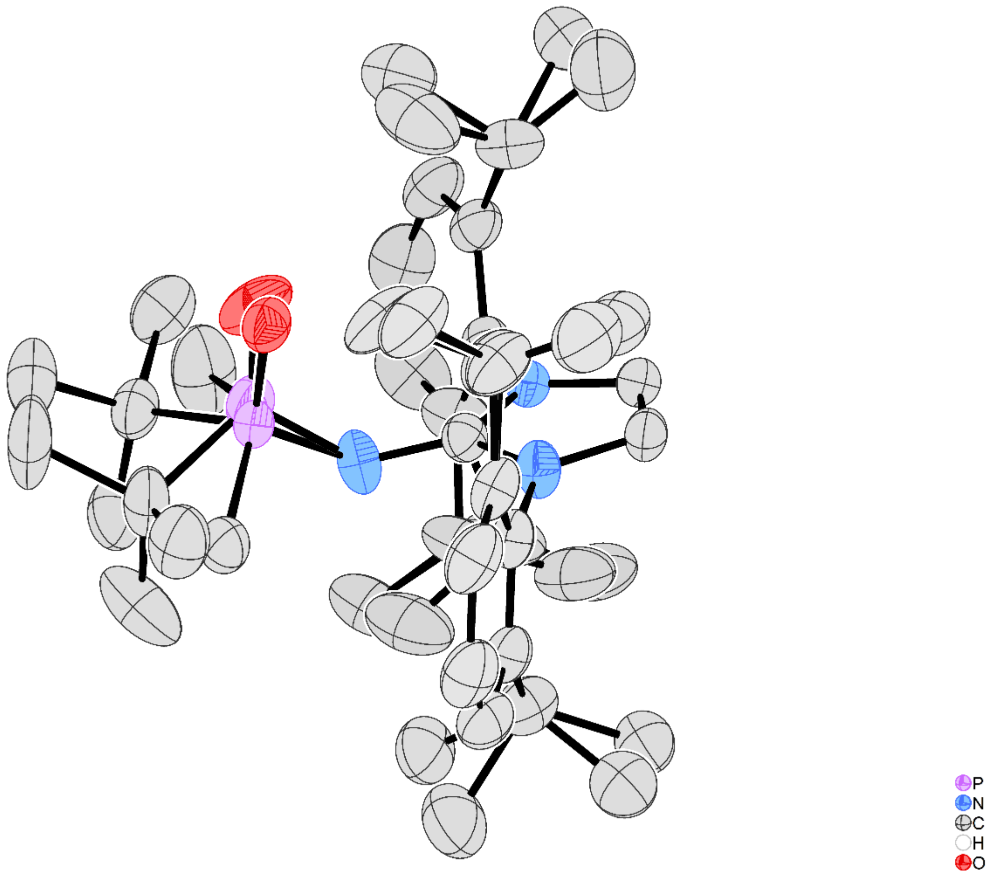

Figure S150: Asymmetric unit of **12**. Hydrogen atoms are omitted for clarity. Ellipsoids are displayed at 50% probability.

CCDC number	2531299	ρ_{calc}g/cm^3	1.077
Empirical formula	$C_{32}H_{48}N_3OP$	μ/mm^{-1}	0.112
Formula weight	521.70	F(000)	1136.0
Temperature/K	150.00	Crystal size/mm^3	0.36 × 0.33 × 0.28
Crystal system	orthorhombic	Radiation	MoKα (λ = 0.71073)
Space group	$P2_12_12_1$	2Θ range for data collection/°	4.664 to 52.832
a/Å	11.331(2)	Index ranges	-14 ≤ h ≤ 14, -17 ≤ k ≤ 17, -25 ≤ l ≤ 25
b/Å	13.707(3)	Reflections collected	62103

c/Å	20.720(4)	Independent reflections	6598 [R_{int} = 0.0843, R_{sigma} = 0.0482]
α/°	90	Data/restraints/parameters	6598/371/516
β/°	90	Goodness-of-fit on F^2	1.111
γ/°	90	Final R indexes [I>=2σ (I)]	R_1 = 0.0583, wR_2 = 0.1364
Volume/Å^3	3218.2(10)	Final R indexes [all data]	R_1 = 0.0644, wR_2 = 0.1392
Z	4	Largest diff. peak/hole / e Å^{-3}	0.14/-0.19

Single-crystal X-ray structure analysis of **14**

Single crystals of **14** suitable for X-ray diffraction analysis were obtained from a benzene solution of **14** at room temperature. The single-crystal X-ray structure analysis revealed that **14** crystallizes in the orthorhombic space group Fddd. The asymmetric unit contains one molecule of **14** and half a molecule of benzene. One half solvent molecule of C_6H_6 is strongly disordered. Therefore, a solvent mask was calculated. 1192 electrons were found in a volume of 5896 Å^3 in 4 voids per unit cell. This is consistent with the presence of half a benzene molecule per Asymmetric Unit which account for 672 electrons per unit cell. One half molecule is disordered at the Me-P-*t*Bu moiety and one half molecule is disordered at the CC-PMe*t*Bu over two positions (occupancy 0.66 : 0.33).

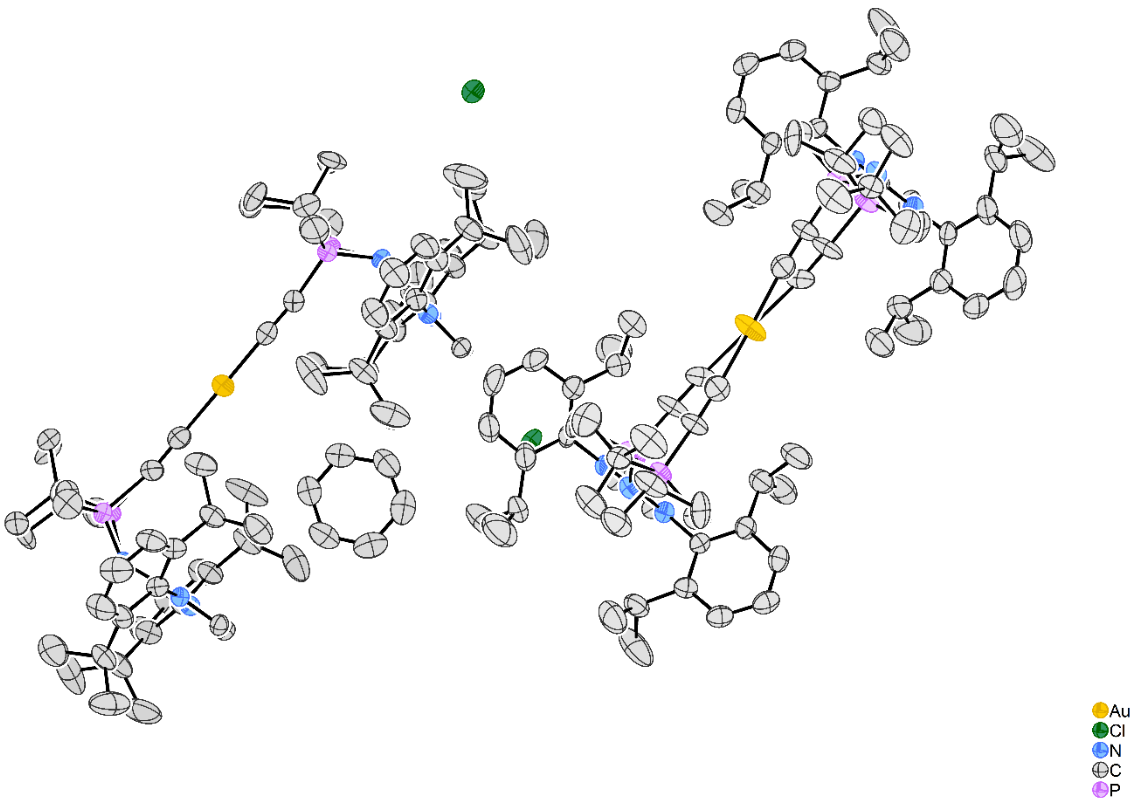

Figure S151: Molecular structure of **14**. Hydrogen atoms are omitted for clarity. Ellipsoids are displayed at 50% probability.

CCDC number	2531300	ρ_{calc}g/cm^3	1.236
Empirical formula	$AuC_{74}ClH_{102}N_6P_2$	μ/mm^{-1}	2.121
Formula weight	1369.97	F(000)	22848.0
Temperature/K	173.00	Crystal size/mm^3	0.15 × 0.07 × 0.04
Crystal system	orthorhombic	Radiation	MoKα (λ = 0.71073)
Space group	Fddd	2Θ range for data collection/°	3.904 to 50.794
a/Å	22.0484(15)	Index ranges	-26 ≤ h ≤ 26, -52 ≤ k ≤ 52, -60 ≤ l ≤ 73
b/Å	43.612(3)	Reflections collected	145471
c/Å	61.233(5)	Independent reflections	13522 [R_{int} = 0.1033, R_{sigma} = 0.0637]
α/°	90	Data/restraints/parameters	13522/0/869
β/°	90	Goodness-of-fit on F^2	1.025
γ/°	90	Final R indexes [I>=2σ (I)]	R_1 = 0.0587, wR_2 = 0.1329
Volume/Å^3	58880(7)	Final R indexes [all data]	R_1 = 0.0911, wR_2 = 0.1495
Z	32	Largest diff. peak/hole / e Å^{-3}	0.71/-1.44

Single-crystal X-ray structure analysis of **16'**

Single crystals of **16'** suitable for X-ray diffraction analysis were obtained at −40 °C from an THF solution of the reaction mixture with **10**, [ClAu(*i*Pr_2-bimy)] and $TlPF_6$ with diethyl ether. The single-crystal X-ray structure analysis revealed that **16'** crystallizes in the triclinic space group P-1. The asymmetric unit contains one molecule of **16'** and one molecule of THF. One solvent molecule of THF is strongly disordered. Therefore, a solvent mask was calculated. 74 electrons were found in a volume of 239 Å^3 in 2 voids per unit cell. This is consistent with the presence of one THF molecule per Asymmetric Unit which account for 80 electrons per unit cell. The Au-C-C-P(Me)(tBu)-N-unit is disordered over two positions (occupancy 0.64 : 0.36). One *iso*-propyl group of the dipp is disordered over two positions (occupancy 0.54 : 0.46).

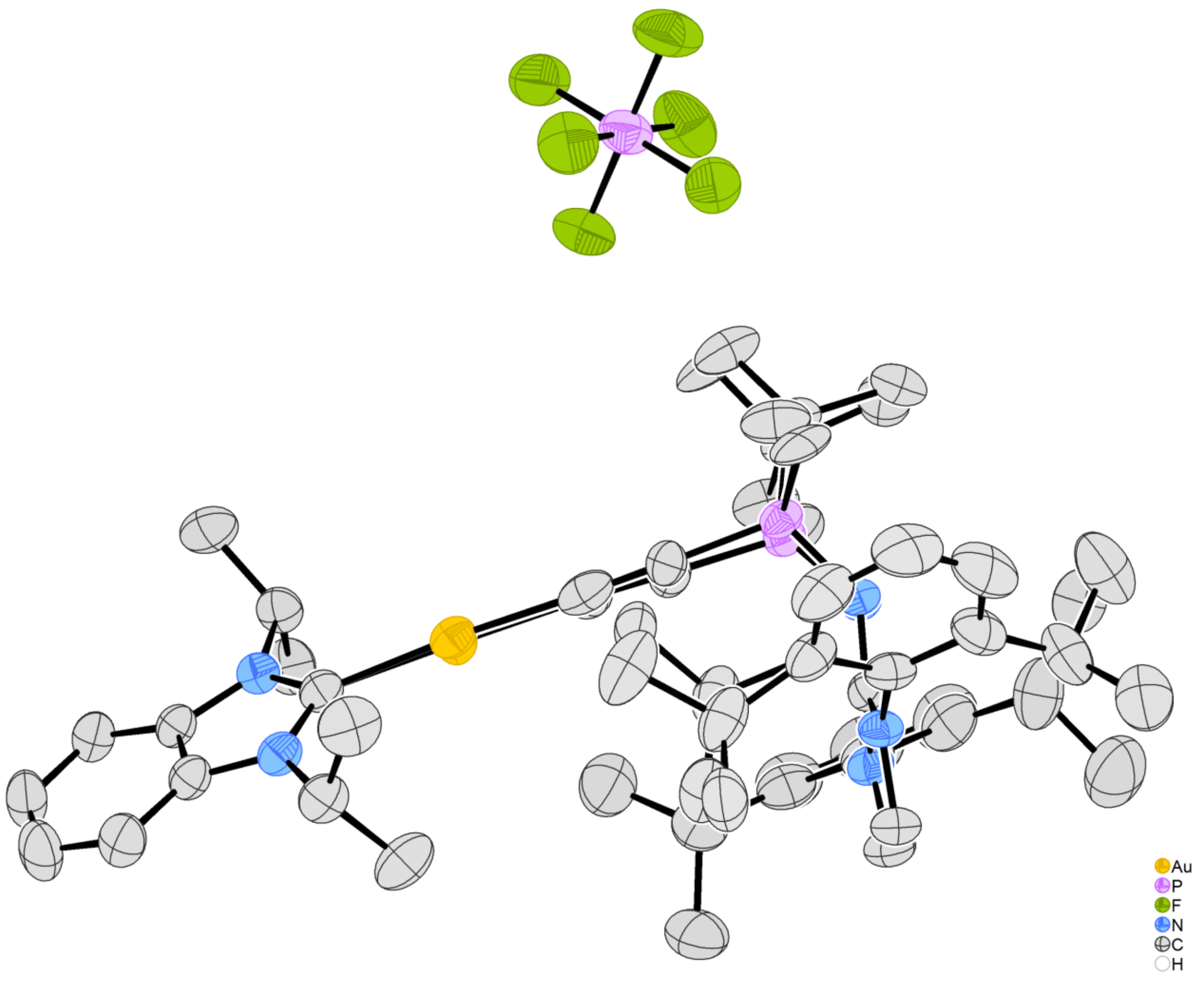

Figure S152: Asymmetric unit of **16’**. Hydrogen atoms are omitted for clarity. Ellipsoids are displayed at 50% probability.

CCDC number	2546887	ρ_{calc}g/cm^3	1.441
Empirical formula	$C_{51}H_{74}AuF_6N_5OP_2$	μ/mm^{-1}	2.907
Formula weight	1146.05	F(000)	1172.0
Temperature/K	153.00	Crystal size/mm^3	0.094 × 0.085 × 0.03
Crystal system	triclinic	Radiation	MoKα (λ = 0.71073)
Space group	P-1	2Θ range for data collection/°	4.426 to 52.744
a/Å	10.3847(4)	Index ranges	-12 ≤ h ≤ 12, -18 ≤ k ≤ 18, -23 ≤ l ≤ 23
b/Å	14.4526(6)	Reflections collected	81766
c/Å	19.0428(9)	Independent reflections	10777 [R_{int} = 0.0509, R_{sigma} = 0.0273]
α/°	85.336(2)	Data/restraints/parameters	10777/114/691
β/°	79.489(2)	Goodness-of-fit on F^2	1.035
γ/°	70.095(2)	Final R indexes [I>=2σ (I)]	R_1 = 0.0255, wR_2 = 0.0618

Volume/Å^3	2641.6(2)	Final R indexes [all data]	R_1 = 0.0322, wR_2 = 0.0646
Z	2	Largest diff. peak/hole / e Å^{-3}	0.54/-0.53

DFT calculations

Calculation of the relative energies of the frontier orbitals corresponding to the donor and acceptor properties

The calculation followed a method reported by Wagler in 2009.[10] The geometry optimizations and frequency calculations were carried out using the B3LYP[11,12] functional and the 6-311 G(d)[13,14] basis set implemented in Gaussian 16[15].[16–18] No imaginary frequencies were obtained, therefore confirming the calculated structure is a local minimum.

Analogous to our previous paper, the π-acceptor, σ-donor and π-donor orbitals were selected with the most similar topology to those of CO, CNMe and $CCPPh_3$. For the π-acceptor and π-donor orbitals, two orthogonal orbitals were found, respectively. In case of the π-donor orbital, the highest lying orbital with suitable orbital coefficients was chosen. For the π-acceptor orbitals, the lowest lying orbital with suitable orbital coefficients was chosen.[19]

Other than expected, the energies are not always on the same level of those orthogonal orbitals as for large systems orbital mixing is observed. Additionally, the LUMOs are also depicted if they don't correspond to the above-mentioned orbitals.

The topologies of the orbitals are displayed with Chemcraft[20].

Depicted relevant frontier orbitals

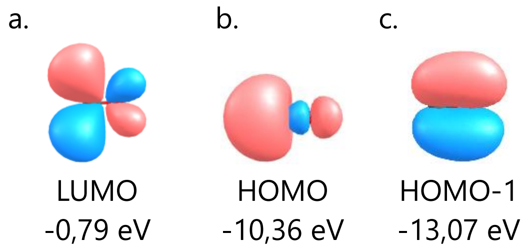

Figure S153: a.-c. Frontier orbitals of CO corresponding to a. the π acceptor, b. the σ donor and c. the π donor orbital.

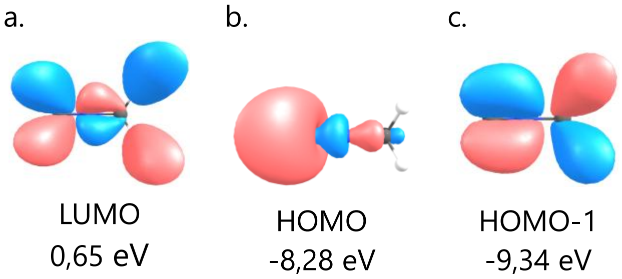

Figure S154: a.-c. Frontier orbitals of CNMe corresponding to a. the π acceptor, b. the σ donor and c. the π donor orbital.

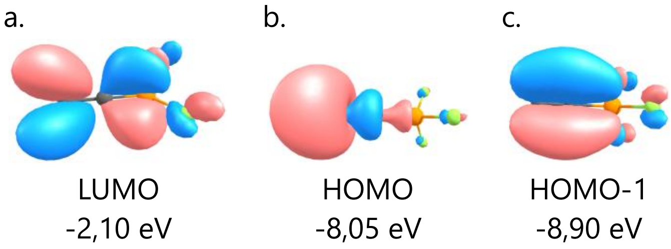

Figure S155: a.-c. Frontier orbitals of $CCPF_3$ corresponding to a. the π acceptor, b. the σ donor and c. the π donor orbital.

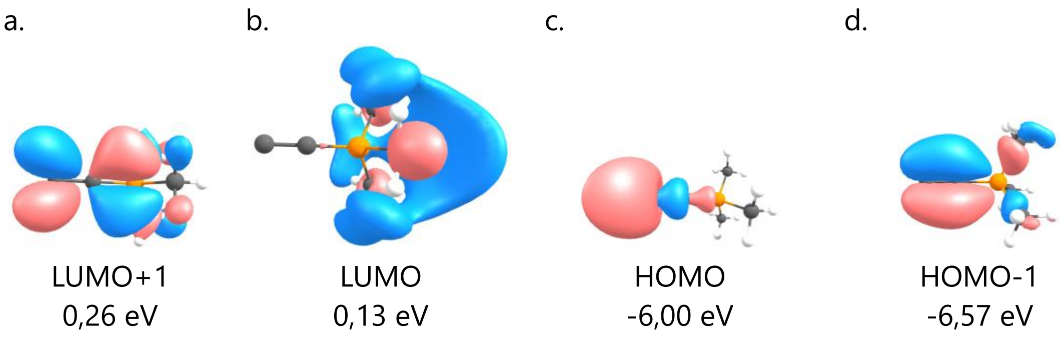

Figure S156: a.-d. Frontier orbitals of $CCPMe_3$ corresponding to a. the π acceptor, b. the LUMO, c. the σ donor and d. the π donor orbital.

a. b. c. d.

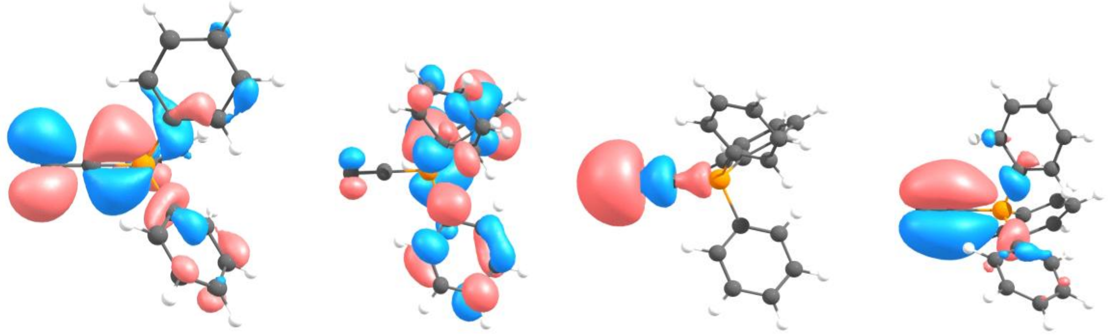

LUMO+6 0,66 eV | LUMO -1,45 eV | HOMO -5,95 eV | HOMO-1 -6,48 eV

Figure S157: a.-d. Frontier orbitals of $CCPPh_3$ (**A**) corresponding to a. the π acceptor, b. the LUMO, c. the σ donor and d. the π donor orbital.

a. b. c. d.

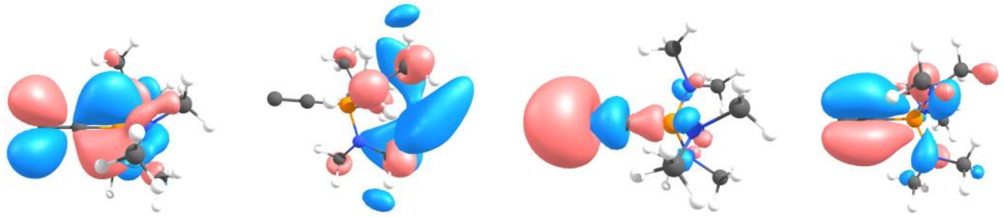

LUMO+1 0,56 eV | LUMO 0,24 eV | HOMO -5,79 eV | HOMO-1 -6,27 eV

Figure S158: a.-d. Frontier orbitals of $CCP(NMe_2)_3$ corresponding to a. the π acceptor, b. the LUMO, c. the σ donor and d. the π donor orbital.

a. b. c.

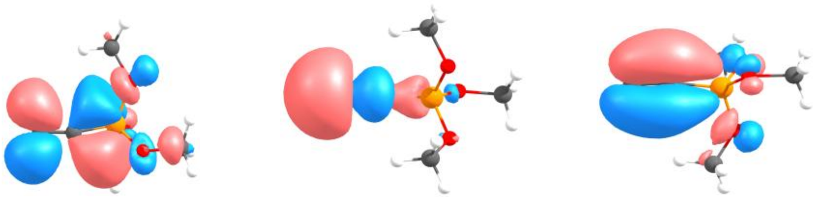

LUMO -0,32 eV | HOMO -6,54 eV | HOMO-1 -7,20 eV

Figure S159: a.-c. Frontier orbitals of $CCP(OMe)_3$ corresponding to a. the π acceptor, b. the σ donor and c. the π donor orbital.

a. b. c. d.

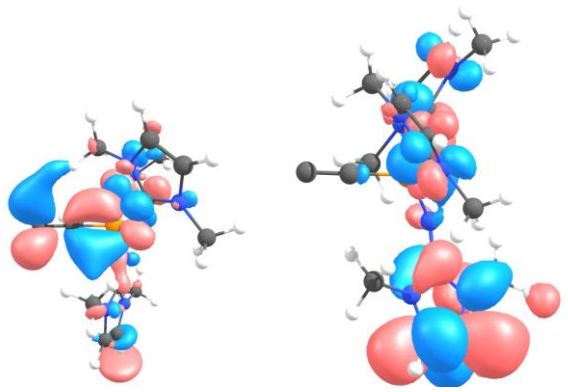

LUMO+13 2,13 eV | LUMO 0,59 eV | HOMO -4,85 eV | HOMO-1 -4,82 eV

Figure S160: a.-d. Frontier orbitals of $CCP(NIMe)_3$ corresponding to a. the π acceptor, b. the LUMO, c. the π donor and d. the σ donor orbital.

d. b. c. d.

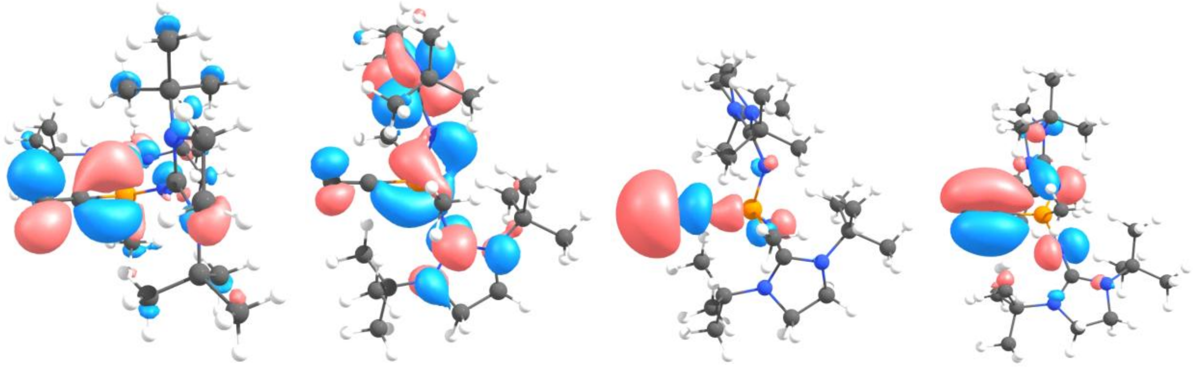

LUMO+7 1,47 eV | LUMO -0,03 eV | HOMO -5,07 eV | HOMO-1 -5,35 eV

Figure S161: d. Frontier orbitals of $CCPMe(NsI\textit{t}Bu)_2$ (**C**) corresponding to a. the π acceptor, b. the LUMO, c. the σ donor and d. the π donor orbital.

a. b. c.

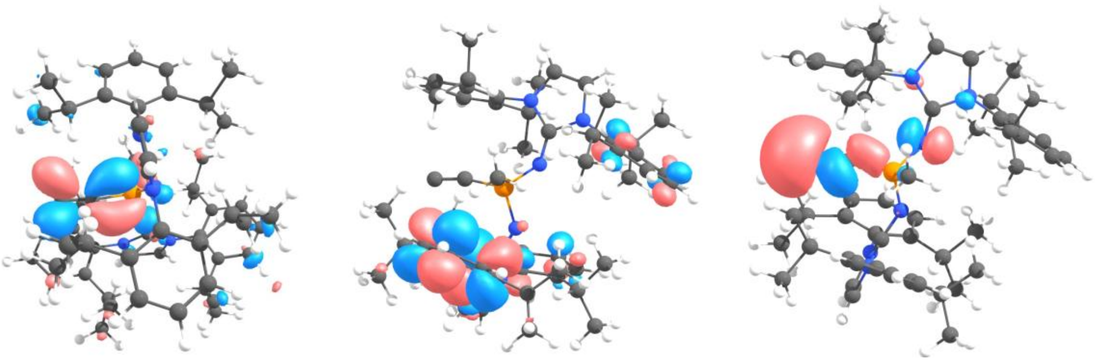

LUMO+20 1,46 eV | LUMO -0,54 eV | HOMO -5,13 eV

Figure S162: a.-c. Frontier orbitals of $CCPMe(NIdipp)_2$ (**B**) corresponding to a. the π acceptor, b. the LUMO, c. the σ donor orbital.

a.

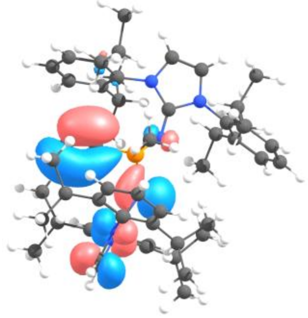

HOMO-1
-5,25 eV

Figure S163: a. Frontier orbital of CCPMe(NIdipp)$_2$ (**B**) corresponding to a. the π donor orbital.

a.

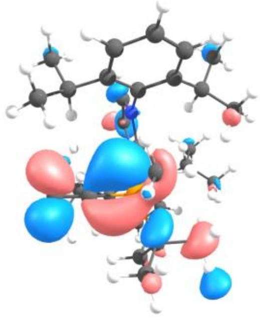

LUMO+7
0,89 eV

b.

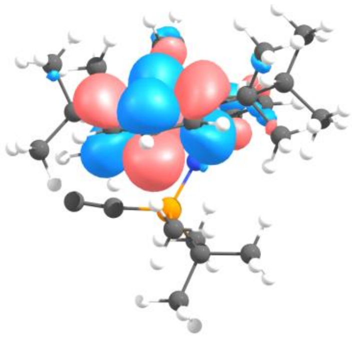

LUMO
-0,79 eV

c.

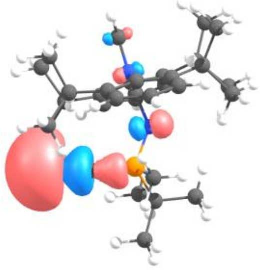

HOMO
-5,54 eV

Figure S164: a.-c. Frontier orbitals of CCPMe*t*Bu(NIdipp) (**10**) corresponding to a. the π acceptor, b. the LUMO and c. the σ donor orbital.

a.

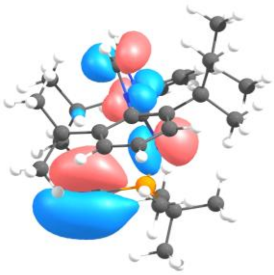

HOMO-1
-5,71 eV

Figure S165: a. Frontier orbital of CCPMe*t*Bu(NIdipp) (**10**) corresponding to the π donor orbital.

Natural Bond Orbital Analysis

NBO analysis was performed at B3LYP[11,12]/6-311G(d)[13,14] level of theory. The following table lists selected natural atomic charges of P, C_α and C_β and Wiberg bond indices.

Table S1: Selected calculated natural atomic charges and Wiberg bond indices.

Compound	*P in e*	*C_α in e*	*C_β in e*	*P–C_α*	*C_α–C_β*
$CCPF_3$	2.43	-1.10	0.20	1.27	2.28
$CCPMe_3$	1.54	-0.98	0.01	1.13	2.52
CCPMe*t*Bu(NIdipp) (**10**)	1.79	-0.95	-0.11	1.05	2.61
$CCPMe(NIdipp)_2$ (**B**)	1.95	-0.93	-0.15	1.01	2.63
CCPMe(NsI*t*Bu)$_2$ (**C**)	1.98	-0.98	-0.10	1.02	2.59
$CCP(NIMe)_3$	2.11	-0.96	-0.17	0.98	2.64

Gas phase proton affinity

Using the previous calculated geometries (B3LYP[11,12]/6-311G(d)[13,14]), all structures were reoptimized with the B3LYP[11,12] functional and the 6-311+G(2df,p) basis set[16–18,21] along with Grimme's dispersion correction (D3)[22] implemented in Gaussian 16[15]. This followed a method reported by Sundermeyer and coworkers in 2019.[23] A frequency calculation at the same level of theory was performed of the final structure to obtain ΔH and confirm the calculated structure is a local minimum.

To determine the proton affinity in the gas-phase the enthalpy of following reaction is calculated:

ΔH: $B + H^+ \rightarrow BH^+$

$PA = -\Delta H$[24]

$\Delta H = H(BH^+) - H(B) - H(H^+)$

Table S2: Calculated gas-phase proton affinities.

B	*proton affinity (kcal/mol)*
OC	140,7
MeNC	199,9
F_3PCC	222,6
Me_3CC	265,1
$(NIMe)_3CC$	297,9
IMe	261,9

EDA-NOCV analysis

EDA-NOCV analysis was performed at B3LYP[11,12]/6-311G(d)[13,14] level of theory. The calculation was performed using Orca 6.1.1[25,26].

EDA-NOCV analysis separates the interaction energy of two fragments in different energy contributions

ΔE(int) = ΔE(elstat) + ΔE(Pauli) + ΔE(orb) + ΔE(disp)

with ΔE(elstat) as energy contribution for the electrostatic interactions, ΔE(orb) as energy contribution for the orbital interactions and ΔE(disp) as energy contribution for dispersion (in this calculation the dispersion was neglected). ΔE(elstat), ΔE(orb) and ΔE(disp) combined are the energy contributions for the attractive energy contributions to the interaction energy.[27] ΔE(Pauli) is the energy contribution for the Pauli repulsion and is calculated from the sum of $\Delta\tilde{E}$(Pauli) and ΔE^0_{XC}.

With this method the ΔE(orb) is divided into contributions $\Delta E(k)^{orb}$ corresponding to pairs of orbitals.[25]

The calculation depends on the fragmentation of the molecule. The phosphonioacetylides are fragmented into the C_2 moiety and the PR_3 moiety. Here, three fragmentations are probed:

S: Cleavage into C_2 + PR_3 in the singlet state

D: Cleavage into $C_2^{-\cdot}$ + $PR_3^{+\cdot}$ in the doublet state

T: Cleavage into C_2 + PR_3 in the triplet state

Table S3: EDA-NOCV analysis of F_3PCC, Me_3PCC and $(NIMe)_3PCC$. Calculated interaction energy and energy contributions in kcal/mol are listed. The percentage of contributions to the overall attractive energy contributions is given in parenthesis.

Compound	*F_3PCC*			*Me_3PCC*			*$(NIMe)_3PCC$*		
Type of Bond Cleavage / State	S	D	T	S	D	T	S	D	T
E(int) in kcal/mol	-107,68	-285,21	-224,83	-130,47	-216,19	-233,64	-159,55	-159,83	-211,24
E(elstat) in kcal/mol	-196,32 (36%)	-377,94 (43%)	-186,86 (25%)	-200,18 (38%)	-327,03 (44%)	-172,77 (24%)	-220,18 (40%)	-320,80 (45%)	-226,39 (30%)
E(Pauli) in kcal/mol	439,60	587,51	521,39	392,26	522,44	473,74	387,14	554,12	538,30
E(orb) in kcal/mol	-351,00 (64%)	-494,79 (57%)	-559,37 (75%)	-322,52 (62%)	-411,60 (56%)	-534,60 (76%)	-326,53 (60%)	-393,17 (55%)	-523,17 (70%)

The fragmentation method where the percentage of ΔE(orb) of the attractive energy contributions is the smallest describes the bonding situation the most precise.[28] For all phosphonioacetylides this corresponds to the cleavage into the anionic C_2^- and the cationic PR_3^+ in the doublet state (see Table S3). The contributions $\Delta E(k)^{orb}$ corresponding to pairs of orbitals were examined. As the doublet state is an open shell system the energy contributions are given divided in spin-up (alpha) and spin-down (beta). The NOCV orbital pair is combined

by equivalent number of row in the output file (alpha and beta table) and the energy values added to obtain ΔE(k)^{orb}.

Table S4: EDA-NOCV analysis of F_3PCC, Me_3PCC and $(NIMe)_3PCC$. Calculated energy contributions E(k) to E(orb) corresponding to pairs of orbitals. Fragmentation in a doublet state. Spin-up E(k)$^{\alpha\text{-orb(n)}}$, spin-down E(k)$^{\beta\text{-orb(n)}}$ and total energy values E(k)$^{\text{orb(n)}}$ are listed.

Compound		F_3PCC	Me_3PCC	$(NIMe)_3PCC$
E(k)$^{\text{orb(1)}}$ in kcal/mol	spin-up (α)	-128,91	-140,98	-179,96
	spin-down (β)	-231,23	-189,31	-144,69
	Total (α+ β)	-360,14 (73%)	-330,29 (80%)	-324,65 (83%)
E(k)$^{\text{orb(2)}}$ in kcal/mol	spin-up (α)	-28,92	-15,67	-10,07
	spin-down (β)	-26,81	-9,70	-3,57
	total	-55,73 (11%)	-25,37 (6%)	-13,64 (3%)
E(k)$^{\text{orb(3)}}$ in kcal/mol	spin-up (α)	-12,64	-15,93	-13,66
	spin-down (β)	-45,26	-14,33	-9,34
	total	-57,90 (12%)	-30,26 (7%)	-23,00 (6%)
E(k)^{rest} in kcal/mol	total	-21,02 (4%)	-25,68 (6%)	-31,88 (8%)

<u>Calculated IR spectrum of **8**</u>

Geometry optimization and frequency calculation was performed at B3LYP[11,12]/6-311G(d)[13,14] level of theory.

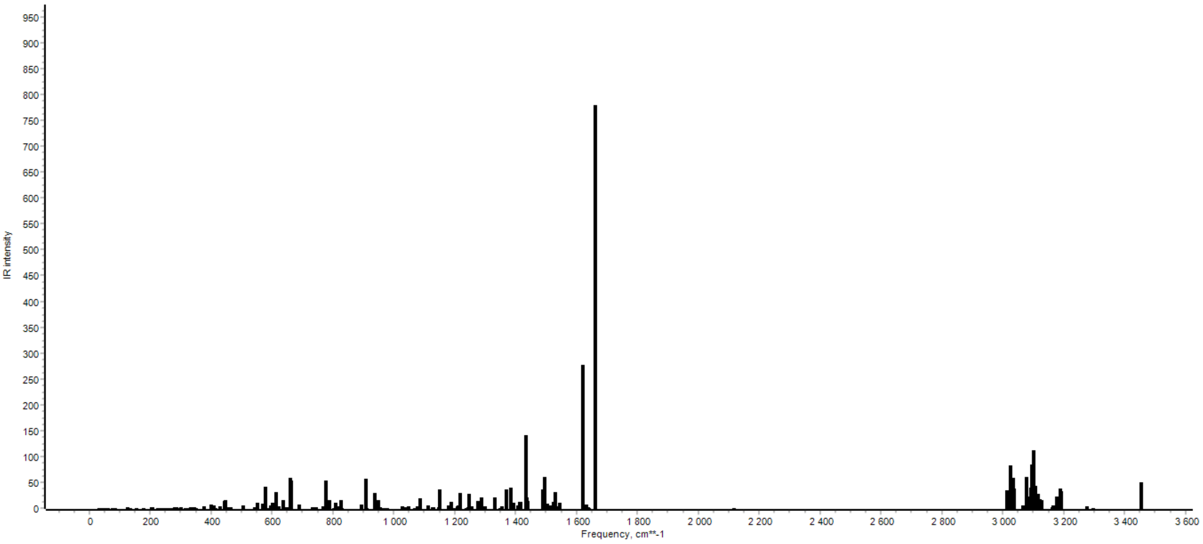

Figure S166: Calculated IR spectrum for phosphine **8** showing the low intensity CC stretch at 2120 cm^{-1}. Depicted with Chemcraft[20].

<u>Atomic coordinates of the optimized geometry of CO (B3LYP/6-311G(d))</u>

C 0.000000000 0.000000000 -0.644021000

O	0.000000000	0.000000000	0.483016000

Atomic coordinates of the optimized geometry of CO (B3LYP-D3/6-311+G(2df,p))

C	0.000000000	0.000000000	-0.642939000
O	0.000000000	0.000000000	0.482204000

Atomic coordinates of the optimized geometry of CNMe (B3LYP/6-311G(d))

C	1.482572000	-0.000078000	-0.000002000
N	0.313085000	0.000129000	0.000004000
C	-1.108092000	-0.000033000	-0.000001000
H	-1.479447000	-0.490052000	0.901434000
H	-1.479430000	-0.535771000	-0.875043000
H	-1.479594000	1.025584000	-0.026399000

Atomic coordinates of the optimized geometry of CNMe (B3LYP-D3/6-311+G(2df,p))

C	1.477849000	-0.000024000	-0.000207000
N	0.313177000	0.000040000	0.000348000
C	-1.105689000	-0.000010000	-0.000095000
H	-1.475217000	-0.832179000	0.599923000
H	-1.474760000	-0.103678000	-1.020936000
H	-1.475221000	0.935783000	0.420391000

Atomic coordinates of the optimized geometry of $CCPF_3$ (B3LYP/6-311G(d))

C	-2.725236000	0.000006000	-0.000252000
C	-1.459991000	-0.000147000	-0.000177000
P	0.141502000	-0.000015000	-0.000011000
F	0.851132000	1.207119000	0.680407000
F	0.851316000	-1.192726000	0.705196000
F	0.851865000	-0.014274000	-1.385299000

Atomic coordinates of the optimized geometry of $CCPF_3$ (B3LYP-D3/6-311+G(2df,p))

C	2.713198000	0.000006000	-0.000116000
C	1.454390000	-0.000065000	-0.000106000
P	-0.148319000	-0.000007000	-0.000051000
F	-0.843813000	1.186267000	-0.692388000
F	-0.843890000	-1.192825000	-0.680972000
F	-0.843490000	0.006609000	1.373593000

Atomic coordinates of the optimized geometry of $CCP(OMe)_3$ (B3LYP/6-311G(d))

P	-0.003070000	0.024750000	-0.237168000
C	1.296904000	-0.191701000	-1.219254000
C	2.483526000	-0.310346000	-1.616801000
O	-0.224638000	1.492569000	0.344933000
C	0.857348000	2.228689000	0.957610000

H	1.102035000	1.790355000	1.925957000
H	0.490070000	3.243606000	1.087358000
H	1.731022000	2.221703000	0.304687000
O	-1.381660000	-0.269667000	-0.967444000
C	-2.665197000	-0.056100000	-0.338497000
H	-2.810455000	1.005600000	-0.141792000
H	-2.734184000	-0.624945000	0.589958000
H	-3.404432000	-0.412711000	-1.051175000
O	0.041492000	-0.863536000	1.098827000
C	0.657603000	-2.171679000	1.139816000
H	0.013177000	-2.903790000	0.650753000
H	0.765225000	-2.414793000	2.194267000
H	1.630937000	-2.144382000	0.649741000

Atomic coordinates of the optimized geometry of $CCPMe_3$ (B3LYP/6-311G(d))

C	-2.791683000	0.000024000	0.000021000
C	-1.536429000	0.000010000	0.000041000
P	0.131174000	-0.000017000	0.000010000
C	0.845103000	0.577842000	-1.580203000
C	0.845189000	1.079622000	1.290472000
C	0.845130000	-1.657454000	0.289673000
H	1.937155000	1.060377000	1.267541000
H	0.496207000	0.746177000	2.268513000
H	0.496332000	2.101196000	1.134832000
H	1.937067000	0.567713000	-1.552060000
H	0.496034000	1.591546000	-1.780437000
H	0.496292000	-0.067773000	-2.387079000
H	1.937098000	-1.628071000	0.284477000
H	0.496125000	-2.337699000	-0.488146000
H	0.496232000	-2.033466000	1.252179000

Atomic coordinates of the optimized geometry of $CCPPh_3$ (**A**) (B3LYP/6-311G(d))

C	-0.001272000	-0.000269000	3.705536000
C	-0.000554000	0.000059000	2.451571000
P	-0.000119000	-0.000005000	0.778434000
C	-1.596059000	-0.594103000	0.105119000
C	0.283489000	1.679195000	0.105235000
C	1.312612000	-1.085107000	0.105638000
C	-2.334020000	-1.506472000	0.868090000
C	-2.084750000	-0.167840000	-1.135069000
C	-0.140916000	2.774280000	0.866747000
C	0.900244000	1.889582000	-1.133252000
C	1.186967000	-1.724243000	-1.132995000
C	2.472804000	-1.265228000	0.867647000

H	-1.971092000	-1.802636000	1.846434000
C	-3.540856000	-2.000545000	0.381746000
C	-3.294109000	-0.666264000	-1.614766000
H	-1.539775000	0.566324000	-1.718395000
H	-0.581394000	2.607948000	1.843934000
C	0.034603000	4.066506000	0.380567000
C	1.073371000	3.186201000	-1.612744000
H	1.265990000	1.050770000	-1.715425000
H	0.277883000	-1.621456000	-1.715441000
C	2.223502000	-2.522467000	-1.612140000
C	3.504331000	-2.063357000	0.381854000
H	2.548537000	-0.800712000	1.844929000
H	-4.112009000	-2.703390000	0.979029000
C	-4.019832000	-1.585001000	-0.859589000
H	-3.672990000	-0.327692000	-2.573527000
H	-0.291087000	4.912381000	0.976702000
C	0.637323000	4.273825000	-0.859105000
H	1.558609000	3.345267000	-2.570160000
H	2.119065000	-3.022095000	-2.569674000
C	3.383091000	-2.688797000	-0.857975000
H	4.399424000	-2.204376000	0.978412000
H	-4.964159000	-1.967771000	-1.232901000
H	0.778064000	5.283069000	-1.232264000
H	4.186903000	-3.315283000	-1.230841000

Atomic coordinates of the optimized geometry of $CCP(NMe_2)_3$ (B3LYP/6-311G(d))

C	-0.000413000	0.001039000	3.259796000
C	-0.000084000	0.000670000	2.006485000
P	0.000007000	0.000134000	0.336790000
N	-0.525799000	-1.469210000	-0.271899000
N	-1.009412000	1.189865000	-0.272754000
N	1.535316000	0.279115000	-0.272333000
C	0.011587000	-2.107745000	-1.464469000
C	-1.633656000	-2.177094000	0.367624000
C	1.819367000	1.062364000	-1.465924000
C	2.702473000	-0.325448000	0.367776000
C	-1.830972000	1.043374000	-1.465381000
C	-1.068577000	2.503440000	0.366393000
H	-1.849019000	-1.733245000	1.338548000
H	-1.356867000	-3.223596000	0.530494000
H	-2.540462000	-2.149873000	-0.249865000
H	0.883925000	-1.568237000	-1.828518000
H	-0.729757000	-2.151339000	-2.273771000
H	0.318168000	-3.134298000	-1.235321000

H	-1.799608000	0.018149000	-1.829442000
H	-1.498114000	1.707255000	-2.274651000
H	-2.873391000	1.290882000	-1.236416000
H	-0.576670000	2.468145000	1.337414000
H	-2.113258000	2.787166000	0.528990000
H	-0.591403000	3.274931000	-0.251203000
H	0.915837000	1.547316000	-1.830722000
H	2.228010000	0.441168000	-2.274366000
H	2.554895000	1.841678000	-1.237912000
H	2.425882000	-0.732820000	1.339177000
H	3.470337000	0.437757000	0.529664000
H	3.132312000	-1.125036000	-0.248847000

Atomic coordinates of the optimized geometry of CCPMe*t*Bu(NIdipp) (**10**) (B3LYP/6-311G(d))

N	0.109479000	-0.003348000	0.891505000
C	-0.021252000	0.284796000	-0.374008000
P	-0.353443000	-1.297060000	1.780310000
N	1.026634000	0.551292000	-1.235213000
N	-1.162684000	0.520378000	-1.125293000
C	-0.408509000	-2.746985000	0.906481000
C	-1.980085000	-1.006665000	2.572596000
C	0.885491000	-1.409577000	3.181140000
C	0.538181000	0.975928000	-2.467307000
C	2.432821000	0.438695000	-0.927478000
C	-0.806736000	0.956006000	-2.401598000
C	-2.523094000	0.527699000	-0.642982000
C	-0.351590000	-3.737627000	0.145133000
H	-1.976169000	-0.085547000	3.156982000
H	-2.234046000	-1.848622000	3.217178000
H	-2.739463000	-0.926492000	1.796433000
C	0.936730000	-0.075818000	3.945419000
C	0.474756000	-2.555471000	4.124022000
C	2.263558000	-1.717696000	2.572740000
H	1.201335000	1.247643000	-3.269891000
C	3.098570000	-0.769705000	-1.216784000
C	3.096744000	1.563305000	-0.404926000
H	-1.552399000	1.216214000	-3.131922000
C	-2.974650000	1.662287000	0.060098000
C	-3.366155000	-0.562948000	-0.942740000
H	1.184955000	0.754105000	3.281571000
H	1.707932000	-0.129384000	4.720813000
H	-0.007422000	0.154339000	4.446417000
H	0.366250000	-3.498874000	3.584732000
H	-0.461420000	-2.348863000	4.648806000

H	1.248603000	-2.690961000	4.886282000
H	2.249865000	-2.664376000	2.029752000
H	3.004933000	-1.796543000	3.374706000
H	2.587138000	-0.933150000	1.887595000
C	4.473366000	-0.816988000	-0.967571000
C	2.380778000	-1.982095000	-1.801200000
C	4.471098000	1.455873000	-0.172632000
C	2.384840000	2.879939000	-0.115727000
C	-4.302131000	1.669115000	0.497221000
C	-2.097131000	2.882578000	0.319742000
C	-4.687208000	-0.493310000	-0.487819000
C	-2.903938000	-1.766576000	-1.758384000
H	5.018253000	-1.730657000	-1.173352000
C	5.153483000	0.279749000	-0.452417000
H	1.330814000	-1.930603000	-1.506323000
C	2.910962000	-3.320734000	-1.264076000
C	2.444244000	-1.972735000	-3.341943000
H	5.013836000	2.304948000	0.229161000
H	1.315570000	2.728368000	-0.275826000
C	2.560225000	3.324206000	1.346198000
C	2.840448000	3.984685000	-1.087181000
H	-4.679300000	2.525566000	1.046004000
C	-5.149995000	0.602768000	0.229357000
H	-1.095687000	2.670677000	-0.058062000
C	-1.953485000	3.188843000	1.819929000
C	-2.619648000	4.110641000	-0.449343000
H	-5.362082000	-1.315412000	-0.693930000
H	-1.817610000	-1.833538000	-1.675908000
C	-3.462477000	-3.100946000	-1.239257000
C	-3.266650000	-1.588869000	-3.247017000
H	6.221142000	0.215490000	-0.266315000
H	2.993240000	-3.313178000	-0.176482000
H	3.889236000	-3.576668000	-1.684175000
H	2.211493000	-4.117378000	-1.521877000
H	3.480088000	-2.024691000	-3.692174000
H	1.992181000	-1.075254000	-3.770690000
H	1.910056000	-2.837287000	-3.745507000
H	2.215232000	2.551286000	2.035515000
H	1.981979000	4.232620000	1.539852000
H	3.604299000	3.545978000	1.583961000
H	3.905141000	4.206423000	-0.970685000
H	2.287267000	4.910861000	-0.904536000
H	2.676411000	3.696518000	-2.128895000
H	-6.178524000	0.627640000	0.575810000

H	-2.914569000	3.433912000	2.280714000
H	-1.290210000	4.045496000	1.971204000
H	-1.526613000	2.338044000	2.353696000
H	-2.688528000	3.912049000	-1.522046000
H	-1.949929000	4.964148000	-0.308356000
H	-3.613331000	4.410003000	-0.104345000
H	-4.536313000	-3.200985000	-1.428303000
H	-3.274246000	-3.226928000	-0.173259000
H	-2.957141000	-3.927046000	-1.742697000
H	-2.908335000	-2.444196000	-3.826424000
H	-2.824803000	-0.688409000	-3.680273000
H	-4.350818000	-1.523856000	-3.382703000

Atomic coordinates of the optimized geometry of CCPMe(NIdipp)$_2$ (**B**) (B3LYP/6-311G(d))

P	0.198400000	0.707644000	-0.434537000
N	-1.255387000	-0.047727000	-0.299594000
N	1.298556000	-0.436289000	-0.050146000
C	0.476605000	2.126813000	0.459428000
C	0.307420000	1.114526000	-2.217412000
C	-2.495864000	0.254160000	-0.498006000
C	2.527543000	-0.375060000	0.365617000
C	0.931039000	3.098452000	1.101195000
H	0.245918000	0.196707000	-2.802682000
H	1.248905000	1.616082000	-2.435813000
H	-0.513928000	1.775209000	-2.499081000
N	-3.253416000	1.397436000	-0.235659000
N	-3.419305000	-0.651457000	-1.025431000
N	3.679792000	0.042867000	-0.293656000
N	2.988829000	-0.878895000	1.571809000
C	-4.589614000	1.168423000	-0.579180000
C	-2.880704000	2.559306000	0.541025000
C	-4.691567000	-0.083094000	-1.058032000
C	-3.145593000	-2.013583000	-1.400999000
C	4.802824000	-0.246151000	0.481066000
C	3.777278000	0.508096000	-1.654876000
C	4.377480000	-0.813688000	1.625078000
C	2.172044000	-1.411351000	2.632189000
H	-5.334792000	1.930424000	-0.430175000
C	-2.936163000	2.471068000	1.944183000
C	-2.582878000	3.761749000	-0.124291000
H	-5.542474000	-0.632988000	-1.421493000
C	-2.750065000	-2.293671000	-2.723719000
C	-3.347230000	-3.029553000	-0.449231000
H	5.795682000	-0.027750000	0.129242000
C	3.947808000	1.886680000	-1.901094000
C	3.770847000	-0.447980000	-2.689368000
H	4.924344000	-1.177731000	2.477180000
C	1.699852000	-2.731833000	2.521227000
C	1.929980000	-0.612100000	3.767084000
C	-2.629120000	3.618598000	2.677105000
C	-3.337666000	1.197245000	2.678566000
C	-2.303070000	4.882614000	0.657966000
C	-2.570816000	3.876080000	-1.643690000
C	-2.542470000	-3.630673000	-3.070900000
C	-2.608076000	-1.193733000	-3.769908000
C	-3.124905000	-4.350901000	-0.849426000
C	-3.832868000	-2.742688000	0.966813000

C	4.085882000	2.286839000	-3.234301000
C	4.011900000	2.922149000	-0.782673000
C	3.896787000	0.013827000	-4.002676000
C	3.675470000	-1.947993000	-2.430679000
C	0.934605000	-3.237000000	3.575651000
C	2.019746000	-3.621562000	1.325910000
C	1.168720000	-1.174832000	4.796199000
C	2.511393000	0.789763000	3.929480000
H	-2.637215000	3.577328000	3.761128000
C	-2.309187000	4.810585000	2.043413000
H	-3.551358000	0.427563000	1.934665000
C	-4.629509000	1.402603000	3.491241000
C	-2.202112000	0.662379000	3.564937000
H	-2.047926000	5.820159000	0.177523000
H	-2.554166000	2.861855000	-2.052081000
C	-3.852126000	4.554303000	-2.165375000
C	-1.321346000	4.607399000	-2.165030000
H	-2.236526000	-3.881469000	-4.080146000
C	-2.723562000	-4.650307000	-2.143607000
H	-2.416873000	-0.257616000	-3.240465000
C	-1.433107000	-1.419775000	-4.734282000
C	-3.918748000	-1.018610000	-4.562684000
H	-3.271108000	-5.155997000	-0.137097000
H	-3.878331000	-1.660033000	1.093994000
C	-5.257225000	-3.287358000	1.183987000
C	-2.867479000	-3.277956000	2.036355000
H	4.213858000	3.338744000	-3.460914000
C	4.051443000	1.366383000	-4.274410000
H	3.490955000	2.515583000	0.085950000
C	5.472799000	3.211199000	-0.382294000
C	3.298012000	4.236766000	-1.134058000
H	3.886339000	-0.697331000	-4.822062000
H	3.599883000	-2.104045000	-1.353597000
C	4.948816000	-2.675004000	-2.901893000
C	2.416924000	-2.565695000	-3.061870000
H	0.548722000	-4.249402000	3.518019000
C	0.668747000	-2.467274000	4.700885000
H	2.649050000	-3.050753000	0.641087000
C	2.826393000	-4.862645000	1.750334000
C	0.753223000	-4.014655000	0.549044000
H	0.962726000	-0.589602000	5.685292000
H	2.866632000	1.127022000	2.954172000
C	3.715666000	0.764123000	4.892612000
C	1.477218000	1.827363000	4.391250000

H	-2.058045000	5.686959000	2.631867000
H	-4.492340000	2.139262000	4.287821000
H	-4.939616000	0.464094000	3.961229000
H	-5.451589000	1.749391000	2.859035000
H	-1.298635000	0.482000000	2.981734000
H	-2.496233000	-0.280994000	4.035106000
H	-1.949985000	1.365318000	4.363653000
H	-4.751548000	4.007638000	-1.870262000
H	-3.841349000	4.613546000	-3.258290000
H	-3.942311000	5.573003000	-1.776908000
H	-1.358427000	5.678125000	-1.945773000
H	-1.247991000	4.505401000	-3.252280000
H	-0.411720000	4.209961000	-1.711990000
H	-2.555042000	-5.682618000	-2.434406000
H	-0.504868000	-1.631877000	-4.200086000
H	-1.273463000	-0.527583000	-5.345861000
H	-1.618977000	-2.249301000	-5.422486000
H	-4.173158000	-1.934770000	-5.104236000
H	-3.821370000	-0.211914000	-5.295568000
H	-4.758833000	-0.773211000	-3.909439000
H	-5.961117000	-2.878947000	0.453784000
H	-5.620224000	-3.024385000	2.181943000
H	-5.288065000	-4.377484000	1.097363000
H	-2.784233000	-4.368324000	2.000584000
H	-3.225659000	-3.011029000	3.035025000
H	-1.868757000	-2.857067000	1.914704000
H	4.152768000	1.705277000	-5.300947000
H	5.996550000	2.315170000	-0.041457000
H	5.501836000	3.939843000	0.432686000
H	6.037644000	3.626543000	-1.223212000
H	3.847728000	4.821492000	-1.879358000
H	3.197350000	4.845281000	-0.233974000
H	2.287516000	4.057137000	-1.501932000
H	5.078518000	-2.605601000	-3.985670000
H	4.899376000	-3.736932000	-2.643460000
H	5.844637000	-2.257613000	-2.434630000
H	1.514277000	-2.106453000	-2.655923000
H	2.369928000	-3.637589000	-2.848100000
H	2.407832000	-2.446519000	-4.149373000
H	0.075313000	-2.879273000	5.511488000
H	3.744174000	-4.585764000	2.276465000
H	3.107192000	-5.453424000	0.873051000
H	2.250054000	-5.514483000	2.413109000
H	0.079619000	-4.622814000	1.159848000

H	1.016178000	-4.605008000	-0.334246000
H	0.211454000	-3.128721000	0.215656000
H	3.409642000	0.467858000	5.900828000
H	4.169284000	1.757054000	4.960515000
H	4.491020000	0.064875000	4.567731000
H	0.655174000	1.906584000	3.681404000
H	1.944576000	2.813805000	4.439412000
H	1.077734000	1.599343000	5.384713000

Atomic coordinates of the optimized geometry of CCPMe(NsI*t*Bu)$_2$ (**C**) (B3LYP/6-311G(d))

C	0.664187000	-3.436069000	0.677761000
C	0.369882000	-2.339697000	0.150473000
P	0.064365000	-0.820270000	-0.539660000
N	1.343657000	0.160947000	-0.334215000
N	-1.268712000	-0.020302000	-0.045149000
C	-0.239002000	-1.062133000	-2.333059000
C	2.527489000	0.128396000	0.201266000
C	-2.530525000	0.146246000	-0.250362000
H	-0.341590000	-0.102993000	-2.842103000
H	0.572820000	-1.630545000	-2.784778000
H	-1.163593000	-1.628634000	-2.457724000
N	2.830469000	0.314169000	1.539041000
N	3.715361000	-0.004325000	-0.503549000
N	-3.581166000	-0.686271000	0.091225000
N	-3.062890000	1.306082000	-0.829974000
C	4.278341000	0.464953000	1.692167000
C	1.932119000	0.955938000	2.557240000
C	4.813214000	-0.205049000	0.440650000
C	3.854148000	-0.480807000	-1.913869000
C	-4.808517000	-0.190068000	-0.529243000
C	-3.515684000	-2.065702000	0.679715000
C	-4.515722000	1.292709000	-0.669609000
C	-2.357642000	2.629842000	-0.845622000
H	4.561742000	1.527029000	1.718596000
H	4.643015000	-0.012758000	2.599403000
C	1.621689000	2.400525000	2.126467000
C	0.642221000	0.146885000	2.754976000
C	2.667572000	0.973636000	3.911141000
H	4.993408000	-1.275106000	0.609882000
H	5.739249000	0.253709000	0.099161000
C	5.329286000	-0.337297000	-2.337301000
C	3.459491000	-1.965652000	-2.022948000
C	3.037340000	0.420946000	-2.854993000
H	-4.973007000	-0.659233000	-1.509994000

H	-5.685205000	-0.370397000	0.087246000
C	-4.931257000	-2.460107000	1.145550000
C	-2.612275000	-2.066568000	1.922207000
C	-3.048645000	-3.091095000	-0.368430000
H	-4.824988000	1.835337000	0.236075000
H	-5.025365000	1.732189000	-1.525843000
C	-2.064038000	3.087229000	0.594692000
C	-1.061897000	2.565799000	-1.670039000
C	-3.278243000	3.658567000	-1.529519000
H	2.535176000	2.998556000	2.048858000
H	1.118055000	2.413223000	1.159931000
H	0.969922000	2.885394000	2.858651000
H	0.155144000	0.467495000	3.680797000
H	-0.068132000	0.293888000	1.946970000
H	0.859417000	-0.919468000	2.830077000
H	2.921099000	-0.037399000	4.239795000
H	3.576598000	1.578480000	3.900619000
H	2.003593000	1.406748000	4.661349000
H	5.416622000	-0.599984000	-3.393513000
H	5.684299000	0.690307000	-2.221435000
H	5.995572000	-1.003259000	-1.785230000
H	4.142814000	-2.590927000	-1.441674000
H	2.458618000	-2.164359000	-1.644722000
H	3.514874000	-2.299417000	-3.063425000
H	3.159342000	0.079206000	-3.886491000
H	1.982421000	0.425175000	-2.608613000
H	3.398792000	1.451131000	-2.793399000
H	-5.634827000	-2.579842000	0.317971000
H	-4.868592000	-3.422844000	1.655045000
H	-5.342215000	-1.738413000	1.856895000
H	-2.965065000	-1.325189000	2.645165000
H	-2.650580000	-3.051435000	2.393712000
H	-1.571862000	-1.872772000	1.685486000
H	-1.996376000	-2.970233000	-0.617625000
H	-3.155433000	-4.104851000	0.026522000
H	-3.655030000	-3.022180000	-1.277653000
H	-1.410925000	2.376143000	1.099017000
H	-1.571473000	4.063659000	0.594131000
H	-2.986169000	3.183691000	1.175917000
H	-1.263967000	2.156980000	-2.663559000
H	-0.668696000	3.578273000	-1.800915000
H	-0.293081000	1.967121000	-1.190995000
H	-4.197902000	3.846302000	-0.971793000
H	-2.749096000	4.610402000	-1.605460000

H	-3.543122000	3.345182000	-2.542938000

Atomic coordinates of the optimized geometry of $CCP(NIMe)_3$ (B3LYP/6-311G(d))

P	-0.101697000	0.039599000	0.347857000
C	-0.094408000	0.273936000	2.040247000
C	-0.074383000	0.485890000	3.271436000
N	-0.011813000	-1.549627000	-0.083291000
C	0.964774000	-2.407311000	-0.091941000
N	-1.472264000	0.627781000	-0.349691000
C	-2.735164000	0.346342000	-0.224696000
N	1.171303000	0.781541000	-0.383692000
C	1.696831000	1.965844000	-0.275685000
N	2.921316000	2.270969000	-0.851782000
N	1.289937000	3.142274000	0.324062000
C	3.256653000	3.593952000	-0.597623000
C	2.254847000	4.126767000	0.129517000
C	3.703485000	1.328496000	-1.624183000
H	3.130834000	0.405947000	-1.689524000
H	4.663439000	1.127054000	-1.140395000
H	3.884957000	1.712419000	-2.631563000
C	0.037955000	3.372433000	1.043529000
H	0.083091000	2.968721000	2.053608000
H	-0.780723000	2.887545000	0.515138000
H	-0.139999000	4.448443000	1.068086000
H	2.133371000	5.116408000	0.536368000
H	4.174342000	4.028866000	-0.956431000
N	2.172996000	-2.487840000	0.576019000
N	0.912710000	-3.559308000	-0.861596000
C	2.054973000	-4.322455000	-0.665894000
C	2.833944000	-3.658483000	0.211325000
H	2.204391000	-5.264881000	-1.165621000
H	3.796867000	-3.909007000	0.623533000
C	2.697847000	-1.542265000	1.557032000
H	3.359378000	-2.085285000	2.233096000
H	1.887872000	-1.103651000	2.137263000
H	3.254376000	-0.740164000	1.069595000
C	-0.199037000	-3.889932000	-1.729136000
H	-0.796136000	-2.989400000	-1.856782000
H	-0.825977000	-4.672745000	-1.291594000
H	0.170243000	-4.227351000	-2.699923000
N	-3.450583000	-0.512802000	0.588336000
N	-3.694659000	0.973042000	-1.004878000
C	-2.903197000	-1.403738000	1.611135000
H	-2.155457000	-2.062182000	1.171718000

H	-3.725495000	-2.000328000	2.006153000
H	-2.434618000	-0.836537000	2.413909000
C	-4.810572000	-0.392068000	0.315218000
C	-4.962657000	0.523174000	-0.662609000
C	-3.382082000	1.969833000	-2.007750000
H	-2.317118000	1.902958000	-2.220549000
H	-3.610081000	2.979067000	-1.651295000
H	-3.951553000	1.775482000	-2.919163000
H	-5.542040000	-0.973512000	0.850523000
H	-5.849964000	0.893438000	-1.147950000

Atomic coordinates of the optimized geometry of $CCP(NIMe)_3$ (B3LYP-D3/6-311+G(2df,p))

P	0.188708000	0.128421000	0.154048000
C	0.429828000	-0.235266000	1.804926000
C	0.620294000	-0.560916000	2.989430000
N	-0.451698000	1.616387000	-0.093895000
C	-1.681976000	2.020347000	-0.051880000
N	1.600818000	0.074241000	-0.660262000
C	2.805961000	0.416549000	-0.328900000
N	-0.852940000	-0.915658000	-0.552360000
C	-1.119028000	-2.161952000	-0.320382000
N	-2.349227000	-2.723075000	-0.608444000
N	-0.367884000	-3.201302000	0.180245000
C	-2.346615000	-4.071412000	-0.289157000
C	-1.128627000	-4.361610000	0.205521000
C	-3.445465000	-1.996889000	-1.210707000
H	-3.170940000	-0.944595000	-1.242814000
H	-4.354143000	-2.122484000	-0.618729000
H	-3.630915000	-2.345708000	-2.229683000
C	1.040599000	-3.148510000	0.549823000
H	1.161094000	-2.881398000	1.598116000
H	1.537358000	-2.392730000	-0.054299000
H	1.484163000	-4.123165000	0.342272000
H	-0.730463000	-5.286962000	0.581464000
H	-3.210297000	-4.695285000	-0.434406000
N	-2.790515000	1.596846000	0.652072000
N	-2.122026000	3.106215000	-0.780674000
C	-3.461475000	3.345987000	-0.526560000
C	-3.875403000	2.410107000	0.349148000
H	-3.995973000	4.155781000	-0.990096000
H	-4.840275000	2.251513000	0.796577000
C	-2.830725000	0.535952000	1.648849000
H	-3.583273000	0.792526000	2.394323000
H	-1.863785000	0.441569000	2.139011000

H	-3.080456000	-0.422570000	1.193862000
C	-1.260485000	3.866076000	-1.660230000
H	-0.522682000	3.192501000	-2.092952000
H	-0.732668000	4.652712000	-1.114874000
H	-1.861216000	4.316242000	-2.450132000
N	3.306354000	1.317917000	0.583786000
N	3.921268000	-0.120614000	-0.942367000
C	2.529315000	2.201171000	1.444529000
H	1.612981000	2.489429000	0.933237000
H	3.125223000	3.092884000	1.641373000
H	2.262261000	1.702462000	2.374863000
C	4.693496000	1.306766000	0.541477000
C	5.074186000	0.424471000	-0.401398000
C	3.863865000	-1.144163000	-1.962262000
H	2.836272000	-1.210695000	-2.313445000
H	4.169155000	-2.114545000	-1.561836000
H	4.517370000	-0.879878000	-2.795424000
H	5.280568000	1.932884000	1.189060000
H	6.054506000	0.136262000	-0.736262000

Atomic coordinates of the optimized geometry of $[HCO]^+$ (B3LYP-D3/6-311+G(2df,p))

C	0.000000000	0.000000000	0.514347000
O	0.000000000	0.000000000	-0.587227000
H	0.000000000	0.000000000	1.611736000

Atomic coordinates of the optimized geometry of $[HCNMe]^+$ (B3LYP-D3/6-311+G(2df,p))

C	1.345606000	0.000101000	0.000071000
N	0.212482000	-0.000715000	0.000109000
C	-1.219197000	0.000258000	-0.000060000
H	-1.556992000	-1.007831000	-0.241816000
H	-1.555835000	0.714003000	-0.752419000
H	-1.556219000	0.295292000	0.994002000
H	2.423224000	0.001390000	-0.000593000

Atomic coordinates of the optimized geometry of $[HCCPF_3]^+$ (B3LYP-D3/6-311+G(2df,p))

C	2.588915000	0.000045000	0.000013000
C	1.385910000	-0.000027000	0.000043000
P	-0.285278000	0.000005000	0.000005000
F	-0.860540000	0.892076000	1.070906000
F	-0.860444000	0.481386000	-1.308046000
F	-0.860374000	-1.373508000	0.237097000
H	3.662438000	0.000223000	-0.000024000

Atomic coordinates of the optimized geometry of $[HCCPMe_3]^+$ (B3LYP-D3/6-311+G(2df,p))

C	2.653632000	0.000230000	-0.000049000
C	1.452711000	0.000547000	-0.000145000
P	-0.277557000	0.000015000	0.000005000
C	-0.857548000	1.693271000	0.201816000
C	-0.856982000	-0.672147000	-1.567389000
C	-0.856639000	-1.021634000	1.365700000
H	-1.948422000	-0.675729000	-1.574377000
H	-0.486876000	-1.690796000	-1.686012000
H	-0.487734000	-0.055342000	-2.387084000
H	-1.948993000	1.700672000	0.202899000
H	-0.488208000	2.305395000	-0.621340000
H	-0.487870000	2.094982000	1.145565000
H	-1.948077000	-1.026506000	1.372093000
H	-0.486923000	-0.614852000	2.307260000
H	-0.486810000	-2.039708000	1.241421000
H	3.722232000	0.000054000	-0.000098000

Atomic coordinates of the optimized geometry of $[HCCPNIMe_3]^+$ (B3LYP-D3/6-311+G(2df,p))

P	0.000766000	0.000443000	0.801281000
C	-0.000454000	-0.000926000	2.579547000
C	-0.000975000	-0.001811000	3.782967000
N	-0.155197000	1.527249000	0.326567000
C	-1.189867000	2.216210000	-0.090350000
N	1.401479000	-0.627298000	0.327733000
C	2.515687000	-0.077377000	-0.090126000
N	-1.243196000	-0.897575000	0.325176000
C	-1.324323000	-2.137735000	-0.091998000
N	-2.120844000	-2.535325000	-1.130852000
N	-0.748599000	-3.280619000	0.388798000
C	-2.041348000	-3.909970000	-1.286807000
C	-1.187116000	-4.369751000	-0.351875000
C	-2.950775000	-1.639058000	-1.914931000
H	-2.600433000	-0.621963000	-1.756317000
H	-3.995246000	-1.709354000	-1.605478000
H	-2.870009000	-1.895229000	-2.970891000
C	0.113471000	-3.358622000	1.559729000
H	-0.298198000	-2.741490000	2.356822000
H	1.116624000	-3.006419000	1.326178000
H	0.147231000	-4.393457000	1.893713000
H	-0.853817000	-5.369908000	-0.140353000
H	-2.596384000	-4.431684000	-2.045741000
N	-2.467299000	2.288487000	0.391080000
N	-1.137649000	3.103082000	-1.130743000

C	-2.368657000	3.719816000	-1.287130000
C	-3.192791000	3.210543000	-0.350857000
H	-2.544124000	4.460131000	-2.046985000
H	-4.225680000	3.421238000	-0.138974000
C	-2.966094000	1.580070000	1.561340000
H	-3.874506000	2.073203000	1.900873000
H	-2.222533000	1.620216000	2.355661000
H	-3.170704000	0.537356000	1.324913000
C	0.051968000	3.372061000	-1.917742000
H	0.761267000	2.564835000	-1.751456000
H	0.508804000	4.316859000	-1.617342000
H	-0.210576000	3.418881000	-2.974223000
N	3.216286000	0.994521000	0.388362000
N	3.258850000	-0.569545000	-1.128026000
C	2.851500000	1.783530000	1.556512000
H	2.053260000	2.483860000	1.317107000
H	3.733480000	2.321591000	1.897533000
H	2.510574000	1.121556000	2.350929000
C	4.378195000	1.159637000	-0.353104000
C	4.408467000	0.187920000	-1.285840000
C	2.898739000	-1.738028000	-1.910226000
H	1.844361000	-1.947329000	-1.746288000
H	3.487030000	-2.604884000	-1.603508000
H	3.074094000	-1.539537000	-2.967158000
H	5.076439000	1.949989000	-0.143498000
H	5.138076000	-0.032596000	-2.044316000
H	-0.001086000	-0.002384000	4.848267000

Atomic coordinates of the optimized geometry of IMe (B3LYP-D3/6-311+G(2df,p))

C	0.000002000	0.978907000	-0.000626000
N	1.060448000	0.123686000	-0.000055000
N	-1.060450000	0.123683000	-0.000067000
C	0.675691000	-1.208391000	0.000045000
C	-0.675691000	-1.208387000	-0.000298000
C	2.441209000	0.567760000	0.000334000
H	2.442037000	1.654782000	-0.000345000
H	2.963309000	0.205674000	-0.888283000
H	2.962531000	0.206792000	0.889868000
C	-2.441209000	0.567760000	0.000300000
H	-2.963154000	0.206109000	-0.888586000
H	-2.442034000	1.654782000	0.000145000
H	-2.962690000	0.206364000	0.889567000
H	-1.376559000	-2.025989000	-0.000355000
H	1.376562000	-2.025989000	0.000324000

Atomic coordinates of the optimized geometry of $[IMe]^{+}$ (B3LYP-D3/6-311+G(2df,p))

C	0.000005000	0.860514000	0.000042000
N	1.084441000	0.085230000	0.000004000
N	-1.084454000	0.085211000	-0.000025000
C	0.678671000	-1.232410000	-0.000037000
C	-0.678671000	-1.232399000	-0.000035000
C	2.475875000	0.549550000	0.000086000
H	2.488157000	1.636621000	-0.000339000
H	2.979555000	0.180108000	-0.891443000
H	2.979239000	0.180791000	0.892080000
C	-2.475864000	0.549549000	-0.000046000
H	-2.979417000	0.180415000	-0.891779000
H	-2.488142000	1.636621000	-0.000112000
H	-2.979406000	0.180521000	0.891737000
H	-1.381609000	-2.047158000	-0.000074000
H	1.381652000	-2.047128000	-0.000059000
H	-0.000025000	1.937289000	0.000077000

Atomic coordinates of the optimized geometry of P(CCH)MeNIdipp (**8**) (B3LYP/6-311G(d))

N	0,231111000	0,123967000	0,875315000
C	0,105579000	-0,091379000	-0,389038000
P	-0,954688000	0,646838000	1,991460000
N	1,211711000	-0,338451000	-1,209655000
N	-0,986415000	-0,167104000	-1,266378000
C	-1,276230000	-0,850589000	2,925675000
C	0,153808000	1,542162000	3,263498000
C	0,804533000	-0,559390000	-2,524732000
C	2,575431000	-0,383714000	-0,760477000
C	-0,533915000	-0,451004000	-2,562997000
C	-2,390149000	-0,050805000	-0,962896000
C	-1,612697000	-1,822456000	3,563566000
C	0,630347000	2,831711000	2,575437000
C	-0,726430000	1,894889000	4,472526000
C	1,363257000	0,707259000	3,696556000
H	1,515522000	-0,778122000	-3,302740000
C	3,062443000	-1,566337000	-0,173165000
C	3,395622000	0,744475000	-0,957083000
H	-1,225805000	-0,546181000	-3,381782000
C	-2,990614000	1,223638000	-0,986764000
C	-3,136979000	-1,226047000	-0,747275000
H	1,236315000	2,605605000	1,695668000
H	1,245856000	3,421282000	3,264072000
H	-0,208177000	3,460368000	2,260119000
H	-1,080236000	1,000523000	4,990507000

H	-1,601965000	2,483687000	4,181185000
H	-0,152837000	2,490469000	5,191536000
H	1,055784000	-0,195703000	4,229006000
H	2,003974000	1,290762000	4,369211000
H	1,959441000	0,407596000	2,833284000
C	4,401793000	-1,593035000	0,225487000
C	2,193069000	-2,801882000	0,023680000
C	4,730876000	0,659800000	-0,552890000
C	2,879856000	2,020068000	-1,614454000
C	-4,367607000	1,299036000	-0,771076000
C	-2,189817000	2,488172000	-1,274804000
C	-4,512091000	-1,091992000	-0,532944000
C	-2,507714000	-2,614974000	-0,788084000
H	4,803221000	-2,489319000	0,686744000
C	5,230050000	-0,494323000	0,037419000
H	1,198919000	-2,577681000	-0,367517000
C	2,024952000	-3,145983000	1,513344000
C	2,733657000	-4,003674000	-0,772526000
H	5,388924000	1,510105000	-0,696340000
H	1,789851000	1,957306000	-1,651006000
C	3,234647000	3,284335000	-0,813807000
C	3,385849000	2,141362000	-3,064698000
H	-4,859054000	2,265493000	-0,778309000
C	-5,121992000	0,154758000	-0,542100000
H	-1,134475000	2,256589000	-1,113915000
C	-2,545869000	3,648232000	-0,331238000
C	-2,348866000	2,914096000	-2,747209000
H	-5,114558000	-1,976710000	-0,358955000
H	-1,423650000	-2,492931000	-0,730542000
C	-2,927163000	-3,495429000	0,400510000
C	-2,824985000	-3,322640000	-2,120110000
H	6,268532000	-0,537417000	0,351089000
H	1,584063000	-2,309638000	2,056674000
H	2,982328000	-3,396180000	1,980694000
H	1,364687000	-4,010608000	1,631736000
H	3,719277000	-4,317010000	-0,416256000
H	2,825611000	-3,771784000	-1,837212000
H	2,061312000	-4,861050000	-0,672537000
H	2,903975000	3,207069000	0,223591000
H	2,751174000	4,159831000	-1,257113000
H	4,310729000	3,479048000	-0,807645000
H	4,477241000	2,211623000	-3,097776000
H	2,979177000	3,037824000	-3,542512000
H	3,092933000	1,279557000	-3,669383000

H	-6,191191000	0,236036000	-0,372242000
H	-3,552754000	4,034465000	-0,513713000
H	-1,851536000	4,480140000	-0,480731000
H	-2,485526000	3,337917000	0,713513000
H	-2,028944000	2,125573000	-3,432873000
H	-1,748195000	3,804180000	-2,958298000
H	-3,391657000	3,152616000	-2,977415000
H	-3,988842000	-3,756206000	0,363663000
H	-2,730949000	-2,996398000	1,350258000
H	-2,365241000	-4,434124000	0,386516000
H	-2,340478000	-4,302935000	-2,159600000
H	-2,478733000	-2,745532000	-2,980652000
H	-3,901826000	-3,477931000	-2,236498000
H	-1,892639000	-2,683687000	4,123634000

References

[1] M. M. Rahman, G. Meng, E. Bisz, B. Dziuk, R. Lalancette, R. Szostak, M. Szostak, *Chem. Sci.* **2023**, *14*, 5141.

[2] M. Tamm, S. Randoll, E. Herdtweck, N. Kleigrewe, G. Kehr, G. Erker, B. Rieger, *Dalton Trans.* **2006**, 459.

[3] a) H. D. Kaesz (Ed.) *Inorganic Syntheses*, Wiley, New York, **1989**; b) R. Uson, A. Laguna, M. Laguna, D. A. Briggs, H. H. Murray, J. P. Fackler in *Inorganic Syntheses* (Ed.: H. D. Kaesz), Wiley, New York, **1989**, pp. 85–91.

[4] M. Gil-Moles, S. Türck, U. Basu, A. Pettenuzzo, S. Bhattacharya, A. Rajan, X. Ma, R. Büssing, J. Wölker, H. Burmeister et al., *Chem. Eur. J.* **2021**, *27*, 17928.

[5] R. Jothibasu, H. V. Huynh, L. L. Koh, *J. Organomet. Chem.* **2008**, *693*, 374.

[6] G. M. Sheldrick, *Acta Cryst.* **2008**, *A64*, 112.

[7] G. M. Sheldrick, *Acta Cryst.* **2015**, *A71*, 3.

[8] O. V. Dolomanov, L. J. Bourhis, R. J. Gildea, J. A. K. Howard, H. Puschmann, *J. Appl. Cryst.* **2009**, *42*, 339.

[9] G. M. Sheldrick, *Acta Cryst.* **2015**, *C71*, 3.

[10] R. L. Cordiner, A. F. Hill, J. Wagler, *Organometallics* **2009**, *28*, 4880.

[11] A. D. Becke, *J. Chem. Phys.* **1993**, *98*, 5648.

[12] C. Lee, W. Yang, R. G. Parr, *Phys. Rev. B* **1988**, *37*, 785.

[13] R. Krishnan, J. S. Binkley, R. Seeger, J. A. Pople, *J. Chem. Phys.* **1980**, *72*, 650.

[14] A. D. McLean, G. S. Chandler, *J. Chem. Phys.* **1980**, *72*, 5639.

[15] M. J. Frisch, G. W. Trucks, H. B. Schlegel, G. E. Scuseria, M. A. Robb, J. R. Cheeseman, G. Scalmani, V. Barone, G. A. Petersson, H. Nakatsuji, X. Li, M. Caricato, A. V. Marenich, J. Bloino, B. G. Janesko, R. Gomperts, B. Mennucci, H. P. Hratchian, J. V. Ortiz, A. F. Izmaylov, J. L. Sonnenberg, D. Williams-Young, F. Ding, F. Lipparini, F. Egidi, J. Goings, B. Peng, A. Petrone, T. Henderson, D. Ranasinghe, V. G. Zakrzewski, J. Gao, N. Rega, G. Zheng, W. Liang, M. Hada, M. Ehara, K. Toyota, R. Fukuda, J. Hasegawa, M. Ishida, T. Nakajima, Y. Honda, O. Kitao, H. Nakai, T. Vreven, K. Throssell, J. A. Montgomery, Jr., J. E. Peralta, F. Ogliaro, M. J. Bearpark, J. J. Heyd, E. N. Brothers, K. N. Kudin, V. N. Staroverov, T. A. Keith,

R. Kobayashi, J. Normand, K. Raghavachari, A. P. Rendell, J. C. Burant, S. S. Iyengar, J. Tomasi, M. Cossi, J. M. Millam, M. Klene, C. Adamo, R. Cammi, J. W. Ochterski, R. L. Martin, K. Morokuma, O. Farkas, J. B. Foresman, D. J. Fox, *Gaussian 16, Revision A.03*, Gaussian, Inc., Wallingford CT, **2016**.

[16] K. L. Schuchardt, B. T. Didier, T. Elsethagen, L. Sun, V. Gurumoorthi, J. Chase, J. Li, T. L. Windus, *J. Chem. Inf. Model.* **2007**, *47*, 1045.

[17] B. P. Pritchard, D. Altarawy, B. Didier, T. D. Gibson, T. L. Windus, *J. Chem. Inf. Model.* **2019**, *59*, 4814.

[18] D. Feller, *J. Comput. Chem.* **1996**, *17*, 1571.

[19] F. Brylak, P. Löwe, K. Wurst, S. Hohloch, F. Dielmann, *Inorg. Chem. Front.* **2025**, *12*, 7556.

[20] *Chemcraft - graphical software for visualization of quantum chemistry computations. Version 1.8, build 682,* can be found under https://www.chemcraftprog.com.

[21] a) R. Ditchfield, W. J. Hehre, J. A. Pople, *J. Chem. Phys.* **1971**, *54*, 724; b) W. J. Hehre, R. Ditchfield, J. A. Pople, *J. Chem. Phys.* **1972**, *56*, 2257; c) P. C. Hariharan, J. A. Pople, *Theoret. Chim. Acta* **1973**, *28*, 213; d) T. Clark, J. Chandrasekhar, G. W. Spitznagel, P. V. R. Schleyer, *J. Comput. Chem.* **1983**, *4*, 294; e) M. S. Gordon, J. S. Binkley, J. A. Pople, W. J. Pietro, W. J. Hehre, *J. Am. Chem. Soc.* **1982**, *104*, 2797; f) G. W. Spitznagel, T. Clark, P. von Ragué Schleyer, W. J. Hehre, *J. Comput. Chem.* **1987**, *8*, 1109; g) M. M. Francl, W. J. Pietro, W. J. Hehre, J. S. Binkley, M. S. Gordon, D. J. DeFrees, J. A. Pople, *J. Chem. Phys.* **1982**, *77*, 3654.

[22] S. Grimme, J. Antony, S. Ehrlich, H. Krieg, *J. Chem. Phys.* **2010**, *132*, 154104.

[23] S. Ullrich, B. Kovačević, X. Xie, J. Sundermeyer, *Angew. Chem. Int. Ed.* **2019**, *58*, 10335.

[24] M. Witt, H.-F. Grützmacher, *Int. J. Mass Spectrom. Ion Processes* **1997**, *164*, 93.

[25] F. Neese, *WIREs Comput Mol Sci* **2012**, *2*, 73.

[26] a) F. Neese, *WIREs Comput Mol Sci* **2025**, *15*; b) B. Helmich-Paris, B. de Souza, F. Neese, R. Izsák, *J. Chem. Phys.* **2021**, *155*, 104109; c) R. Izsák, A. Hansen, F. Neese, *Mol. Phys.* **2012**, *110*, 2413; d) R. Izsák, F. Neese, *J. Chem. Phys.* **2011**, *135*, 144105; e) R. Izsák, F. Neese, W. Klopper, *J. Chem. Phys.* **2013**, *139*, 94111; f) F. Neese, *Chem. Phys. Lett.* **2000**, *325*, 93; g) F. Neese, *J Comput Chem* **2003**, *24*, 1740; h) F. Neese, *WIREs Comput Mol Sci* **2018**, *8*; i) F. Neese, *WIREs Comput Mol Sci* **2022**, *12*; j) F. Neese, *J Comput Chem* **2023**, *44*, 381; k) F. Neese, F. Wennmohs, U. Becker, C. Riplinger, *J. Chem. Phys.* **2020**, *152*, 224108; l) F. Neese, F. Wennmohs, A. Hansen, U. Becker, *Chem. Phys.* **2009**, *356*, 98; m) R. C. Sabando, C. Riplinger, F. Wennmohs, F. Neese, G. Bistoni, *J. Chem. Theory Comput.* **2025**, *21*, 7920.

[27] a) F. M. Bickelhaupt, C. Fonseca Guerra, M. Mitoraj, F. Sagan, A. Michalak, S. Pan, G. Frenking, *Phys. Chem. Chem. Phys.* **2022**, *24*, 15726; b) M. Francis, S. Roy, *ACS omega* **2022**, *7*, 5730; c) G. Frenking, F. Matthias Bickelhaupt in *The chemical bond* (Eds.: G. Frenking, S. Shaik), Wiley-VCH, Weinheim, **2014**, pp. 121–157; d) L. Zhao, M. von Hopffgarten, D. M. Andrada, G. Frenking, *WIREs Comput Mol Sci* **2018**, *8*.

[28] T.-F. Leung, D. Jiang, M.-C. Wu, D. Xiao, W.-M. Ching, G. P. A. Yap, T. Yang, L. Zhao, T.-G. Ong, G. Frenking, *Nat. Chem.* **2021**, *13*, 89.

[29] a) G. Schaftenaar, J. H. Noordik, *J. Comput. Aided Mol. Des.* **2000**, *14*, 123; b) G. Schaftenaar, E. Vlieg, G. Vriend, *J. Comput. Aided Mol. Des.* **2017**, *31*, 789.
